# Supplementary material for: Hospital bed capacity and usage across secondary healthcare providers in England during the first wave of the COVID-19 pandemic: a descriptive analysis
Source: BMJ Open. 2021 Jan 26;11(1):e042945. doi: 10.1136/bmjopen-2020-042945 (PMC7843315; doi:10.1136/bmjopen-2020-042945)
Supplement: Supplementary data [file bmjopen-2020-042945supp001.pdf]

Supplementary Material

Supplementary Methods

Representativeness of Sample
Using the 2nd of May (randomly chosen) as an exemplar date, the non-specialist acute trusts to which we have restricted this survey represented 6,359 of the 6,866 beds (i.e. 92.6%) compatible with mechanical ventilation across England (comprising all institutions reporting to SitRep). Similarly, for all bed types, our sample represents 92.4% (i.e. 98,882 or the total 106,981 across England).

Organizational Units of Healthcare Provision
Although hospitals are relatively self-explanatory, the remaining units may require further context for readers unfamiliar with the organization structure utilized in the UK, as such, the following are brief summaries of the higher-order units of healthcare provision. Trusts are the core functional unit of hospital-based (i.e. secondary) care provision in England. They represent the first-level of aggregation above individual sites/hospitals, i.e. a trust is a collection of 1 or more geographically co-located hospitals which for specific administrative reasons operate as a single entity, although individual hospitals retain differing degrees of financial and operational autonomy depending on the specific trust structure. STPs are the aggregated unit of trusts, in combination with other units of healthcare provision, such as Clinical Commissioning Groups which administer the portion of the healthcare budget allocated to a specific geographic locale. There are 42 STPs each mapped to a specific health-geography ‘footprint’, and their express purpose was to deliver improvements pertaining to efficacy of services and integration of geographically co-located care providers.[35] The STPs are then mapped to 7 distinct geographical regions across England.

COVID-19 Status Recording
Data reported with reference to COVID-19 status, for example the number of general and acute beds occupied by individuals with the infection, refers to those whom had a confirmed positive result from a reverse transcriptase polymerase chain reaction (PCR) of nasopharyngeal and/or oropharyngeal swab.[36\] PCR was the only available testing method during the study period. Although national testing policy changed throughout the study period, all people for whom there was a suspicion of COVID-19 infection and who were admitted to hospital were tested, and potentially re-tested multiple times if the initial results were negative but clinical suspicion remained high (recorded as suspected COVID; for the purposes of the subsequent analysis ‘confirmed’ and ‘suspected’ were treated as one group due to the relatively small numbers reported for the latter).

Historical Baselines for Bed Availability
Baseline data comprised: 1) the average number of general and acute (G&A) beds available between January-March 2020, sourced from previously published routine situation report (SitRep) data;[37] 2) the number of critical care beds prior to the first reported case of COVID-19 in the UK, i.e. the value reported on the 30th January 2020 Critical Care SitRep,[38] and similarly to previous modelling studies was used for the baseline availabilities of HDU / ITU, critical care and ventilated beds;[30] 3) the maximum theoretical capacity for field hospitals was based on official government press releases;[39,40] and, 4) independent sector provider baseline capacity extracted from appendix 1 of the NHS England documentation confirming the 14 week block contract with the Independent Healthcare Providers Network.[28] Baselines were available for all of the trusts from the sources mentioned above and was propagated through into the STP and Regional datasets alongside aggregation of other values. Baseline bed numbers were not available for site level data. The choice of the period prior to the first wave of the pandemic instead of the historical baseline from 12 months prior was informed by two important piece of information: 1) the UK has experienced a gradual downward trend in bed numbers [1], and thus to be able to use the comparable period from 2019 we would have required an adjustment for that trend to produce a realistic baseline (there was a chance that we would have hypothesized there being more beds than were created after the first few weeks of mobilization by over-estimating the baseline number without this correction); 2) we deemed that use of the exact number of beds available at the time of operational planning (i.e. in February/early march) had greater ecological validity, as this was about reflecting the change from what we know was available rather than an abstracted version of what might have existed relative to similar periods in previous years.

Quality Control
All of the data was acquired through the daily site reports provided by NHS-Improvement & NHS England. These reports were loaded and appended sequentially with checks to ensure consistency in headings and data composition. The data spanned multiple sheets; these sheets were joined using Hospital, Trust and STP level

codes where appropriate. In some cases it was necessary to resort to using site names where no codes were present on the sheets containing hospital-level information regarding general and acute and critical care bed availability and occupancy. It was immediately apparent that extracting comprehensive site-level data from these records was non-trivial and for reasons discussed later, we maintain two datasets moving forward: one at site-level and one at trust-level that is used to aggregate to STP, Regional and Total figures as well.

Bed availability and total occupancy was recorded directly for G&A and critical care beds, alongside percentages of covid-confirmed occupants allowing for the calculation of a covid / non-covid / unoccupied breakdown for the G&A beds *only* (due to discrepancies in the definition of HDU / ITU and critical care beds, the percentage occupancy for critical care beds often resulted in impossible values of over 100%; it was decided to forego calculating a covid-breakdown for these beds due to how prolific these inconsistencies and issues were). For all of the other bed types, data was recorded in a different way. The number of covid positive patients (and in some cases covid-suspected patients), non-covid patients and the remaining unoccupied beds were recorded, allowing for total occupancy and availability to be calculated through simple transformation of these columns.

There are two key dates and several more minor milestones in the period we have data for (26th March to 5th June) where significant, non-trivial changes occurred in the site report structure and content. Prior to April 1st there was no information on bed availability beyond G&A and Critical Care beds; only the number of covid-positive patients were recorded for each type of bed. After the 1st of April, more granular bed availability was provided along with the means to work out the covid/non-covid breakdown of occupancy for Ventilated beds. From the 27th April onwards similar breakdowns and availability were recorded for HDU / ITU, IDU and most other types of bed at a site-level.

After loading in the data and accounting for the above described changes to its composition, the trust-level data used for the majority of our analysis had:

- 8.7% of Ventilated bed non-covid and unoccupied numbers missing across all records (no missing records for covid occupancy)
- No missing records for G&A, Critical care bed availability and occupancy
- No missing records for HDU / ITU after April 26th, otherwise 45.0%
- All other columns containing information regarding the hospital, trust, etc. were complete

Both datasets were filtered to remove children’s hospitals, mental health hospitals and other sites / trusts that were not relevant to the analysis. STP linkage data was acquired via NHS Digital’s library of public datasets source: (https://digital.nhs.uk/services/organisation-data-service/data-downloads/other-nhs-organisations) and augmented to include populations within STPs to facilitate our “beds-per-capita” figures (values were scrapped manually from the NHS England website, source: https://www.england.nhs.uk/integratedcare/stps/view-stps/). It was found that 7 trust codes were duplicated across 2 STPs; it was inappropriate to double count them so they were arbitrarily assigned to one of the STPs. The following table contains the STP and Trust code pairs that were chosen / removed from the linkage data to ensure a one-to-one mapping:

STable 1: STP and trust code pairs (for duplicated trusts)

Trust Code	STP Code (Assigned)	STP Code (Removed)
RDU	QRL	QNQ
RFS	QJ2	QF7
RK9	QJK	QT6
RMC	QOP	QHM
RNN	QE1	QHM
RVR	QXU	QWE
RVY	QYG	QE1

Additionally, due to some trust-level mergers that took place and missing data in the source, 4 updated STP-Trust pairs were manually added to the linkage data to facilitate their inclusion in the analysis (source:

https://www.england.nhs.uk/integratedcare/stps/view-stps/).

STable 2: Updated Trust-STP pairs (for merged trusts)

Trust Code	STP Code
RBA	QSL
RA3	QUY
RTP	QNX

Finally, it was found that two STPs spanned two regions. It was decided that QHM should fall under the North West region (all but one of its trusts are in that region) and QF7 should fall under the Midlands region (all but one of its trusts are in that region). The region definitions are inferred from the regions assigned to each trust in the site reports making up our primary dataset.

Despite our best efforts there were some missing values that persisted in critical columns outside of the key milestones mentioned in the section above. Moreover, in preparing the data it was noted that on several occasions there were substantial and improbable changes in the number of available beds that lasted 24 hours (even after allowing for the weekly trend of cyclical fluctuations in beds availability), prior to reversion to a value that fit the overall trend. These outliers follow from the reasonable assumption of the presence of data entry errors; it was decided that a cleaning rule should be applied to the data to avoid these seemingly impossible daily fluctuations and outliers.

First, a rolling median centred on each record was calculated using the 5 applicable days surrounding the record (smaller windows used at extremities of the data with correction not being possible at its absolute extremes). Missing values as well as values deemed to be outliers (a change greater than the 95th percentile of all differences between each record and the centred median spanning five days around it) were replaced with the aforementioned rolling median values. Highly improbable fluctuations were filtered out and missing values could be imputed in a robust way. This imputation and outlier detection process was applied to every applicable bed column spanning every type contained in the data. Only after this cleaning took place were other columns created through transformation, e.g. the number of available ventilated beds etc. The effect of cleaning the data is shown below in a before and after comparison, 4 trusts were chosen for their high initial volatility in G&A bed occupancy (See SFigure 1 & SFigure 2).

Statistical Analysis Notes
Temporalized values, i.e. hospital-days, were calculated by multiplying the absolute number of each functional unit for which data was available, and the number of days for which data is available for each.

After cleaning the data, two more key issues had to be dealt with in the trust-level and site-level datasets respectively:

1. Due to the aforementioned trust-level mergers, the composition of the data changed slightly throughout its duration. In an effort to achieve consistency, we merged and coalesced records prior to each mergers' appearance in the data to match their state post-merger. I.e. any rows corresponding to trusts that were eventually merged into some other trust were merged consistently throughout the dataset, even before this change actually took place. This was applied to records for the trusts RQ8, RDD and RAJ which were merged to fall under the single code RAJ on April 1st. This was also applied to RC9 and RC1 merged into RC9, and RA7 and RA3 merged into RA7: mergers that also occurred on April 1st and were reflected in the data shortly after.
2. It was observed that in one of the sheets relied upon for ventilated bed numbers, separate rows were included for both the sites and the corresponding trusts (given a "catch-all" label as their organisation type rather than "site"). In cases where only one hospital was associated with a trust, the numbers for that hospital were sometimes - inconsistently - recorded in the catch-all row rather than the site row as was done fairly consistently across all other situations. To achieve consistency without losing significant portions of the site-level data, we coalesced those rows where only one site was present and the catch all row contained numbers whilst the site row had zeroes or missing values. In order to achieve this, the organisation types of the two rows were swapped so that the catch-all row would be used in place of the site row, such that the site code and name was consistent throughout the entirety of the data.

Data Limitations

One persistent concern was the formulas by which bed occupancy proportions were generated. For example, the COVID-19 specific G&A bed percentage-occupancy was initially calculated as the sum of COVID-19 patients in IDU (infectious disease unit) beds and COVID-19 patients in "any other beds" divided by the total number of available G&A beds. This eventually changed to being the sum of the number of mechanical ventilated beds, non-invasive ventilated beds, oxygen-supporting beds and "any other beds" occupied by COVID patients minus the number of HDU / ITU beds occupied by confirmed COVID patients, all divided by the total number of available G&A beds. Whilst this is not in-and-of-itself problematic, the nature of the "any other beds" item was deemed concerning by the authors.

To understand the aforementioned concern, we first need to explain the data specification in more detail. It was noted that columns of the form "Number of Covid-19 confirmed patients in … beds at 0800" did not seem to contain values consistent with "Number of … beds available, as at 08:00 (COVID)", which we expected to have mirrored values. Importantly, the latter set of columns did not contain an "any other bed" column. As such, the formula used by NHS-E in the above calculation of G&A bed proportions drew the "any other beds" value from the first set of data, whereas all of the other information was drawn from the latter columns as they were internally consistent. We acknowledge that the use of this formula could have introduced an error of unknown magnitude or direction (as the two versions of data reporting were not consistent). Similar issues were seen with the independent sector data as well.

SFigure 1: G&A Bed Availability Across the Most Volatile Trusts in Terms of G&A Bed Occupancy (Pre−Correction)

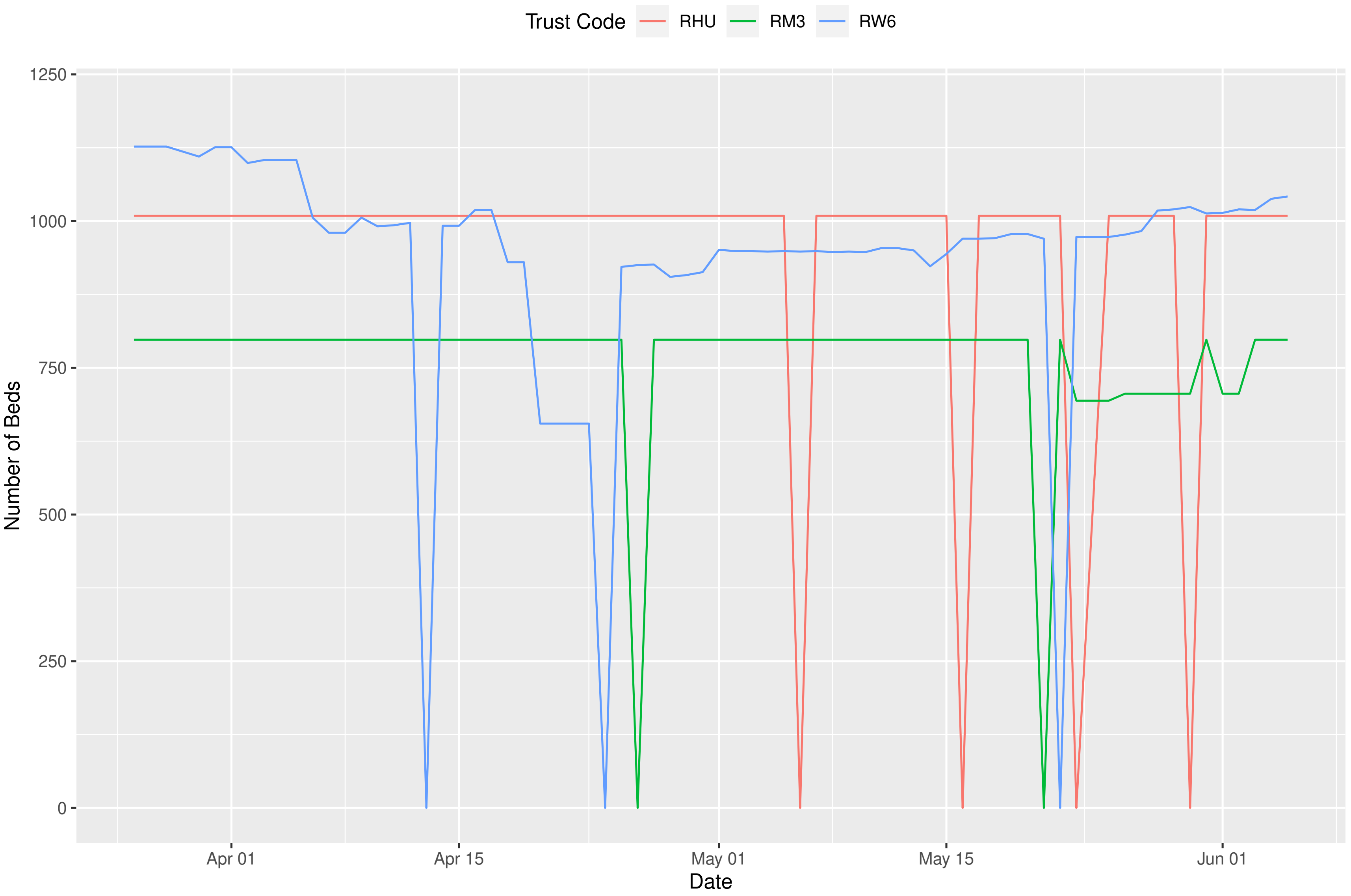

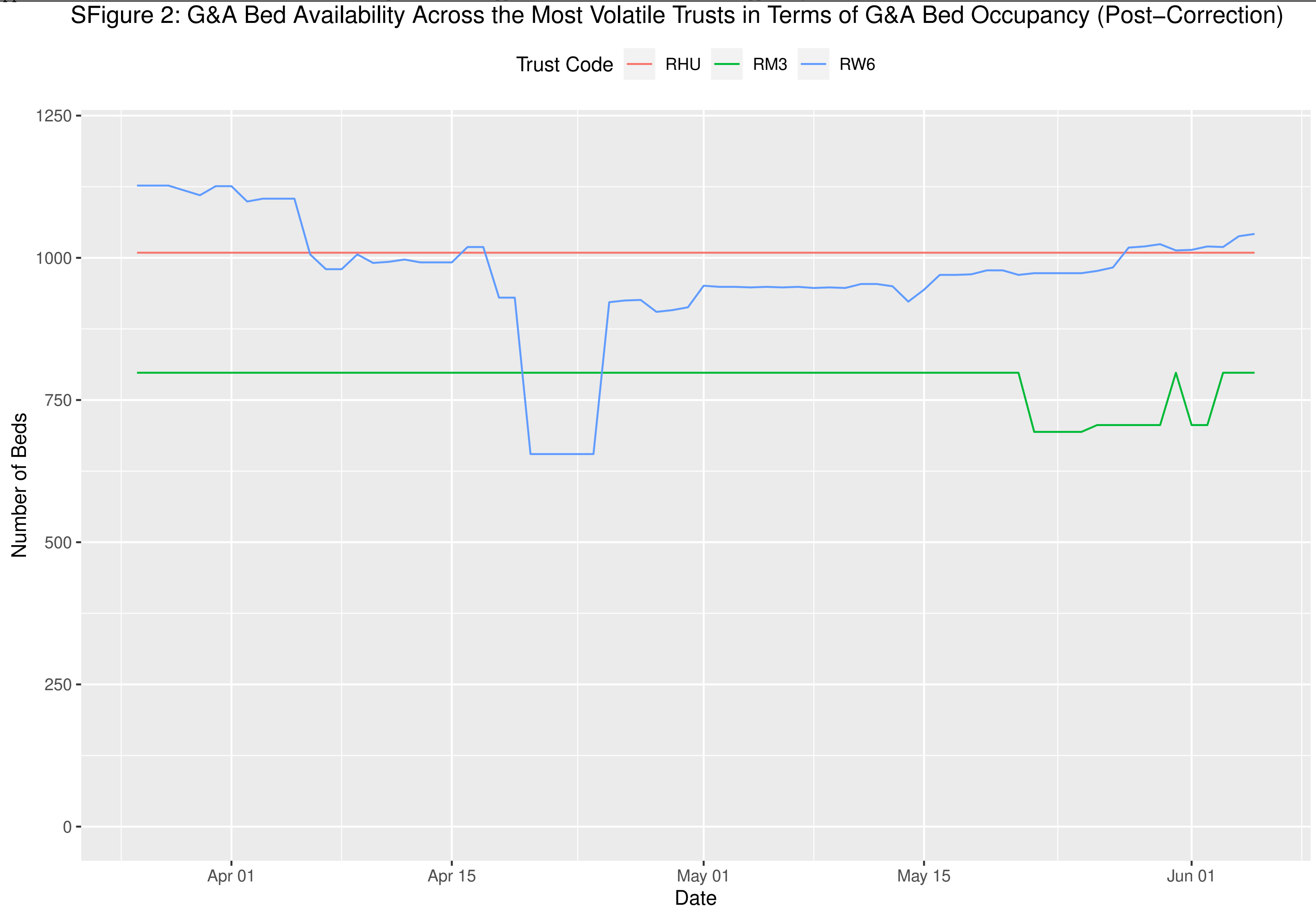
SFigure 2: G&A Bed Availability Across the Most Volatile Trusts in Terms of G&A Bed Occupancy (Post−Correction)
Trust Code
RHU
RM3
RW6
1250
1000
750
500
250
0
Number of Beds
Apr 01
Apr 15
May 01
May 15
Jun 01
Date

Supplementary Results

STable 3: Descriptive Summaries of the Size and Geographic Locations of Hospitals Stratified by the Peak Occupancy Achieved

	**Hospitals reaching 100% saturation of mechanical ventilation beds (n = 51*)**	**Hospitals reaching >92%, but not 100% saturation of mechanical ventilation bed (n = 20)**	**Hospitals reaching >85%, but not 92% saturation of mechanical ventilation bed (n = 19)**	**All other Hospitals (n = 77**)**
**Peak G&A bed capacity (Median [Range])**	438 [197 - 1012]	484 [256 - 841]	459 [253 - 910]	558 [44 - 1499]
**Peak mechanical ventilation compatible bed capacity (restricted to April 2nd onwards) (Median [Range])**	29 [7 - 77]	49 [18 - 141]	33 [15 - 153]	40 [7 - 159]
**Peak HDU/ITU bed capacity (restricted to April 27th onwards) (Median [Range])**	29 [0 - 99]	45 [17 - 161]	37 [14 - 157]	40 [6 - 152]
Location				
England (n = 167)	***51 (30.5%)***	***20 (12.0%)***	***19 (11.4%)***	***77 (46.1%)***
**London (n = 27)**	6 (22.2%)	11 (40.7%)	7 (25.9%)	3 (11.1%)
**Midlands (n = 27)**	11 (40.7%)	3 (11.1%)	1 (3.7%)	12 (44.4%)
**East of England (n = 20)**	7 (35.0%)	2 (10.0%)	1 (5.0%)	10 (50.0%)
**South West (n = 17)**	3 (17.6%)	0 (0.0%)	0 (0.0%)	14 (82.3%)
**South East (n = 24)**	6 (25.0%)	2 (8.3%)	3 (12.5%)	13 (54.2%)
**North East and Yorkshire (n = 29)**	8 (27.6%)	1 (3.4%)	4 (13.8%)	16 (65.5%)
**North West (n = 23)**	10 (43.5%)	1 (4.3%)	3 (13.0%)	9 (39.1%)

* One hospital is excluded from this n and the subsequent calculations as it does not have any information regarding G&A beds, despite reaching 100% capacity for mechanical ventilator bed capacity.
** 5 hospitals were excluded from this table as they had no ventilated beds at any time, or no data was available for their ventilated bed capacity in the dataset.

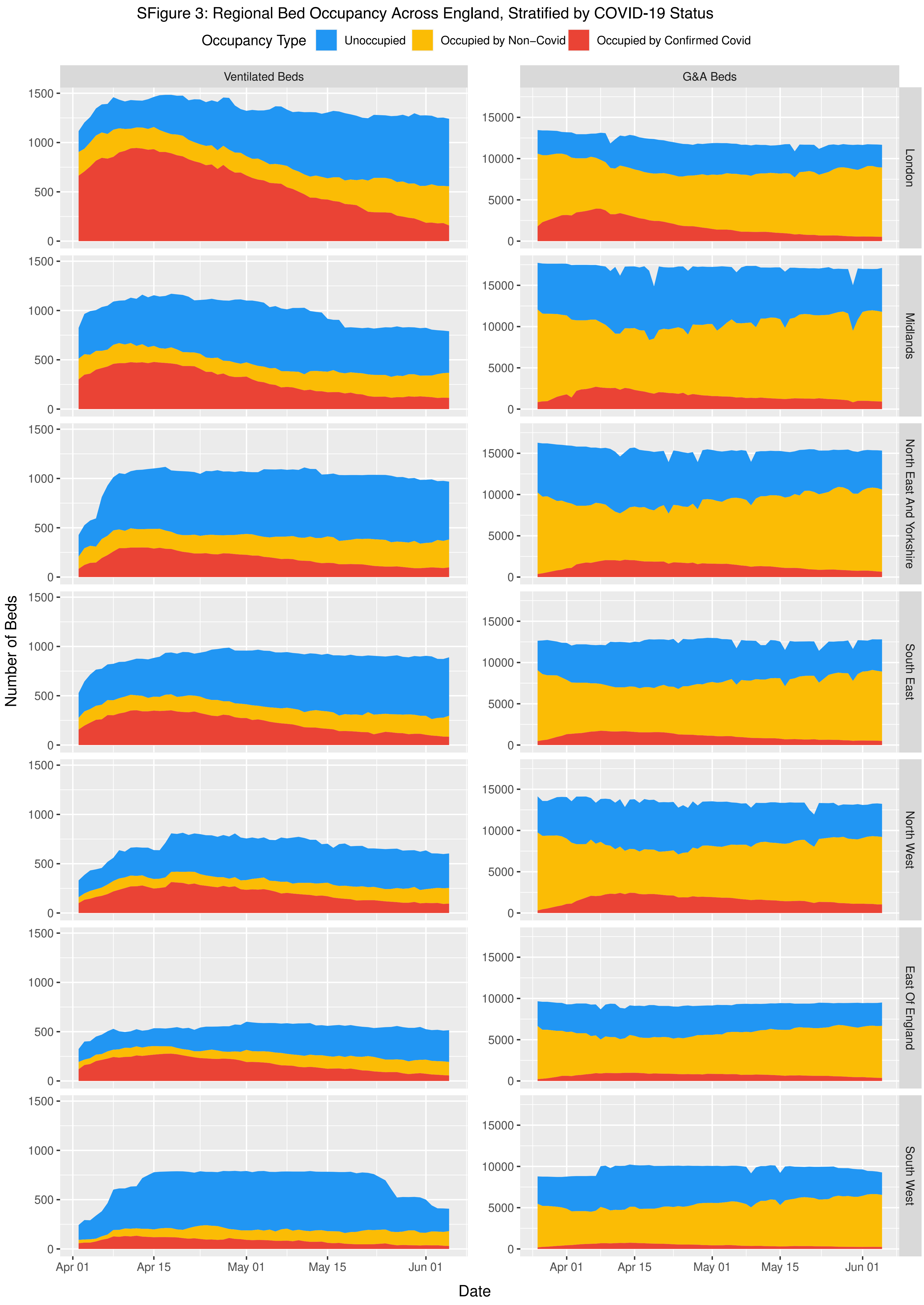
SFigure 3: Regional Bed Occupancy Across England, Stratified by COVID-19 Status
Occupancy Type
Unoccupied
Occupied by Non−Covid
Occupied by Confirmed Covid
Ventilated Beds
G&A Beds
London
Midlands
North East And Yorkshire
South East
North West
East Of England
South West
Number of Beds
Date
Apr 01
Apr 15
May 01
May 15
Jun 01
0
500
1000
1500
5000
10000
15000

SFigure 3: Bed Occupancy Across England by geographical region, stratified by COVID-19 Status

Legend: SFigure 3A (Left) illustrates the time-varying trends in mechanical ventilator bed capacity and occupancy across the 7 regions of England, from March 27th to June 5th; note that availability information is only present from 1st April onwards. SFigure 3B (Right) illustrates general and acute bed capacity and occupancy across the 7 regions of England, from 1st April to 5th June. Occupancy in both figures is stratified by whether the individual in the bed has a positive COVID-19 test or not).
Critical Care Beds

Critical Care Beds

In the context of surge capacity, at the site-level, 1558 hospital days (13.1%; median number of days per hospital = 10 [range: 1 to 65]) were at or above 85% of capacity, which corresponds to 120 hospitals spending at least 1 day at, or above, the aforementioned threshold. 948 hospital days (8.0%; median number of days per hospital = 6 [range: 1 to 51]) were spent above 92%, representing 102 hospitals. And 88 (50.9%) hospitals reached 100% capacity, representing 640 hospital days at saturation (median number of days per hospital = 5 [range: 1 to 51])). At the trust-level, 965 trust days (11.0%; median number of days per trust = 8 [range: 1 to 56]) were at or above 85% of capacity, representing 80 trusts. 567 trust days (6.5%; median number of days per trust = 5 [range: 1 to 47]) were spent above 92%, representing 64 trusts. And 47 (37.6%) trusts reached 100% capacity, representing 339 trust days at saturation (median number of days per trust = 5 [range: 1 to 21])). At the STP-level, 138 STP days (median number of days per STP = 5 [range: 1 to 43]) were at or above 85% of capacity, representing 18 STPs. 74 STP days (median number of days per STP = 2 [range: 1 to 26]) were spent above 92%, representing 14 STPs. And 6 STPs reached 100% capacity, representing 34 STP days at saturation (median number of days per STP = 3 [range: 1 to 19])). See SFigure 4 for a visual summary of these results. See SFigure 5 for the aggregate occupancy, stratified by COVID-19 status at the regional level.

In the context of baseline capacity, at the trust-level, 2620 trust days (22.1%; median number of days per trust = 27 [range: 1 to 69]) were at or above 100% capacity, which corresponds to 92 trusts spending at least 1 day at, or above, their-pre-pandemic baseline. 230 trusts days (median number of days per trust = 9 [range: 1 to 49]) were at or above 200% capacity, which corresponds to 19 trusts spending at least 1 day more than 100% above their-pre-pandemic baseline. At the STP-level, 620 STP days (median number of days per STP = 24 [range: 1 to 63]) were at an occupancy-level above 100% of baseline availability, which corresponds to 27 STPs spending at least 1 day at, or above, their-pre-pandemic baseline. 44 STP days (median number of days per STP = 14 [range: 10 to 20]) were at an occupancy-level above 200% of baseline availability which corresponds to 3 STPs spending at least 1 day more than 100% above their-pre-pandemic baseline. See SFigure 8 for a visual summary.

HDU/ITU Beds

The following results should be interpreted in the context of the date range available, i.e. data is only present after the 27th of April. Thus, the results are likely a significant underestimation of peak occupancy as, in retrospect, the peak number of cases and fatalities in the UK were near the beginning of April.

In the context of surge capacity, at the site-level, 315 hospital days (2.7%; median number of days per hospital = 2 [range: 1 to 39]) were at or above 85% of capacity, which corresponds to 59 hospitals spending at least 1 day at, or above, the aforementioned threshold. 216 hospital days (1.8%; median number of days per hospital = 1 [range: 1 to 39]) were spent above 92%, representing 45 hospitals. And 40 hospitals reached 100% capacity, representing 192 hospital days at saturation (median number of days per hospital = 1 [range: 1 to 39])). At the trust-level, 192 trust days (median number of days per trust = 3 [range: 1 to 39]) were at or above 85% of capacity, representing 36 trusts. 122 trust days (median number of days per trust = 2 [range: 1 to 39]) were spent above 92%, representing 24 trusts. And 19 trusts reached 100% capacity, representing 106 trust days at saturation (median number of days per trust = 2 [range: 1 to 39])). At the STP-level, 138 STP days (median number of days per STP = 3 [range: 1 to 43]) were at or above 85% of capacity, representing 18 STPs. 74 STP days (median number of days per STP= 2 [range: 1 to 26]) were spent above 92%, representing 14 STPs. And 6 STPs reached 100% capacity, representing 34 STP days at saturation (median number of days per STP = 3 [range: 1 to 19])). See SFigure 5 for a visual summary.

SFigure 4a: Number of Hospitals/Trusts/STPs at Varying Critical Care Bed Occupancy Levels Compared to Surge Capacity

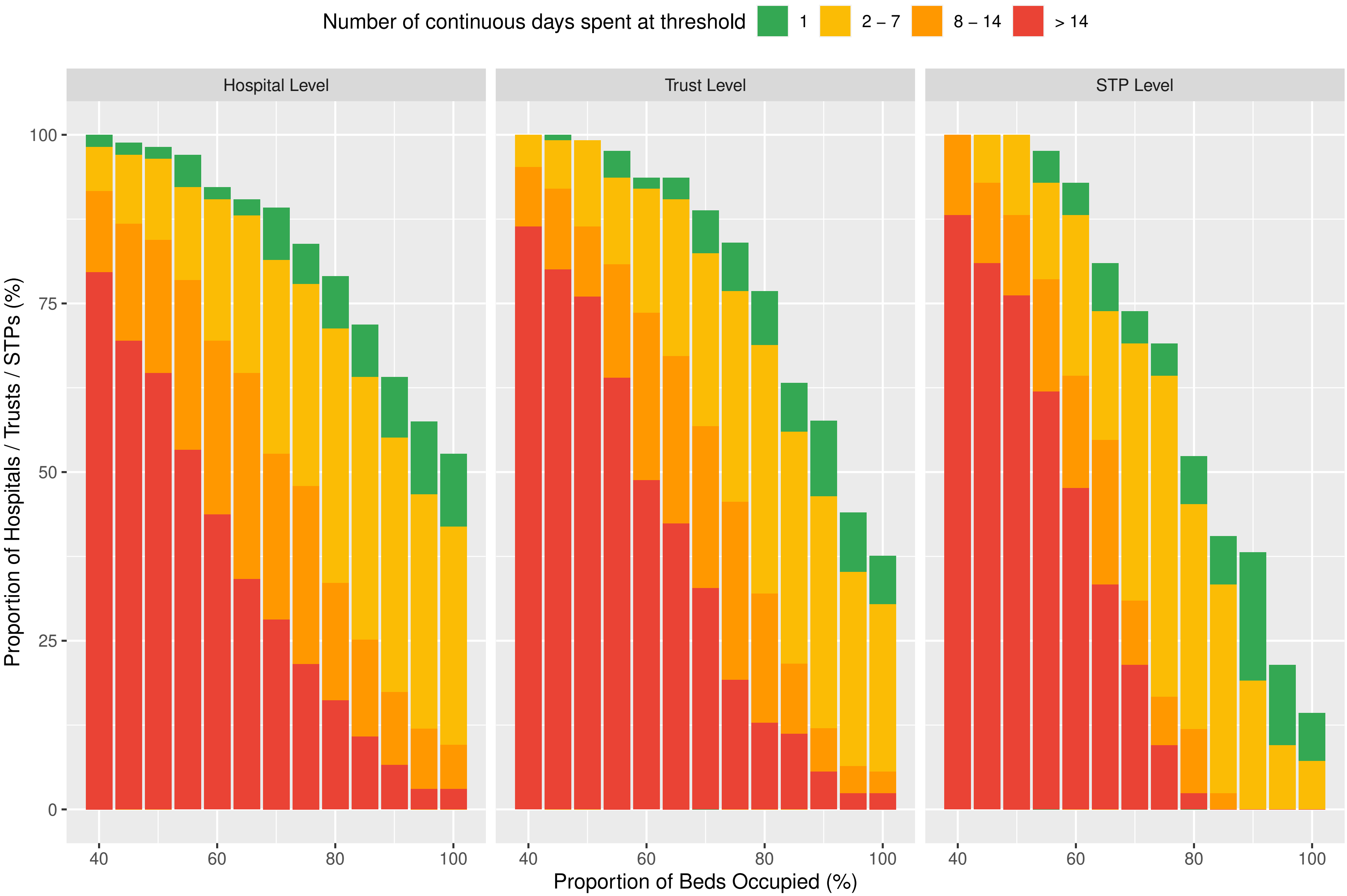

SFigure 4b: Number of Hospitals/Trusts/STPs at Varying HDU/ITU Bed Occupancy Levels Compared to Surge Capacity

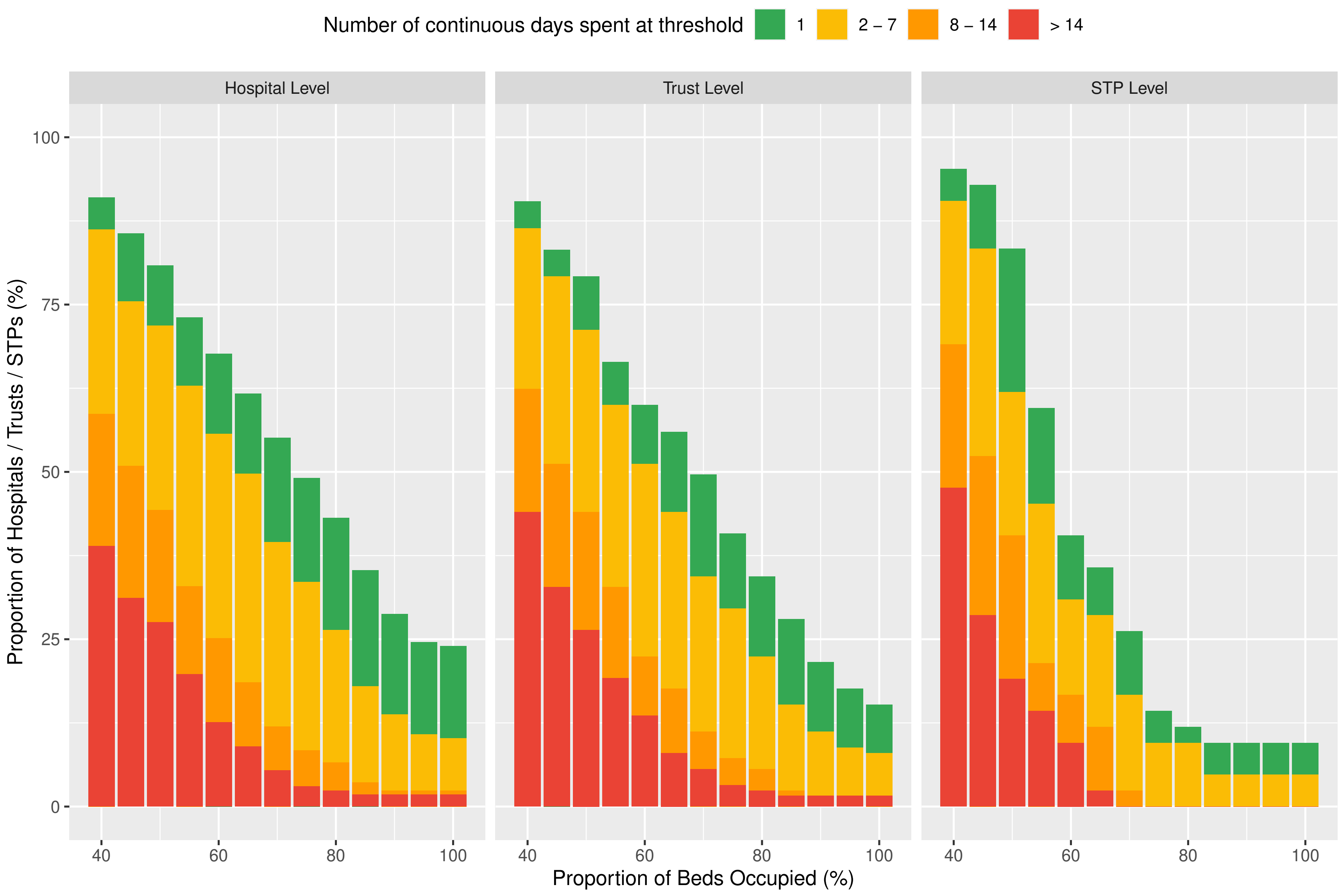

SFigure 4: Critical Care (Top) & HDU/ITU (Bottom) Occupancy (Based on Surge Capacities) Across England

Legend: SFigure 4A (Top) illustrates the proportion of hospitals/trusts/STPs at different occupancy thresholds for surge critical care bed capacity, across England, from April 1st to June 5th. SFigure 4B (Bottom) illustrates the proportion of hospitals/trusts/STPs at different occupancy thresholds for surge critical care bed capacity, across England, from April 1st to June 5th. The superimposed colours represent how long the trusts spent at each specific threshold.

SFigure 5: Number of Trusts/STPs at Varying G&A Bed Occupancy Levels Compared to Baseline Capacity

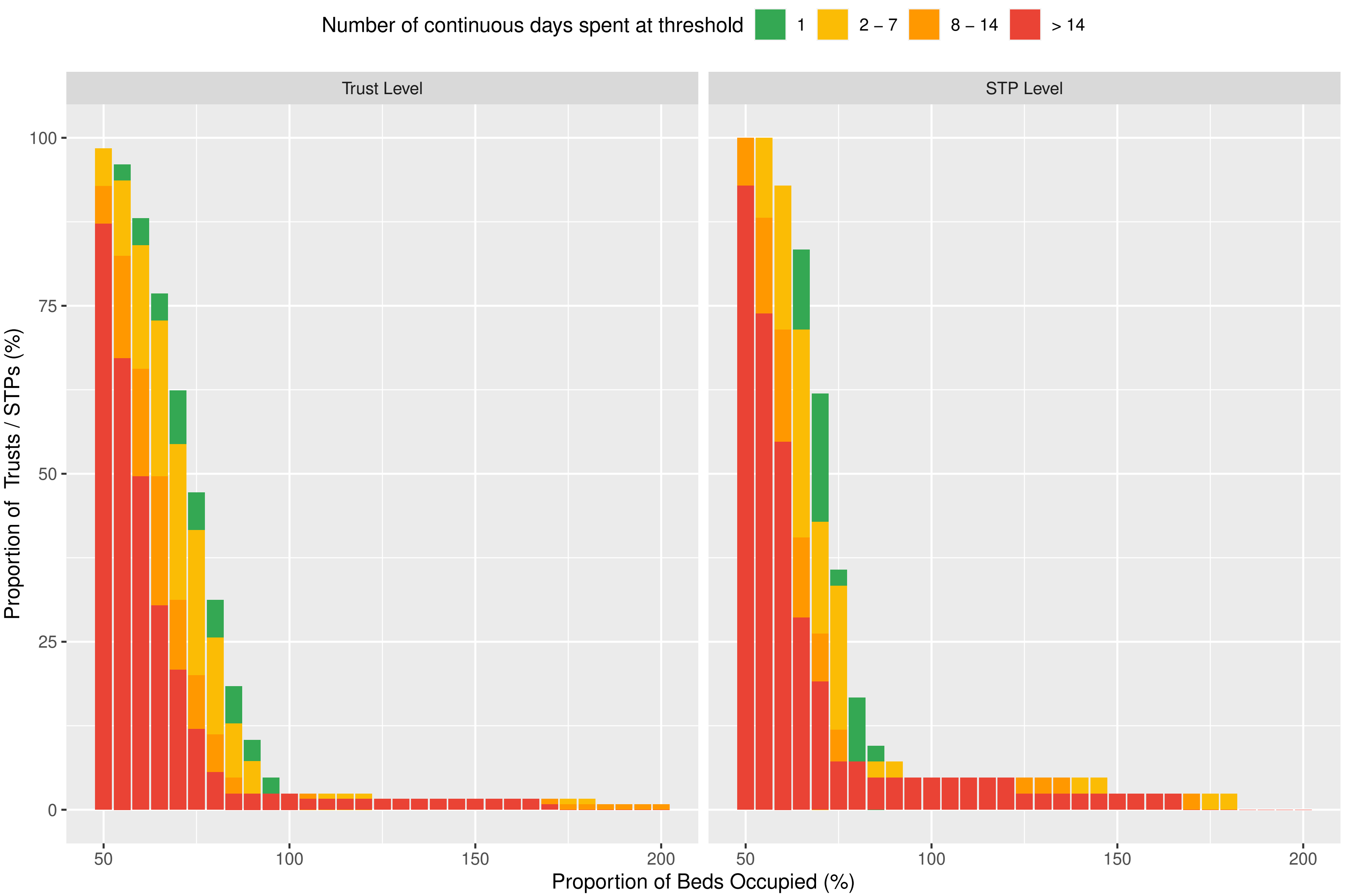

sFigure 5: Trust-Level General & Acute Bed Occupancy (Based on Baseline Capacities) Across England

Legend: The proportion of all trusts, and sustainability and transformation partnerships (STPs), at varying general and acute (G&A) bed occupancy thresholds relative to their baseline (mean availability January-March 2020) capacity, across England, from April 1st to June 5th. The superimposed colours represent how long the trusts spent at each specific threshold.

BMJ Publishing Group Limited (BMJ) disclaims all liability and responsibility arising from any reliance placed on this supplemental material which has been supplied by the author(s)

SFigure 6: Number of Trusts/STPs at Varying Ventilated Bed Occupancy Levels Compared to Baseline Capacity

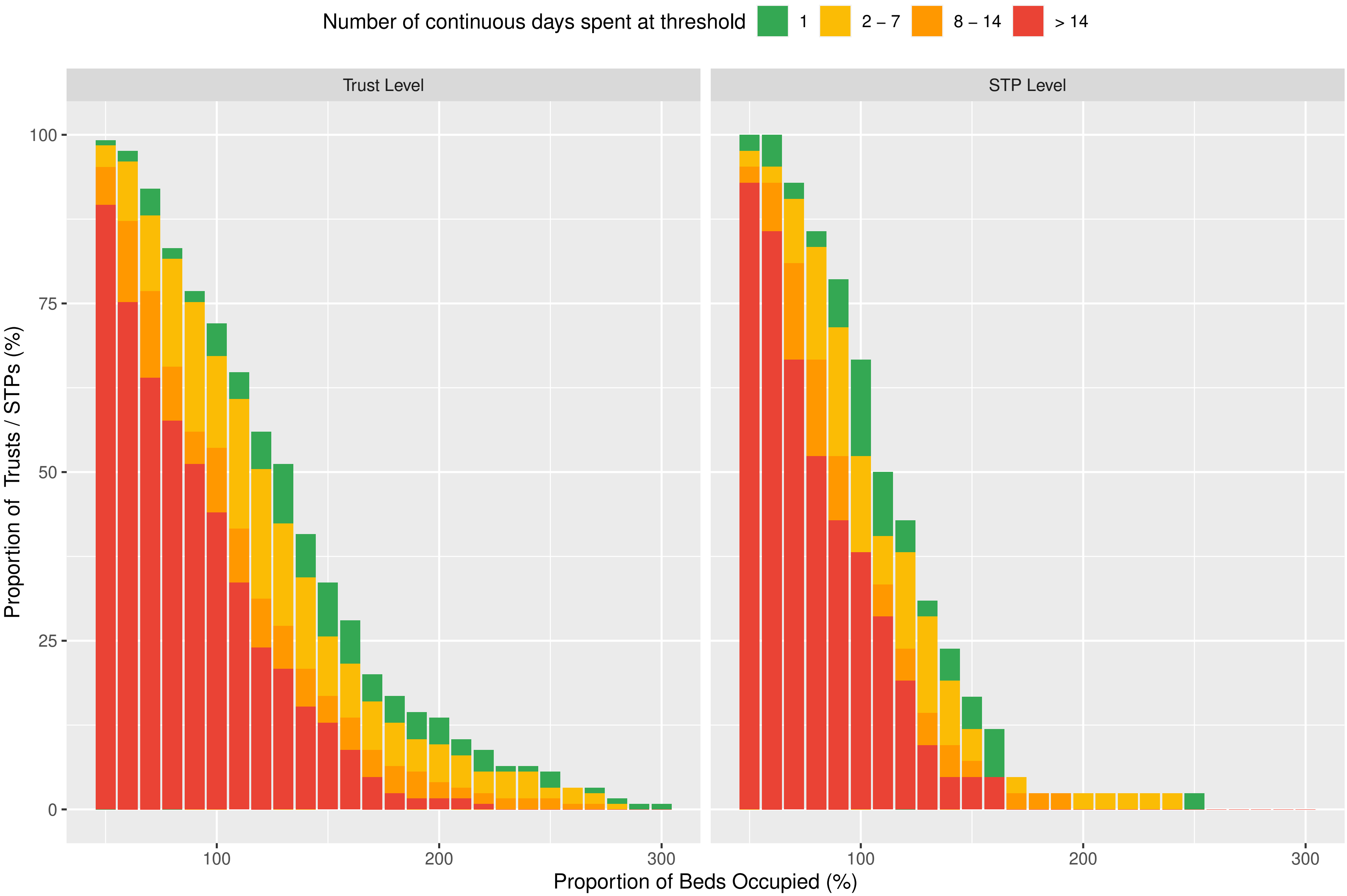

sFigure 6: Trust-Level Ventilator Bed Occupancy (Based on Baseline Capacities) Across England

Legend: The proportion of all trusts, and sustainability and transformation partnerships (STPs), at varying ventilator bed occupancy thresholds relative to their baseline capacity, across England, from April 1st to June 5th. The superimposed colours represent how long the trusts spent at each specific threshold.

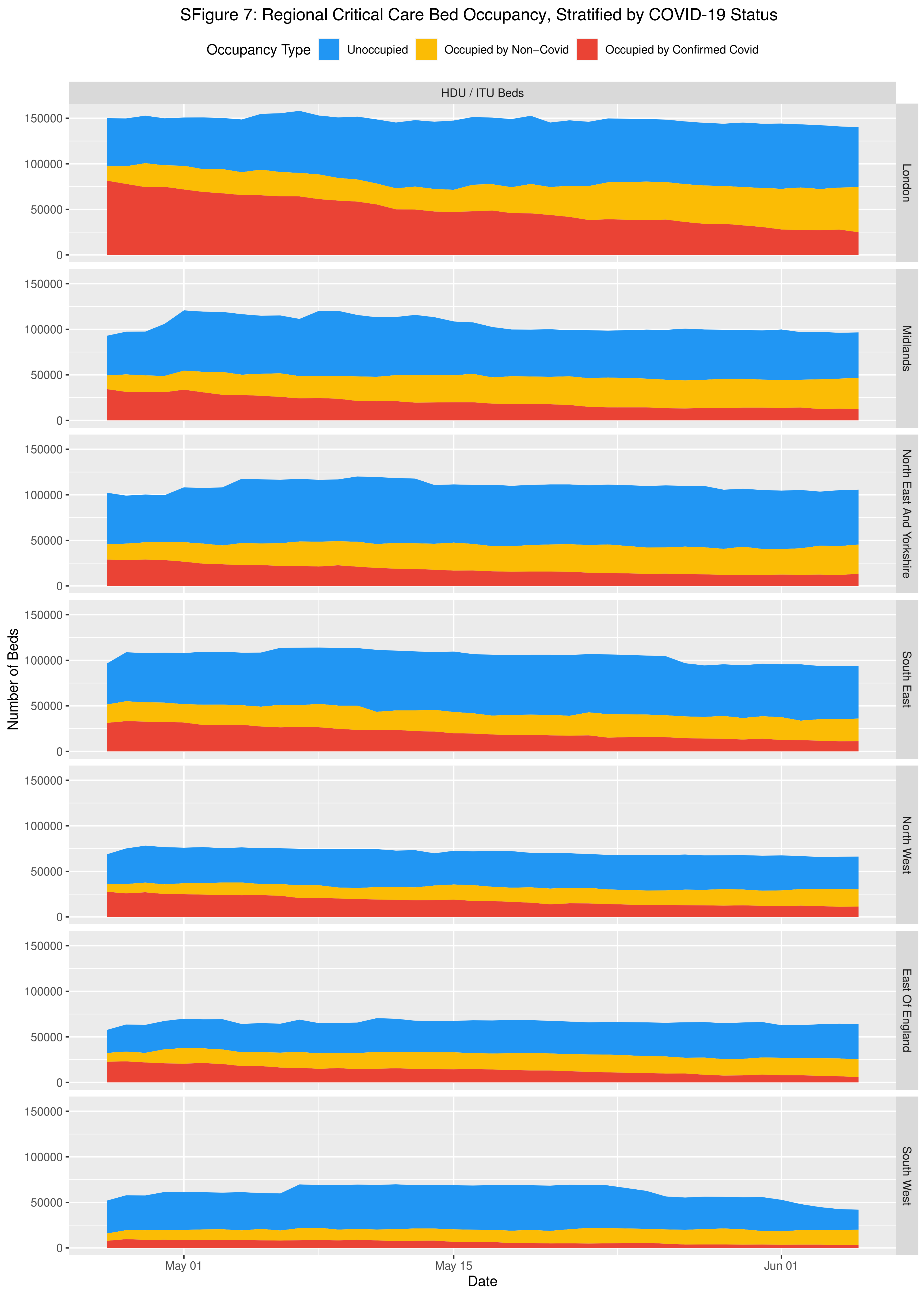
SFigure 7: Regional Critical Care Bed Occupancy, Stratified by COVID-19 Status
Occupancy Type
Unoccupied
Occupied by Non−Covid
Occupied by Confirmed Covid
HDU / ITU Beds
London
Midlands
North East And Yorkshire
South East
North West
East Of England
South West
Number of Beds
0
50000
100000
150000
May 01
May 15
Jun 01
Date

SFigure 7: Regional Critical Care Bed Occupancy, Stratified by COVID-19 Status

Legend: The time-varying trends in critical care bed capacity and occupancy across the 7 regions of England, from March 27th to June 5th. Occupancy is stratified by whether the individual in the bed has a positive COVID-19 test or not).

SFigure 8: Proportion of Trusts/STPs at Varying Critical Care Bed Occupancy Levels Compared to Baseline Capacity

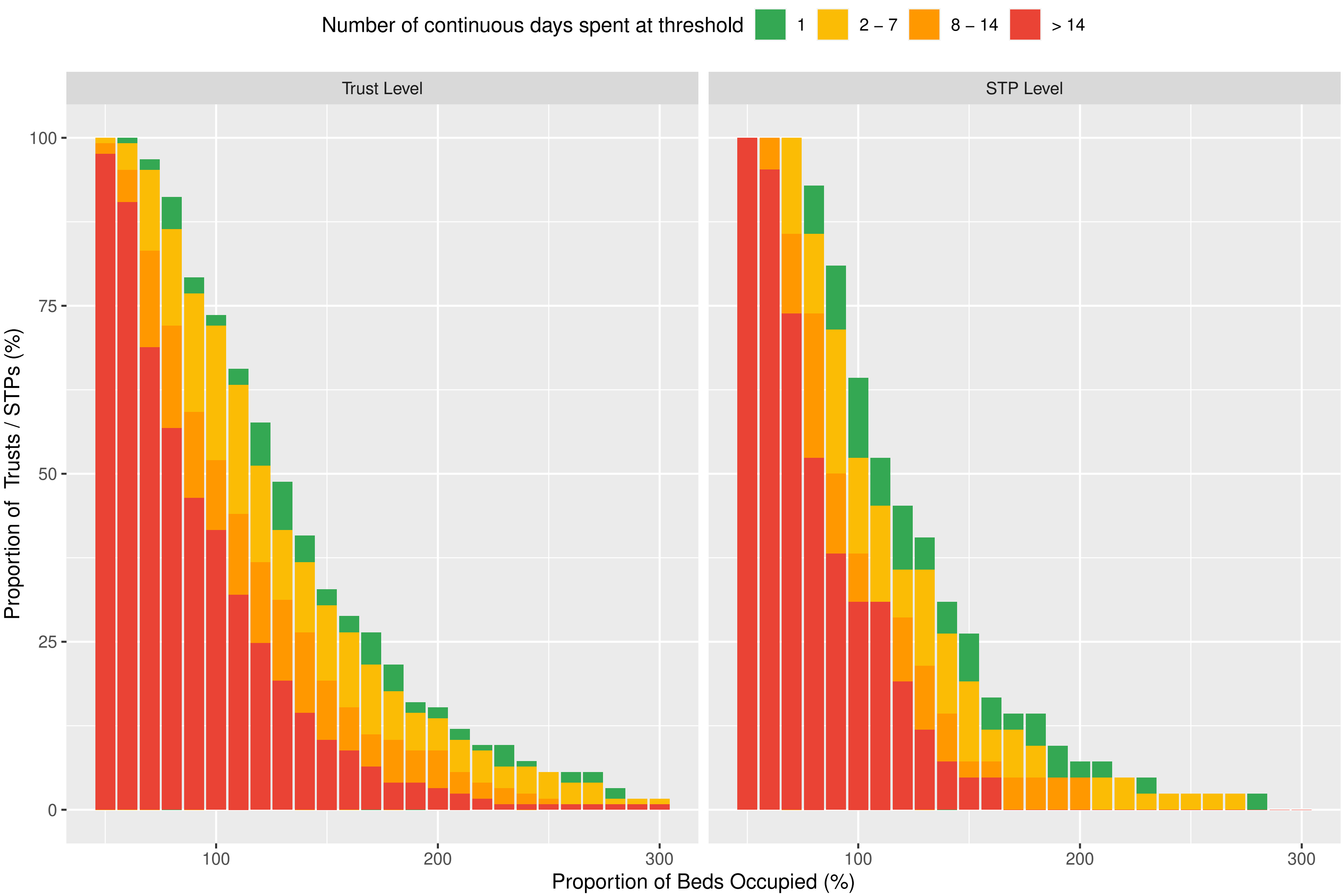

SFigure 8: Critical Care Bed Occupancy (Based on Baseline Capacities) Across England

Legend: SFigure 6A (Left) illustrates the proportion of trusts at different occupancy thresholds based on baseline critical care bed capacity, across England, from April 1st to June 5th. SFigure 6B (Right) illustrates the proportion of STPs at different occupancy thresholds based on their baseline critical care bed capacity, across England, from April 1st to June 5th. The superimposed colours represent how long the trusts spent at each specific threshold.

SFigure 9: Number of Trusts Operating Above Various Ventilated Bed Surge Capacity Thresholds

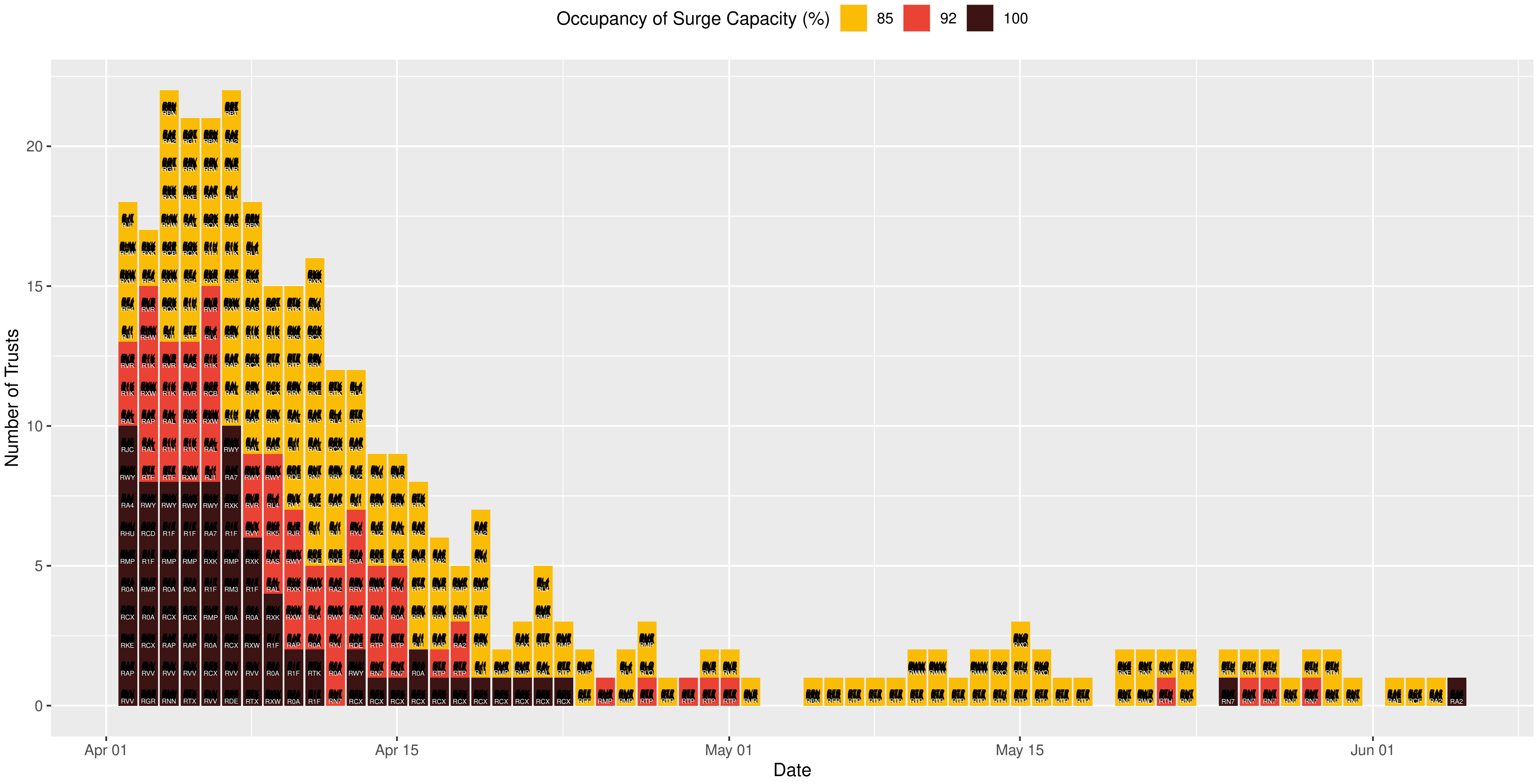

SFigure 9: Trust-Level Ventilator Bed Occupancy (Based on Surge Capacities) Across England

Legend: The conversion of trust code to name for all trusts included in the figure are: Manchester University NHS Foundation Trust (R0A), Isle Of Wight NHS Trust (R1F), Barts Health NHS Trust (R1H), London North West University Healthcare NHS Trust (R1K), Royal Surrey County Hospital NHS Foundation Trust (RA2), Yeovil District Hospital NHS Foundation Trust (RA4), University Hospitals Bristol NHS Foundation Trust (RA7), University Hospitals Bristol And Weston NHS Foundation Trust (RA7), Bradford Teaching Hospitals NHS Foundation Trust (RAE), Royal Free London NHS Foundation Trust (RAL), North Middlesex University Hospital NHS Trust (RAP), The Hillingdon Hospitals NHS Foundation Trust (RAS), Kingston Hospital NHS Foundation Trust (RAX), St Helens And Knowsley Teaching Hospitals NHS Trust (RBN), Mid Cheshire Hospitals NHS Foundation Trust (RBT), York Teaching Hospital NHS Foundation Trust (RCB), Harrogate And District NHS Foundation Trust (RCD), Airedale NHS Foundation Trust (RCF), The Queen Elizabeth Hospital, King's Lynn, NHS Foundation Trust (RCX), East Suffolk And North Essex NHS Foundation Trust (RDE), Barking, Havering And Redbridge University Hospitals NHS Trust (RF4), Barnsley Hospital NHS Foundation Trust (RFF), The Rotherham NHS Foundation Trust (RFR), West Suffolk NHS Foundation Trust (RGR), Cambridge University Hospitals NHS Foundation Trust (RGT), Portsmouth Hospitals NHS Trust (RHU), Royal Berkshire NHS Foundation Trust (RHW), Guy's And St Thomas' NHS Foundation Trust (RJ1), St George's University Hospitals NHS Foundation Trust (RJ7), South Warwickshire NHS Foundation Trust (RJC), Countess Of Chester Hospital NHS Foundation Trust (RJR), King's College Hospital NHS Foundation Trust (RJZ), Sherwood Forest Hospitals NHS Foundation Trust (RK5), Whittington Health NHS Trust (RKE), The Royal Wolverhampton NHS Trust (RL4), Wye Valley NHS Trust (RLQ), Salford Royal NHS Foundation Trust (RM3), Tameside And Glossop Integrated Care NHS Foundation Trust (RMP), Dartford And Gravesham NHS Trust (RN7), North Cumbria Integrated Care NHS Foundation Trust (RNN), Homerton University Hospital NHS Foundation Trust (RQX), Wrightington, Wigan And Leigh NHS Foundation Trust (RRF), University College London Hospitals NHS Foundation Trust (RRV), Northumbria Healthcare NHS Foundation Trust (RTF), Oxford University Hospitals NHS Foundation Trust (RTH), Ashford And St Peter's Hospitals NHS Foundation Trust (RTK), Surrey And Sussex Healthcare NHS Trust (RTP), University Hospitals Of Morecambe Bay NHS Foundation Trust (RTX), Epsom And St Helier University Hospitals NHS Trust (RVR), East Kent Hospitals University NHS Foundation Trust (RVV), Southport And Ormskirk Hospital NHS Trust (RVY), United Lincolnshire Hospitals NHS Trust (RWD), Warrington And Halton Hospitals NHS Foundation Trust (RWW), Calderdale And Huddersfield NHS Foundation Trust (RWY), Sandwell And West Birmingham Hospitals NHS Trust (RXK), Buckinghamshire Healthcare NHS Trust (RXQ), East Lancashire Hospitals NHS Trust (RXR), Shrewsbury And Telford Hospital NHS Trust (RXW), Imperial College Healthcare NHS Trust (RYJ)

BMJ Publishing Group Limited (BMJ) disclaims all liability and responsibility arising from any reliance placed on this supplemental material which has been supplied by the author(s)

SFigure 10a: Proportion of Hospitals/Trusts/STPs at Varying Ventilated Bed Occupancy Levels Compared to Surge Capacity

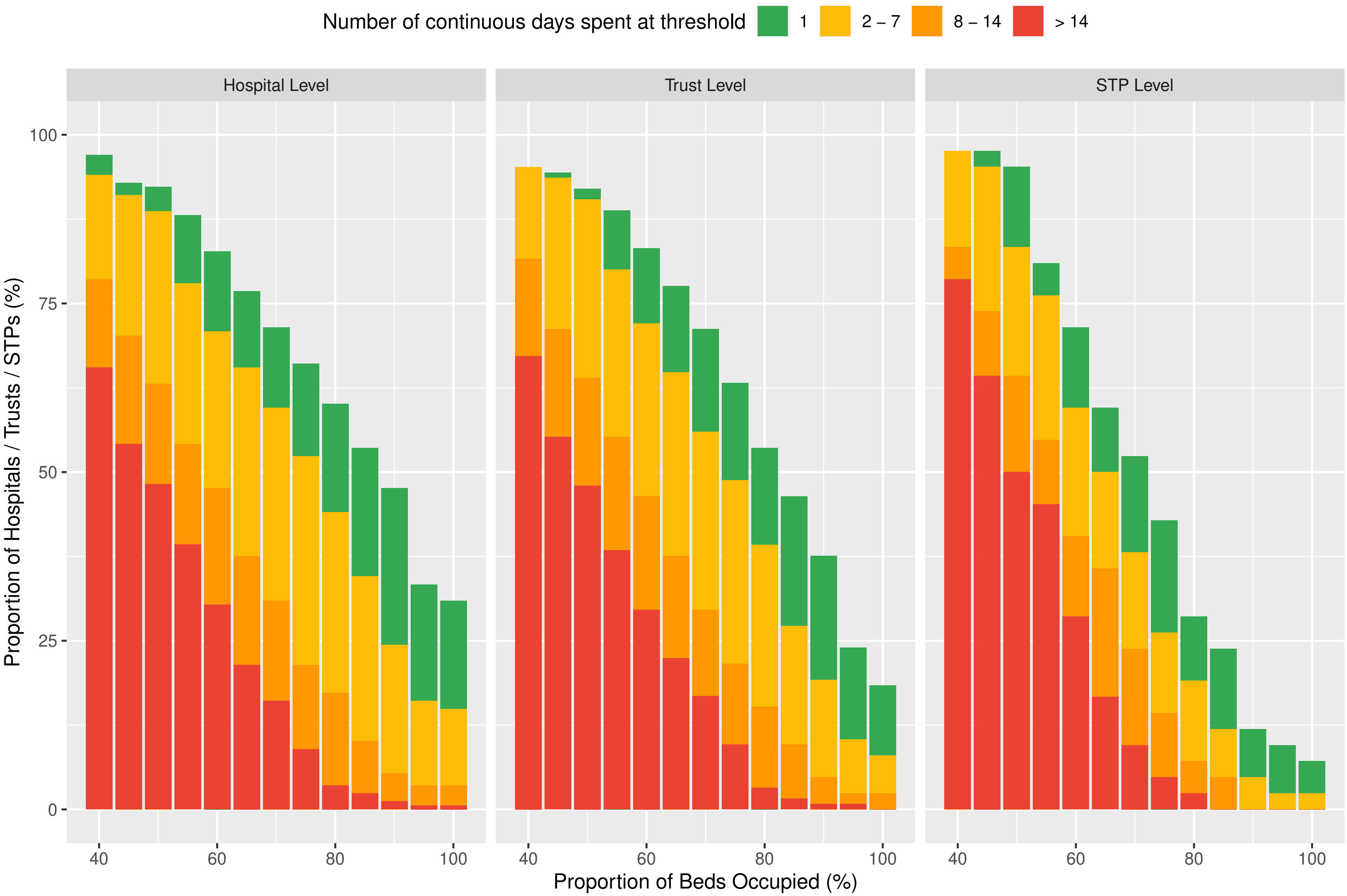

SFigure 10b: Proportion of Hospitals/Trusts/STPs at Varying G&A Bed Occupancy Levels Compared to Surge Capacity

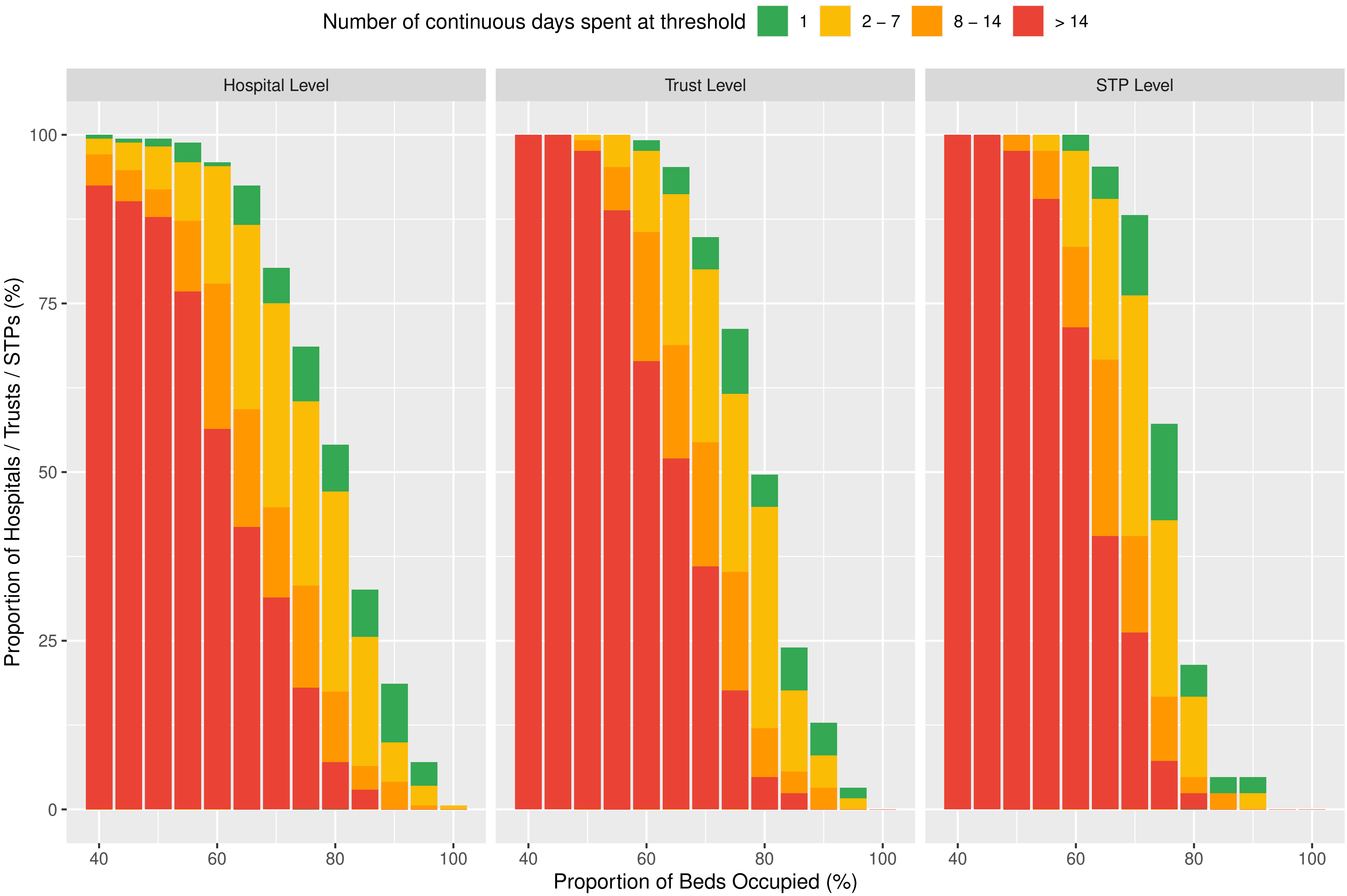

SFigure 10: Mechanical Ventilator Beds (Top) & General and Acute (Bottom) Occupancy (Based on Surge Capacities) Across England

Legend: SFigure 8A (Top) illustrates the proportion of STPs at different occupancy thresholds for surge mechanical ventilator bed capacity, across England. SFigure 8B (Bottom) illustrates the proportion of STPs at different occupancy thresholds for surge general and acute capacity, across England. The superimposed colours represent how long the trusts spent at each specific threshold.

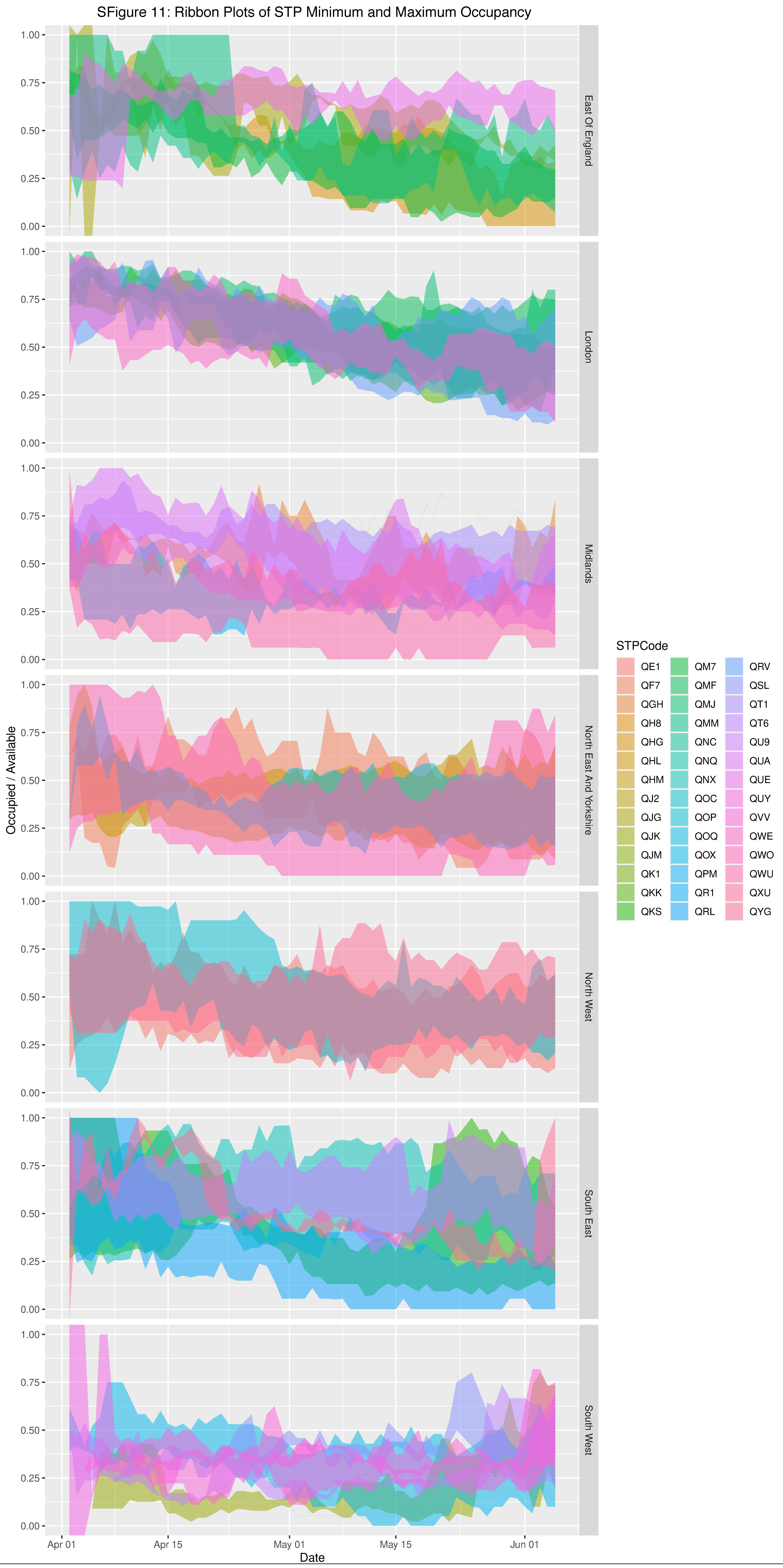
SFigure 11: Ribbon Plots of STP Minimum and Maximum Occupancy
Occupied / Available
Date
Apr 01
Apr 15
May 01
May 15
Jun 01
1.00
0.75
0.50
0.25
0.00
East Of England
London
Midlands
North East And Yorkshire
North West
South East
South West
STPCode
QE1
QF7
QGH
QH8
QHG
QHL
QHM
QJ2
QJG
QJK
QJM
QK1
QKK
QKS
QM7
QMF
QMJ
QMM
QNC
QNQ
QNX
QOC
QOP
QOQ
QOX
QPM
QR1
QRL
QRV
QSL
QT1
QT6
QU9
QUA
QUE
QUY
QVV
QWE
QWO
QWU
QXU
QYG

Supplementary Video 1

[See attached link for time-lapse of G&A bed capacity at the STP level across England]

Supplementary Video 2

[See attached link for time-lapse of mechanical ventilator bed capacity at the STP level across England]

Supplementary References

35 King's F. Sustainability and transformation plans (STPs) explained. 2017. https://www.kingsfund.org.uk/topics/integrated-care/sustainability-transformation-plans-explained.

36 Public Health England. COVID-19: investigation and initial clinical management of possible cases. 2020; published online Jan 10. https://www.gov.uk/government/publications/wuhan-novel-coronavirus-initial-investigation-of-possible-cases/investigation-and-initial-clinical-management-of-possible-cases-of-wuhan-novel-coronavirus-wn-cov-infection (accessed June 23, 2020).

37 Statistics. Statistics » Bed Availability and Occupancy Data – Overnight. https://www.england.nhs.uk/statistics/statistical-work-areas/bed-availability-and-occupancy/bed-data-overnight/. (accessed June 23, 2020).

38 Statistics. Statistics » Critical Care Bed Capacity and Urgent Operations Cancelled. https://www.england.nhs.uk/statistics/statistical-work-areas/critical-care-capacity (accessed June 23, 2020).

39 England NHS. NHS England » NHS steps up coronavirus fight with two more Nightingale Hospitals. https://www.england.nhs.uk/2020/04/nhs-steps-up-coronavirus-fight-with-two-more-nightingale-hospitals/ (accessed June 23, 2020).

40 England NHS. NHS England » NHS to build more Nightingale Hospitals, as London set for opening. https://www.england.nhs.uk/2020/04/nhs-to-build-more-nightingale-hospitals-as-london-set-for-opening/ (accessed June 23, 2020).
