# Supplementary material for: Hospital bed capacity and usage across secondary healthcare providers in England during the first wave of the COVID-19 pandemic: a descriptive analysis
Source: BMJ Open. 2021 Jan 26;11(1):e042945. doi: 10.1136/bmjopen-2020-042945 (PMC7843315; doi:10.1136/bmjopen-2020-042945)
Supplement: Author's manuscript [file bmjopen-2020-042945.draft_revisions.pdf]

BMJ Open is committed to open peer review. As part of this commitment we make the peer review history of every article we publish publicly available.

When an article is published we post the peer reviewers' comments and the authors' responses online. We also post the versions of the paper that were used during peer review. These are the versions that the peer review comments apply to.

The versions of the paper that follow are the versions that were submitted during the peer review process. They are not the versions of record or the final published versions. They should not be cited or distributed as the published version of this manuscript.

BMJ Open is an open access journal and the full, final, typeset and author-corrected version of record of the manuscript is available on our site with no access controls, subscription charges or pay-per-view fees (http://bmjopen.bmj.com).

If you have any questions on BMJ Open's open peer review process please email info.bmjopen@bmj.com

BMJ Open

Hospital bed capacity and usage across secondary healthcare providers during the first wave of the COVID-19 Pandemic

Journal:	*BMJ Open*
Manuscript ID	bmjopen-2020-042945
Article Type:	Original research
Date Submitted by the Author:	21-Jul-2020
Complete List of Authors:	Mateen, Bilal Akhter; The Alan Turing Institute,<br>Wilde, Harrison; University of Warwick<br>Dennis, John; University of Exeter, The Institute of Biomedical & Clinical Science<br>Duncan, Andrew; Imperial College London<br>Thomas, Nick; University of Exeter, The Institute of Biomedical & Clinical Science<br>McGovern, Andrew; University of Exeter, The Institute of Biomedical & Clinical Science<br>Denaxas, S; UCL<br>Keeling, Matt; University of Warwick,<br>Vollmer, Sebastian; University of Warwick
Keywords:	COVID-19, INTENSIVE & CRITICAL CARE, Public health < INFECTIOUS DISEASES, Health policy < HEALTH SERVICES ADMINISTRATION & MANAGEMENT

Note: The following files were submitted by the author for peer review, but cannot be converted to PDF. You must view these files (e.g. movies) online.

G&A Time Lapse.mp4
Vent Time Lapse.mp4

SCHOLARONE™ Manuscripts

I, the Submitting Author has the right to grant and does grant on behalf of all authors of the Work (as defined in the below author licence), an exclusive licence and/or a non-exclusive licence for contributions from authors who are: i) UK Crown employees; ii) where BMJ has agreed a CC-BY licence shall apply, and/or iii) in accordance with the terms applicable for US Federal Government officers or employees acting as part of their official duties; on a worldwide, perpetual, irrevocable, royalty-free basis to BMJ Publishing Group Ltd ("BMJ") its licensees and where the relevant Journal is co-owned by BMJ to the co-owners of the Journal, to publish the Work in this journal and any other BMJ products and to exploit all rights, as set out in our licence.

The Submitting Author accepts and understands that any supply made under these terms is made by BMJ to the Submitting Author unless you are acting as an employee on behalf of your employer or a postgraduate student of an affiliated institution which is paying any applicable article publishing charge ("APC") for Open Access articles. Where the Submitting Author wishes to make the Work available on an Open Access basis (and intends to pay the relevant APC), the terms of reuse of such Open Access shall be governed by a Creative Commons licence – details of these licences and which Creative Commons licence will apply to this Work are set out in our licence referred to above.

Other than as permitted in any relevant BMJ Author's Self Archiving Policies, I confirm this Work has not been accepted for publication elsewhere, is not being considered for publication elsewhere and does not duplicate material already published. I confirm all authors consent to publication of this Work and authorise the granting of this licence.

For peer review only

Hospital bed capacity and usage across secondary healthcare providers during the first wave of the COVID-19 Pandemic

Bilal A. Mateen (MBBS)[1,2,3]*, Harrison Wilde (BSc)[4], John M. Dennis (PhD)[5], Andrew Duncan (PhD)[1,6], Nicholas J. Thomas (MRCP)[5,7], Andrew P. McGovern (MD Res)[5,7], Spiros Denaxas (PhD)[1,8,9], Matt J Keeling (PhD)[10], Sebastian J. Vollmer (PhD)[1,4]

1 The Alan Turing Institute. Address: British Library, 96 Euston Road, London, NW1 2DB, UK
2 University of Warwick Medical School, Social Science and Systems in Health Unit. Address: Medical School Building, University of Warwick, Coventry, CV4 7AL, UK
3 Kings College Hospital NHS Foundation Trust. Address: Hambleden Wing, Kings College Hospital, Denmark Hill, London, SE5 9RS, UK
4 University of Warwick, Department of Statistics. Address: Department of Statistics, University of Warwick, Coventry, CV4 7AL, UK
5 University of Exeter Medical School. Address: Institute of Biomedical & Clinical Science, RILD Building, Royal Devon & Exeter Hospital, Barrack Road, Exeter EX2 5DW, UK
6 Imperial College London, Faculty of Natural Sciences. Address: Huxley Building, London, SW7 2AZ, UK
7 Royal Devon and Exeter NHS Foundation Trust, Diabetes and Endocrinology. Address: Exeter, Barrack Road, EX2 5DW
8 University College London, Institute of Health Informatics. Address: University College London, 222 Euston Rd, London NW1 2DA, UK
9 Health Data Research UK. Address: Gibbs Building, 215 Euston Road, London, NW1 2BE, UK
10 The Zeeman Institute for Systems Biology & Infectious Disease Epidemiology Research. Address: School of Life Sciences and Mathematics Institute, University of Warwick, Coventry, CV4 7AL, United Kingdom.

Author for Correspondence (*)
Dr. Bilal A. Mateen
Social Science and Systems in Health Unit
Warwick Medical School, University of Warwick
Coventry, CV4 7AL, UK
Email: bilal.mateen@nhs.net
Tel: +44 (0)24 7657 4880

Word Count: 3,641

For peer review only

Abstract

Objectives: In this study, we describe the pattern of bed occupancy across England during the first wave of the pandemic, January 31st to June 5th 2020.

Design: Descriptive survey

Setting: All non-specialist secondary care providers in England

Participants: Acute (non-specialist) Trusts with a type 1 (i.e. 24 hours/day, consultant-led) accident and emergency department (n = 125), Nightingale (Field) Hospitals (n = 7), and independent sector secondary care providers (n = 195).

Main Outcome Measures: Two thresholds for 'safe occupancy' were utilized, 85% as per Royal College of Emergency Medicine and 92% as per NHS Improvement.

Results: At peak availability, there were 2711 additional beds compatible with mechanical ventilation across England, reflecting a 53% increase in capacity, and occupancy never exceeded 62%. A consequence of the repurposing of beds meant that at the trough, there were 8.7% (8,508) fewer general and acute (G&A) beds across England, but occupancy never exceeded 72%. The closest to (surge) capacity that any trust in England reached was 99.8% for general and acute beds. For beds compatible with mechanical ventilation there were 326 trust-days (3.7%) spent above 85% of surge capacity, and 154 trust-days (1.8%) spent above 92%. 23 trusts spent a cumulative 81 days at 100% saturation of their surge ventilator bed capacity (median number of days per trust = 1 [range: 1 to 17]). However, only 3 STPs (aggregates of geographically co-located trusts) reached 100% saturation of their mechanical ventilation beds.

Conclusions: Throughout the first wave of the pandemic, an adequate supply of all bed-types existed at a national level. Due to an unequal distribution of bed utilization, many trusts spent a significant period operating above 'safe-occupancy' thresholds, despite substantial capacity in geographically co-located trusts; a key operational issue to address in preparing for a potential second wave.

For peer review only

Article Summary

What is already known on this topic

- A UK-specific modelling study of the impact of non-pharmacological interventions suggested that their introduction might prevent the healthcare system being overwhelmed.
- Several news reports were found pertaining to hospitals that reached ventilator capacity in England during the first wave of the pandemic, however, no single authoritative source was identified detailing impact across all hospital in England.

What this study adds

- This national study found evidence of an unequal distribution of resource utilization across England.
- Close examination of the geospatial data revealed that in the vast majority of circumstances there was relief capacity in geographically co-located hospitals.

Strengths and Limitations of this Study

- The use of an administrative data that is collected by the statutory regulator as part of its legal mandate resulted in minimal missing information.
- Results are presented in the context of several geographic units of healthcare provision (i.e. hospital/site, trust, and STP-level), thus providing a much richer understanding of resource utilization that is less prone to the diluent effects of higher level geographies.
- The data represents a daily snapshot and therefore is unable to capture the nuances of the hospital throughput; in essence, both under and over-reporting of occupancy is possible using this method.
- The use of the occupancy thresholds reflects a limitation of our analysis, in that a proxy for adverse outcomes had to be utilized given that the necessary information was not readily available to directly explore the relationship between occupancy and patient-level outcome.
- The results of this study may not be generalizable to other countries given that it is specific to the UK National Health System infrastructure.

For peer review only

Introduction

The ability of hospitals to cope with large influxes of patients, either due to a pandemic illness or seasonal increases in respiratory disease exacerbations is in part dictated by the availability of beds.[1] Since 1987, when formal reporting of the number of hospital beds began in the UK, there has been a sustained decline in the number of available beds across the NHS.[2] In recent years, this issue has garnered more attention due to the annual 'winter bed crisis',[3,4] where the end of the calendar year heralds a surge in emergency admissions often resulting in hospitals operating well above quality and operational performance tipping points, i.e. 85% or 92% total bed occupancy.[5-7] The saturation of hospital beds is not only problematic through it impact on the ability of the workforce to deliver high-quality care,[8] but additionally the bottle-necking of the emergency care workflow has been shown to contribute to suboptimal outcomes for patients,[9] including increased numbers of healthcare-acquired infections,[10] and increased mortality.[11-13]

These concerns about the NHS' ability to cope with large influxes of patients took on a new level of significance in early 2020, when the World Health Organization (WHO) formally declared COVID-19 a pandemic illness, due to its virulence, and the magnitude of the disease's impact globally.[14] As early reports from China were published, it became apparent that a relatively large proportion of individuals who contracted COVID-19 required admission to hospital,[15] for example due to: new oxygen requirements, sepsis, acute respiratory distress syndrome (ARDS), and even multi-organ dysfunction (MODS). Forecasts of the potential number of people requiring hospital admission and mechanical ventilation across the UK suggested that the baseline capacity of the NHS would be insufficient.[16] In an effort to ensure sufficient capacity the British government instituted a series of policies, including facilitating the discharge of individuals who had been delayed due to non-medical reasons in an effort to unlock capacity,[17] cancelling all non-urgent clinical work, opening large field hospitals (i.e. the Nightingale hospitals),[18] and increasing mechanical ventilator availability for clinical areas repurposed to manage high care patients.[19]

The UK has started making significant strides towards rolling back its non-pharmacological interventions including. reopening schools, and planning for the discontinuation of shielding for vulnerable people,[20] signaling an end to the first wave of the pandemic.[21] Following these changes, there is the potential for a second wave of infections in the coming months. Understanding regional differences in hospital capacity is fundamental

For peer review only

to informing the UK's response to a second wave, as well as for elucidating how to safely wind down repurposed surge capacity such as operating theatres to allow other much needed clinical activity to restart.[22] However, other than a few isolated news reports of hospitals exceeding their ventilator capacity,[23] it is unclear how well the NHS as a whole managed to respond to the additional demand for beds over recent months. In this study, we sought to describe the pattern of bed occupancy in hospitals across England during the first wave of the COVID-19 pandemic.

For peer review only

Methods

Primary Data Source

Data were accessed from the daily situation reports ('SitReps', covering the previous 24 hours) provided to the Scientific Pandemic Influence Group on Modelling (SPI-M) by NHS England on behalf of all secondary care providers. All NHS acute care providers, independent sector care providers, and field hospitals in England submitting information to the daily situation reports were eligible for inclusion.

Study population

The data is presented in the context of several different units of secondary care provision: hospitals/sites, trusts, sustainability and transformation partnerships (STPs), regions, and the whole of England (i.e. national), where each is an aggregate of the preceding unit (the structure of UK care providers is explained in the supplementary material).

Inclusion and Exclusion Criteria

Exclusions were applied at the trust level for NHS-specific care providers. Exclusion criteria were as follows: acute specialist trusts: women and/or children (n = 4), neurology & ophthalmology (n = 2), heart & lung (n = 3), orthopedic, burns & plastics (n = 4), cancer (n = 3). The remaining care providers were grouped into three categories and analyzed separately: 1) Acute (non-specialist) Trusts with a type 1 (i.e. 24 hours/day, consultant-led) accident and emergency department (n = 125); 2) Nightingale (Field) Hospitals (n = 7), and; 3) independent sector providers (n = 195).

Recruitment Period

Data was available from 27th March 2020 (the first available SitRep) to 5th June 2020 inclusive.

Recorded Information

The data specification comprised resource utilization and capacity-specific information, including the number of beds at each trust, stratified by several factors of interest, including acuity and COVID-19 ascertainment (further defined in supplementary material). Notably, beds were only recorded as being available if they were 'funded' (i.e. there was adequate staffing and resources for the bed to be occupied), so as to prevent counting of beds that could not accommodate a new patient. Bed acuity was organized into: general and acute (G&A), beds compatible

For peer review only

with non-mechanical ventilation, and beds compatible with mechanical ventilation. Occupancy is calculated based on the status of each bed at 08:00 each day, and then later separated by the proportion that had a positive COVID-19 test.

Reporting fields changed on the 27th of April 2020, with several additional columns being added, which included specific fields for level 2 (HDU) and level 3 (ITU) beds. The impact of these changes is detailed in the supplementary material. However, one crucial outcome was that it became apparent the definition of critical care beds utilized prior to 27th April 2020 was not consistent with prior reporting practices of only including level 2 (HDU) and level 3 (ITU) beds,[24] as the newly reported values did not equal the simultaneously reported critical care values. As such, any results pertaining to critical care, HDU and ITU are reported separately in the supplementary material.

NHS England reports trust-level data, whereas we additionally attempted to disaggregate this information into the individual hospitals that the trusts comprise. Not all of the trusts were amenable to disaggregation from the trust-level data into independently reported sites in the available extracts, resulting in a final sample of 173 unique hospital sites, comprising 91.7% of the total number of ventilated beds and 81.4% of the general and acute beds when compared to trust level. The change in data reporting introduced on the 27th of April 2020 also resulted in variation in information capture; for data prior to the 27th of April, the results available reflect 89.6% of all mechanical ventilator beds and 86.9% of general and acute beds, where data from the 27th onwards the results reflect 93.0% of all mechanical ventilator beds but 77.0% of general and acute beds.

Outcome

The primary outcomes of interest were bed availability, and bed occupancy by patients with and without COVID-19, for each level of secondary care provision, i.e. hospital, trust, sustainability and transformation partnership (STP). Different 'safe occupancy' thresholds were used to interpret the results; 85% as per the Royal College of Emergency Medicine, and 92% as per NHS Improvement. We also compared occupancy against baseline bed occupancy (see supplementary material for definitions), and 100% of surge capacity.

For peer review only

Statistical Analysis

We generated and reported descriptive summaries (e.g. medians, ranges, counts, proportions) of the data. We reported absolute numbers for hospital, trusts and STPs attaining specific occupancy thresholds. In light of the discordant critical care and HDU/ITU values, this analysis was handled and reported separately (see supplementary material). To capture the temporal aspect of the information available, the number of hospital-days, trust-days, and STP-days above hospital baseline capacity and surge capacities of 85%, 92% and 100% is also reported. Full details on the quality control procedures are reported in the supplementary material (SFigures 1 & SFigure 2). Details on aggregation and disaggregation of geographic information are provided in STable 1 & STable 2. Analysis were carried out in R,[25] ggplot2 package.[26] Maps were acquired from the UK's Office for National Statistics Open Geography Portal.[27]

Patient and Public Involvement

No patients were involved in the design of the study, interpretation of the results, or drafting of this manuscript.

For peer review only

Results

National Mobilization

During the first wave of the pandemic, the NHS repurposed general/acute beds into those suitable for higher acuity patients (i.e. HDU/ITU), and patients requiring mechanical ventilation. Available ventilated bed capacity peaked at an additional 2711 beds, a 53% increase from a baseline of 4123 beds. Ventilated beds occupancy never exceeded 62% of this capacity (Figure 1), however there were notable regional differences in COVID-19 specific demand (Figure 2, Figure 3 & SFigure 3). Similar patterns were observed in critical care/HDU and ITU beds (SFigure 4). A consequence of the repurposing of beds for higher acuity patients there was a 8.7% reduction (n= 8,508) of general and acute beds from a baseline of 97,293 beds. There was a large reduction of the number of beds occupied by patients without COVID-19; 53,136 fewer beds were occupied (58.8% reduction) at the nidus compared to average occupancy from January to March 2020. Total bed occupancy never exceeded 72% nationally (Figure 1).

Occupancy relative to Baseline Capacity

Out of the 125 trusts (aggregates of hospitals), 3 trusts (2.4%) at some point during the first wave were operating above their baseline bed availability for general and acute beds (124 trust-days [1.4% of the total 8738 days at risk]; median number of days per trust = 36 days [range: 30 to 58]; Figure 4). For beds compatible with mechanical ventilation, 87 trusts (69.6%) at some point during the first wave were operating above their baseline bed availability (2456 trust-days [28.1% of total at risk]; median number of days per trust = 24 days [range: 1 to 61]; Figure 5). Similar results to that of mechanical ventilation compatible beds were seen for critical care / HDU and ITU bed occupancy (see supplementary material, SFigure 5 & SFigure 6).

Occupancy relative to Surge Capacity

Table 1 summarizes the number of hospitals, trusts and STPs operating above each of the thresholds for ‘safe occupancy’, and details the duration (i.e. median number of days) that each spent above the designated thresholds.

Hospital-level Occupancy

Of the total 11,851 English hospital-days at risk over the study period, 494 hospital-days (4.17% of total days at risk) were at or above 85% of bed (surge) capacity, 110 hospital-days (0.92%) were at or above 92% of bed

For peer review only

(surge) capacity, and only 10 were spent at 100% of surge capacity (Figure 6). Similarly, for beds compatible with mechanical ventilation there were 586 hospital-days (4.94%) spent above 85% of surge capacity, 320 hospital-days (2.70%) were spent above 92%, and 226 hospital-days (1.9%) were spent at 100% occupancy (see Figure 7). Summaries of the size and geographic locations of hospitals stratified by saturation are in STable 3.

Trust-level Bed Occupancy

Over the study period, there were 287 trust-days (3.3% of total days at risk) where general and acute bed occupancy exceeded 85% occupancy of surge capacity, and 57 trust-days (0.7%) were at or above 92% of bed (surge) capacity. The closest to capacity any trust in England reached was 99.8% for general and acute beds. However, for beds compatible with mechanical ventilation there were 326 trust-days (3.7%) spent above 85% of surge capacity, and 154 trust-days (1.8%) spent above 92%. 23 trusts reached 100% saturation of their mechanical ventilator bed capacity (Figure 8 & SFigure 7).

Table 1: The Number of Hospital/Trusts/STPs at each Occupancy Threshold for Different Bed Types

Bed Type	Organizational Unit	Occupancy Threshold					
		> 85%		> 92%		= 100%	
		Number (%)	Median number of days at or above threshold (range)	Number (%)	Median number of days at or above threshold (range)	Number (%)	Median number of days at or above threshold (range)
General & Acute	Hospital/Site (n = 173)	56 (32.4%)	6 (1 to 45]	19 (11.0%)	3 (1 to 19)	1 (0.6%)	10 days
	Trust (n = 125)	30 (24.0%)	5 (1 to 46)	14 (11.2%)	3 (1 to 13)	0 (0.0%)	-
	Sustainability and Transformation Partnership (n = 42)	2 (4.8%)	10 (3 to 17)	2 (4.8%)	1 (1 to 1)	0 (0.0%)	-
Mechanical Ventilation	Hospital/Site (n = 173)	91 (52.6%)	4 (1 to 48)	72 (41.6%)	3 (1 to 48)	52 (30.0%)	2 (1 to 48)
	Trust (n = 125)	58 (46.4%)	3 (1 to 27)	40 (32.0%)	2 (1 to 17)	23 (18.4%)	1 (1 to 17)
	Sustainability and Transformation Partnership (n = 42)	10 (23.8%)	2 (1 to 11)	5 (10.4%)	1 (1 to 6)	3 (7.1%)	1 (1 to 2)

For peer review only

Sustainability and Transformation Partnerships (STP) level Bed Occupancy

Across the 42 STPs (aggregates of trusts), there were 20 STP-days (0.7% of total days at risk) where general and acute bed occupancy exceeded 85% occupancy of surge capacity. The highest any STP reached for G&A bed occupancy was 92.7%. For beds compatible with mechanical ventilation, there were 35 STP-days (1.2%) where occupancy exceeded 85% occupancy of surge capacity, 11 STP-days (0.4%) in excess of 92% occupancy, and 4 STP-days (0.1%) at full occupancy (all of which were for STPs outside London: 1) Somerset, 2) Suffolk and North East Essex, and 3) Shropshire, Telford and Wrekin; SFigure 8). Figure 9 illustrates the number of STPs operating at each distinct occupancy threshold as a proportion of baseline and actual surge capacity. The full time-lapse for G&A (Video 1) & ventilator bed (Video 2) occupancy over the period of interest can be found in the supplementary material. A similar pattern was seen in the context of critical care / HDU & ITU beds across the STPs (SFigure 5 & SFigure 6).

Field (Nightingale) Hospital Occupancy

Of the reported bed capacity achievable through opening the Nightingale hospitals, at peak occupancy only 1.23% of the theoretical maximum were being utilized (Table 2). This equates to 618 bed days for patients with COVID-19 requiring mechanical ventilation, and 1483 bed days for all other types of intervention for patients with COVID-19 (i.e. oxygenation, non-invasive respiratory support, non-respiratory organ support, etc.).

Table 2: Field (Nightingale) Hospital Occupancy, and Capacity

Nightingale Hospital Location	Number of Occupied Beds at peak*	Maximum number of Operational Beds*	Maximum Theoretical Capacity (Beds)
London (Excel Centre)	66	112	4000
Manchester (Convention Centre)	47	72	1000
Birmingham (National Exhibition Centre)	0	0	2000
Bristol (University of West England)	NA	NA	1000
Washington (Centre of Excellence for Sustainable Advanced Manufacturing)	NA	NA	450
Harrogate (Convention Centre)	0	0	500
Exeter (Westpoint Arena)	NA	NA	200

**Several hospitals were formally opened, but never reported an occupied bed, as such they did not appear in the SitRep dataset (denoted by NA in the table). Those that were in the dataset but had no patients are denoted by a '0'.*

For peer review only

Independent Sector Care Providers

Variations in reporting meant that the number of providers reporting each day varied, median 181 providers (range: 172 to 187). At peak occupancy, no more than 134 independent sector beds were occupied with patients who were confirmed COVID-19 positive. With regards to patients without COVID-19, at peak occupancy there were 1350 people in independent sector beds, representing a peak saturation of 18.7% (based on the total number of beds reported during contractual negotiations). In summary, there were 3360 bed days for patients with confirmed COVID-19 accommodated by the independent sector (86 mechanical ventilator bed days, 104 non-invasive ventilation bed days, and 3170 other bed days), and 53937 bed days for patients without COVID-19 (2771 mechanical ventilator bed days, 2046 non-invasive ventilation bed days, and 49120 other bed days), between 2nd April and 5th June across England.

For peer review only

Discussion

This national study of hospital-level bed occupancy provides unique and timely insight into the impact of COVID-19 on bed-specific resource utilization across an entire country. Our analysis suggests that the response of the NHS and British government to COVID-19 was sufficient to alleviate early concerns regarding the number of mechanical ventilators and critical care beds at a national level, however local variation in demand still meant that many trusts reached 100% capacity for both. Moreover, examining occupancy in the context of different organizational units (i.e. trust-level versus STP-level), suggests that the higher-order networks (i.e. STPs) were not efficiently utilized to off-load disproportionately impacted trusts, as it was theoretically possible to have 95.1% fewer trust days at 100% mechanical ventilator bed capacity assuming load was better distributed. On the other hand, despite a reduction in overall capacity, G&A bed occupancy-levels relatively infrequently reached 'unsafe' levels, even at the individual hospital-level. This in part may explain why the field hospitals and independent sector care provider beds were never substantially utilized. Only a very small fraction of the theoretical maximum field hospital bed capacity was operationalized (1.23%). Similarly, despite signing a 14 week block contract with all of the major independent sector care providers valued at £235 million,[28] these beds too remained largely unoccupied, with less than 24% of the theoretical maximum beds days for established ventilators (i.e. not including the 1012 theatre-specific mechanical ventilators) having been used.

Context

Initial estimates suggested that an additional 30,000 mechanical ventilators would be necessary to accommodate the impact of the COVID-19 pandemic. These estimates were later updated to just 18,000 mechanical ventilators, from an estimated baseline of 8,000 across the UK.[29] It is difficult to determine the accuracy of these projections, as they were made in the absence of the impact of non-pharmacological interventions. However, the results of our study suggests that, at the population level, UK-based models of ventilator and bed resource utilization which integrated the impact of non-pharmacological interventions were actually remarkably accurate.[16,30] Arguably the most influential modelling study was that of the Imperial MRC group, wherein the authors clearly illustrate that with full 'lockdown' (i.e. the suite of non-pharmacological interventions that were eventually instituted), that critical care bed capacity would not be overwhelmed.[16] The nuance that this modelling study lacked was that it failed to explicitly incorporate the impact of unequal distribution of burden,

For peer review only

which manifested in our data as specific hospitals and trusts reaching full occupancy, despite the fact that at the national-level there was a substantial number of unoccupied beds.

This retrospective analysis also highlights some of the early incorrect assumptions made about the UK's baseline resource availability. For example, in contrast to ministerial statements suggesting that there were approximately 8,000 ventilators in the UK prior the pandemic,[29] our results identified only 4123 operational beds compatible with mechanical ventilation on the first day of reporting in England. Even after acknowledging that our value does not account for the devolved nations (Wales, Scotland, and Northern Ireland), it is unlikely that the initial figures reported by members of parliament truly reflected operational capacity, as that would suggest only 50% of such equipment was in England, despite it representing 84% of the UK population. Interestingly, the absolute increase in ventilator numbers due to the government incentives (e.g. the UK's Industrial Ventilator Challenge) is much more similar to our reported results.

Strengths and Limitations

There are several strengths to this study. For example, the use of an administrative (i.e. 'SitRep') data that is a statutory collection by NHS England, via a well-established reporting mechanism that has been exploited for research,[31] confers robustness to the data. One example of how this robustness manifested is, unlike other attempts to collect data at a national level to inform the COVID-19 response plan in the UK,[32] the degree of missingness in the data utilized in this study was minimal (see supplementary material). Moreover, in light of the unique access to the raw 'SitRep' data, we have been able to present our results not only at the trust-level, to which previous endeavors have been limited,[33] but rather have been able to present information at a much more granular layer (i.e. hospital/site-level) thus providing a much richer understanding of resource utilization that is less prone to the diluent effects of higher level geographies. Finally, a further strength of this study is the relative simplicity of the analysis; there are no complex statistical methods utilized as the descriptive summaries presented are sufficient to describe the experiences of nationalized (single-payer) health system in a high-income economy during the first wave of the COVID-19 pandemic.

Notably though, there are also several limitations to the dataset and our analysis. Firstly, the changes introduced in 'SitRep' data collection half-way through the reporting period limited our ability to investigate critical care bed occupancy which was the third bed-specific potential concern identified by forecasting experts. The

For peer review only

hospital-level results should also be interpreted in the context of the fact that it is an incomplete representation of the core trust-level information, and thus may not truly reflect the exact position of each organization; for example, the trust corresponding to the single site that achieved 100% G&A occupancy was never itself at 100% occupancy. On a related note, the core weakness of the 'SitRep' data is that it presents data as a daily snapshot (at 08:00/8am), and therefore is unable to capture the nuances of the hospital throughput; in essence, both under and over-reporting of occupancy is possible using this method. As such, any marginal results where hospitals are only just over one of the 'safe occupancy-level' thresholds should be interpreted with caution as they could represent reporting artefacts. Moreover, the use of the occupancy thresholds reflects a limitation of our analysis, in that a proxy for adverse outcomes had to be utilized given that the necessary information was not readily available to directly explore the relationship between occupancy and patient-level outcome. Finally, the results of this study may not be generalizable to other countries given that it is specific to the UK National Health System infrastructure.

Implications for Policy Makers, and Clinicians

This study illustrates the potential for near real-time results reporting by which to determine the need for and effectiveness of government policies introduced to address resource utilization-specific issues as a consequence of the COVID-19 pandemic. For example, due to an unequal distribution of the resource utilization burden across England, many trusts spent a significant period of time operating above 'safe-occupancy' thresholds, despite the fact that in the vast majority of circumstances there was relief capacity in geographically co-located trusts (i.e. at the STP-level). Out of the 81 trust-days spent at 100% saturation of their mechanical ventilation beds (which pertains to 23 trusts reaching this threshold), on all but 5 days there was spare capacity at the corresponding STP level, which would have resulted in only 4 trusts reaching 100% saturation at any point (SFigure 9: STP min-max occupancy plots). This reflects a key operational issue for policymakers to address in preparing for a potential second wave, and would have been identifiable if the SitRep data had been utilized for now-casting. Moreover, other policies for which these results may be relevant, include the creation of the Nightingale (field) hospitals, and independent sector network partnership. Our results suggest that the early investment and the creation of an operational field hospital and independent sector network may yield more overtly positive results in the winter, when G&A occupancy-levels regularly exceed 92%,[34] however, during the first wave of the pandemic they were under-utilized.

For peer review only

Conclusion

Using administrative data submitted by all secondary care organizations in England, we can conclude that at the national level there was an adequate supply of all bed-types throughout the first wave of the COVID-19 pandemic. However, the burden of need was not equally distributed, and thus in many cases local demand exceeded the supply of beds, especially where it concerned mechanical ventilation. Although several of the policies introduced by the government, both historical (i.e. STPs) and pandemic-specific (e.g. the independent sector block contract), could have potentially addressed this issue, there is evidence that these interventions were not optimally utilized.

For peer review only

Acknowledgements

BAM, SJV, and SD are supported by The Alan Turing Institute (EPSRC grant EP/N510129/). JMD is supported by an Independent Fellowship funded by Research England's Expanding Excellence in England (E3) fund. SJV is supported by the University of Warwick IAA funding. HW is supported by the Feuer International Scholarship in Artificial Intelligence. JMD, NJT, and APM are supported by the NIHR Exeter Clinical Research Facility. We thank NHS Improvement & NHS England for providing access to the SitRep data and Prof. Matt Keeling for establishing data access. Finally, we thank Dr. Bu'Hussain Hayee for sense-checking the final draft.

Contributors

BAM and SJV conceived the study. Analysis was carried out by HW under the supervision of SJV and BAM. BAM drafted the manuscript, with input from HW and SJV. All authors made substantial contributions to the critical revision of the article, and approved the final version for submission. BAM and SJV take responsibility for the integrity of the data and the accuracy of the data analysis.

Declaration of interest

APM declares previous research funding from Eli Lilly and Company, Pfizer, and AstraZeneca. SJV declares funding from IQVIA. All other authors declare no competing interests.

Ethics & Governance

Data utilized in this study were made available through an agreement between the University of Warwick and the Scientific Pandemic Influence Group on Modelling (SPI-M), whom were acting on behalf of the British Government. The study was reviewed and approved by the Warwick BSREC (BSREC 120/19-20).

Data Access Statement

Trust-level data will eventually be published by NHS England as a freely accessible data resource, but outputs have been delayed by the COVID-19 pandemic. For expedited or more granular access, requests will need to be made directly to NHS England (contact via england.dailysitrep@nhs.net). All code for this study is available on request.

Guarantor Statement

For peer review only

The corresponding and the senior author had full access to all data and had final responsibility for the decision to submit for publication.

Role of the Funding Source

There was no direct funding for this study. No funder was involved in the study design, analysis, interpretation of data; in the writing of the report; or in the decision to submit the paper for publication.

Dissemination

We will work with the relevant governmental groups to ensure that the patient organizations are informed of these results in a timely manner.

For peer review only

References

1 NHS hospital bed numbers. The King's Fund. 2020; published online March 26. Available at: https://www.kingsfund.org.uk/publications/nhs-hospital-bed-numbers (accessed June 21, 2020).

2 Appleby J. The hospital bed: on its way out? *BMJ* 2013; **346**: f1563.

3 The NHS this winter: looking beneath the national view | The Health Foundation. The Health Foundation. Available at: https://www.health.org.uk/news-and-comment/blogs/the-nhs-this-winter-looking-beneath-the-national-view (accessed June 21, 2020).

4 British Medical Association. Bed occupancy in the NHS - Pressures - BMA. The British Medical Association is the trade union and professional body for doctors in the UK. 2020; published online Jan 20. Available at: https://www.bma.org.uk/advice-and-support/nhs-delivery-and-workforce/pressures/bed-occupancy-in-the-nhs (accessed June 21, 2020).

5 Bagust A, Place M, Posnett JW. Dynamics of bed use in accommodating emergency admissions: stochastic simulation model. *BMJ* 1999; **319**: 155–8.

6 Fixing Emergency Department Staffing, Systems & Support to deliver excellent patient centered care. The Royal College of Emergency Medicine. Available at: https://www.rcem.ac.uk/docs/Policy/RCEM%20Vision%202020.pdf.

7 Improvement NHS. NHS review of winter 2017/18: annex. Available at: https://improvement.nhs.uk/documents/3202/Winter_review_annex_2.pdf (accessed June 22, 2020).

8 Against the odds: experiences from the NHS front line. RCP London. 2017; published online Feb 28. Available at: https://www.rcplondon.ac.uk/projects/outputs/against-odds-experiences-nhs-front-line (accessed June 23, 2020).

9 The implications of high bed occupancy rates on readmission rates in England | The Health Foundation. The Health Foundation. Available at: https://www.health.org.uk/publications/journal-articles/the-implications-of-high-bed-occupancy-rates-on-readmission- (accessed June 22, 2020).

10 Great Britain: National Audit Office. National Audit Office (NAO) - Department of Health:

For peer review only

Emergency Admissions to Hospital: Managing the Demand - HC 739: Managing the Demand, Department of Health. The Stationery Office, 2013.

11 Cowan RM, Trzeciak S. Clinical review: Emergency department overcrowding and the potential impact on the critically ill. *Crit Care* 2005; **9**: 291–5.

12 Sprivulis PC, Da Silva J-A, Jacobs IG, Frazer ARL, Jelinek GA. The association between hospital overcrowding and mortality among patients admitted via Western Australian emergency departments. *Med J Aust* 2006; **184**: 208–12.

13 Emergency and acute medical care in over 16s: service delivery and organisation. NICE. Available at: https://www.nice.org.uk/guidance/ng94/evidence/39.bed-occupancy-pdf-172397464704.

14 World Health Organization. Coronavirus Disease (COVID-19) Situation Reports. Available at:https://www.who.int/emergencies/diseases/novel-coronavirus-2019/situation-reports (accessed May 21, 2020).

15 Zhou F, Yu T, Du R, *et al.* Clinical course and risk factors for mortality of adult inpatients with COVID-19 in Wuhan, China: a retrospective cohort study. *Lancet* 2020; **395**: 1054–62.

16 Ferguson NM, Laydon D, Nedjati-Gilani G, *et al.* Impact of non-pharmaceutical interventions (NPIs) to reduce COVID-19 mortality and healthcare demand. 2020. *DOI* 2020;**10**:77482.

17 Stevens S. NHS-E and NHS-I LETTER ON NEXT STEPS ON NHS RESPONSE TO COVID-19. NHS England. Available at: https://www.england.nhs.uk/coronavirus/wp-content/uploads/sites/52/2020/03/urgent-next-steps-on-nhs-response-to-covid-19-letter-simon-stevens.pdf.

18 NHS England. New NHS nightingale hospital to fight coronavirus. Available at: https://www.england.nhs.uk/2020/03/new-nhs-nightingale-hospital-to-fight-coronavirus/ (accessed June 21, 2020).

19 UK Government Ventilator Challenge. 2020; published online April 22. Available at: https://www.gov.uk/government/topical-events/coronavirus-uk-government-ventilator-challenge/about (accessed June 21, 2020).

20 Department of Health, Care S. Plans to ease guidance for over 2 million shielding. 2020; published

For peer review only

online June 22. Available at: https://www.gov.uk/government/news/plans-to-ease-guidance-for-over-2-million-shielding (accessed June 23, 2020).

21 Prime Minister's Office, 10 Downing Street. Six people can meet outside under new measures to ease lockdown. 2020; published online May 28. Available at: https://www.gov.uk/government/news/pm-six-people-can-meet-outside-under-new-measures-to-ease-lockdown (accessed June 21, 2020).

22 Shock to the system: COVID-19's long-term impact on the NHS | The Health Foundation. The Health Foundation. Available at: https://www.health.org.uk/news-and-comment/blogs/shock-to-the-system-covid-19s-long-term-impact-on-the-nhs (accessed June 23, 2020).

23 Coronavirus: 'Critical incident' declared at Northwick Park Hospital in London after running out of intensive care beds [Internet]. ITV News. 2020 [cited 24 June 2020]. Available from: https://www.itv.com/news/2020-03-20/northwick-park-hospital-london-coronavirus-covid-19/

24 Monthly Trust SitRep (MSitRep) Critical Care Bed Capacity and Urgent Operations Cancelled. NHS England. Available at: https://www.england.nhs.uk/statistics/wp-content/uploads/sites/2/2019/11/MSitRep-Guidance-v1.09.pdf (accessed June 22, 2020).

25 R Core Team. R: A language and environment for statistical computing. R Foundation for Statistical Computing. Vienna, Austria. 2011.

26 Wickham H. ggplot2: Elegant Graphics for Data Analysis. Springer-Verlag New York. 2016.

27 Sustainability and Transformation Partnerships (April 2020) Boundaries. Available at: https://geoportal.statistics.gov.uk/datasets/sustainability-and-transformation-partnerships-april-2020-boundaries-en-buc?geometry=-43.431%2C48.021%2C39.142%2C57.304 (accessed June 23, 2020).

28 Permain N. COVID-19: Partnership working with the Independent Sector Providers and the Independent Healthcare Providers Network (IHPN). NHS England. Available at: https://www.england.nhs.uk/coronavirus/wp-content/uploads/sites/52/2020/03/ihpn-partnership-letter-25-march-2020.pdf (accessed June 22, 2020).

29 Balogun B. Coronavirus: Ventilator availability in the UK. 2020; published online June 3. Available at:

https://commonslibrary.parliament.uk/research-briefings/cbp-8904/ (accessed June 23, 2020).

30 Deasy J, Rocheteau E, Kohler K, *et al.* Forecasting ultra-early intensive care strain from COVID-19 in England. *medRxiv* 2020. https://www.medrxiv.org/content/medrxiv/early/2020/03/23/2020.03.19.20039057.full.pdf.

31 Friebel R, Fisher R, Deeny SR, Gardner T, Molloy A, Steventon A. The implications of high bed occupancy rates on readmission rates in England: A longitudinal study. *Health Policy* 2019; **123**: 765–72.

32 Dennis JM, Mateen BA, Sonabend R, Thomas NJ, Patel KA, Hattersley AT, Denaxas S, McGovern AP, Vollmer SJ. Diabetes and COVID-19 Related Mortality in the Critical Care Setting: A Real-Time National Cohort Study in England. Available at SSRN 3615999. 2020 May 27.

33 The NHS this winter: looking beneath the national view | The Health Foundation. The Health Foundation. Available at: https://www.health.org.uk/news-and-comment/blogs/the-nhs-this-winter-looking-beneath-the-national-view (accessed June 23, 2020).

34 The NHS this winter: looking beneath the national view | The Health Foundation. The Health Foundation. Available at: https://www.health.org.uk/news-and-comment/blogs/the-nhs-this-winter-looking-beneath-the-national-view (accessed June 23, 2020).

For peer review only

Figure Legends

Figure 1: National and Regional Bed Occupancy

Legend: Figure 1 illustrates total capacity and occupancy status, for both general and acute (G&A), as well as bed compatible with mechanical ventilation, at the national level.

Figure 2: Total bed occupancy in each of the 7 regions of England

Figure 2 illustrates total occupancy (COVID-19 positive and negative) in each of the 7 regions across England, for both general and acute (G&A), as well as bed compatible with mechanical ventilation. Note: the highly-saturated solid line represents a smoothened function of the raw data, whereas the less saturated solid line represents the underlying raw values. The former is based on the ggplot loess fit for trend lines, using local poly-regression curve fitting.

Figure 3: COVID-19 specific bed occupancy in each of the 7 regions of England

Figure 3 illustrates COVID-19 specific occupancy in each of the 7 regions across England, for both general and acute (G&A), as well as bed compatible with mechanical ventilation. Note: the highly-saturated solid line represents a smoothened function of the raw data, whereas the less saturated solid line represents the underlying raw values. The former is based on the ggplot loess fit for trend lines, using local poly-regression curve fitting.

Figure 4: Trust-Level General & Acute Bed Occupancy (Based on Baseline Capacities) Across England

Legend: Figure 4 illustrates the proportion of all trusts, and sustainability and transformation partnerships (STPs), at varying general and acute (G&A) bed occupancy thresholds relative to their baseline (mean availability January-March 2020) capacity, across England, from April 1st to June 5th. The superimposed colours represent how long the trusts spent at each specific threshold.

Figure 5: Trust-Level Ventilator Bed Occupancy (Based on Baseline Capacities) Across England

Figure 2B (Bottom) illustrates the proportion of all trusts, and sustainability and transformation partnerships (STPs), at varying ventilator bed occupancy thresholds relative to their baseline capacity, across England, from April 1st to June 5th. The superimposed colours represent how long the trusts spent at each specific threshold.

For peer review only

Figure 6: Hospital-level General & Acute Bed Occupancy (Based on Surge Capacities) Across England

Legend: Figure 6 illustrates the number of hospitals with general and acute bed occupancy in excess of the thresholds for 'safe and effective' functioning, i.e. 85% as defined by the Royal College of Emergency Medicine,[6] and 92% as defined by NHS Improvement and NHS England (green and yellow, respectively),[7] across England, from 26th March to June 5th. Note: all data was missing for the 29th of March and the 24th of May.

Figure 7: Hospital-level Ventilator Bed Occupancy (Based on Baseline Capacities) Across England

Figure 7 illustrates the number of hospitals with occupancy of mechanical ventilation beds in excess of the thresholds for 'safe and effective' functioning, i.e. 85% as defined by the Royal College of Emergency Medicine,[6] and 92% as defined by NHS Improvement and NHS England (green and yellow, respectively),[7] across England, from 1st April to June 5th. Note: all data was missing for the 24th of May.

Figure 8: Trust-Level Ventilator Bed Occupancy (Based on Surge Capacities) Across England

Legend: Figure 8 illustrates the number of trusts with occupancy of mechanical ventilation beds in excess of the thresholds for 'safe and effective' functioning, i.e. 85% as defined by the Royal College of Emergency Medicine,[6] and 92% as defined by NHS Improvement and NHS England (yellow and red, respectively),[7] across England, from March 26th to June 5th. Note: all data was missing for the 29th of March and the 24th of May. Several hospitals reported values consistent with 100% occupancy (black).

Figure 9: Peak Sustainability and Transformation Partnership (STP) Bed Occupancy Across England

Legend: Figure 9 illustrates the date on which general and acute bed occupancy (Left) and mechanical ventilator beds (Right) peaked, based on surge capacities at the Sustainability and Transformation Partnership (STP) level, across England. The geo-temporal pattern of peak occupancy clearly demonstrates that there was always residual G&A capacity at the STP level, and that all regions across England experienced similar levels of saturation. However, saturation of mechanical ventilator beds differed substantially by location.

For peer review only

Figure 1: National Bed Availability, Stratified by Occupancy Status

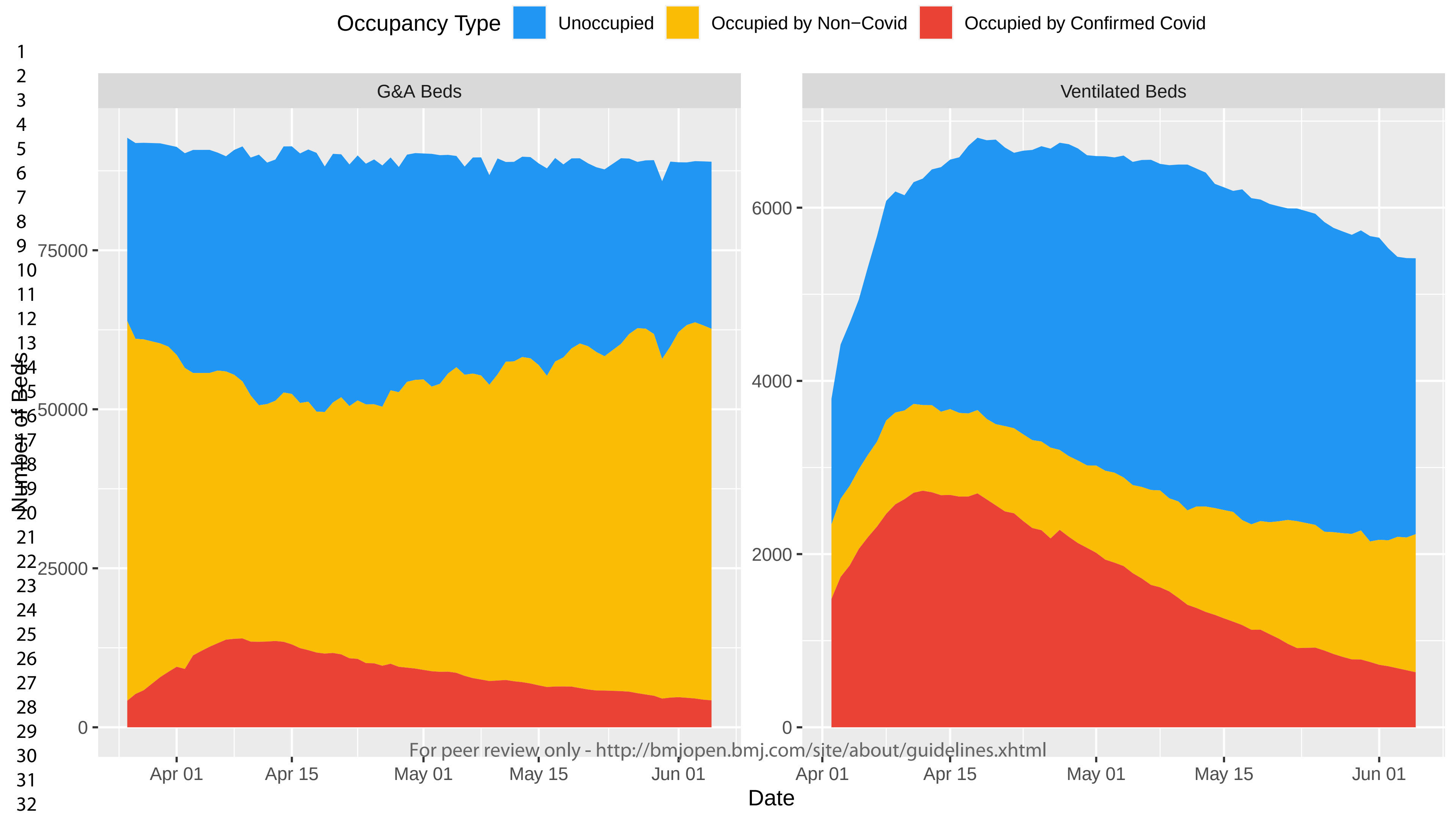

For peer review only - http://bmjopen.bmj.com/site/about/guidelines.xhtml

Figure 2: Total Regional Bed Occupancy (COVID and Non-Covid)

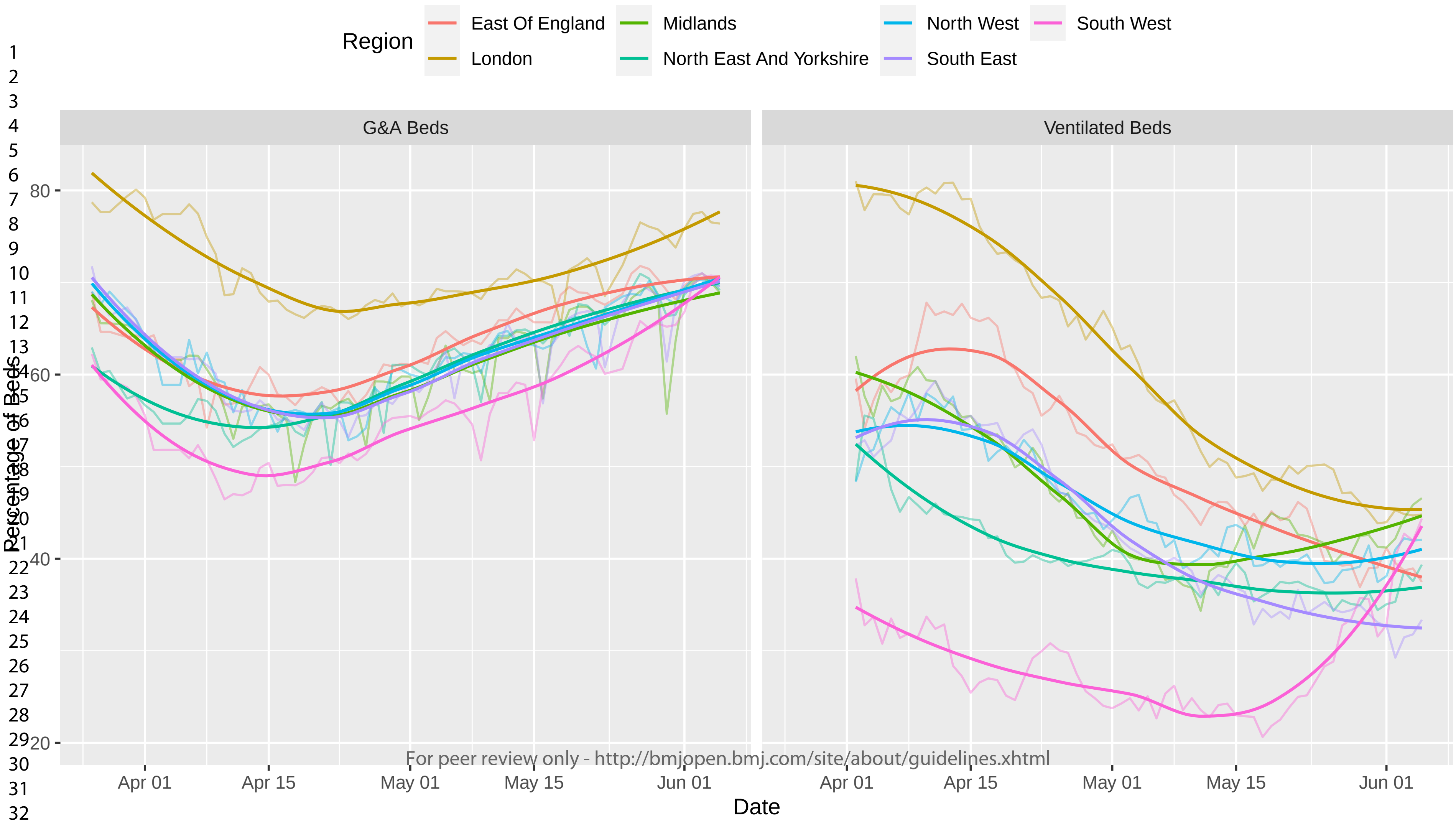

Figure 3: Total Confirmed COVID-19 Specific Bed Occupancy by Region

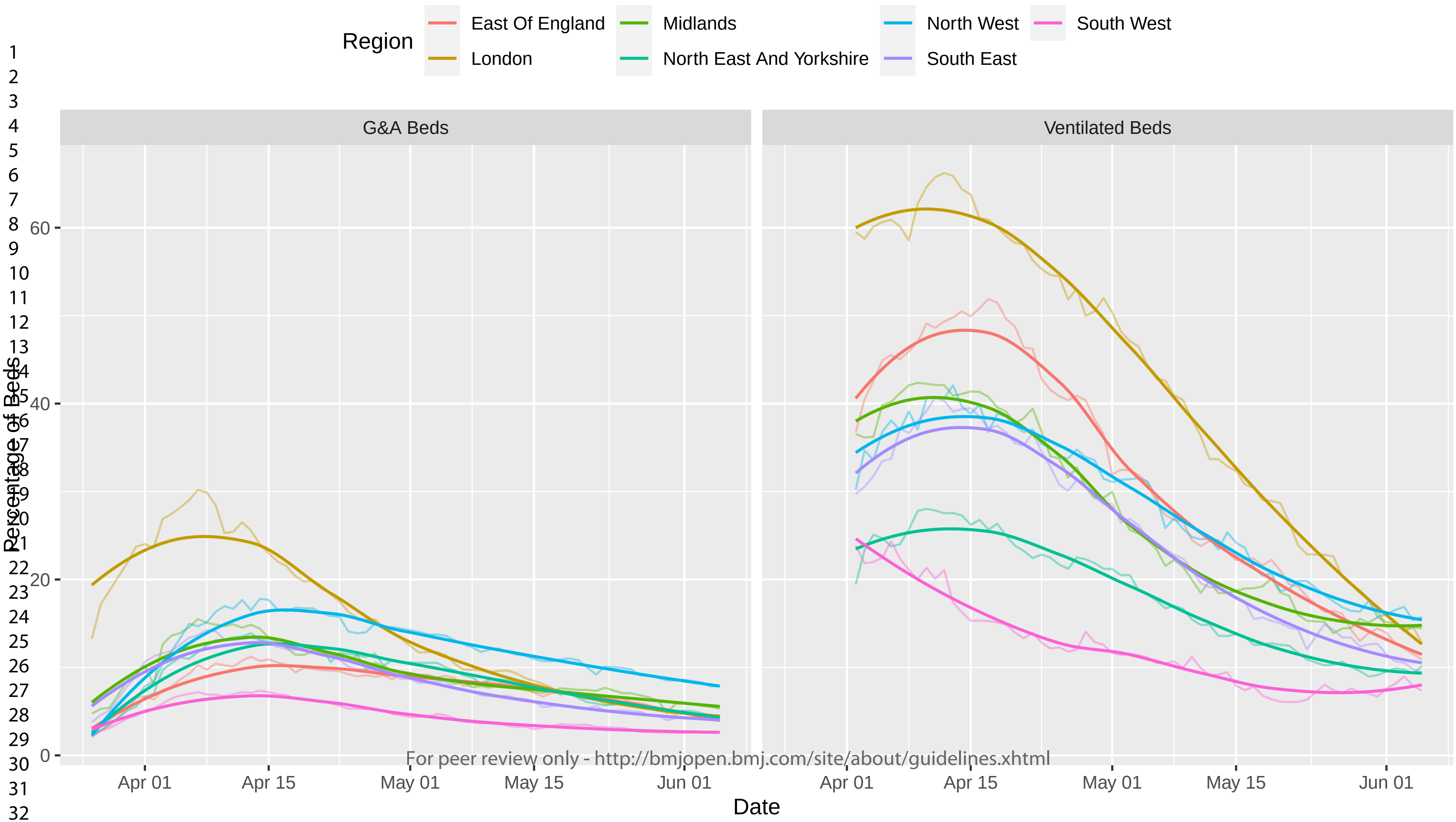

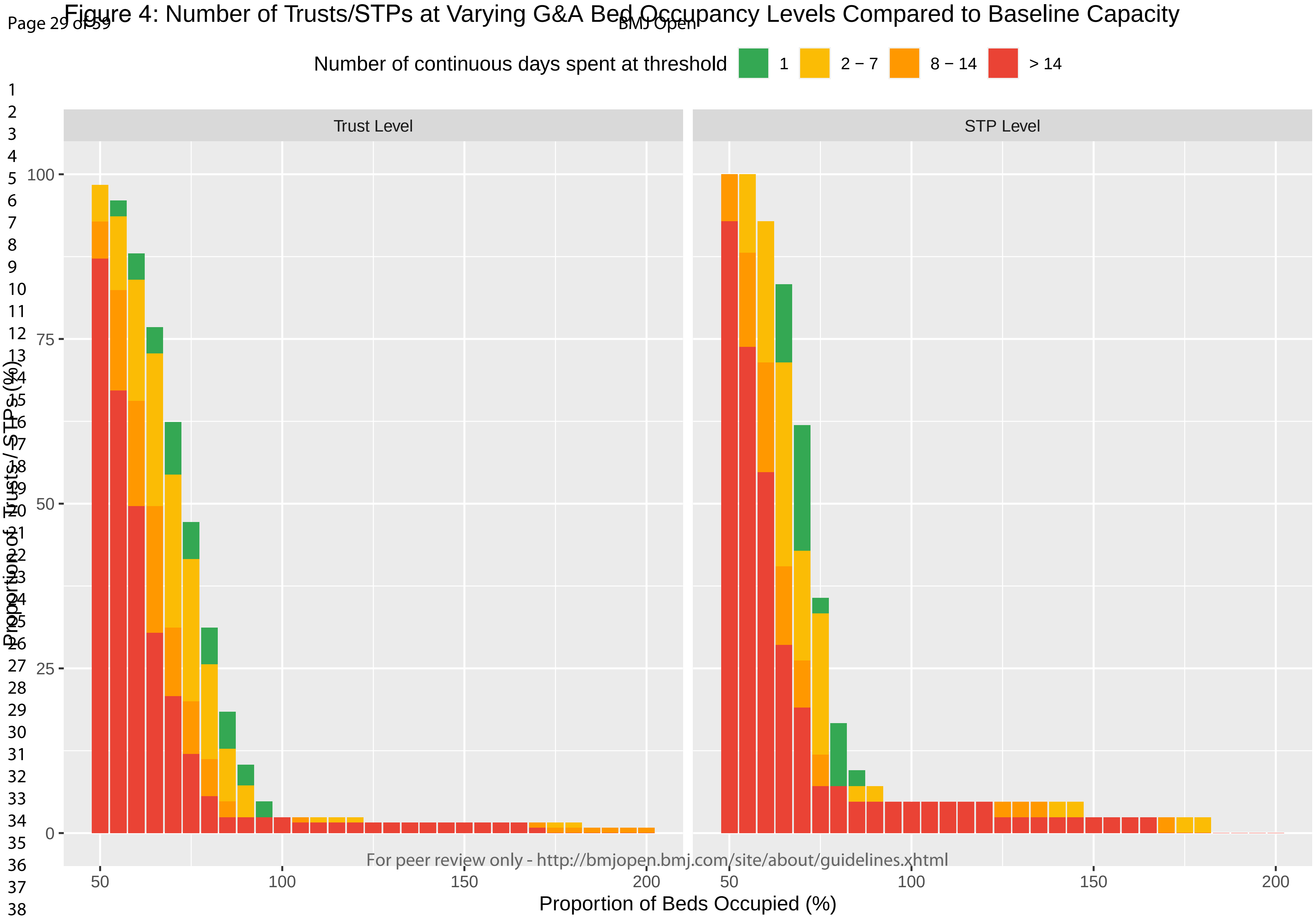
Figure 4: Number of Trusts/STPs at Varying G&A Bed Occupancy Levels Compared to Baseline Capacity
Number of continuous days spent at threshold
1
2 − 7
8 − 14
> 14
Trust Level
STP Level
100
75
50
25
0
50
100
150
200
Proportion of Trusts / STPs (%)
Proportion of Beds Occupied (%)

Figure 5: Number of Trusts/STPs at Varying Ventilated Bed Occupancy Levels Compared to Baseline Capacity

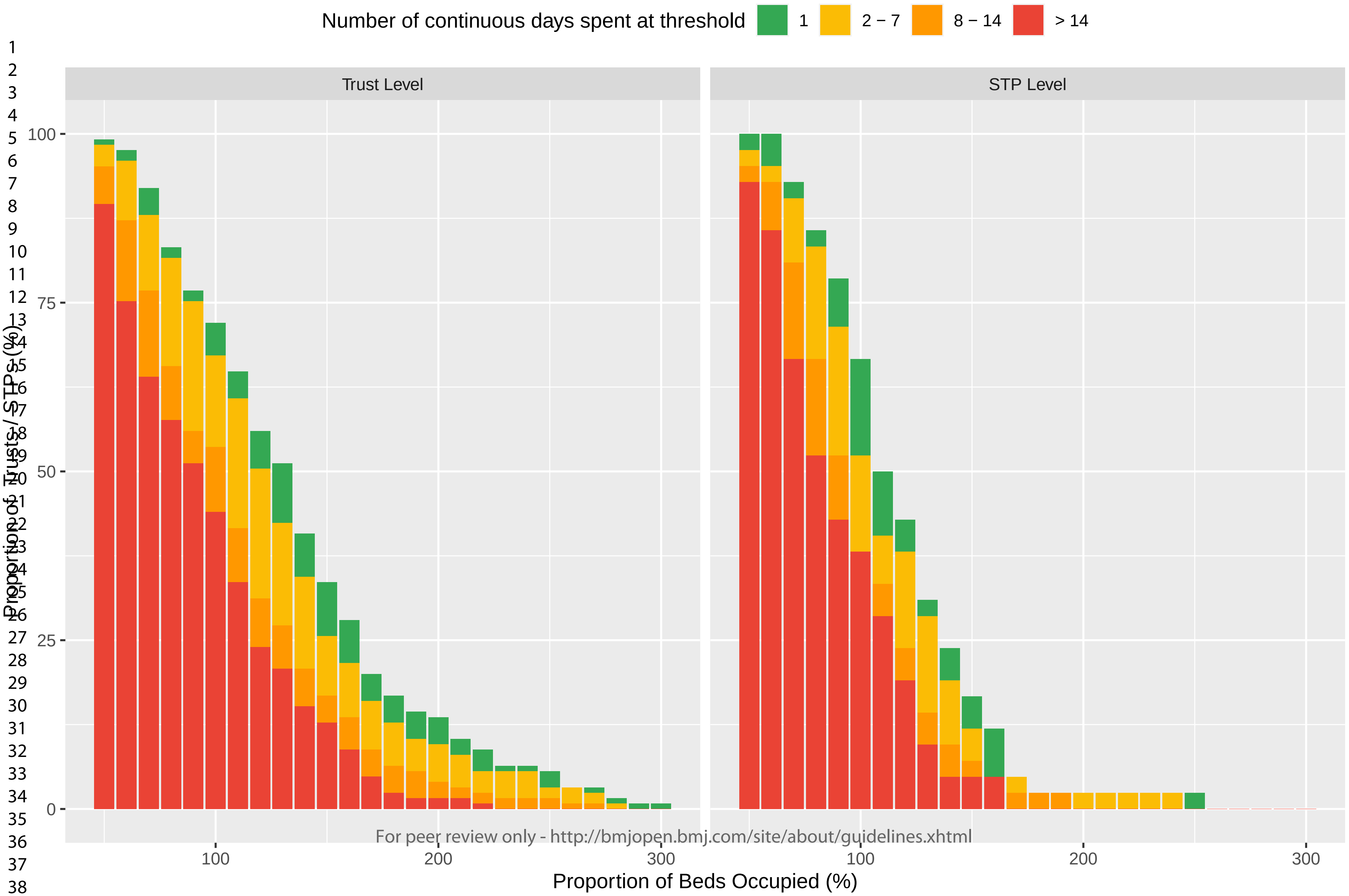

Figure 6: Number of Hospitals Operating Above Various G&A Bed Surge Capacity Thresholds

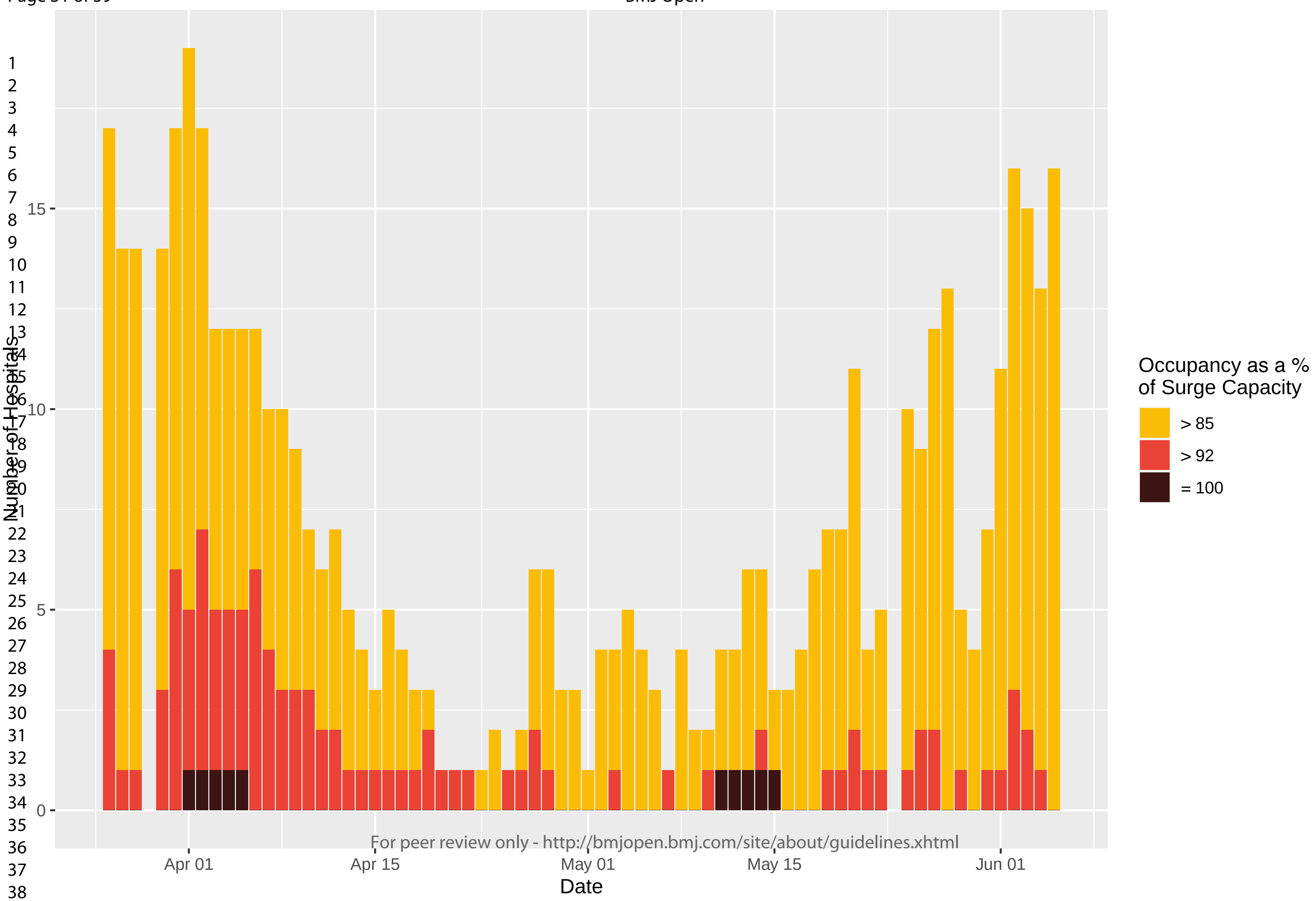

For peer review only - http://bmjopen.bmj.com/site/about/guidelines.xhtml

Figure 7: Number of Hospitals Operating Above Various Ventilated Bed Surge Capacity Thresholds

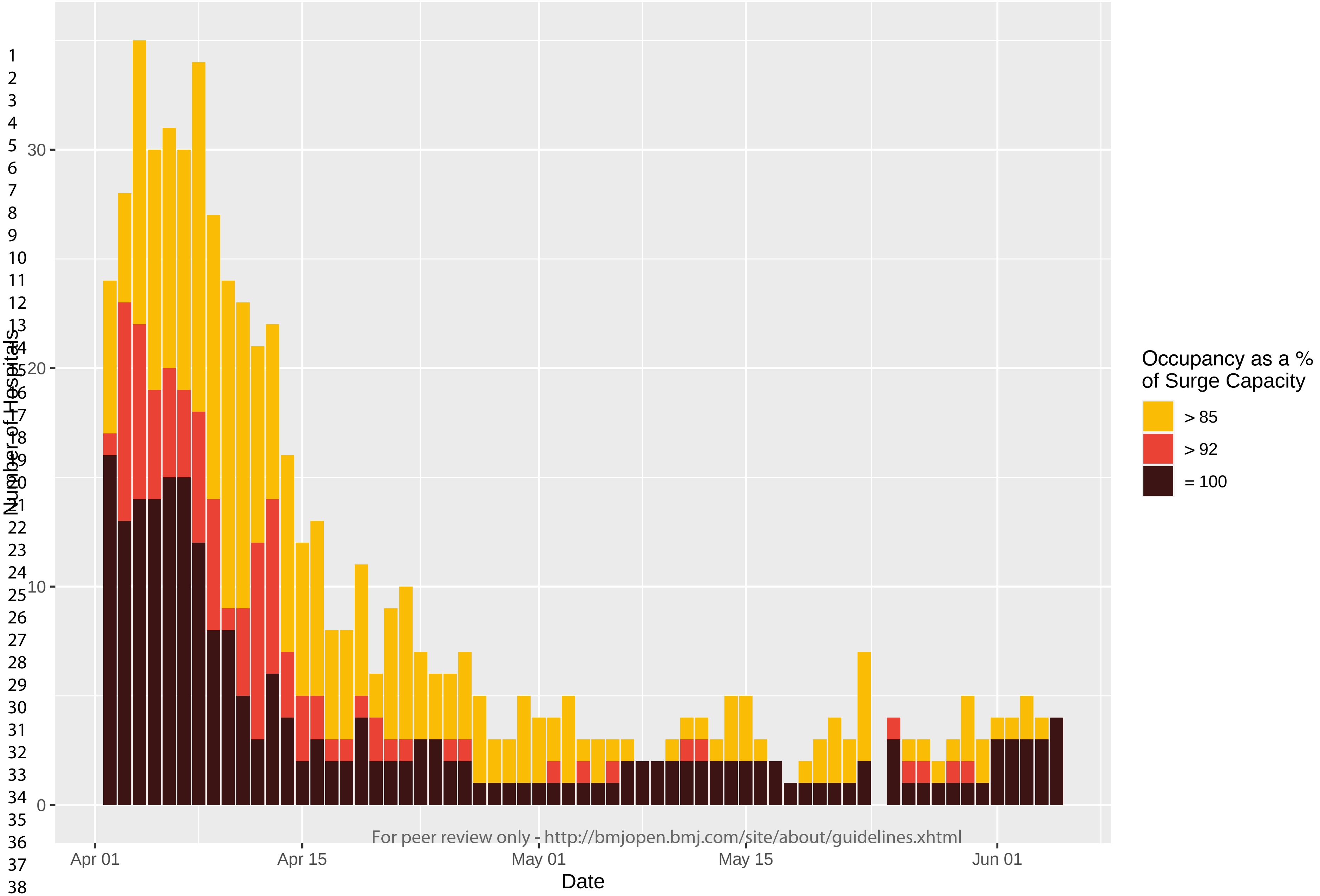

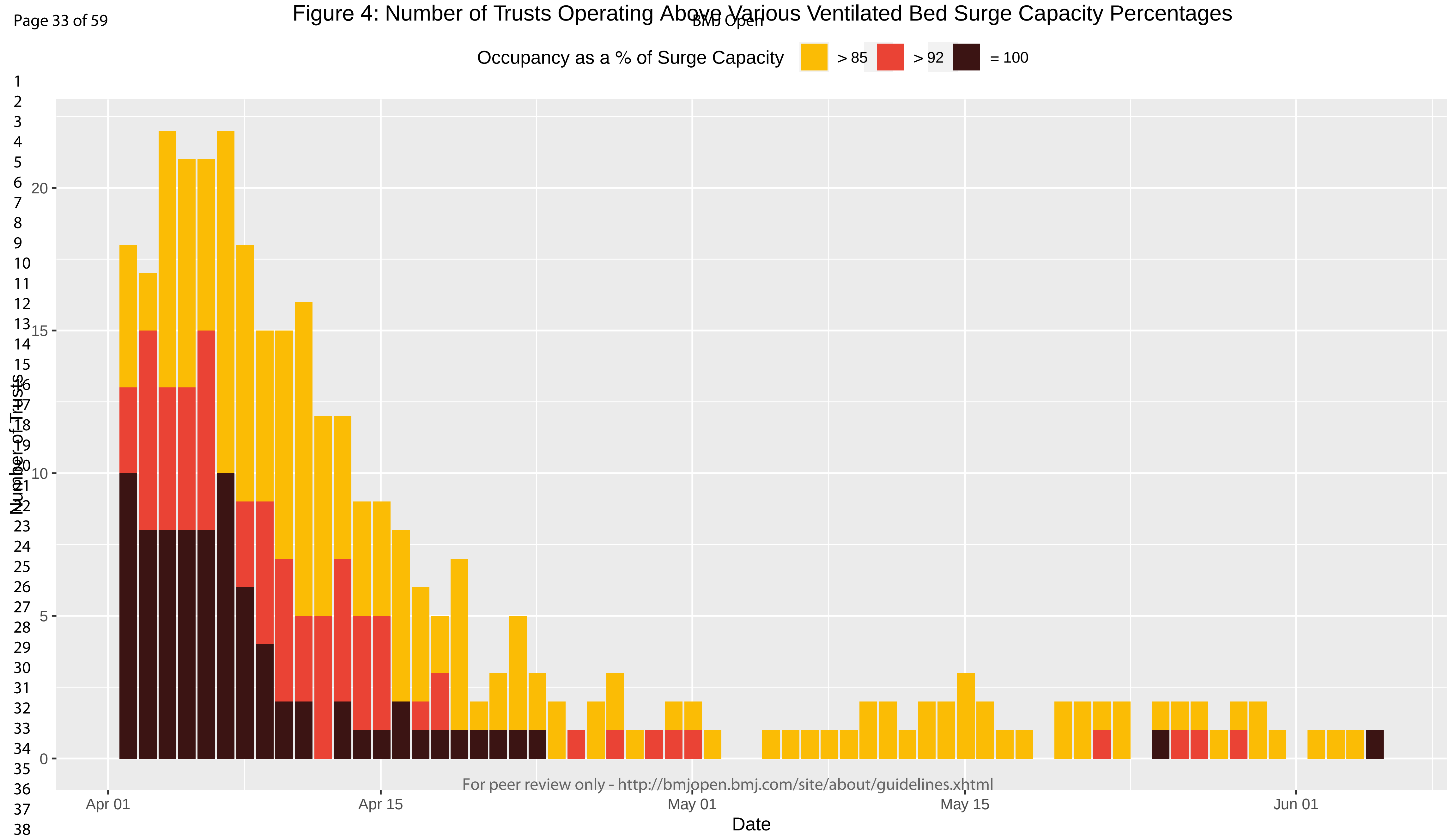

For peer review only - http://bmjopen.bmj.com/site/about/guidelines.xhtml

Figure 5: Peak STP Bed Occupancy Based on Surge Capacity

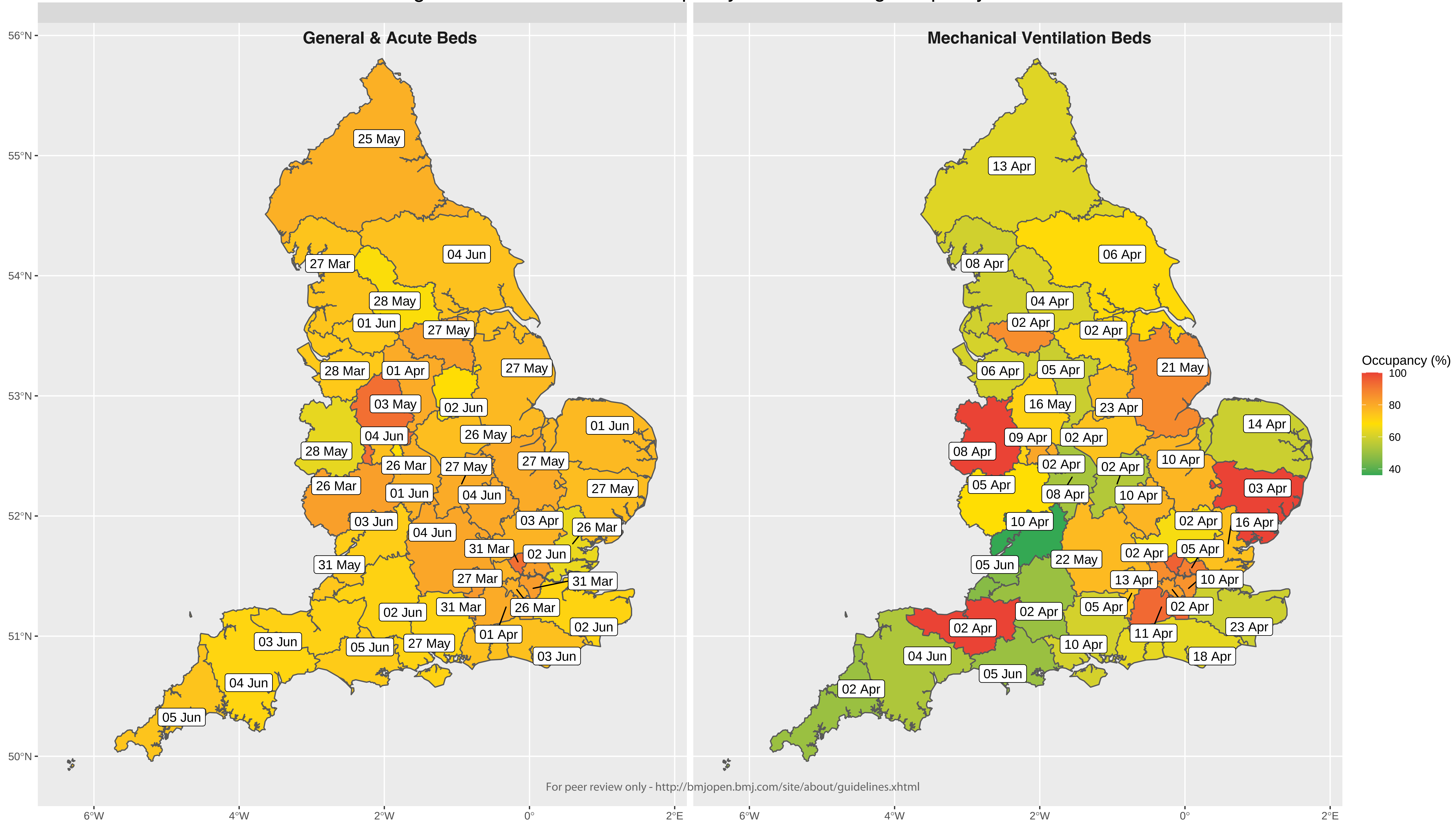

For peer review only - http://bmjopen.bmj.com/site/about/guidelines.xhtml

Supplementary Material

Supplementary Methods

Organizational Units of Healthcare Provision
Although hospitals are relatively self-explanatory, the remaining units may require further context for readers unfamiliar with the organization structure utilized in the UK, as such, the following are brief summaries of the higher-order units of healthcare provision. Trusts are the core functional unit of hospital-based (i.e. secondary) care provision in England. They represent the first-level of aggregation above individual sites/hospitals, i.e. a trust is a collection of 1 or more geographically co-located hospitals which for specific administrative reasons operate as a single entity, although individual hospitals retain differing degrees of financial and operational autonomy depending on the specific trust structure. STPs are the aggregated unit of trusts, in combination with other units of healthcare provision, such as Clinical Commissioning Groups which administer the portion of the healthcare budget allocated to a specific geographic locale. There are 42 STPs each mapped to a specific health-geography 'footprint', and their express purpose was to deliver improvements pertaining to efficacy of services and integration of geographically co-located care providers.[[35]] The STPs are then mapped to 7 distinct geographical regions across England.

COVID-19 Status Recording
Data reported with reference to COVID-19 status, for example the number of general and acute beds occupied by individuals with the infection, refers to those whom had a confirmed positive result from a reverse transcriptase polymerase chain reaction (PCR) of nasopharyngeal and/or oropharyngeal swab.[[36]\] PCR was the only available testing method during the study period. Although national testing policy changed throughout the study period, all people for whom there was a suspicion of COVID-19 infection and who were admitted to hospital were tested, and potentially re-tested multiple times if the initial results were negative but clinical suspicion remained high (recorded as suspected COVID; for the purposes of the subsequent analysis 'confirmed' and 'suspected' were treated as one group due to the relatively small numbers reported for the latter).

Historical Baselines for Bed Availability
Baseline data comprised: 1) the average number of general and acute (G&A) beds available between January-March 2020, sourced from previously published routine situation report (SitRep) data;[[37]] 2) the number of critical care beds prior to the first reported case of COVID-19 in the UK, i.e. the value reported on the 30th January 2020 Critical Care SitRep,[[38]\] and similarly to previous modelling studies was used for the baseline availabilities of HDU / ITU, critical care and ventilated beds;[[30]] 3) the maximum theoretical capacity for field hospitals was based on official government press releases;[[39,40]] and, 4) independent sector provider baseline capacity extracted from appendix 1 of the NHS England documentation confirming the 14 week block contract with the Independent Healthcare Providers Network.[[28]\] Baselines were available for all of the trusts from the sources mentioned above and was propagated through into the STP and Regional datasets alongside aggregation of other values. Baseline bed numbers were not available for site level data.

Quality Control
All of the data was acquired through the daily site reports provided by NHS-Improvement & NHS England. These reports were loaded and appended sequentially with checks to ensure consistency in headings and data composition. The data spanned multiple sheets; these sheets were joined using Hospital, Trust and STP level codes where appropriate. In some cases it was necessary to resort to using site names where no codes were present on the sheets containing hospital-level information regarding general and acute and critical care bed availability and occupancy. It was immediately apparent that extracting comprehensive site-level data from these records was non-trivial and for reasons discussed later, we maintain two datasets moving forward: one at site-level and one at trust-level that is used to aggregate to STP, Regional and Total figures as well.

Bed availability and total occupancy was recorded directly for G&A and critical care beds, alongside percentages of covid-confirmed occupants allowing for the calculation of a covid / non-covid / unoccupied breakdown for the G&A beds *only* (due to discrepancies in the definition of HDU / ITU and critical care beds, the percentage occupancy for critical care beds often resulted in impossible values of over 100%; it was decided to forego calculating a covid-breakdown for these beds due to how prolific these inconsistencies and issues were). For all of the other bed types, data was recorded in a different way. The number of covid positive patients (and in some cases covid-suspected patients), non-covid patients and the remaining unoccupied beds were

For peer review only - http://bmjopen.bmj.com/site/about/guidelines.xhtml

recorded, allowing for total occupancy and availability to be calculated through simple transformation of these columns.

There are two key dates and several more minor milestones in the period we have data for (26th March to 5th June) where significant, non-trivial changes occurred in the site report structure and content. Prior to April 1st there was no information on bed availability beyond G&A and Critical Care beds; only the number of covid-positive patients were recorded for each type of bed. After the 1st of April, more granular bed availability was provided along with the means to work out the covid/non-covid breakdown of occupancy for Ventilated beds. From the 27th April onwards similar breakdowns and availability were recorded for HDU / ITU, IDU and most other types of bed at a site-level.

After loading in the data and accounting for the above described changes to its composition, the trust-level data used for the majority of our analysis had:

- 8.7% of Ventilated bed non-covid and unoccupied numbers missing across all records (no missing records for covid occupancy)
- No missing records for G&A, Critical care bed availability and occupancy
- No missing records for HDU / ITU after April 26th, otherwise 45.0%
- All other columns containing information regarding the hospital, trust, etc. were complete

Both datasets were filtered to remove children's hospitals, mental health hospitals and other sites / trusts that were not relevant to the analysis. STP linkage data was acquired via NHS Digital's library of public datasets source: (https://digital.nhs.uk/services/organisation-data-service/data-downloads/other-nhs-organisations) and augmented to include populations within STPs to facilitate our "beds-per-capita" figures (values were scrapped manually from the NHS England website, source: https://www.england.nhs.uk/integratedcare/stps/view-stps/). It was found that 7 trust codes were duplicated across 2 STPs; it was inappropriate to double count them so they were arbitrarily assigned to one of the STPs. The following table contains the STP and Trust code pairs that were chosen / removed from the linkage data to ensure a one-to-one mapping:

STable 1: STP and trust code pairs (for duplicated trusts)

Trust Code	STP Code (Assigned)	STP Code (Removed)
RDU	QRL	QNQ
RFS	QJ2	QF7
RK9	QJK	QT6
RMC	QOP	QHM
RNN	QE1	QHM
RVR	QXU	QWE
RVY	QYG	QE1

Additionally, due to some trust-level mergers that took place and missing data in the source, 4 updated STP-Trust pairs were manually added to the linkage data to facilitate their inclusion in the analysis (source: https://www.england.nhs.uk/integratedcare/stps/view-stps/).

STable 2: Updated Trust-STP pairs (for merged trusts)

Trust Code	STP Code
RBA	QSL
RA3	QUY
RTP	QNX
RTK	QXU

Finally, it was found that two STPs spanned two regions. It was decided that QHM should fall under the North West region (all but one of its trusts are in that region) and QF7 should fall under the Midlands region (all but one of its trusts are in that region). The region definitions are inferred from the regions assigned to each trust in the site reports making up our primary dataset.

Despite our best efforts there were some missing values that persisted in critical columns outside of the key milestones mentioned in the section above. Moreover, in preparing the data it was noted that on several occasions there were substantial and improbable changes in the number of available beds that lasted 24 hours (even after allowing for the weekly trend of cyclical fluctuations in beds availability), prior to reversion to a value that fit the overall trend. These outliers follow from the reasonable assumption of the presence of data entry errors; it was decided that a cleaning rule should be applied to the data to avoid these seemingly impossible daily fluctuations and outliers.

First, a rolling median centred on each record was calculated using the 5 applicable days surrounding the record (smaller windows used at extremities of the data with correction not being possible at its absolute extremes). Missing values as well as values deemed to be outliers (a change greater than the 95th percentile of all differences between each record and the centred median spanning five days around it) were replaced with the aforementioned rolling median values. Highly improbable fluctuations were filtered out and missing values could be imputed in a robust way. This imputation and outlier detection process was applied to every applicable bed column spanning every type contained in the data. Only after this cleaning took place were other columns created through transformation, e.g. the number of available ventilated beds etc. The effect of cleaning the data is shown below in a before and after comparison, 4 trusts were chosen for their high initial volatility in G&A bed occupancy (See SFigure 1 & SFigure 2).

Statistical Analysis Notes
Temporalized values, i.e. hospital-days, were calculated by multiplying the absolute number of each functional unit for which data was available, and the number of days for which data is available for each.

After cleaning the data, two more key issues had to be dealt with in the trust-level and site-level datasets respectively:

1. Due to the aforementioned trust-level mergers, the composition of the data changed slightly throughout its duration. In an effort to achieve consistency, we merged and coalesced records prior to each mergers' appearance in the data to match their state post-merger. I.e. any rows corresponding to trusts that were eventually merged into some other trust were merged consistently throughout the dataset, even before this change actually took place. This was applied to records for the trusts RQ8, RDD and RAJ which were merged to fall under the single code RAJ on April 1st. This was also applied to RC9 and RC1 merged into RC9, and RA7 and RA3 merged into RA7: mergers that also occurred on April 1st and were reflected in the data shortly after.
2. It was observed that in one of the sheets relied upon for ventilated bed numbers, separate rows were included for both the sites and the corresponding trusts (given a "catch-all" label as their organisation type rather than "site"). In cases where only one hospital was associated with a trust, the numbers for that hospital were sometimes - inconsistently - recorded in the catch-all row rather than the site row as was done fairly consistently across all other situations. To achieve consistency without losing significant portions of the site-level data, we coalesced those rows where only one site was present and the catch all row contained numbers whilst the site row had zeroes or missing values. In order to achieve this, the organisation types of the two rows were swapped so that the catch-all row would be used in place of the site row, such that the site code and name was consistent throughout the entirety of the data.

Data Limitations
One persistent concern was the formulas by which bed occupancy proportions were generated. For example, the COVID-19 specific G&A bed percentage-occupancy was initially calculated as the sum of COVID-19 patients in IDU (infectious disease unit) beds and COVID-19 patients in "any other beds" divided by the total number of available G&A beds. This eventually changed to being the sum of the number of mechanical ventilated beds, non-invasive ventilated beds, oxygen-supporting beds and "any other beds" occupied by COVID patients minus the number of HDU / ITU beds occupied by confirmed COVID patients, all divided by the total number of available G&A beds. Whilst this is not in-and-of-itself problematic, the nature of the "any other beds" item was deemed concerning by the authors.

For peer review only

To understand the aforementioned concern, we first need to explain the data specification in more detail. It was noted that columns of the form "Number of Covid-19 confirmed patients in … beds at 0800" did not seem to contain values consistent with "Number of … beds available, as at 08:00 (COVID)", which we expected to have mirrored values. Importantly, the latter set of columns did not contain an "any other bed" column. As such, the formula used by NHS-E in the above calculation of G&A bed proportions drew the "any other beds" value from the first set of data, whereas all of the other information was drawn from the latter columns as they were internally consistent. We acknowledge that the use of this formula could have introduced an error of unknown magnitude or direction (as the two versions of data reporting were not consistent). Similar issues were seen with the independent sector data as well.

For peer review only

SFigure 1: G&A Bed Availability Across the Most Volatile Trusts in Terms of G&A Bed Occupancy (Pre−Correction)

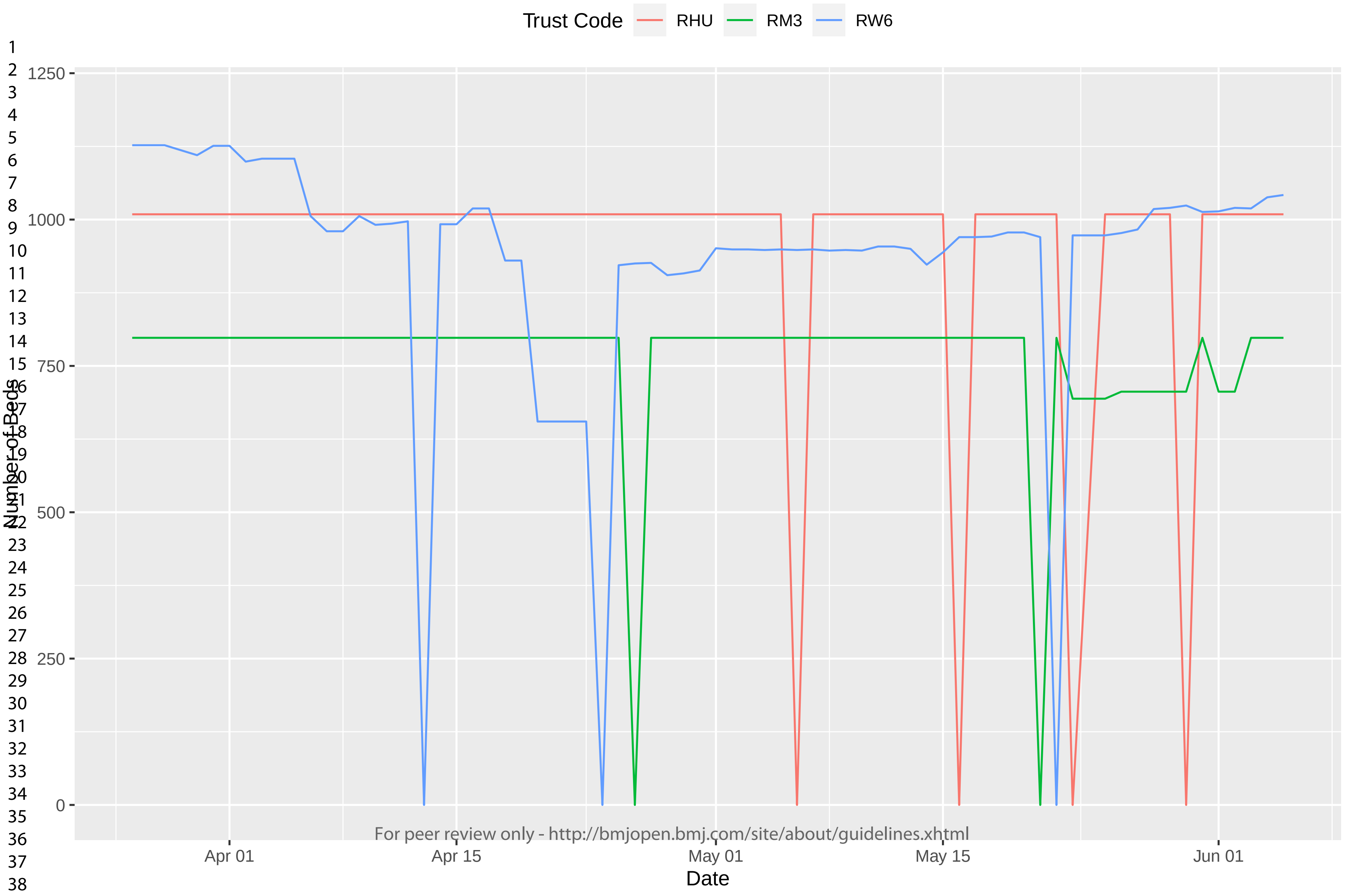

For peer review only - http://bmjopen.bmj.com/site/about/guidelines.xhtml

SFigure 2: G&A Bed Availability Across the Most Volatile Trusts in Terms of G&A Bed Occupancy (Post−Correction)

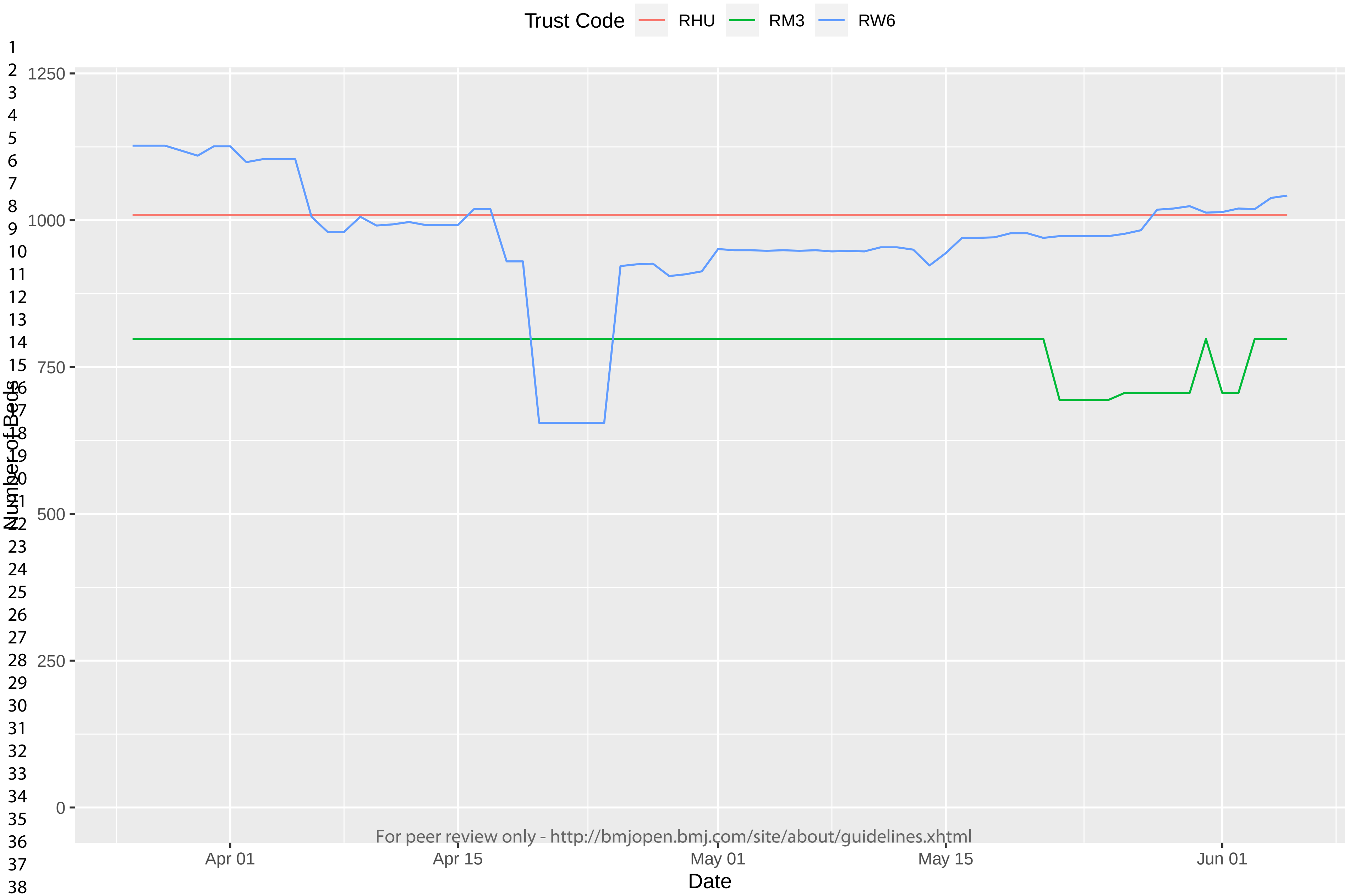

Supplementary Results

STable 1: Descriptive Summaries of the Size and Geographic Locations of Hospitals Stratified by the Peak Occupancy Achieved

	**Hospitals reaching 100% saturation of mechanical ventilation beds (n = 51*)**	**Hospitals reaching >92%, but not 100% saturation of mechanical ventilation bed (n = 20)**	**Hospitals reaching >85%, but not 92% saturation of mechanical ventilation bed (n = 19)**	**All other Hospitals (n = 77**)**
**Peak G&A bed capacity (Median [Range])**	438 [197 - 1012]	484 [256 - 841]	459 [253 - 910]	558 [44 - 1499]
**Peak mechanical ventilation compatible bed capacity (restricted to April 2nd onwards) (Median [Range])**	29 [7 - 77]	49 [18 - 141]	33 [15 - 153]	40 [7 - 159]
**Peak HDU/ITU bed capacity (restricted to April 27th onwards) (Median [Range])**	29 [0 - 99]	45 [17 - 161]	37 [14 - 157]	40 [6 - 152]
Location				
England (n = 167)	***51 (30.5%)***	***20 (12.0%)***	***19 (11.4%)***	***77 (46.1%)***
**London (n = 27)**	6 (22.2%)	11 (40.7%)	7 (25.9%)	3 (11.1%)
**Midlands (n = 27)**	11 (40.7%)	3 (11.1%)	1 (3.7%)	12 (44.4%)
**East of England (n = 20)**	7 (35.0%)	2 (10.0%)	1 (5.0%)	10 (50.0%)
**South West (n = 17)**	3 (17.6%)	0 (0.0%)	0 (0.0%)	14 (82.3%)
**South East (n = 24)**	6 (25.0%)	2 (8.3%)	3 (12.5%)	13 (54.2%)
**North East and Yorkshire (n = 29)**	8 (27.6%)	1 (3.4%)	4 (13.8%)	16 (65.5%)
**North West (n = 23)**	10 (43.5%)	1 (4.3%)	3 (13.0%)	9 (39.1%)

* One hospital is excluded from this n and the subsequent calculations as it does not have any information regarding G&A beds, despite reaching 100% capacity for mechanical ventilator bed capacity.
** 5 hospitals were excluded from this table as they had no ventilated beds at any time, or no data was available for their ventilated bed capacity in the dataset.

For peer review only

SFigure 3: Regional Bed Occupancy Across England, Stratified by COVID-19 Status

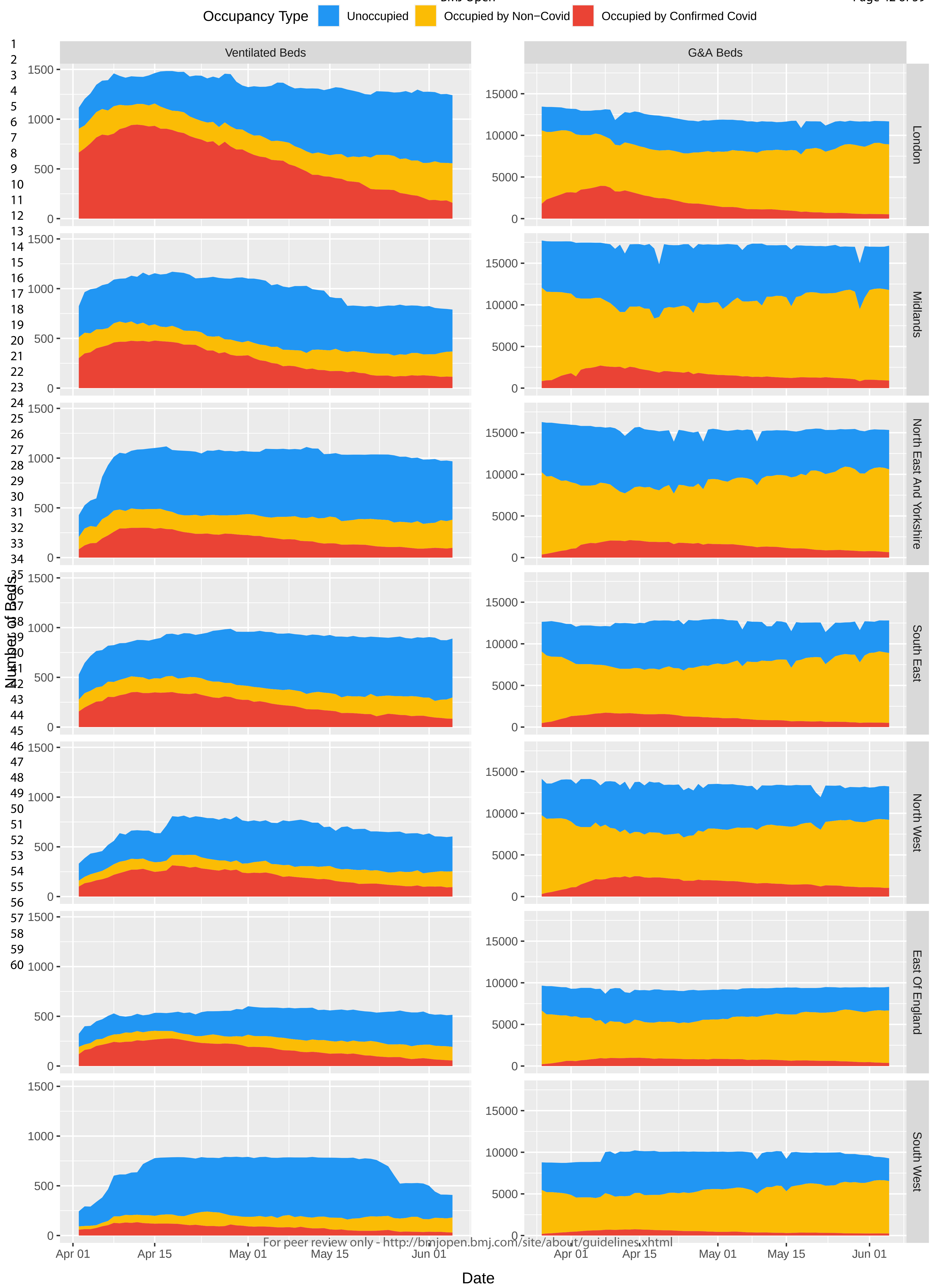

SFigure 3: Bed Occupancy Across England by geographical region, stratified by COVID-19 Status

Legend: SFigure 3A (Left) illustrates the time-varying trends in mechanical ventilator bed capacity and occupancy across the 7 regions of England, from April 1st o June 5th; note that availability information is only present from 1st April onwards. SFigure 3B (Right) illustrates general and acute bed capacity and occupancy across the 7 regions of England, from March 27th to 5th June. Occupancy in both figures is stratified by whether the individual in the bed has a positive COVID-19 test or not.

For peer review only

Critical Care Beds

In the context of surge capacity, at the site-level, 1558 hospital days (13.1%; median number of days per hospital = 10 [range: 1 to 65]) were at or above 85% of capacity, which corresponds to 120 hospitals spending at least 1 day at, or above, the aforementioned threshold. 948 hospital days (8.0%; median number of days per hospital = 6 [range: 1 to 51]) were spent above 92%, representing 102 hospitals. And 88 (50.9%) hospitals reached 100% capacity, representing 640 hospital days at saturation (median number of days per hospital = 5 [range: 1 to 51])). At the trust-level, 965 trust days (11.0%; median number of days per trust = 8 [range: 1 to 56]) were at or above 85% of capacity, representing 80 trusts. 567 trust days (6.5%; median number of days per trust = 5 [range: 1 to 47]) were spent above 92%, representing 64 trusts. And 47 (37.6%) trusts reached 100% capacity, representing 339 trust days at saturation (median number of days per trust = 5 [range: 1 to 21])). At the STP-level, 138 STP days (median number of days per STP = 5 [range: 1 to 43]) were at or above 85% of capacity, representing 18 STPs. 74 STP days (median number of days per STP = 2 [range: 1 to 26]) were spent above 92%, representing 14 STPs. And 6 STPs reached 100% capacity, representing 34 STP days at saturation (median number of days per STP = 3 [range: 1 to 19])). See SFigure 4 for a visual summary of these results. See SFigure 5 for the aggregate occupancy, stratified by COVID-19 status at the regional level.

In the context of baseline capacity, at the trust-level, 2620 trust days (22.1%; median number of days per trust = 27 [range: 1 to 69]) were at or above 100% capacity, which corresponds to 92 trusts spending at least 1 day at, or above, their-pre-pandemic baseline. 230 trusts days (median number of days per trust = 9 [range: 1 to 49]) were at or above 200% capacity, which corresponds to 19 trusts spending at least 1 day more than 100% above their-pre-pandemic baseline. At the STP-level, 620 STP days (median number of days per STP = 24 [range: 1 to 63]) were at an occupancy-level above 100% of baseline availability, which corresponds to 27 STPs spending at least 1 day at, or above, their-pre-pandemic baseline. 44 STP days (median number of days per STP = 14 [range: 10 to 20]) were at an occupancy-level above 200% of baseline availability which corresponds to 3 STPs spending at least 1 day more than 100% above their-pre-pandemic baseline. See SFigure 8 for a visual summary.

HDU/ITU Beds

The following results should be interpreted in the context of the date range available, i.e. data is only present after the 27th of April. Thus, the results are likely a significant underestimation of peak occupancy as, in retrospect, the peak number of cases and fatalities in the UK were near the beginning of April.

In the context of surge capacity, at the site-level, 315 hospital days (2.7%; median number of days per hospital = 2 [range: 1 to 39]) were at or above 85% of capacity, which corresponds to 59 hospitals spending at least 1 day at, or above, the aforementioned threshold. 216 hospital days (1.8%; median number of days per hospital = 1 [range: 1 to 39]) were spent above 92%, representing 45 hospitals. And 40 hospitals reached 100% capacity, representing 192 hospital days at saturation (median number of days per hospital = 1 [range: 1 to 39])). At the trust-level, 192 trust days (median number of days per trust = 3 [range: 1 to 39]) were at or above 85% of capacity, representing 36 trusts. 122 trust days (median number of days per trust = 2 [range: 1 to 39]) were spent above 92%, representing 24 trusts. And 19 trusts reached 100% capacity, representing 106 trust days at saturation (median number of days per trust = 2 [range: 1 to 39])). At the STP-level, 138 STP days (median number of days per STP = 3 [range: 1 to 43]) were at or above 85% of capacity, representing 18 STPs. 74 STP days (median number of days per STP= 2 [range: 1 to 26]) were spent above 92%, representing 14 STPs. And 6 STPs reached 100% capacity, representing 34 STP days at saturation (median number of days per STP = 3 [range: 1 to 19])). See SFigure 5 for a visual summary.

For peer review only

SFigure 4a: Number of Hospitals/Trusts/STPs at Varying Critical Care Bed Occupancy Levels Compared to Surge Capacity

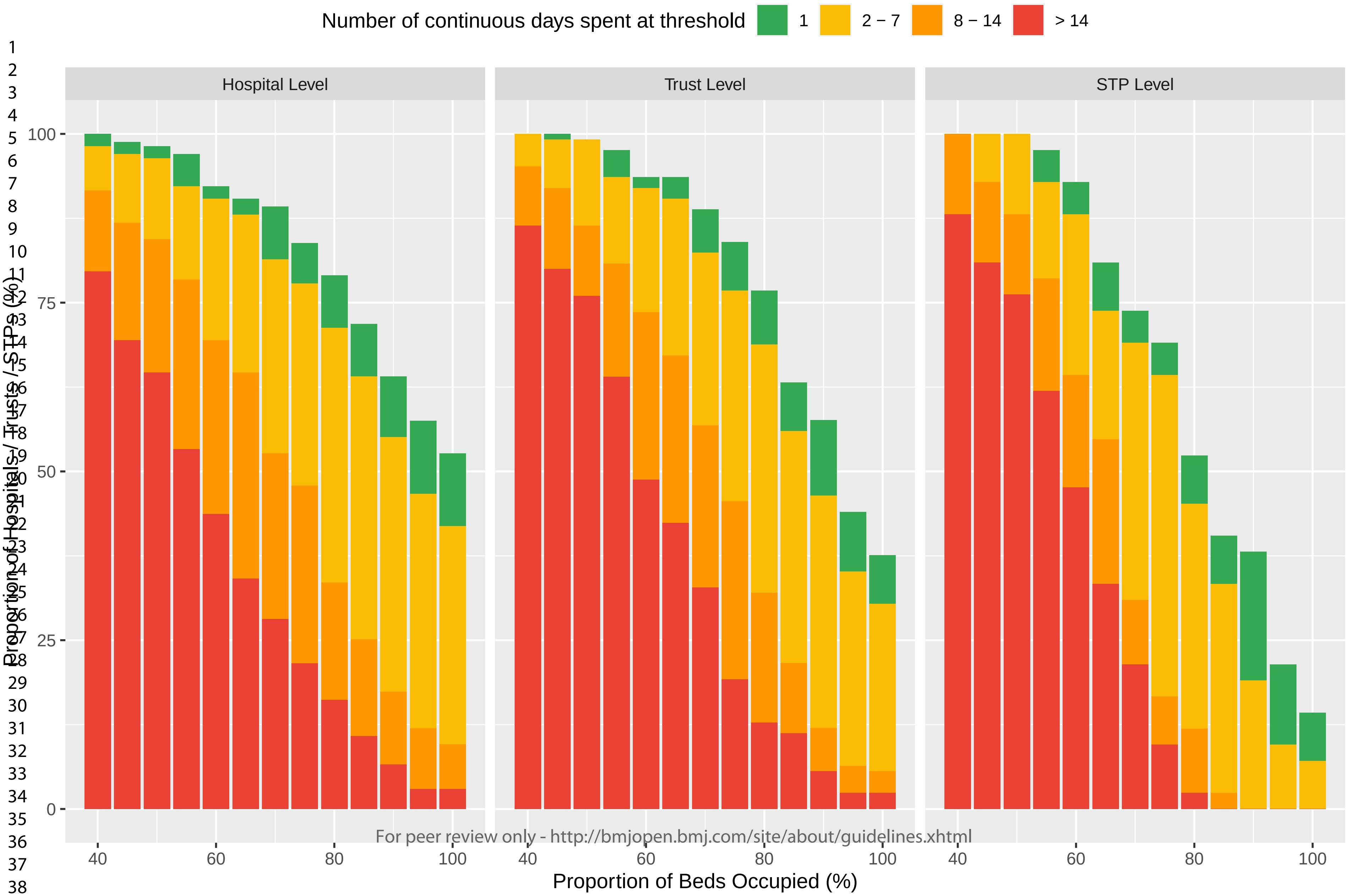

SFigure 4b: Number of Hospitals/Trusts/STPs at Varying HDU/ITU Bed Occupancy Levels Compared to Surge Capacity

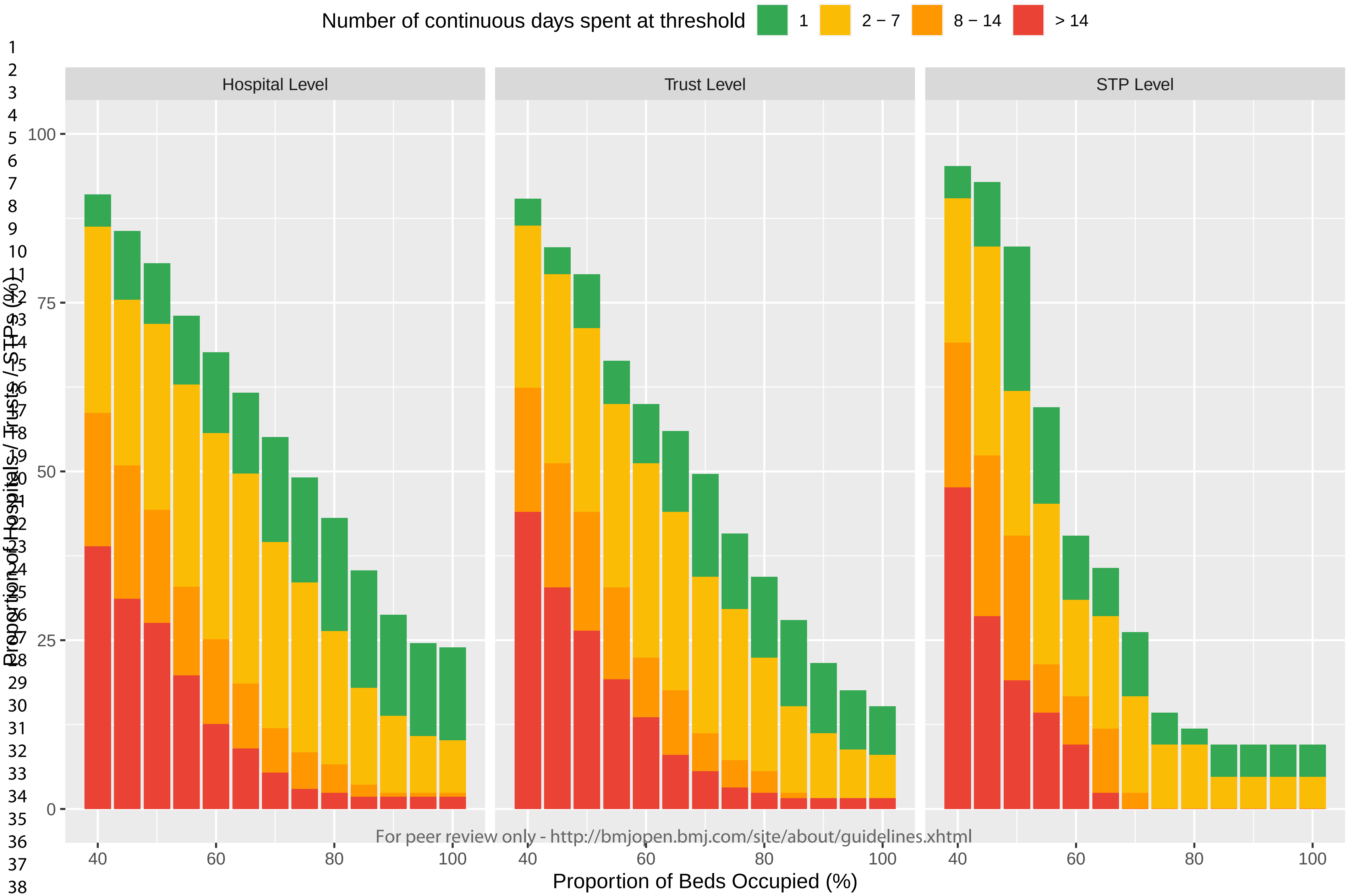

For peer review only - http://bmjopen.bmj.com/site/about/guidelines.xhtml

For peer review only

SFigure 4: Critical Care (Top) & HDU/ITU (Bottom) Occupancy (Based on Surge Capacities) Across England

Legend: SFigure 4A (Top) illustrates the proportion of hospitals/trusts/STPs at different occupancy thresholds for surge critical care bed capacity, across England, from April 1st to June 5th. SFigure 4B (Bottom) illustrates the proportion of hospitals/trusts/STPs at different occupancy thresholds for surge critical care bed capacity, across England, from April 1st to June 5th. The superimposed colours represent how long the trusts spent at each specific threshold.

SFigure 5: Regional Critical Care Bed Occupancy, Stratified by COVID-19 Status

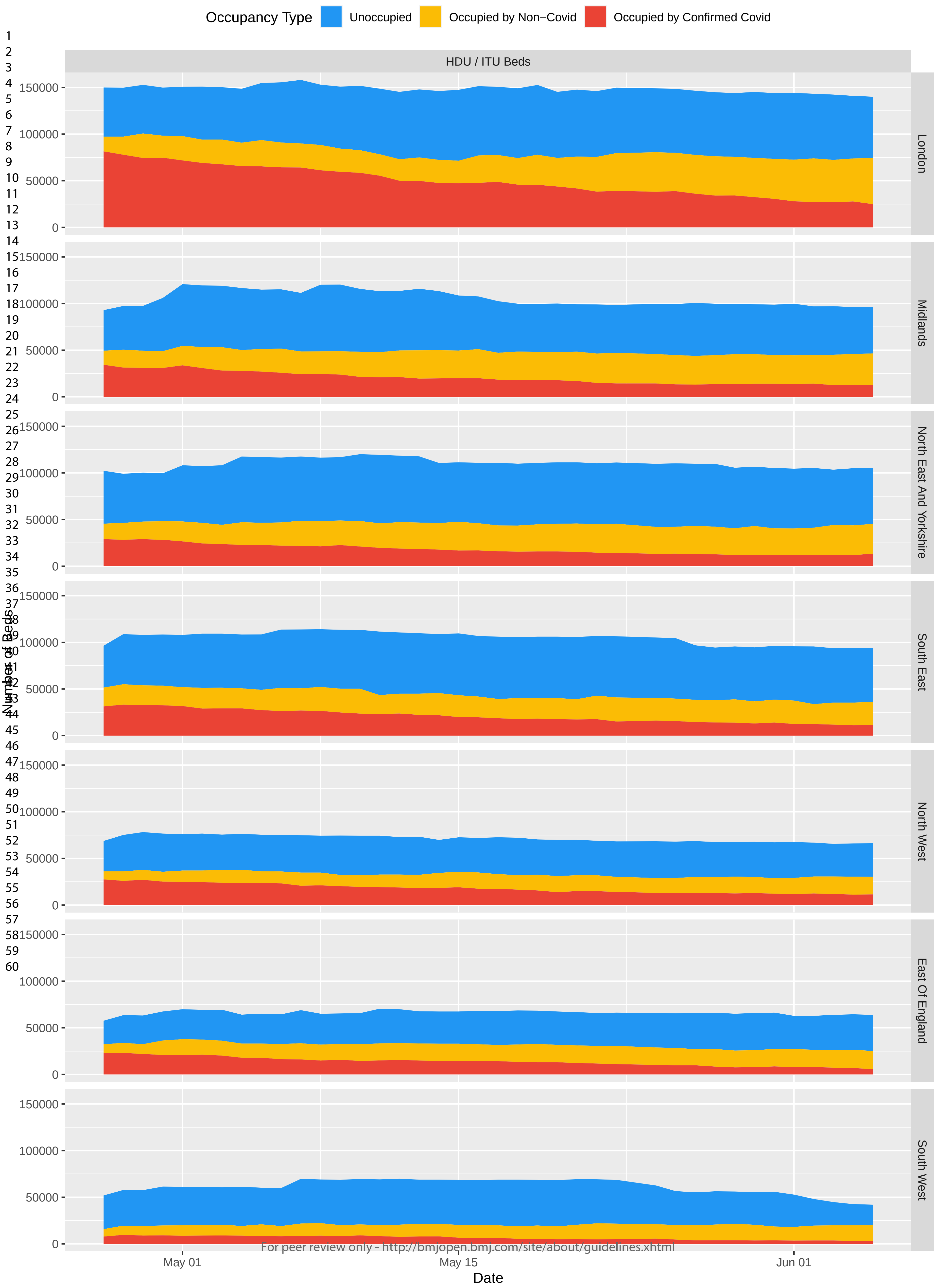

SFigure 5: Regional Critical Care Bed Occupancy, Stratified by COVID-19 Status

Legend: SFigure 5 illustrates the time-varying trends in critical care bed capacity and occupancy across the 7 regions of England, from March 27th to June 5th. Occupancy is stratified by whether the individual in the bed has a positive COVID-19 test or not.

For peer review only

SFigure 6: Proportion of Trusts/STPs at Varying Critical Care Bed Occupancy Levels Compared to Baseline Capacity

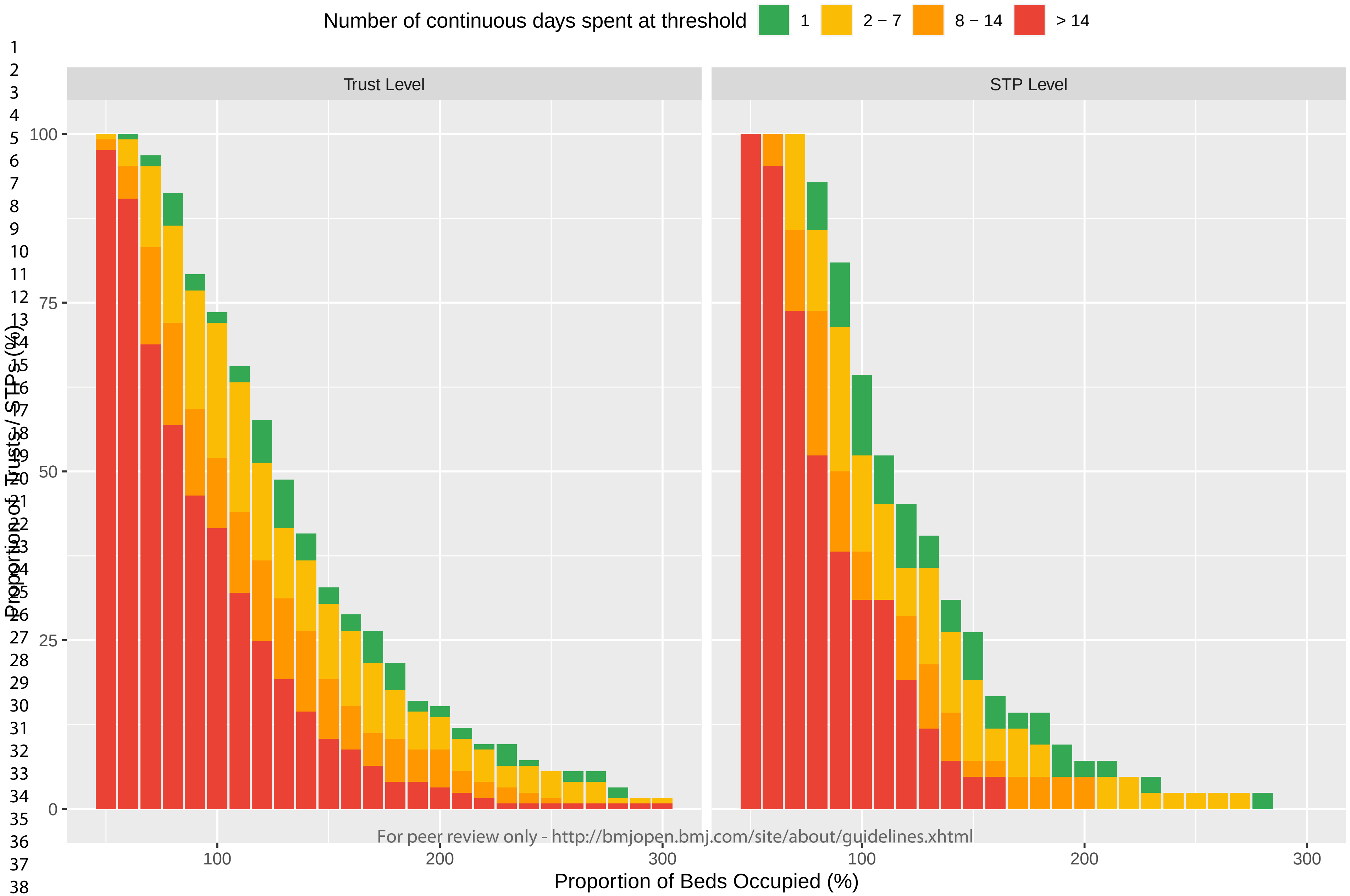

For peer review only - http://bmjopen.bmj.com/site/about/guidelines.xhtml

SFigure 6: Critical Care Bed Occupancy (Based on Baseline Capacities) Across England

Legend: SFigure 6A (Left) illustrates the proportion of trusts at different occupancy thresholds based on baseline critical care bed capacity, across England, from April 1st to June 5th. SFigure 6B (Right) illustrates the proportion of STPs at different occupancy thresholds based on their baseline critical care bed capacity, across England, from April 1st to June 5th. The superimposed colours represent how long the trusts spent at each specific threshold.

For peer review only

Number of Trusts Operating Above Various Ventilated Bed Surge Capacity Percentages

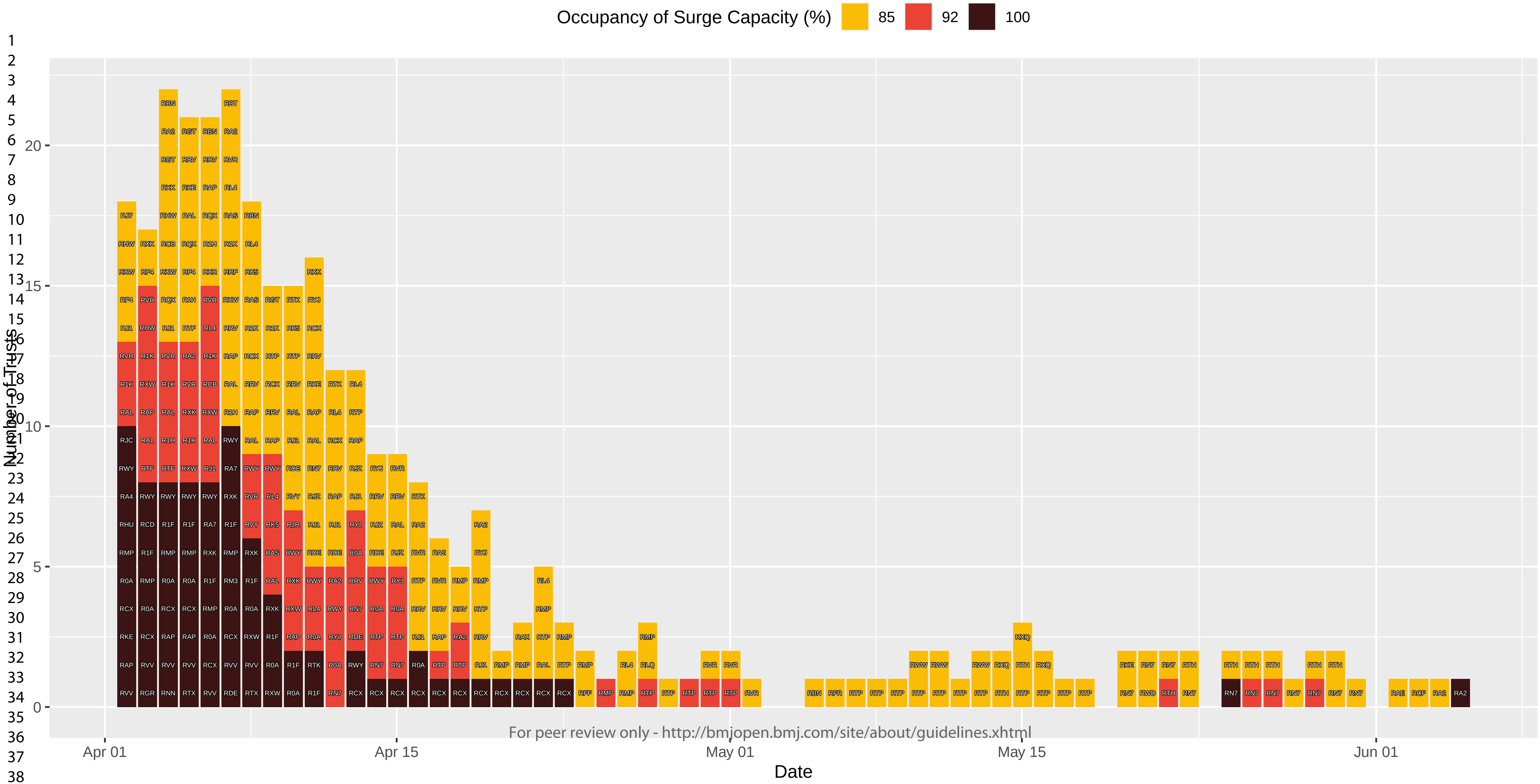

For peer review only - http://bmjopen.bmj.com/site/about/guidelines.xhtml

SFigure 7: Trust-Level Ventilator Bed Occupancy (Based on Surge Capacities) Across England

Legend: The conversion of trust code to name for all trusts included in the figure are: Manchester University NHS Foundation Trust (R0A), Isle Of Wight NHS Trust (R1F), Barts Health NHS Trust (R1H), London North West University Healthcare NHS Trust (R1K), Royal Surrey County Hospital NHS Foundation Trust (RA2), Yeovil District Hospital NHS Foundation Trust (RA4), University Hospitals Bristol NHS Foundation Trust (RA7), University Hospitals Bristol And Weston NHS Foundation Trust (RA7), Bradford Teaching Hospitals NHS Foundation Trust (RAE), Royal Free London NHS Foundation Trust (RAL), North Middlesex University Hospital NHS Trust (RAP), The Hillingdon Hospitals NHS Foundation Trust (RAS), Kingston Hospital NHS Foundation Trust (RAX), St Helens And Knowsley Teaching Hospitals NHS Trust (RBN), Mid Cheshire Hospitals NHS Foundation Trust (RBT), York Teaching Hospital NHS Foundation Trust (RCB), Harrogate And District NHS Foundation Trust (RCD), Airedale NHS Foundation Trust (RCF), The Queen Elizabeth Hospital, King's Lynn, NHS Foundation Trust (RCX), East Suffolk And North Essex NHS Foundation Trust (RDE), Barking, Havering And Redbridge University Hospitals NHS Trust (RF4), Barnsley Hospital NHS Foundation Trust (RFF), The Rotherham NHS Foundation Trust (RFR), West Suffolk NHS Foundation Trust (RGR), Cambridge University Hospitals NHS Foundation Trust (RGT), Portsmouth Hospitals NHS Trust (RHU), Royal Berkshire NHS Foundation Trust (RHW), Guy's And St Thomas' NHS Foundation Trust (RJ1), St George's University Hospitals NHS Foundation Trust (RJ7), South Warwickshire NHS Foundation Trust (RJC), Countess Of Chester Hospital NHS Foundation Trust (RJR), King's College Hospital NHS Foundation Trust (RJZ), Sherwood Forest Hospitals NHS Foundation Trust (RK5), Whittington Health NHS Trust (RKE), The Royal Wolverhampton NHS Trust (RL4), Wye Valley NHS Trust (RLQ), Salford Royal NHS Foundation Trust (RM3), Tameside And Glossop Integrated Care NHS Foundation Trust (RMP), Dartford And Gravesham NHS Trust (RN7), North Cumbria Integrated Care NHS Foundation Trust (RNN), Homerton University Hospital NHS Foundation Trust (RQX), Wrightington, Wigan And Leigh NHS Foundation Trust (RRF), University College London Hospitals NHS Foundation Trust (RRV), Northumbria Healthcare NHS Foundation Trust (RTF), Oxford University Hospitals NHS Foundation Trust (RTH), Ashford And St Peter's Hospitals NHS Foundation Trust (RTK), Surrey And Sussex Healthcare NHS Trust (RTP), University Hospitals Of Morecambe Bay NHS Foundation Trust (RTX), Epsom And St Helier University Hospitals NHS Trust (RVR), East Kent Hospitals University NHS Foundation Trust (RVV), Southport And Ormskirk Hospital NHS Trust (RVY), United Lincolnshire Hospitals NHS Trust (RWD), Warrington And Halton Hospitals NHS Foundation Trust (RWW), Calderdale And Huddersfield NHS Foundation Trust (RWY), Sandwell And West Birmingham Hospitals NHS Trust (RXK), Buckinghamshire Healthcare NHS Trust (RXQ), East Lancashire Hospitals NHS Trust (RXR), Shrewsbury And Telford Hospital NHS Trust (RXW), Imperial College Healthcare NHS Trust (RYJ)

For peer review only

SFigure 8a: Proportion of Hospitals/Trusts/STPs at Varying Ventilated Bed Occupancy Levels Compared to Surge Capacity

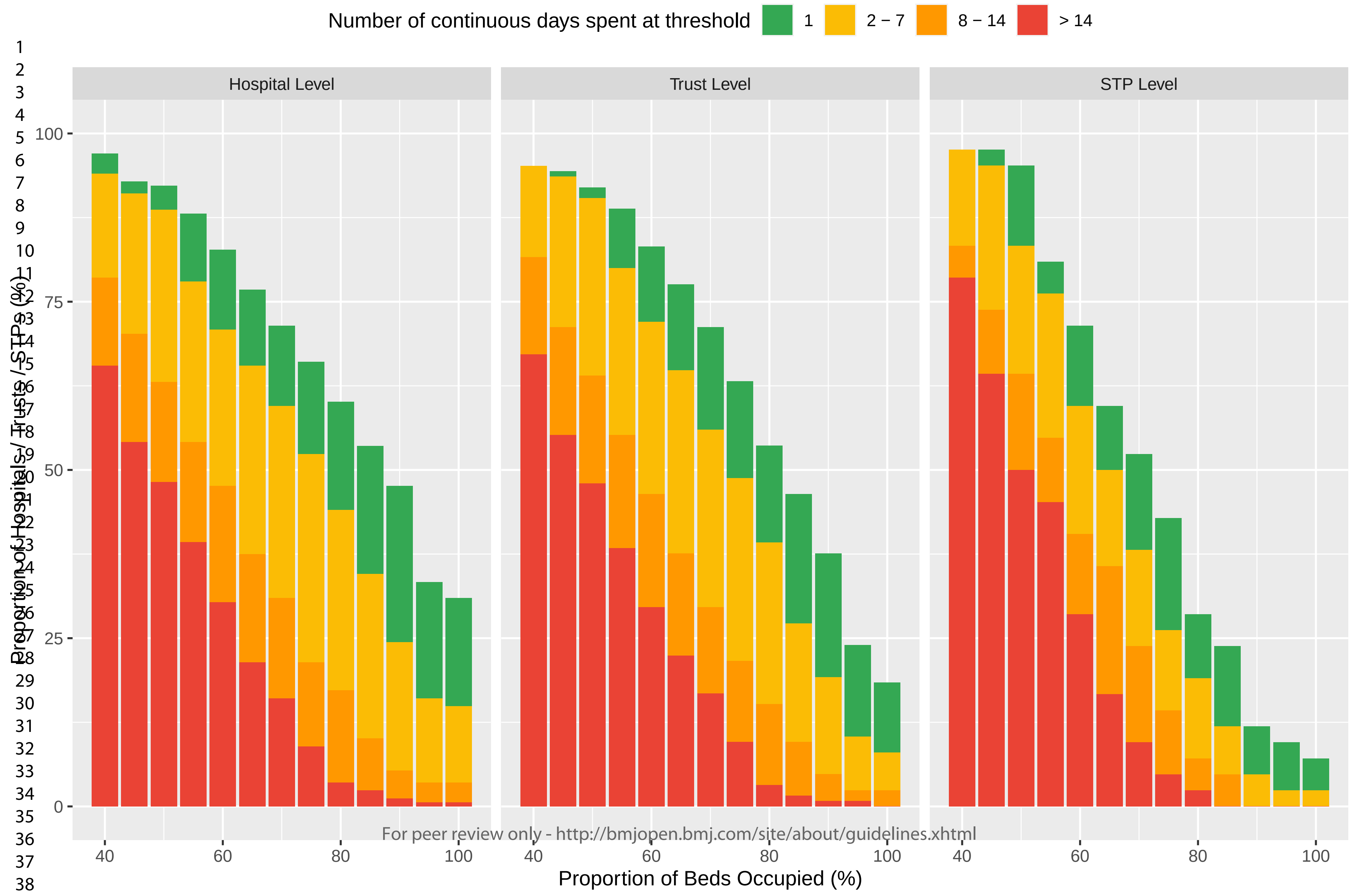

For peer review only - http://bmjopen.bmj.com/site/about/guidelines.xhtml

SFigure 8b: Proportion of Hospitals/Trusts/STPs at Varying G&A Bed Occupancy Levels Compared to Surge Capacity

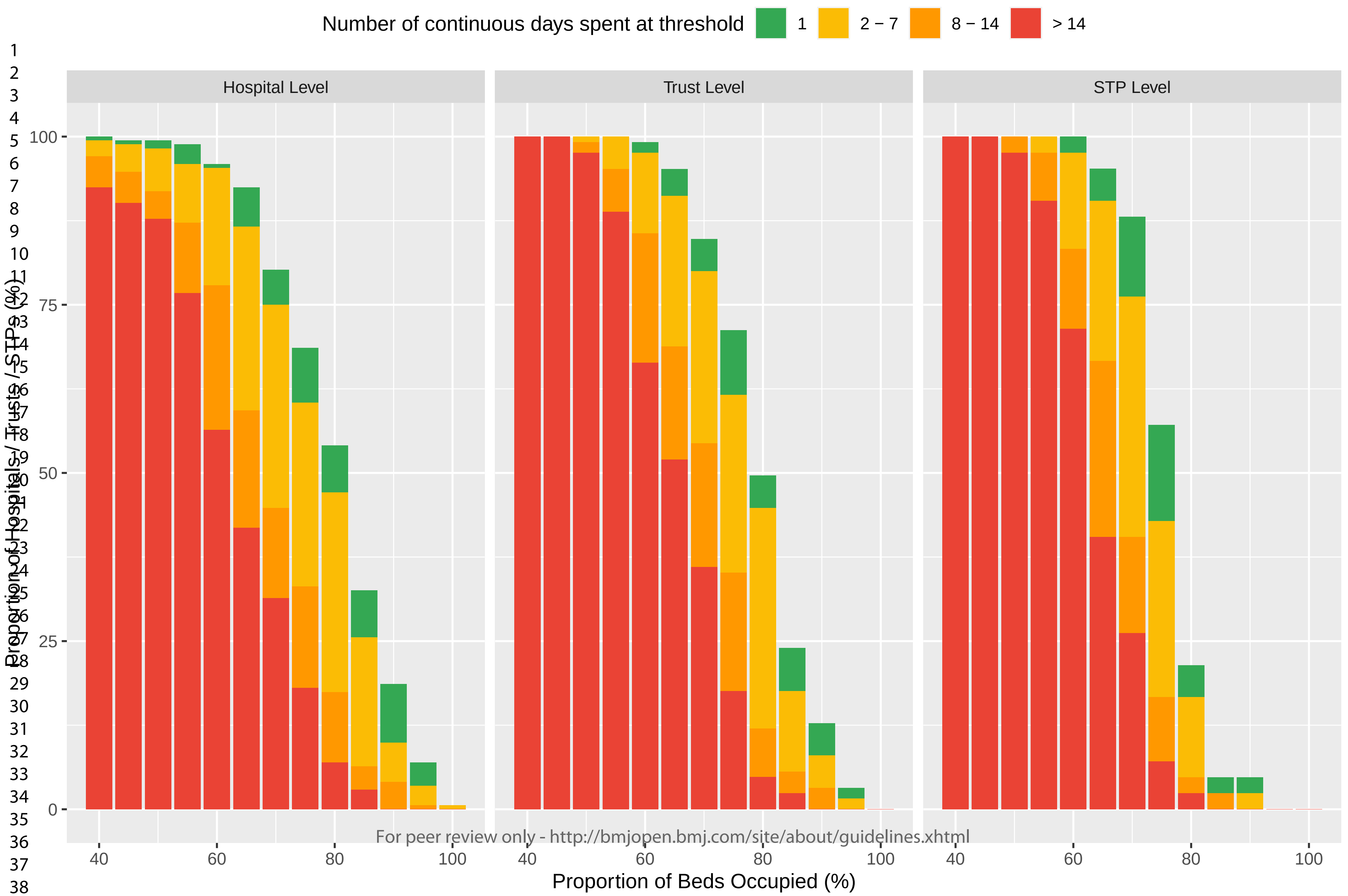

For peer review only - http://bmjopen.bmj.com/site/about/guidelines.xhtml

SFigure 8: Mechanical Ventilator Beds (Top) & General and Acute (Bottom) Occupancy (Based on Surge Capacities) Across England

Legend: SFigure 8A (Top) illustrates the proportion of STPs at different occupancy thresholds for surge mechanical ventilator bed capacity, across England. SFigure 8B (Bottom) illustrates the proportion of STPs at different occupancy thresholds for surge general and acute capacity, across England. The superimposed colours represent how long the trusts spent at each specific threshold.

For peer review only

SFigure 9: Ribbon Plots of STP Minimum and Maximum Occupancy

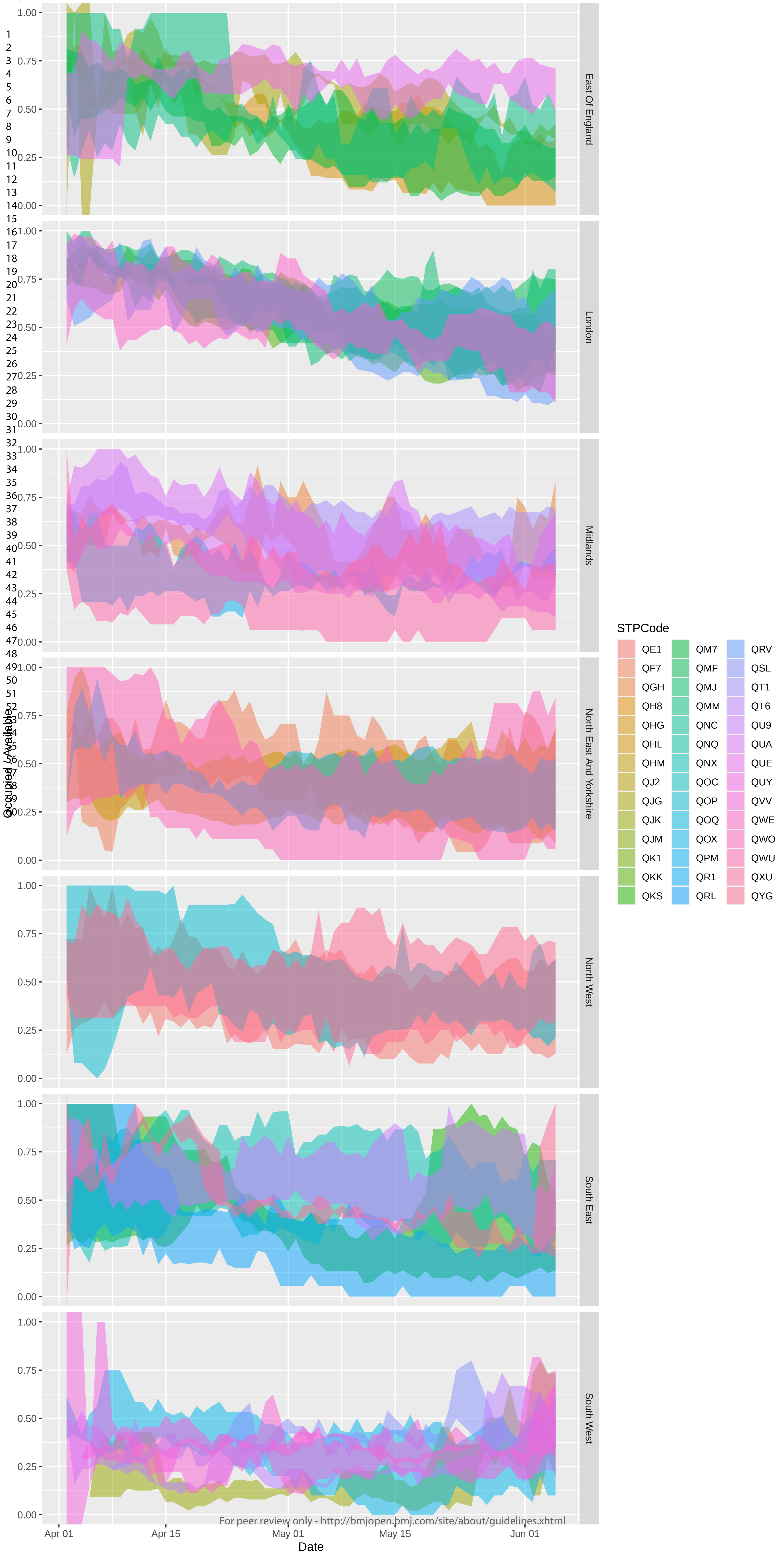

Supplementary Video 1

[See attached link for time-lapse of G&A bed capacity at the STP level across England]

Supplementary Video 2

[See attached link for time-lapse of mechanical ventilator bed capacity at the STP level across England]

Supplementary References

35 King's F. Sustainability and transformation plans (STPs) explained. 2017. https://www.kingsfund.org.uk/topics/integrated-care/sustainability-transformation-plans-explained.

36 Public Health England. COVID-19: investigation and initial clinical management of possible cases. 2020; published online Jan 10. https://www.gov.uk/government/publications/wuhan-novel-coronavirus-initial-investigation-of-possible-cases/investigation-and-initial-clinical-management-of-possible-cases-of-wuhan-novel-coronavirus-wn-cov-infection (accessed June 23, 2020).

37 Statistics. Statistics » Bed Availability and Occupancy Data – Overnight. https://www.england.nhs.uk/statistics/statistical-work-areas/bed-availability-and-occupancy/bed-data-overnight/. (accessed June 23, 2020).

38 Statistics. Statistics » Critical Care Bed Capacity and Urgent Operations Cancelled. https://www.england.nhs.uk/statistics/statistical-work-areas/critical-care-capacity (accessed June 23, 2020).

39 England NHS. NHS England » NHS steps up coronavirus fight with two more Nightingale Hospitals. https://www.england.nhs.uk/2020/04/nhs-steps-up-coronavirus-fight-with-two-more-nightingale-hospitals/ (accessed June 23, 2020).

40 England NHS. NHS England » NHS to build more Nightingale Hospitals, as London set for opening. https://www.england.nhs.uk/2020/04/nhs-to-build-more-nightingale-hospitals-as-london-set-for-opening/ (accessed June 23, 2020).

For peer review only

STROBE Statement—checklist of items that should be included in reports of observational studies

	Item No	Recommendation	Page No
**Title and abstract**	1	(*a*) Indicate the study's design with a commonly used term in the title or the abstract	1
		(*b*) Provide in the abstract an informative and balanced summary of what was done and what was found	2
**Introduction**			
Background/rationale	2	Explain the scientific background and rationale for the investigation being reported	4
Objectives	3	State specific objectives, including any prespecified hypotheses	4
**Methods**			
Study design	4	Present key elements of study design early in the paper	5
Setting	5	Describe the setting, locations, and relevant dates, including periods of recruitment, exposure, follow-up, and data collection	5
Participants	6	(*a*) *Cohort study*—Give the eligibility criteria, and the sources and methods of selection of participants. Describe methods of follow-up	5-6
		(*b*) *Cohort study*—For matched studies, give matching criteria and number of exposed and unexposed	NA
Variables	7	Clearly define all outcomes, exposures, predictors, potential confounders, and effect modifiers. Give diagnostic criteria, if applicable	6
Data sources/ measurement	8*	For each variable of interest, give sources of data and details of methods of assessment (measurement). Describe comparability of assessment methods if there is more than one group	*5-6*
Bias	9	Describe any efforts to address potential sources of bias	Supplementary material; quality control
Study size	10	Explain how the study size was arrived at	5
Quantitative variables	11	Explain how quantitative variables were handled in the analyses. If applicable, describe which groupings were chosen and why	5 & Supplementary material; quality control
Statistical methods	12	(*a*) Describe all statistical methods, including those used to control for confounding	6
		(*b*) Describe any methods used to examine subgroups and interactions	6 & Supplementary material; statistical analysis
		(*c*) Explain how missing data were addressed	Supplementary material; quality control
		(*d*) *Cohort study*—If applicable, explain how loss to follow-up was addressed	NA
		(*e*) Describe any sensitivity analyses	6

For peer review only - http://bmjopen.bmj.com/site/about/guidelines.xhtml

**Results**			
Participants	13*	(a) Report numbers of individuals at each stage of study—eg numbers potentially eligible, examined for eligibility, confirmed eligible, included in the study, completing follow-up, and analysed	7-15
		(b) Give reasons for non-participation at each stage	7
		(c) Consider use of a flow diagram	NA
Descriptive data	14*	(a) Give characteristics of study participants (eg demographic, clinical, social) and information on exposures and potential confounders	Supplementary table 3
		(b) Indicate number of participants with missing data for each variable of interest	Supplementary material; quality control
		(c) *Cohort study*—Summarise follow-up time (eg, average and total amount)	7-8
Outcome data	15*	*Cohort study*—Report numbers of outcome events or summary measures over time	*7-8*
Main results	16	(*a*) Give unadjusted estimates and, if applicable, confounder-adjusted estimates and their precision (eg, 95% confidence interval). Make clear which confounders were adjusted for and why they were included	NA
		(*b*) Report category boundaries when continuous variables were categorized	NA
		(*c*) If relevant, consider translating estimates of relative risk into absolute risk for a meaningful time period	NA
Other analyses	17	Report other analyses done—eg analyses of subgroups and interactions, and sensitivity analyses	Supplementary material; Critical care
**Discussion**			
Key results	18	Summarise key results with reference to study objectives	9
Limitations	19	Discuss limitations of the study, taking into account sources of potential bias or imprecision. Discuss both direction and magnitude of any potential bias	9-10
Interpretation	20	Give a cautious overall interpretation of results considering objectives, limitations, multiplicity of analyses, results from similar studies, and other relevant evidence	10-11
Generalisability	21	Discuss the generalisability (external validity) of the study results	11
**Other information**			
Funding	22	Give the source of funding and the role of the funders for the present study and, if applicable, for the original study on which the present article is based	No Funding

For peer review only

For peer review only - http://bmjopen.bmj.com/site/about/guidelines.xhtml

A descriptive analysis of hospital bed capacity and usage across secondary healthcare providers in England during the first wave of the COVID-19 Pandemic

Journal:	*BMJ Open*
Manuscript ID	bmjopen-2020-042945.R1
Article Type:	Original research
Date Submitted by the Author:	07-Nov-2020
Complete List of Authors:	Mateen, Bilal Akhter; The Alan Turing Institute,<br>Wilde, Harrison; University of Warwick<br>Dennis, John; University of Exeter, The Institute of Biomedical & Clinical Science<br>Duncan, Andrew; Imperial College London<br>Thomas, Nick; University of Exeter, The Institute of Biomedical & Clinical Science<br>McGovern, Andrew; University of Exeter, The Institute of Biomedical & Clinical Science<br>Denaxas, S; UCL<br>Keeling, Matt; University of Warwick,<br>Vollmer, Sebastian; University of Warwick
**Primary Subject Heading**:	Health policy
Secondary Subject Heading:	Infectious diseases, Intensive care, Medical management, Public health, Health policy
Keywords:	COVID-19, INTENSIVE & CRITICAL CARE, Public health < INFECTIOUS DISEASES, Health policy < HEALTH SERVICES ADMINISTRATION & MANAGEMENT

Note: The following files were submitted by the author for peer review, but cannot be converted to PDF. You must view these files (e.g. movies) online.

G&A Time Lapse.mp4
Vent Time Lapse.mp4

I, the Submitting Author has the right to grant and does grant on behalf of all authors of the Work (as defined in the below author licence), an exclusive licence and/or a non-exclusive licence for contributions from authors who are: i) UK Crown employees; ii) where BMJ has agreed a CC-BY licence shall apply, and/or iii) in accordance with the terms applicable for US Federal Government officers or employees acting as part of their official duties; on a worldwide, perpetual, irrevocable, royalty-free basis to BMJ Publishing Group Ltd ("BMJ") its licensees and where the relevant Journal is co-owned by BMJ to the co-owners of the Journal, to publish the Work in this journal and any other BMJ products and to exploit all rights, as set out in our licence.

The Submitting Author accepts and understands that any supply made under these terms is made by BMJ to the Submitting Author unless you are acting as an employee on behalf of your employer or a postgraduate student of an affiliated institution which is paying any applicable article publishing charge ("APC") for Open Access articles. Where the Submitting Author wishes to make the Work available on an Open Access basis (and intends to pay the relevant APC), the terms of reuse of such Open Access shall be governed by a Creative Commons licence – details of these licences and which Creative Commons licence will apply to this Work are set out in our licence referred to above.

Other than as permitted in any relevant BMJ Author's Self Archiving Policies, I confirm this Work has not been accepted for publication elsewhere, is not being considered for publication elsewhere and does not duplicate material already published. I confirm all authors consent to publication of this Work and authorise the granting of this licence.

For peer review only

A descriptive analysis of hospital bed capacity and usage across secondary healthcare providers in England during the first wave of the COVID-19 Pandemic

Bilal A. Mateen (MBBS)[1,2,3]*, Harrison Wilde (BSc)[4], John M. Dennis (PhD)[5], Andrew Duncan (PhD)[1,6], Nicholas J. Thomas (MRCP)[5,7] , Andrew P. McGovern (MD Res)[5,7], Spiros Denaxas (PhD)[1,8,9], Matt J Keeling (PhD)[10], Sebastian J. Vollmer (PhD)[1,4]

1 The Alan Turing Institute. Address: British Library, 96 Euston Road, London, NW1 2DB, UK
2 University of Warwick Medical School, Social Science and Systems in Health Unit. Address: Medical School Building, University of Warwick, Coventry, CV4 7AL, UK
3 Kings College Hospital NHS Foundation Trust. Address: Hambleden Wing, Kings College Hospital, Denmark Hill, London, SE5 9RS, UK
4 University of Warwick, Department of Statistics. Address: Department of Statistics, University of Warwick, Coventry, CV4 7AL, UK
5 University of Exeter Medical School. Address: Institute of Biomedical & Clinical Science, RILD Building, Royal Devon & Exeter Hospital, Barrack Road, Exeter EX2 5DW, UK
6 Imperial College London, Faculty of Natural Sciences. Address: Huxley Building, London, SW7 2AZ, UK
7 Royal Devon and Exeter NHS Foundation Trust, Diabetes and Endocrinology. Address: Exeter, Barrack Road, EX2 5DW
8 University College London, Institute of Health Informatics. Address: University College London, 222 Euston Rd, London NW1 2DA, UK
9 Health Data Research UK. Address: Gibbs Building, 215 Euston Road, London, NW1 2BE, UK
10 The Zeeman Institute for Systems Biology & Infectious Disease Epidemiology Research. Address: School of Life Sciences and Mathematics Institute, University of Warwick, Coventry, CV4 7AL, United Kingdom.

Author for Correspondence (*)
Dr. Bilal A. Mateen
Social Science and Systems in Health Unit
Warwick Medical School, University of Warwick
Coventry, CV4 7AL, UK
Email: bilal.mateen@nhs.net
Tel: +44 (0)24 7657 4880

Word Count: 3,899

For peer review only

Abstract

Objectives: In this study, we describe the pattern of bed occupancy across England during the peak of first wave of the COVID-19 pandemic.

Design: Descriptive survey

Setting: All non-specialist secondary care providers in England, from March 27th to 5th June 2020)

Participants: Acute (non-specialist) Trusts with a type 1 (i.e. 24 hours/day, consultant-led) accident and emergency department (n = 125), Nightingale (Field) Hospitals (n = 7), and independent sector secondary care providers (n = 195).

Main Outcome Measures: Two thresholds for 'safe occupancy' were utilized, 85% as per Royal College of Emergency Medicine and 92% as per NHS Improvement.

Results: At peak availability, there were 2711 additional beds compatible with mechanical ventilation across England, reflecting a 53% increase in capacity, and occupancy never exceeded 62%. A consequence of the repurposing of beds meant that at the trough, there were 8.7% (8,508) fewer general and acute (G&A) beds across England, but occupancy never exceeded 72%. The closest to (surge) capacity that any trust in England reached was 99.8% for general and acute beds. For beds compatible with mechanical ventilation there were 326 trust-days (3.7%) spent above 85% of surge capacity, and 154 trust-days (1.8%) spent above 92%. 23 trusts spent a cumulative 81 days at 100% saturation of their surge ventilator bed capacity (median number of days per trust = 1 [range: 1 to 17]). However, only 3 STPs (aggregates of geographically co-located trusts) reached 100% saturation of their mechanical ventilation beds.

Conclusions: Throughout the first wave of the pandemic, an adequate supply of all bed-types existed at a national level. Due to an unequal distribution of bed utilization, many trusts spent a significant period operating above 'safe-occupancy' thresholds, despite substantial capacity in geographically co-located trusts; a key operational issue to address in preparing for a potential second wave.

For peer review only

Article Summary

Strengths and Limitations of this Study

- The use of an administrative data that is collected by the statutory regulator as part of its legal mandate resulted in minimal missing information.
- Results are presented in the context of several geographic units of healthcare provision (i.e. hospital/site, trust, and STP-level), thus providing a much richer understanding of resource utilization that is less prone to the diluent effects of higher level geographies.
- The data represents a daily snapshot and therefore is unable to capture the nuances of the hospital throughput; in essence, both under and over-reporting of occupancy is possible using this method.
- The use of the occupancy thresholds reflects a limitation of our analysis, in that a proxy for adverse outcomes had to be utilized given that the necessary information was not readily available to directly explore the relationship between occupancy and patient-level outcome.
- The results of this study may not be generalizable to other countries given that it is specific to the UK National Health System infrastructure.

For peer review only

Introduction

The ability of hospitals to cope with large influxes of patients, either due to a pandemic illness or seasonal increases in respiratory disease exacerbations is in part dictated by the availability of beds.[1] Since 1987, when formal reporting of the number of hospital beds began in the UK, there has been a sustained decline in the number of available beds across the NHS.[2] In recent years, this issue has garnered more attention due to the annual 'winter bed crisis',[3,4] where the end of the calendar year heralds a surge in emergency admissions often resulting in hospitals operating well above quality and operational performance tipping points, i.e. 85% or 92% total bed occupancy.[5-7] The saturation of hospital beds is not only problematic through it impact on the ability of the workforce to deliver high-quality care,[8] but additionally the bottle-necking of the emergency care workflow has been shown to contribute to suboptimal outcomes for patients,[9] including increased numbers of healthcare-acquired infections,[10] and increased mortality.[11-13]

These concerns about the NHS' ability to cope with large influxes of patients took on a new level of significance in early 2020, when the World Health Organization (WHO) formally declared COVID-19 a pandemic illness, due to its virulence, and the magnitude of the disease's impact globally.[14] As early reports from China were published, it became apparent that a relatively large proportion of individuals who contracted COVID-19 required admission to hospital,[15] for example due to: new oxygen requirements, sepsis, acute respiratory distress syndrome (ARDS), and even multi-organ dysfunction (MODS). Forecasts of the potential number of people requiring hospital admission and mechanical ventilation across the UK suggested that the baseline capacity of the NHS would be insufficient.[16] In an effort to ensure sufficient capacity the British government instituted a series of policies, including facilitating the discharge of individuals who had been delayed due to non-medical reasons in an effort to unlock capacity,[17] cancelling all non-urgent clinical work, opening large field hospitals (i.e. the Nightingale hospitals),[18] and increasing mechanical ventilator availability for clinical areas repurposed to manage high care patients.[19]

The UK has started making significant strides towards rolling back its non-pharmacological interventions including. reopening schools, and planning for the discontinuation of shielding for vulnerable people,[20] signaling an end to the first wave of the pandemic.[21] Following these changes, there is the potential for a second wave of infections in the coming months. Understanding regional differences in hospital capacity is fundamental

For peer review only

to informing the UK's response to a second wave, as well as for elucidating how to safely wind down repurposed surge capacity such as operating theatres to allow other much needed clinical activity to restart.[22] However, other than a few isolated news reports of hospitals exceeding their ventilator capacity,[23] it is unclear how well the NHS as a whole managed to respond to the additional demand for beds over recent months. In this study, we sought to describe the pattern of bed occupancy in hospitals across England during the first wave of the COVID-19 pandemic.

For peer review only

Methods

Primary Data Source

Data were accessed from the daily situation reports ('SitReps', covering the previous 24 hours) provided to the Scientific Pandemic Influence Group on Modelling (SPI-M) by NHS England on behalf of all secondary care providers. All NHS acute care providers, independent sector care providers, and field hospitals in England submitting information to the daily situation reports were eligible for inclusion.

Study population

The data is presented in the context of several different units of secondary care provision: hospitals/sites, trusts, sustainability and transformation partnerships (STPs; aggregates of geographically co-located trusts), regions, and the whole of England (i.e. national), where each is an aggregate of the preceding unit (the structure of UK care providers is explained in the supplementary material).

Inclusion and Exclusion Criteria

Exclusions were applied at the trust level for NHS-specific care providers. Exclusion criteria were as follows: acute specialist trusts: women and/or children (n = 4), neurology & ophthalmology (n = 2), heart & lung (n = 3), orthopedic, burns & plastics (n = 4), cancer (n = 3). The remaining care providers were grouped into three categories and analyzed separately: 1) Acute (non-specialist) Trusts with a type 1 (i.e. 24 hours/day, consultant-led) accident and emergency department (n = 125); 2) Nightingale (Field) Hospitals (n = 7), and; 3) independent sector providers (n = 195).

Recruitment Period

Data was available from 27th March 2020 (the first available SitRep) to 5th June 2020 inclusive.

Recorded Information

The data specification comprised resource utilization and capacity-specific information, including the number of beds at each trust, stratified by several factors of interest, including acuity and COVID-19 ascertainment (further defined in supplementary material). Notably, beds were only recorded as being available if they were 'funded' (i.e. there was adequate staffing and resources for the bed to be occupied), so as to prevent counting of beds that could not accommodate a new patient. Bed acuity was organized into: general and acute (G&A), beds compatible

For peer review only

with non-mechanical ventilation, and beds compatible with mechanical ventilation. Occupancy is calculated based on the status of each bed at 08:00 each day, and then later separated by the proportion that had a positive COVID-19 test; there was no available information on the temporal relationship between admission and a positive test, and thus this data reflects some combination of community-acquired and nosocomial COVID-19.

Reporting fields changed on the 27th of April 2020, with several additional columns being added, which included specific fields for level 2 (HDU) and level 3 (ITU) beds. The impact of these changes is detailed in the supplementary material. However, one crucial outcome was that it became apparent the definition of critical care beds utilized prior to 27th April 2020 was not consistent with prior reporting practices of only including level 2 (HDU) and level 3 (ITU) beds,[24] as the newly reported values did not equal the simultaneously reported critical care values. As such, any results pertaining to critical care, HDU and ITU are reported separately in the supplementary material.

NHS England reports trust-level data, whereas we additionally attempted to disaggregate this information into the individual hospitals that the trusts comprise. Not all of the trusts were amenable to disaggregation from the trust-level data into independently reported sites in the available extracts, resulting in a final sample of 173 unique hospital sites, comprising 91.7% of the total number of ventilated beds and 81.4% of the general and acute beds when compared to trust level. The change in data reporting introduced on the 27th of April 2020 also resulted in variation in information capture; for data prior to the 27th of April, the results available reflect 89.6% of all mechanical ventilator beds and 86.9% of general and acute beds, where data from the 27th onwards the results reflect 93.0% of all mechanical ventilator beds but 77.0% of general and acute beds.

Outcome

The primary outcomes of interest were bed availability, and bed occupancy by patients with and without COVID-19, for each level of secondary care provision, i.e. hospital, trust, sustainability and transformation partnership (STP; aggregates of geographically co-located trusts). Different 'safe occupancy' thresholds were used to interpret the results; 85% as per the Royal College of Emergency Medicine, and 92% as per NHS Improvement. We also compared occupancy against baseline bed occupancy (see supplementary material for definitions), and 100% of surge capacity.

For peer review only

Statistical Analysis

We generated and reported descriptive summaries (e.g. medians, ranges, counts, proportions) of the data. We reported absolute numbers for hospital, trusts and STPs attaining specific occupancy thresholds. In light of the discordant critical care and HDU/ITU values, this analysis was handled and reported separately (see supplementary material). To capture the temporal aspect of the information available, the number of hospital-days, trust-days, and STP-days above hospital baseline capacity and surge capacities of 85%, 92% and 100% is also reported. Full details on the quality control procedures are reported in the supplementary material (SFigures 1 & SFigure 2). Details on aggregation and disaggregation of geographic information are provided in STable 1 & STable 2. Analysis were carried out in R,[25] ggplot2 package.[26] Maps were acquired from the UK's Office for National Statistics Open Geography Portal.[27]

Patient and Public Involvement

No patients were involved in the design of the study, interpretation of the results, or drafting of this manuscript.

For peer review only

Results

National Mobilization

During the first wave of the pandemic, the NHS repurposed general/acute beds into those suitable for higher acuity patients (i.e. HDU/ITU), and patients requiring mechanical ventilation. Available ventilated bed capacity peaked at an additional 2711 beds, a 53% increase from a baseline of 4123 beds. Ventilated beds occupancy never exceeded 62% of this capacity at a national level (Figure 1), and the proportion of occupied beds which contained patients with COVID-19 fluctuated between 30.4% and 76.0% over the course of the first wave, however there were notable regional differences in COVID-19 specific demand (Figure 2 & SFigure 3). Similar patterns were observed in critical care/HDU and ITU beds (SFigure 4). A consequence of the repurposing of beds for higher acuity patients there was a 8.7% reduction (n= 8,508) of general and acute beds from a baseline of 97,293 beds. There was a large reduction of the number of beds occupied by patients without COVID-19; 53,136 fewer beds were occupied (58.8% reduction) at the nidus compared to average occupancy from January to March 2020. Total bed occupancy never exceeded 72% nationally (Figure 1). Note: Data was relatively complete over the observation period (27th March to 5th June 2020), with no unavailable records for COVID-specific occupancy across G&A and mechanical ventilation compatible beds, and less than 10% for non-COVID/unoccupied beds (see supplementary material).

Occupancy relative to Baseline Capacity

Out of the 125 trusts (aggregates of hospitals), 3 trusts (2.4%) at some point during the first wave were operating above their baseline bed availability for general and acute beds (124 trust-days [1.4% of the total 8738 days at risk]; median number of days per trust = 36 days [range: 30 to 58]; SFigure 5). For beds compatible with mechanical ventilation, 87 trusts (69.6%) at some point during the first wave were operating above their baseline bed availability (2456 trust-days [28.1% of total at risk]; median number of days per trust = 24 days [range: 1 to 61]; SFigure 6). Similar results to that of mechanical ventilation compatible beds were seen for critical care / HDU and ITU bed occupancy (see supplementary material, SFigure 7 & SFigure 8).

Occupancy relative to Surge Capacity

Table 1 summarizes the number of hospitals, trusts and STPs operating above each of the thresholds for ‘safe occupancy’, and details the duration (i.e. median number of days) that each spent above the designated thresholds.

For peer review only

Hospital-level Occupancy

Of the total 11,851 English hospital-days at risk over the study period, 494 hospital-days (4.17% of total days at risk) were at or above 85% of bed (surge) capacity, 110 hospital-days (0.92%) were at or above 92% of bed (surge) capacity, and only 10 were spent at 100% of surge capacity (Figure 3). Similarly, for beds compatible with mechanical ventilation there were 586 hospital-days (4.94%) spent above 85% of surge capacity, 320 hospital-days (2.70%) were spent above 92%, and 226 hospital-days (1.9%) were spent at 100% occupancy (see Figure 4). Summaries of the size and geographic locations of hospitals stratified by saturation are in STable 3.

Trust-level Bed Occupancy

Over the study period, there were 287 trust-days (3.3% of total days at risk) where general and acute bed occupancy exceeded 85% occupancy of surge capacity, and 57 trust-days (0.7%) were at or above 92% of bed (surge) capacity. The closest to capacity any trust in England reached was 99.8% for general and acute beds. However, for beds compatible with mechanical ventilation there were 326 trust-days (3.7%) spent above 85% of surge capacity, and 154 trust-days (1.8%) spent above 92%. 23 trusts reached 100% saturation of their mechanical ventilator bed capacity (Figure 5 & SFigure 9).

Table 1: The Number of Hospital/Trusts/STPs at each Occupancy Threshold for Different Bed Types

Bed Type	Organizational Unit	Occupancy Threshold					
		> 85%		> 92%		= 100%	
		Number (%)	Median number of days at or above threshold (range)	Number (%)	Median number of days at or above threshold (range)	Number (%)	Median number of days at or above threshold (range)
General & Acute	Hospital/Site (n = 173)	56 (32.4%)	6 (1 to 45]	19 (11.0%)	3 (1 to 19)	1 (0.6%)	10 days
	Trust (n = 125)	30 (24.0%)	5 (1 to 46)	14 (11.2%)	3 (1 to 13)	0 (0.0%)	-
	Sustainability and Transformation Partnership (n = 42)	2 (4.8%)	10 (3 to 17)	2 (4.8%)	1 (1 to 1)	0 (0.0%)	-
Mechanical Ventilation	Hospital/Site (n = 173)	91 (52.6%)	4 (1 to 48)	72 (41.6%)	3 (1 to 48)	52 (30.0%)	2 (1 to 48)
	Trust (n = 125)	58 (46.4%)	3 (1 to 27)	40 (32.0%)	2 (1 to 17)	23 (18.4%)	1 (1 to 17)
	Sustainability and Transformation Partnership (n = 42)	10 (23.8%)	2 (1 to 11)	5 (10.4%)	1 (1 to 6)	3 (7.1%)	1 (1 to 2)

Sustainability and Transformation Partnerships (STP) level Bed Occupancy

Across the 42 STPs (aggregates of geographically co-located trusts), there were 20 STP-days (0.7% of total days at risk) where general and acute bed occupancy exceeded 85% occupancy of surge capacity. The highest any STP reached for G&A bed occupancy was 92.7%. For beds compatible with mechanical ventilation, there were 35 STP-days (1.2%) where occupancy exceeded 85% occupancy of surge capacity, 11 STP-days (0.4%) in excess of 92% occupancy, and 4 STP-days (0.1%) at full occupancy (all of which were for STPs outside London: 1) Somerset, 2) Suffolk and North East Essex, and 3) Shropshire, Telford and Wrekin; SFigure 10). Figure 6 illustrates the number of STPs operating at each distinct occupancy threshold as a proportion of baseline and actual surge capacity. The full time-lapse for G&A (Supplementary Video 1) & ventilator bed (Supplementary Video 2) occupancy over the period of interest can be found in the supplementary material. A similar pattern was seen in the context of critical care / HDU & ITU beds across the STPs (SFigure 7 & SFigure 8).

Field (Nightingale) Hospital Occupancy

Of the reported bed capacity achievable through opening the Nightingale hospitals, at peak occupancy only 1.23% of the theoretical maximum were being utilized (Table 2). This equates to 618 bed days for patients with COVID-19 requiring mechanical ventilation, and 1483 bed days for all other types of intervention for patients with COVID-19 (i.e. oxygenation, non-invasive respiratory support, non-respiratory organ support, etc.).

Table 2: Field (Nightingale) Hospital Occupancy, and Capacity

Nightingale Hospital Location	Number of Occupied Beds at peak*	Maximum number of Operational Beds*	Maximum Theoretical Capacity (Beds)
London (Excel Centre)	66	112	4000
Manchester (Convention Centre)	47	72	1000
Birmingham (National Exhibition Centre)	0	0	2000
Bristol (University of West England)	NA	NA	1000
Washington (Centre of Excellence for Sustainable Advanced Manufacturing)	NA	NA	450
Harrogate (Convention Centre)	0	0	500
Exeter (Westpoint Arena)	NA	NA	200

**Several hospitals were formally opened, but never reported an occupied bed, as such they did not appear in the SitRep dataset (denoted by NA in the table). Those that were in the dataset but had no patients are denoted by a '0'.*

For peer review only

Independent Sector Care Providers

Variations in reporting meant that the number of providers reporting each day varied, median 181 providers (range: 172 to 187). At peak occupancy, no more than 134 independent sector beds were occupied with patients who were confirmed COVID-19 positive. With regards to patients without COVID-19, at peak occupancy there were 1350 people in independent sector beds, representing a peak saturation of 18.7% (based on the total number of beds reported during contractual negotiations). In summary, there were 3360 bed days for patients with confirmed COVID-19 accommodated by the independent sector (86 mechanical ventilator bed days, 104 non-invasive ventilation bed days, and 3170 other bed days), and 53937 bed days for patients without COVID-19 (2771 mechanical ventilator bed days, 2046 non-invasive ventilation bed days, and 49120 other bed days), between 2nd April and 5th June across England.

For peer review only

Discussion

This national study of hospital-level bed occupancy provides unique and timely insight into the impact of COVID-19 on bed-specific resource utilization across an entire country. Our analysis suggests that the response of the NHS and British government to COVID-19 was sufficient to alleviate early concerns regarding the number of mechanical ventilators and critical care beds at a national level, however local variation in demand (i.e. regional variation in COVID-19 prevalence) still meant that many trusts reached 100% capacity for both. Moreover, examining occupancy in the context of different organizational units (i.e. trust-level versus STP-level), suggests that the higher-order networks (i.e. STPs) were not efficiently utilized to off-load disproportionately impacted trusts, as it was theoretically possible to have 95.1% fewer trust days at 100% mechanical ventilator bed capacity assuming load was better distributed. On the other hand, despite a reduction in overall capacity, G&A bed occupancy-levels relatively infrequently reached 'unsafe' levels, even at the individual hospital-level. This in part may explain why the field hospitals and independent sector care provider beds were never substantially utilized. Only a very small fraction of the theoretical maximum field hospital bed capacity was operationalized (1.23%). Similarly, despite signing a 14 week block contract with all of the major independent sector care providers valued at £235 million,[28] these beds too remained largely unoccupied, with less than 24% of the theoretical maximum beds days for established ventilators (i.e. not including the 1012 theatre-specific mechanical ventilators) having been used.

Context

Initial estimates suggested that an additional 30,000 mechanical ventilators would be necessary to accommodate the impact of the COVID-19 pandemic. These estimates were later updated to just 18,000 mechanical ventilators, from an estimated baseline of 8,000 across the UK.[29] It is difficult to determine the accuracy of these projections, as they were made in the absence of the impact of non-pharmacological interventions. However, the results of our study suggests that, at the population level, UK-based models of ventilator and bed resource utilization which integrated the impact of non-pharmacological interventions were actually remarkably accurate.[16,30] Arguably the most influential modelling study was that of the Imperial MRC group, wherein the authors clearly illustrate that with full 'lockdown' (i.e. the suite of non-pharmacological interventions that were eventually instituted), that critical care bed capacity would not be overwhelmed.[16] The nuance that this modelling study lacked was that it failed to explicitly incorporate the impact of unequal distribution of burden,

which manifested in our data as specific hospitals and trusts reaching full occupancy, despite the fact that at the national-level there was a substantial number of unoccupied beds.

This retrospective analysis also highlights some of the early incorrect assumptions made about the UK's baseline resource availability. For example, in contrast to ministerial statements suggesting that there were approximately 8,000 ventilators in the UK prior the pandemic,[29] our results identified only 4123 operational beds compatible with mechanical ventilation on the first day of reporting in England. Even after acknowledging that our value does not account for the devolved nations (Wales, Scotland, and Northern Ireland), it is unlikely that the initial figures reported by members of parliament truly reflected operational capacity, as that would suggest only 50% of such equipment was in England, despite it representing 84% of the UK population. Interestingly, the absolute increase in ventilator numbers due to the government incentives (e.g. the UK's Industrial Ventilator Challenge) is much more similar to our reported results.

Strengths and Limitations

There are several strengths to this study. For example, the use of an administrative (i.e. 'SitRep') data that is a statutory collection by NHS England, via a well-established reporting mechanism that has been exploited for research,[31] confers robustness to the data. One example of how this robustness manifested is, unlike other attempts to collect data at a national level to inform the COVID-19 response plan in the UK,[32] the degree of missingness in the data utilized in this study was minimal (see supplementary material). Moreover, in light of the unique access to the raw 'SitRep' data, we have been able to present our results not only at the trust-level, to which previous endeavors have been limited,[33] but rather have been able to present information at a much more granular layer (i.e. hospital/site-level) thus providing a much richer understanding of resource utilization that is less prone to the diluent effects of higher level geographies. Finally, a further strength of this study is the relative simplicity of the analysis; there are no complex statistical methods utilized as the descriptive summaries presented are sufficient to describe the experiences of nationalized (single-payer) health system in a high-income economy during the first wave of the COVID-19 pandemic.

Notably though, there are also several limitations to the dataset and our analysis. Principally we have no information on individual clinician and patient behaviour that will have inevitably influence these occupancy rates, and thus cannot comment on these factors. Secondly, there are limitations inherent to the SitRep data. In

For peer review only

particular, data were not available during February and early March during which some early 'bed mobilization' was likely carried out, and thus our observation period does not cover the entirety of the first wave (however, we believe it is unlikely that this undermines the major findings of this study). Moreover, changes introduced in 'SitRep' data collection half-way through the reporting period limited our ability to investigate critical care bed occupancy which was the third bed-specific potential concern identified by forecasting experts. The hospital-level results should also be interpreted with caution as it is an incomplete representation of the core trust-level information, and thus may not truly reflect the exact position of each organization; for example, the trust corresponding to the single site that achieved 100% G&A occupancy was never itself at 100% total occupancy. On a related note, the core weakness of the 'SitRep' data is that it presents data as a daily snapshot (at 08:00/8am), and therefore is unable to capture the nuances of the hospital throughput; in essence, both under and over-reporting of occupancy is possible using this method. As such, any marginal results where hospitals are only just over one of the 'safe occupancy-level' thresholds should be interpreted with caution as they could represent reporting artefacts. Moreover, the use of the occupancy thresholds reflects a limitation of our analysis, in that a proxy for adverse outcomes had to be utilized given that the necessary information was not readily available to directly explore the relationship between occupancy and patient-level outcome. Finally, the results of this study may not be generalizable to other countries given that it is specific to the UK National Health System infrastructure and reporting systems; for example, it is difficult to draw comparisons with other countries as UK specific factors such as reporting definitions are likely to mediate the hypothesized occupancy-mortality risk relationship, which will inevitably limit the ecological validity of these results in other geographical settings.

For peer review only

Implications for Policy Makers, and Clinicians

This study illustrates the potential for near real-time results reporting by which to determine the need for and effectiveness of government policies introduced to address resource utilization-specific issues as a consequence of the COVID-19 pandemic. For example, due to an unequal distribution of the resource utilization burden across England, many trusts spent a significant period of time operating above 'safe-occupancy' thresholds, despite the fact that in the vast majority of circumstances there was relief capacity in geographically co-located trusts (i.e. at the STP-level). Out of the 81 trust-days spent at 100% saturation of their mechanical ventilation beds (which pertains to 23 trusts reaching this threshold), on all but 5 days there was spare capacity at the corresponding STP level, which would have resulted in only 4 trusts reaching 100% saturation at any point (SFigure 11: STP min-

max occupancy plots). This reflects a key operational issue for policymakers to address in preparing for a potential second wave, and would have been identifiable if the SitRep data had been utilized for now-casting. Moreover, other policies for which these results may be relevant, include the creation of the Nightingale (field) hospitals, and independent sector network partnership. Our results suggest that the early investment and the creation of an operational field hospital and independent sector network may yield more overtly positive results in the winter, when G&A occupancy-levels regularly exceed 92%,[34] however, during the first wave of the pandemic they were under-utilized.

Conclusion

Using administrative data submitted by all secondary care organizations in England, we can conclude that at the national level there was an adequate supply of all bed-types throughout the first wave of the COVID-19 pandemic. However, the burden of need was not equally distributed, and thus in many cases local demand exceeded the supply of beds, especially where it concerned mechanical ventilation. Although several of the policies introduced by the government, both historical (i.e. STPs) and pandemic-specific (e.g. the independent sector block contract), could have potentially addressed this issue, there is evidence that these interventions were not optimally utilized. As such, we hope that this paper acts as exemplar for how routinely collected administrative data can be used to evaluate policy interventions, especially in the context of the COVID-19 pandemic, as well as highlighting the need for locally-relevant (in lieu of national or regional summaries), near-real-time information on service use for operational decision making.

For peer review only

Acknowledgements

BAM, SJV, and SD are supported by The Alan Turing Institute (EPSRC grant EP/N510129/). JMD is supported by an Independent Fellowship funded by Research England's Expanding Excellence in England (E3) fund. SJV is supported by the University of Warwick IAA funding. HW is supported by the Feuer International Scholarship in Artificial Intelligence. JMD, NJT, and APM are supported by the NIHR Exeter Clinical Research Facility. We thank NHS Improvement & NHS England for providing access to the SitRep data and Prof. Matt Keeling for establishing data access. Finally, we thank Dr. Bu'Hussain Hayee for sense-checking the final draft.

Contributors

Based on the CRediT Taxonomy, the authors of this study made contributions to this manuscript in the following ways: conceptualization (BAM & SJV); data curation (HW & SJV); methodology (HW, BAM & SJV); formal analysis (HW, BAM, JMD, SD, SJV); project administration & supervision (BAM, MK & SJV); visualization (HW, BAM, JMD, NJT, APM, AD, SJV); resources (SJV & MK); writing the original draft (BAM & HW); reviewing and editing the draft (all authors).

Declaration of interest

APM declares previous research funding from Eli Lilly and Company, Pfizer, and AstraZeneca. SJV declares funding from IQVIA. All other authors declare no competing interests.

Ethics & Governance

Data utilized in this study were made available through an agreement between the University of Warwick and the Scientific Pandemic Influence Group on Modelling (SPI-M), whom were acting on behalf of the British Government. The study was reviewed and approved by the Warwick BSREC (BSREC 120/19-20).

Data Access Statement

Trust-level data will eventually be published by NHS England as a freely accessible data resource, but outputs have been delayed by the COVID-19 pandemic. For expedited or more granular access, requests will need to be made directly to NHS England (contact via england.dailysitrep@nhs.net). All code for this study is available on request.

For peer review only

Guarantor Statement

The corresponding (BAM) and the senior author (SJV) had full access to all data, and attest to the integrity of the analysis. The decision to submit for publication was agreed by all authors, and BAM and SJV act as guarantors of the work as presented.

Role of the Funding Source

There was no direct funding for this study. No funder was involved in the study design, analysis, interpretation of data; in the writing of the report; or in the decision to submit the paper for publication.

Dissemination

We will work with the relevant governmental groups to ensure that the patient organizations are informed of these results in a timely manner.

For peer review only

References

1 NHS hospital bed numbers. The King's Fund. 2020; published online March 26. Available at: https://www.kingsfund.org.uk/publications/nhs-hospital-bed-numbers (accessed June 21, 2020).

2 Appleby J. The hospital bed: on its way out? *BMJ* 2013; **346**: f1563.

3 The NHS this winter: looking beneath the national view | The Health Foundation. The Health Foundation. Available at: https://www.health.org.uk/news-and-comment/blogs/the-nhs-this-winter-looking-beneath-the-national-view (accessed June 21, 2020).

4 British Medical Association. Bed occupancy in the NHS - Pressures - BMA. The British Medical Association is the trade union and professional body for doctors in the UK. 2020; published online Jan 20. Available at: https://www.bma.org.uk/advice-and-support/nhs-delivery-and-workforce/pressures/bed-occupancy-in-the-nhs (accessed June 21, 2020).

5 Bagust A, Place M, Posnett JW. Dynamics of bed use in accommodating emergency admissions: stochastic simulation model. *BMJ* 1999; **319**: 155–8.

6 Fixing Emergency Department Staffing, Systems & Support to deliver excellent patient centered care. The Royal College of Emergency Medicine. Available at: https://www.rcem.ac.uk/docs/Policy/RCEM%20Vision%202020.pdf.

7 Improvement NHS. NHS review of winter 2017/18: annex. Available at: https://improvement.nhs.uk/documents/3202/Winter_review_annex_2.pdf (accessed June 22, 2020).

8 Against the odds: experiences from the NHS front line. RCP London. 2017; published online Feb 28. Available at: https://www.rcplondon.ac.uk/projects/outputs/against-odds-experiences-nhs-front-line (accessed June 23, 2020).

9 The implications of high bed occupancy rates on readmission rates in England | The Health Foundation. The Health Foundation. Available at: https://www.health.org.uk/publications/journal-articles/the-implications-of-high-bed-occupancy-rates-on-readmission- (accessed June 22, 2020).

10 Great Britain: National Audit Office. National Audit Office (NAO) - Department of Health:

For peer review only

Emergency Admissions to Hospital: Managing the Demand - HC 739: Managing the Demand, Department of Health. The Stationery Office, 2013.

11 Cowan RM, Trzeciak S. Clinical review: Emergency department overcrowding and the potential impact on the critically ill. *Crit Care* 2005; **9**: 291–5.

12 Sprivulis PC, Da Silva J-A, Jacobs IG, Frazer ARL, Jelinek GA. The association between hospital overcrowding and mortality among patients admitted via Western Australian emergency departments. *Med J Aust* 2006; **184**: 208–12.

13 Emergency and acute medical care in over 16s: service delivery and organisation. NICE. Available at: https://www.nice.org.uk/guidance/ng94/evidence/39.bed-occupancy-pdf-172397464704.

14 World Health Organization. Coronavirus Disease (COVID-19) Situation Reports. Available at:https://www.who.int/emergencies/diseases/novel-coronavirus-2019/situation-reports (accessed May 21, 2020).

15 Zhou F, Yu T, Du R, *et al.* Clinical course and risk factors for mortality of adult inpatients with COVID-19 in Wuhan, China: a retrospective cohort study. *Lancet* 2020; **395**: 1054–62.

16 Ferguson NM, Laydon D, Nedjati-Gilani G, *et al.* Impact of non-pharmaceutical interventions (NPIs) to reduce COVID-19 mortality and healthcare demand. 2020. *DOI* 2020;**10**:77482.

17 Stevens S. NHS-E and NHS-I LETTER ON NEXT STEPS ON NHS RESPONSE TO COVID-19. NHS England. Available at: https://www.england.nhs.uk/coronavirus/wp-content/uploads/sites/52/2020/03/urgent-next-steps-on-nhs-response-to-covid-19-letter-simon-stevens.pdf.

18 NHS England. New NHS nightingale hospital to fight coronavirus. Available at: https://www.england.nhs.uk/2020/03/new-nhs-nightingale-hospital-to-fight-coronavirus/ (accessed June 21, 2020).

19 UK Government Ventilator Challenge. 2020; published online April 22. Available at: https://www.gov.uk/government/topical-events/coronavirus-uk-government-ventilator-challenge/about (accessed June 21, 2020).

20 Department of Health, Care S. Plans to ease guidance for over 2 million shielding. 2020; published

For peer review only

online June 22. Available at: https://www.gov.uk/government/news/plans-to-ease-guidance-for-over-2-million-shielding (accessed June 23, 2020).

21 Prime Minister's Office, 10 Downing Street. Six people can meet outside under new measures to ease lockdown. 2020; published online May 28. Available at: https://www.gov.uk/government/news/pm-six-people-can-meet-outside-under-new-measures-to-ease-lockdown (accessed June 21, 2020).

22 Shock to the system: COVID-19's long-term impact on the NHS | The Health Foundation. The Health Foundation. Available at: https://www.health.org.uk/news-and-comment/blogs/shock-to-the-system-covid-19s-long-term-impact-on-the-nhs (accessed June 23, 2020).

23 Coronavirus: 'Critical incident' declared at Northwick Park Hospital in London after running out of intensive care beds [Internet]. ITV News. 2020 [cited 24 June 2020]. Available from: https://www.itv.com/news/2020-03-20/northwick-park-hospital-london-coronavirus-covid-19/

24 Monthly Trust SitRep (MSitRep) Critical Care Bed Capacity and Urgent Operations Cancelled. NHS England. Available at: https://www.england.nhs.uk/statistics/wp-content/uploads/sites/2/2019/11/MSitRep-Guidance-v1.09.pdf (accessed June 22, 2020).

25 R Core Team. R: A language and environment for statistical computing. R Foundation for Statistical Computing. Vienna, Austria. 2011.

26 Wickham H. ggplot2: Elegant Graphics for Data Analysis. Springer-Verlag New York. 2016.

27 Sustainability and Transformation Partnerships (April 2020) Boundaries. Available at: https://geoportal.statistics.gov.uk/datasets/sustainability-and-transformation-partnerships-april-2020-boundaries-en-buc?geometry=-43.431%2C48.021%2C39.142%2C57.304 (accessed June 23, 2020).

28 Permain N. COVID-19: Partnership working with the Independent Sector Providers and the Independent Healthcare Providers Network (IHPN). NHS England. Available at: https://www.england.nhs.uk/coronavirus/wp-content/uploads/sites/52/2020/03/ihpn-partnership-letter-25-march-2020.pdf (accessed June 22, 2020).

29 Balogun B. Coronavirus: Ventilator availability in the UK. 2020; published online June 3. Available at:

For peer review only

https://commonslibrary.parliament.uk/research-briefings/cbp-8904/ (accessed June 23, 2020).

30 Deasy J, Rocheteau E, Kohler K, *et al.* Forecasting ultra-early intensive care strain from COVID-19 in England. *medRxiv* 2020. https://www.medrxiv.org/content/medrxiv/early/2020/03/23/2020.03.19.20039057.full.pdf.

31 Friebel R, Fisher R, Deeny SR, Gardner T, Molloy A, Steventon A. The implications of high bed occupancy rates on readmission rates in England: A longitudinal study. *Health Policy* 2019; **123**: 765–72.

32 Dennis JM, Mateen BA, Sonabend R, Thomas NJ, Patel KA, Hattersley AT, Denaxas S, McGovern AP, Vollmer SJ. Diabetes and COVID-19 Related Mortality in the Critical Care Setting: A Real-Time National Cohort Study in England. Available at SSRN 3615999. 2020 May 27.

33 The NHS this winter: looking beneath the national view | The Health Foundation. The Health Foundation. Available at: https://www.health.org.uk/news-and-comment/blogs/the-nhs-this-winter-looking-beneath-the-national-view (accessed June 23, 2020).

34 The NHS this winter: looking beneath the national view | The Health Foundation. The Health Foundation. Available at: https://www.health.org.uk/news-and-comment/blogs/the-nhs-this-winter-looking-beneath-the-national-view (accessed June 23, 2020).

For peer review only

Figure Legends

Figure 1: National and Regional Bed Occupancy

Legend: (Top) An epidemic curve showing the number of confirmed cases of COVID-19 across England. (Middle) Total capacity and occupancy status for general and acute (G&A) at the national level over the course of the first wave. (Bottom) Total capacity and occupancy status for bed compatible with mechanical ventilation at the national level.

Figure 2: Total bed occupancy in each of the 7 regions of England

Legend: (Top) COVID-19 specific occupancy in each of the 7 regions across England, for both general and acute (G&A; Left), as well as bed compatible with mechanical ventilation (Right). (Bottom) Total occupancy (COVID-19 positive and negative) in each of the 7 regions across England, for both general and acute (G&A; Left), as well as bed compatible with mechanical ventilation (Right). Note: the highly-saturated solid line represents a smoothened function of the raw data, whereas the less saturated solid line represents the underlying raw values. The former is based on the ggplot loess fit for trend lines, using local poly-regression curve fitting.

Figure 3: Hospital-level General & Acute Bed Occupancy (Based on Surge Capacities) Across England

Legend: The number of hospitals with general and acute bed occupancy in excess of the thresholds for 'safe and effective' functioning, i.e. 85% as defined by the Royal College of Emergency Medicine,[6] and 92% as defined by NHS Improvement and NHS England (green and yellow, respectively),[7] across England, from 26th March to June 5th. Note: all data was missing for the 29th of March and the 24th of May.

Figure 4: Hospital-level Ventilator Bed Occupancy (Based on Baseline Capacities) Across England

Legend: The number of hospitals with occupancy of mechanical ventilation beds in excess of the thresholds for 'safe and effective' functioning, i.e. 85% as defined by the Royal College of Emergency Medicine,[6] and 92% as defined by NHS Improvement and NHS England (green and yellow, respectively),[7] across England, from 1st April to June 5th. Note: all data was missing for the 24th of May.

Figure 5: Trust-Level Ventilator Bed Occupancy (Based on Surge Capacities) Across England

Legend: The number of trusts with occupancy of mechanical ventilation beds in excess of the thresholds for 'safe and effective' functioning, i.e. 85% as defined by the Royal College of Emergency Medicine,[6] and 92% as defined by NHS Improvement and NHS England (yellow and red, respectively),[7] across England, from March 26th to June 5th.

For peer review only

Note: all data was missing for the 29th of March and the 24th of May. Several hospitals reported values consistent with 100% occupancy (black).

Figure 6: Peak Sustainability and Transformation Partnership (STP) Bed Occupancy Across England

Legend: The date on which general and acute bed occupancy (Left) and mechanical ventilator beds (Right) peaked, based on surge capacities at the Sustainability and Transformation Partnership (STP) level, across England. The geo-temporal pattern of peak occupancy clearly demonstrates that there was always residual G&A capacity at the STP level, and that all regions across England experienced similar levels of saturation. However, saturation of mechanical ventilator beds differed substantially by location.

For peer review only

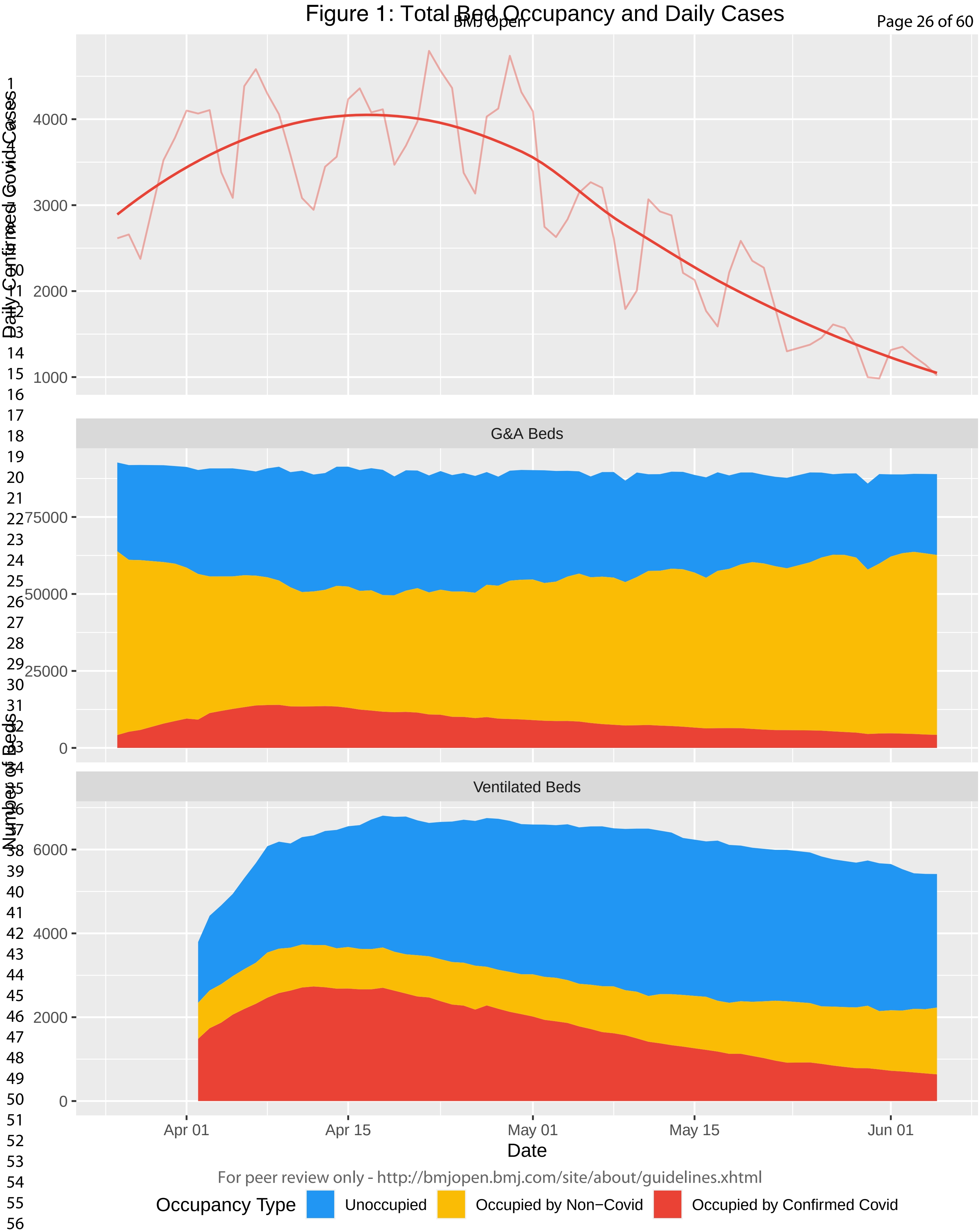
Figure 1: Total Bed Occupancy and Daily Cases
Daily Confirmed Covid Cases
4000
3000
2000
1000
G&A Beds
75000
50000
25000
0
Ventilated Beds
6000
4000
2000
0
Number of Beds
Apr 01
Apr 15
May 01
May 15
Jun 01
Date
Occupancy Type
Unoccupied
Occupied by Non−Covid
Occupied by Confirmed Covid

BMJ Open

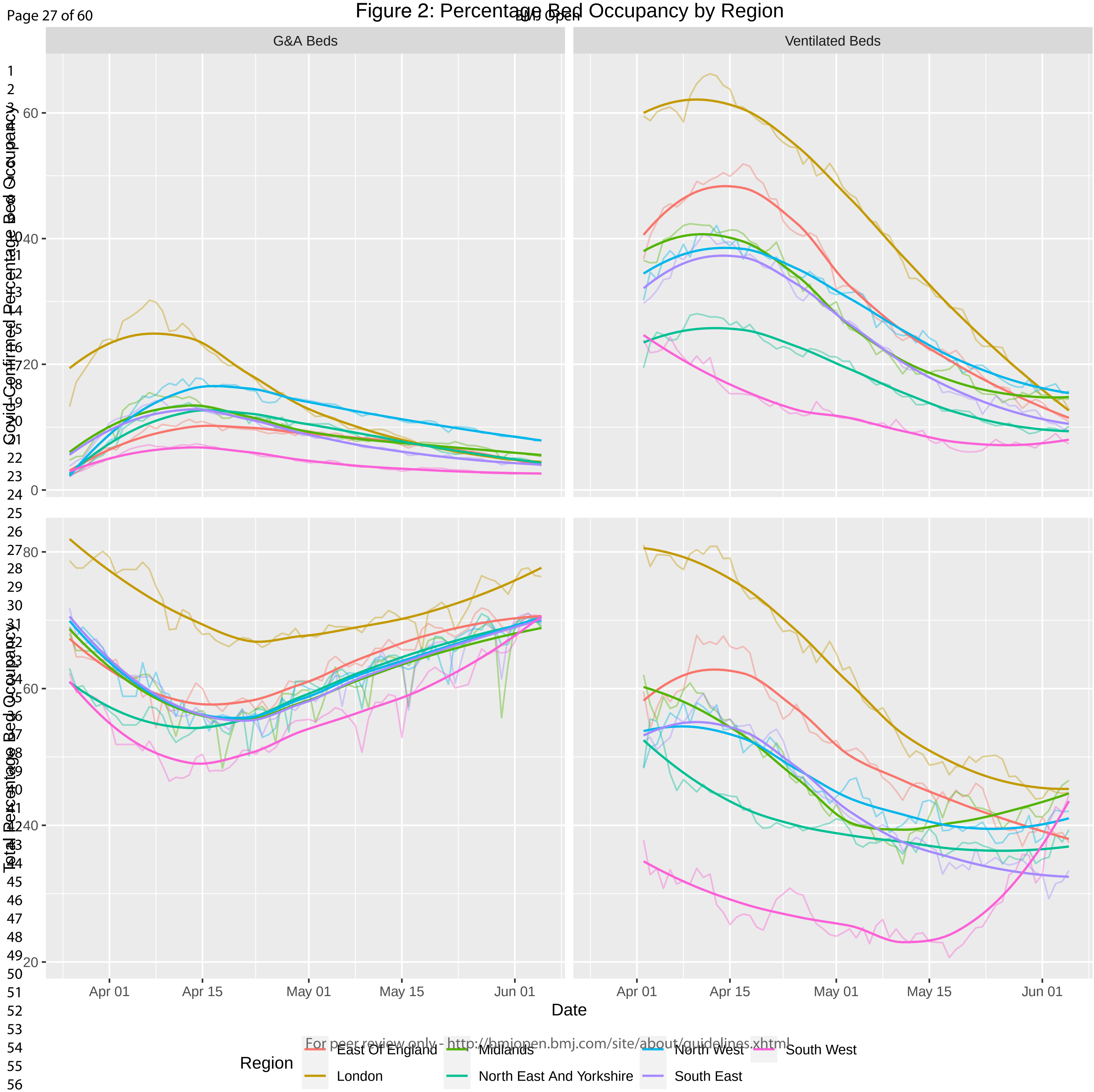

For peer review only - http://bmjopen.bmj.com/site/about/guidelines.xhtml

Figure 3: Number of Hospitals Operating Above Various G&A Bed Surge Capacity Thresholds

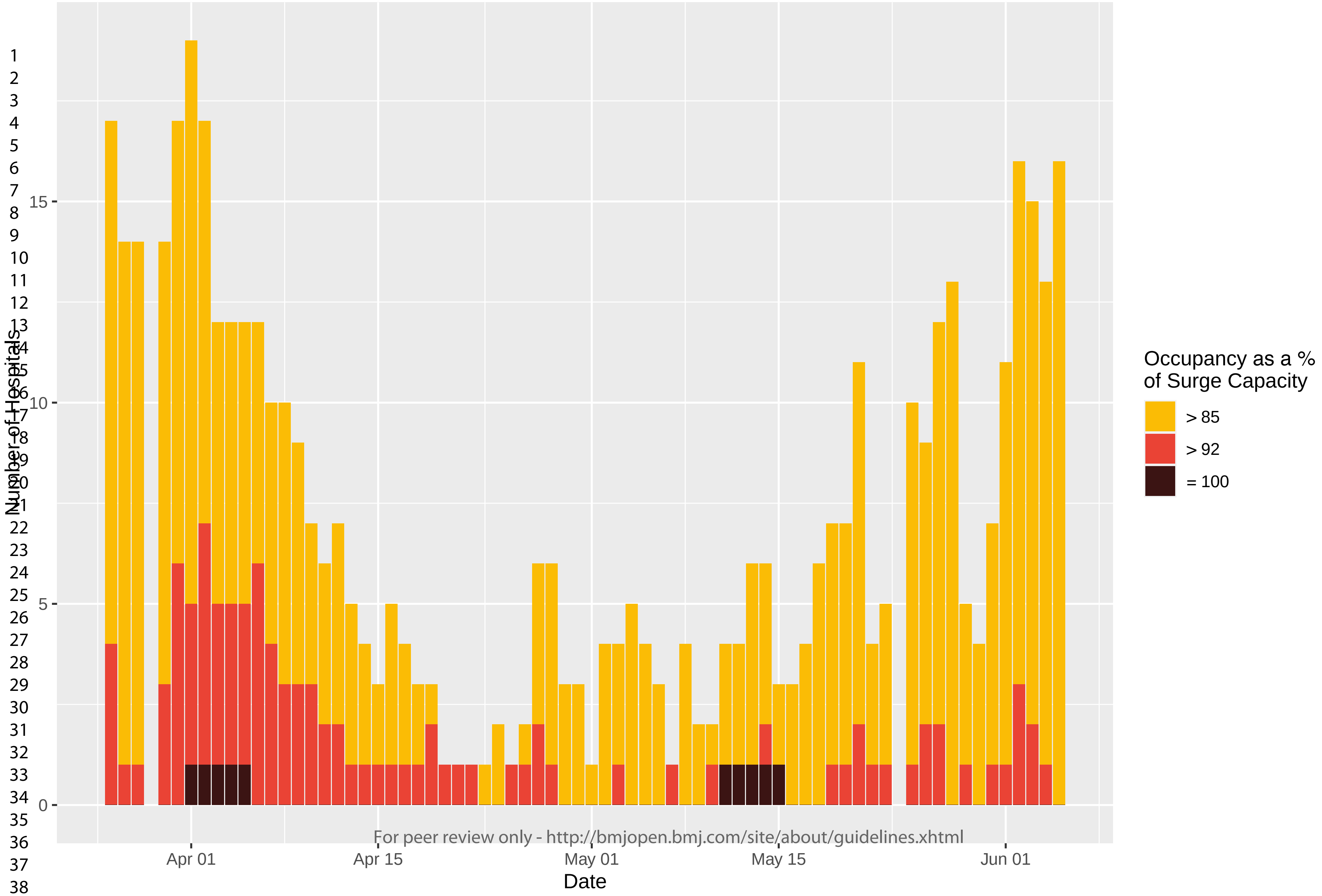

For peer review only - http://bmjopen.bmj.com/site/about/guidelines.xhtml

Figure 4: Number of Hospitals Operating Above Various Ventilated Bed Surge Capacity Thresholds

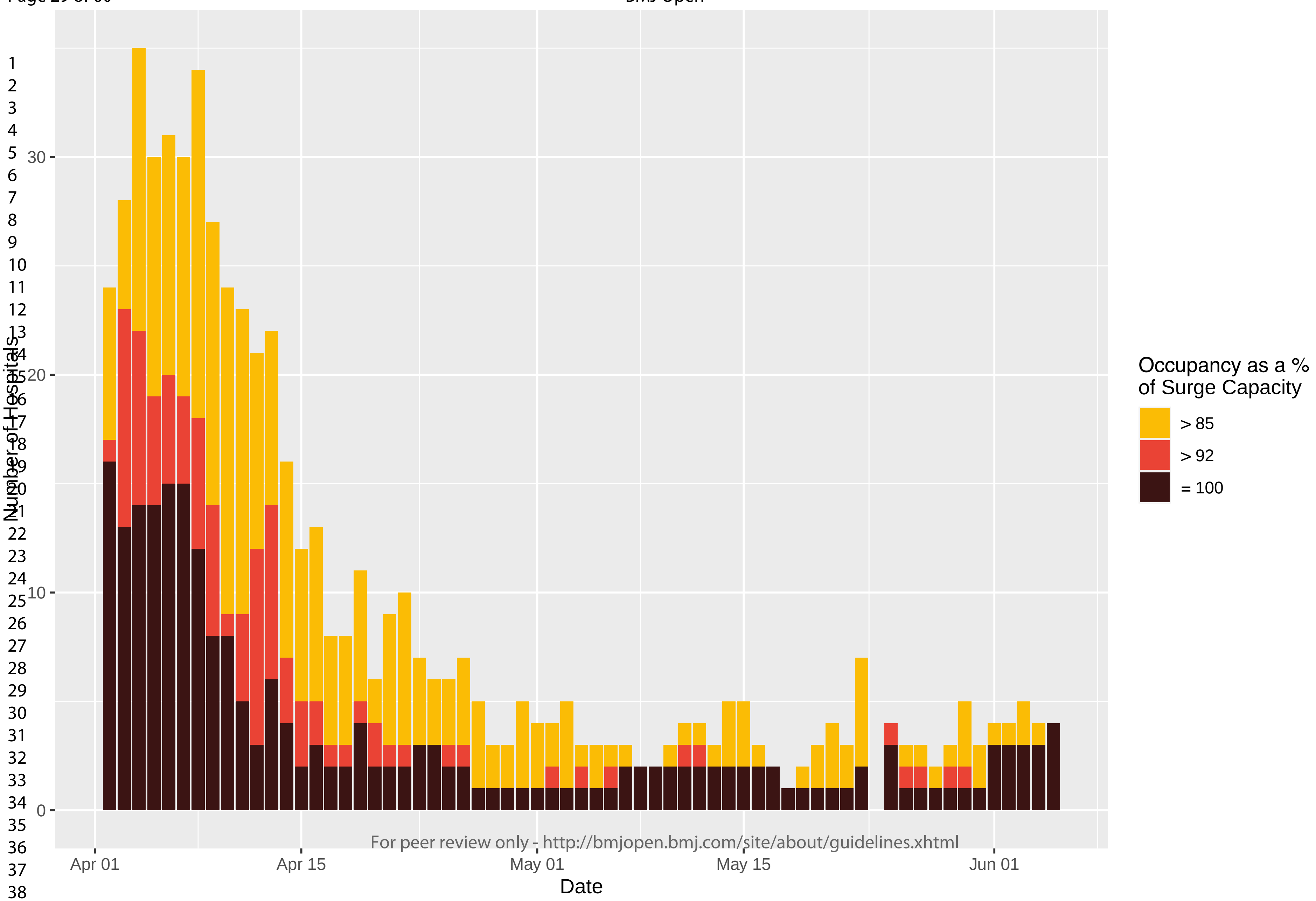

Figure 5: Number of Trusts Operating Above Various Ventilated Bed Surge Capacity Percentages

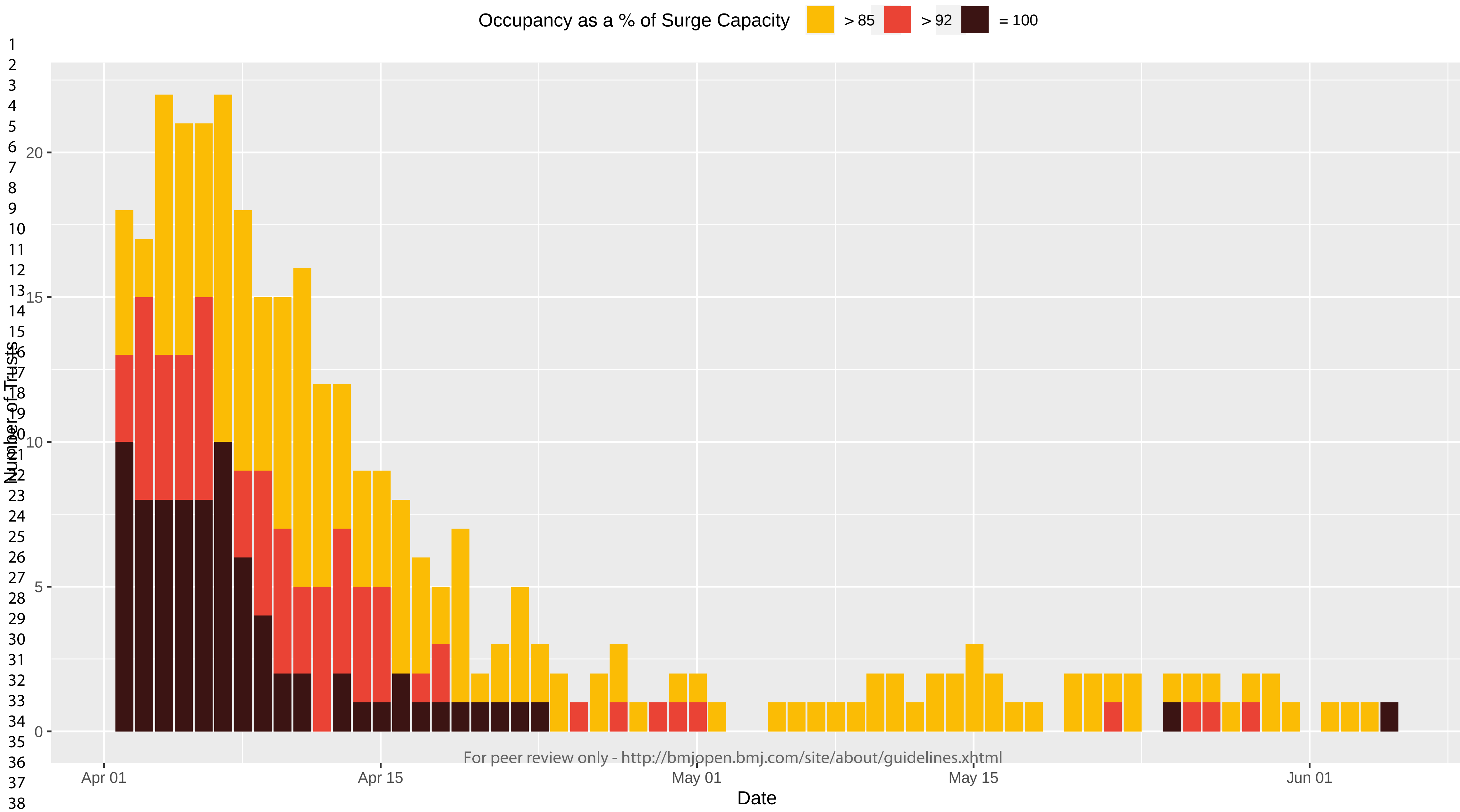

For peer review only - http://bmjopen.bmj.com/site/about/guidelines.xhtml

Figure 6: Peak STP Bed Occupancy Based on Surge Capacity

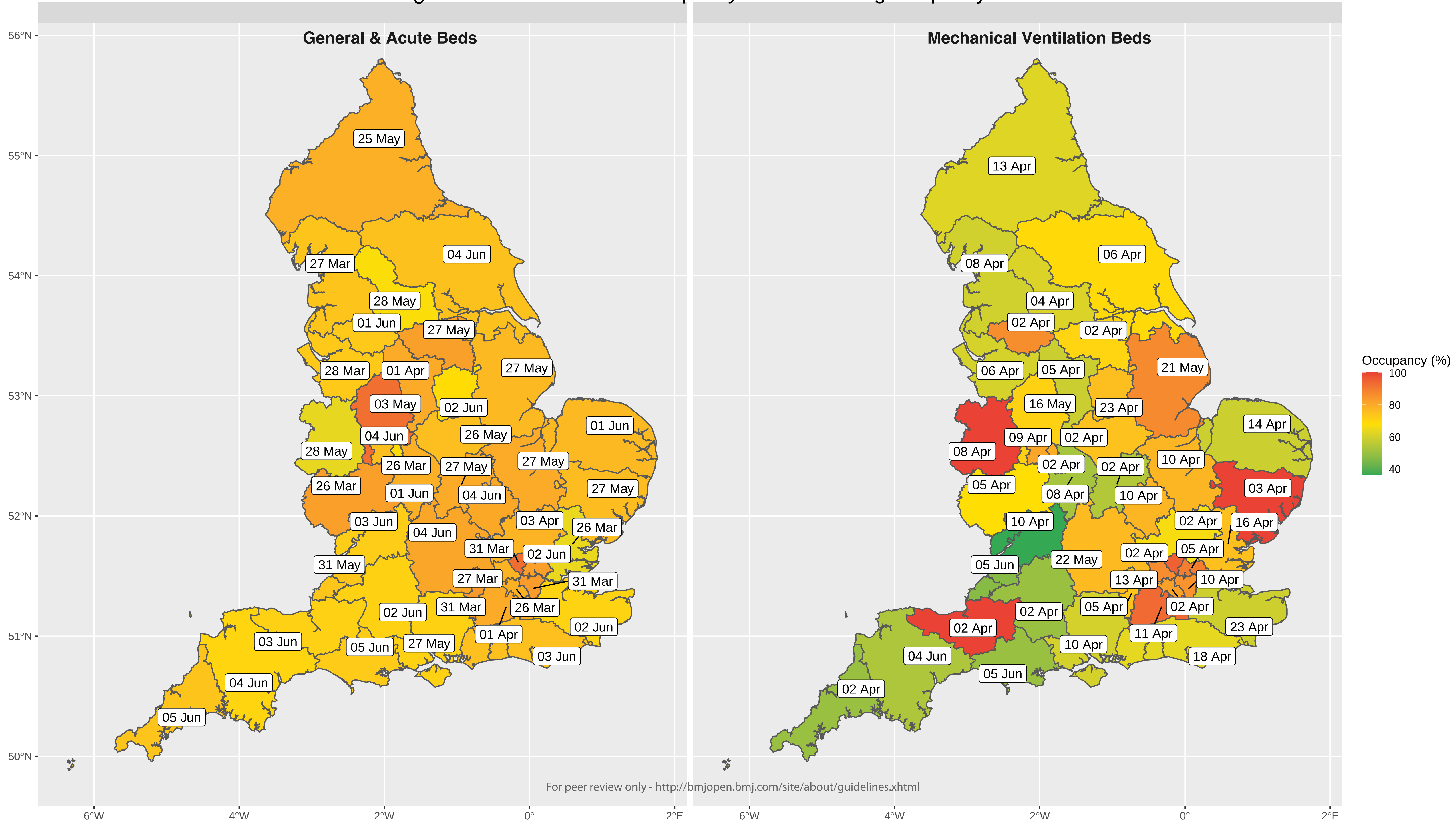

Supplementary Material

Supplementary Methods

Representativeness of Sample
Using the 2nd of May (randomly chosen) as an exemplar date, the non-specialist acute trusts to which we have restricted this survey represented 6,359 of the 6,866 beds (i.e. 92.6%) compatible with mechanical ventilation across England (comprising all institutions reporting to SitRep). Similarly, for all bed types, our sample represents 92.4% (i.e. 98,882 or the total 106,981 across England).

Organizational Units of Healthcare Provision
Although hospitals are relatively self-explanatory, the remaining units may require further context for readers unfamiliar with the organization structure utilized in the UK, as such, the following are brief summaries of the higher-order units of healthcare provision. Trusts are the core functional unit of hospital-based (i.e. secondary) care provision in England. They represent the first-level of aggregation above individual sites/hospitals, i.e. a trust is a collection of 1 or more geographically co-located hospitals which for specific administrative reasons operate as a single entity, although individual hospitals retain differing degrees of financial and operational autonomy depending on the specific trust structure. STPs are the aggregated unit of trusts, in combination with other units of healthcare provision, such as Clinical Commissioning Groups which administer the portion of the healthcare budget allocated to a specific geographic locale. There are 42 STPs each mapped to a specific health-geography 'footprint', and their express purpose was to deliver improvements pertaining to efficacy of services and integration of geographically co-located care providers.[35] The STPs are then mapped to 7 distinct geographical regions across England.

COVID-19 Status Recording
Data reported with reference to COVID-19 status, for example the number of general and acute beds occupied by individuals with the infection, refers to those whom had a confirmed positive result from a reverse transcriptase polymerase chain reaction (PCR) of nasopharyngeal and/or oropharyngeal swab.[36\] PCR was the only available testing method during the study period. Although national testing policy changed throughout the study period, all people for whom there was a suspicion of COVID-19 infection and who were admitted to hospital were tested, and potentially re-tested multiple times if the initial results were negative but clinical suspicion remained high (recorded as suspected COVID; for the purposes of the subsequent analysis 'confirmed' and 'suspected' were treated as one group due to the relatively small numbers reported for the latter).

Historical Baselines for Bed Availability
Baseline data comprised: 1) the average number of general and acute (G&A) beds available between January-March 2020, sourced from previously published routine situation report (SitRep) data;[37] 2) the number of critical care beds prior to the first reported case of COVID-19 in the UK, i.e. the value reported on the 30th January 2020 Critical Care SitRep,[38] and similarly to previous modelling studies was used for the baseline availabilities of HDU / ITU, critical care and ventilated beds;[30] 3) the maximum theoretical capacity for field hospitals was based on official government press releases;[39,40] and, 4) independent sector provider baseline capacity extracted from appendix 1 of the NHS England documentation confirming the 14 week block contract with the Independent Healthcare Providers Network.[28] Baselines were available for all of the trusts from the sources mentioned above and was propagated through into the STP and Regional datasets alongside aggregation of other values. Baseline bed numbers were not available for site level data. The choice of the period prior to the first wave of the pandemic instead of the historical baseline from 12 months prior was informed by two important piece of information: 1) the UK has experienced a gradual downward trend in bed numbers [1], and thus to be able to use the comparable period from 2019 we would have required an adjustment for that trend to produce a realistic baseline (there was a chance that we would have hypothesized there being more beds than were created after the first few weeks of mobilization by over-estimating the baseline number without this correction); 2) we deemed that use of the exact number of beds available at the time of operational planning (i.e. in February/early march) had greater ecological validity, as this was about reflecting the change from what we know was available rather than an abstracted version of what might have existed relative to similar periods in previous years.

Quality Control
All of the data was acquired through the daily site reports provided by NHS-Improvement & NHS England. These reports were loaded and appended sequentially with checks to ensure consistency in headings and data composition. The data spanned multiple sheets; these sheets were joined using Hospital, Trust and STP level

For peer review only - http://bmjopen.bmj.com/site/about/guidelines.xhtml

codes where appropriate. In some cases it was necessary to resort to using site names where no codes were present on the sheets containing hospital-level information regarding general and acute and critical care bed availability and occupancy. It was immediately apparent that extracting comprehensive site-level data from these records was non-trivial and for reasons discussed later, we maintain two datasets moving forward: one at site-level and one at trust-level that is used to aggregate to STP, Regional and Total figures as well.

Bed availability and total occupancy was recorded directly for G&A and critical care beds, alongside percentages of covid-confirmed occupants allowing for the calculation of a covid / non-covid / unoccupied breakdown for the G&A beds *only* (due to discrepancies in the definition of HDU / ITU and critical care beds, the percentage occupancy for critical care beds often resulted in impossible values of over 100%; it was decided to forego calculating a covid-breakdown for these beds due to how prolific these inconsistencies and issues were). For all of the other bed types, data was recorded in a different way. The number of covid positive patients (and in some cases covid-suspected patients), non-covid patients and the remaining unoccupied beds were recorded, allowing for total occupancy and availability to be calculated through simple transformation of these columns.

There are two key dates and several more minor milestones in the period we have data for (26th March to 5th June) where significant, non-trivial changes occurred in the site report structure and content. Prior to April 1st there was no information on bed availability beyond G&A and Critical Care beds; only the number of covid-positive patients were recorded for each type of bed. After the 1st of April, more granular bed availability was provided along with the means to work out the covid/non-covid breakdown of occupancy for Ventilated beds. From the 27th April onwards similar breakdowns and availability were recorded for HDU / ITU, IDU and most other types of bed at a site-level.

After loading in the data and accounting for the above described changes to its composition, the trust-level data used for the majority of our analysis had:

- 8.7% of Ventilated bed non-covid and unoccupied numbers missing across all records (no missing records for covid occupancy)
- No missing records for G&A, Critical care bed availability and occupancy
- No missing records for HDU / ITU after April 26th, otherwise 45.0%
- All other columns containing information regarding the hospital, trust, etc. were complete

Both datasets were filtered to remove children's hospitals, mental health hospitals and other sites / trusts that were not relevant to the analysis. STP linkage data was acquired via NHS Digital's library of public datasets source: (https://digital.nhs.uk/services/organisation-data-service/data-downloads/other-nhs-organisations) and augmented to include populations within STPs to facilitate our "beds-per-capita" figures (values were scrapped manually from the NHS England website, source: https://www.england.nhs.uk/integratedcare/stps/view-stps/). It was found that 7 trust codes were duplicated across 2 STPs; it was inappropriate to double count them so they were arbitrarily assigned to one of the STPs. The following table contains the STP and Trust code pairs that were chosen / removed from the linkage data to ensure a one-to-one mapping:

STable 1: STP and trust code pairs (for duplicated trusts)

Trust Code	STP Code (Assigned)	STP Code (Removed)
RDU	QRL	QNQ
RFS	QJ2	QF7
RK9	QJK	QT6
RMC	QOP	QHM
RNN	QE1	QHM
RVR	QXU	QWE
RVY	QYG	QE1

Additionally, due to some trust-level mergers that took place and missing data in the source, 4 updated STP-Trust pairs were manually added to the linkage data to facilitate their inclusion in the analysis (source:

For peer review only

https://www.england.nhs.uk/integratedcare/stps/view-stps/).

STable 2: Updated Trust-STP pairs (for merged trusts)

Trust Code	STP Code
RBA	QSL
RA3	QUY
RTP	QNX

Finally, it was found that two STPs spanned two regions. It was decided that QHM should fall under the North West region (all but one of its trusts are in that region) and QF7 should fall under the Midlands region (all but one of its trusts are in that region). The region definitions are inferred from the regions assigned to each trust in the site reports making up our primary dataset.

Despite our best efforts there were some missing values that persisted in critical columns outside of the key milestones mentioned in the section above. Moreover, in preparing the data it was noted that on several occasions there were substantial and improbable changes in the number of available beds that lasted 24 hours (even after allowing for the weekly trend of cyclical fluctuations in beds availability), prior to reversion to a value that fit the overall trend. These outliers follow from the reasonable assumption of the presence of data entry errors; it was decided that a cleaning rule should be applied to the data to avoid these seemingly impossible daily fluctuations and outliers.

First, a rolling median centred on each record was calculated using the 5 applicable days surrounding the record (smaller windows used at extremities of the data with correction not being possible at its absolute extremes). Missing values as well as values deemed to be outliers (a change greater than the 95th percentile of all differences between each record and the centred median spanning five days around it) were replaced with the aforementioned rolling median values. Highly improbable fluctuations were filtered out and missing values could be imputed in a robust way. This imputation and outlier detection process was applied to every applicable bed column spanning every type contained in the data. Only after this cleaning took place were other columns created through transformation, e.g. the number of available ventilated beds etc. The effect of cleaning the data is shown below in a before and after comparison, 4 trusts were chosen for their high initial volatility in G&A bed occupancy (See SFigure 1 & SFigure 2).

Statistical Analysis Notes

Temporalized values, i.e. hospital-days, were calculated by multiplying the absolute number of each functional unit for which data was available, and the number of days for which data is available for each.

After cleaning the data, two more key issues had to be dealt with in the trust-level and site-level datasets respectively:

1. Due to the aforementioned trust-level mergers, the composition of the data changed slightly throughout its duration. In an effort to achieve consistency, we merged and coalesced records prior to each mergers' appearance in the data to match their state post-merger. I.e. any rows corresponding to trusts that were eventually merged into some other trust were merged consistently throughout the dataset, even before this change actually took place. This was applied to records for the trusts RQ8, RDD and RAJ which were merged to fall under the single code RAJ on April 1st. This was also applied to RC9 and RC1 merged into RC9, and RA7 and RA3 merged into RA7: mergers that also occurred on April 1st and were reflected in the data shortly after.
2. It was observed that in one of the sheets relied upon for ventilated bed numbers, separate rows were included for both the sites and the corresponding trusts (given a "catch-all" label as their organisation type rather than "site"). In cases where only one hospital was associated with a trust, the numbers for that hospital were sometimes - inconsistently - recorded in the catch-all row rather than the site row as was done fairly consistently across all other situations. To achieve consistency without losing significant portions of the site-level data, we coalesced those rows where only one site was present and the catch all row contained numbers whilst the site row had zeroes or missing values. In order to achieve this, the organisation types of the two rows were swapped so that the catch-all row would be used in place of the site row, such that the site code and name was consistent throughout the entirety of the data.

For peer review only - http://bmjopen.bmj.com/site/about/guidelines.xhtml

Data Limitations

One persistent concern was the formulas by which bed occupancy proportions were generated. For example, the COVID-19 specific G&A bed percentage-occupancy was initially calculated as the sum of COVID-19 patients in IDU (infectious disease unit) beds and COVID-19 patients in "any other beds" divided by the total number of available G&A beds. This eventually changed to being the sum of the number of mechanical ventilated beds, non-invasive ventilated beds, oxygen-supporting beds and "any other beds" occupied by COVID patients minus the number of HDU / ITU beds occupied by confirmed COVID patients, all divided by the total number of available G&A beds. Whilst this is not in-and-of-itself problematic, the nature of the "any other beds" item was deemed concerning by the authors.

To understand the aforementioned concern, we first need to explain the data specification in more detail. It was noted that columns of the form "Number of Covid-19 confirmed patients in … beds at 0800" did not seem to contain values consistent with "Number of … beds available, as at 08:00 (COVID)", which we expected to have mirrored values. Importantly, the latter set of columns did not contain an "any other bed" column. As such, the formula used by NHS-E in the above calculation of G&A bed proportions drew the "any other beds" value from the first set of data, whereas all of the other information was drawn from the latter columns as they were internally consistent. We acknowledge that the use of this formula could have introduced an error of unknown magnitude or direction (as the two versions of data reporting were not consistent). Similar issues were seen with the independent sector data as well.

SFigure 1: G&A Bed Availability Across the Most Volatile Trusts in Terms of G&A Bed Occupancy (Pre−Correction)

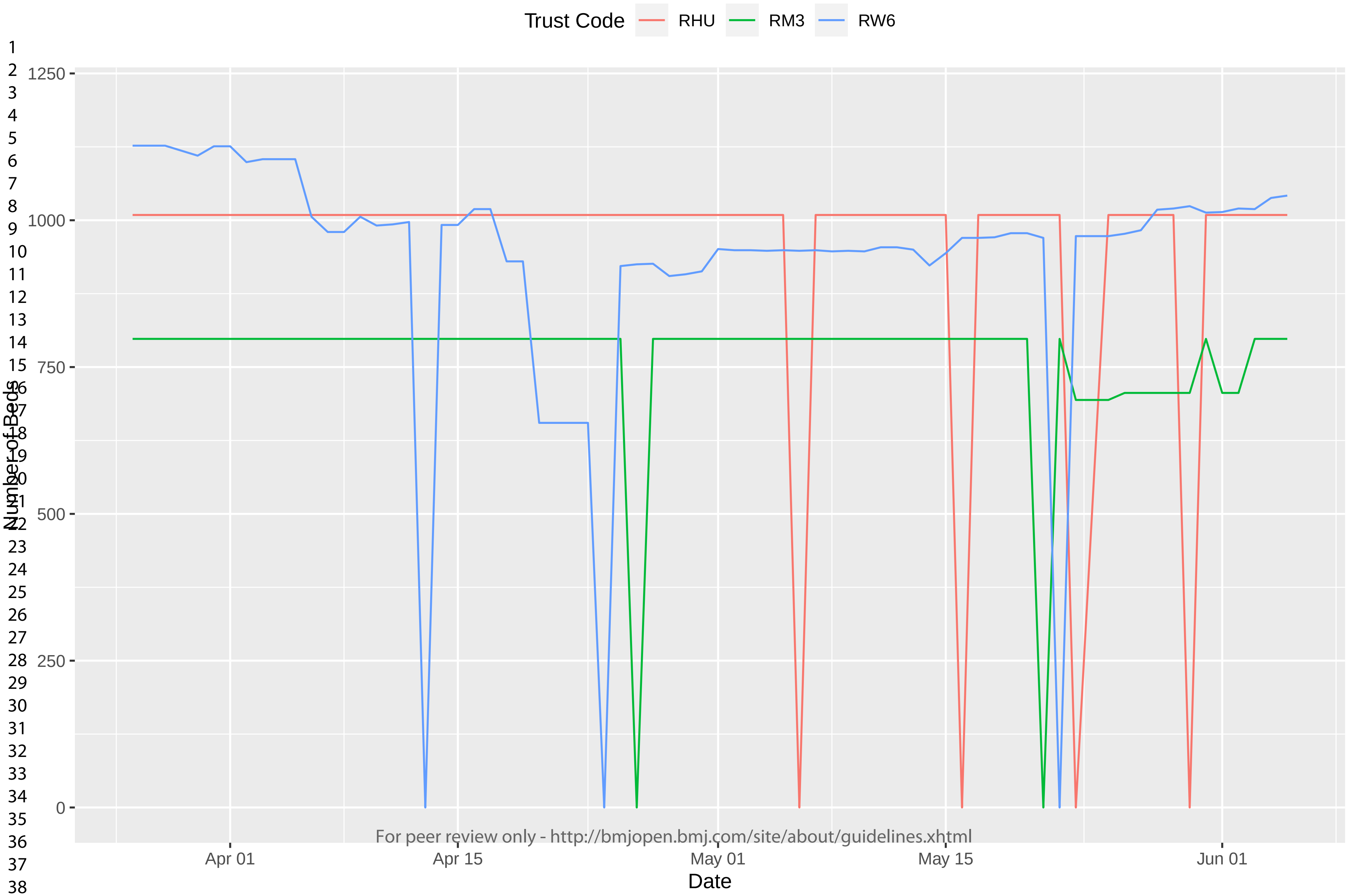

For peer review only - http://bmjopen.bmj.com/site/about/guidelines.xhtml

SFigure 2: G&A Bed Availability Across the Most Volatile Trusts in Terms of G&A Bed Occupancy (Post−Correction)

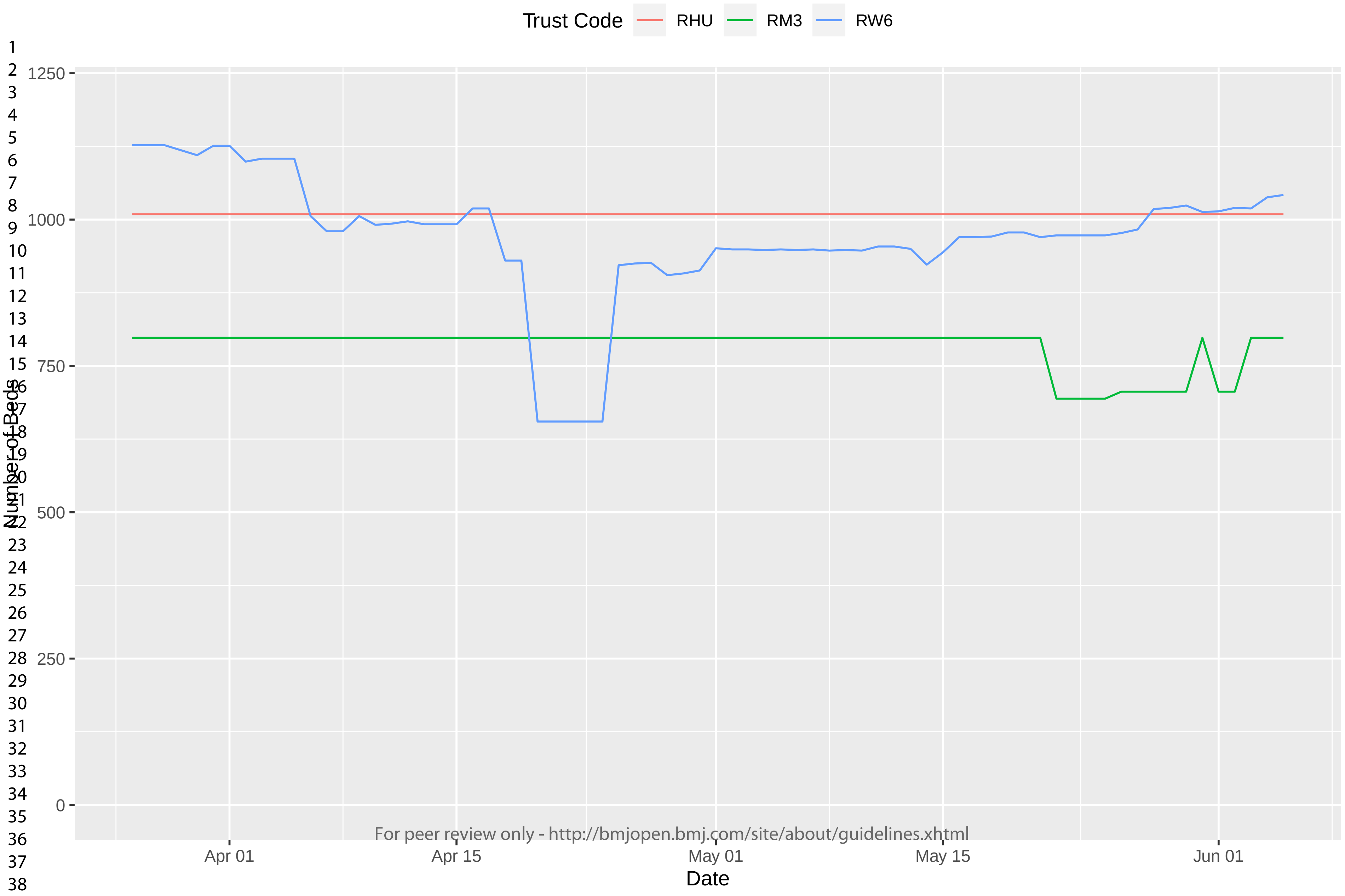

Supplementary Results

STable 1: Descriptive Summaries of the Size and Geographic Locations of Hospitals Stratified by the Peak Occupancy Achieved

	Hospitals reaching 100% saturation of mechanical ventilation beds (n = 51*)	Hospitals reaching >92%, but not 100% saturation of mechanical ventilation bed (n = 20)	Hospitals reaching >85%, but not 92% saturation of mechanical ventilation bed (n = 19)	All other Hospitals (n = 77**)
**Peak G&A bed capacity (Median [Range])**	438 [197 - 1012]	484 [256 - 841]	459 [253 - 910]	558 [44 - 1499]
**Peak mechanical ventilation compatible bed capacity (restricted to April 2nd onwards) (Median [Range])**	29 [7 - 77]	49 [18 - 141]	33 [15 - 153]	40 [7 - 159]
**Peak HDU/ITU bed capacity (restricted to April 27th onwards) (Median [Range])**	29 [0 - 99]	45 [17 - 161]	37 [14 - 157]	40 [6 - 152]
Location				
England (n = 167)	***51 (30.5%)***	***20 (12.0%)***	***19 (11.4%)***	***77 (46.1%)***
**London (n = 27)**	6 (22.2%)	11 (40.7%)	7 (25.9%)	3 (11.1%)
**Midlands (n = 27)**	11 (40.7%)	3 (11.1%)	1 (3.7%)	12 (44.4%)
**East of England (n = 20)**	7 (35.0%)	2 (10.0%)	1 (5.0%)	10 (50.0%)
**South West (n = 17)**	3 (17.6%)	0 (0.0%)	0 (0.0%)	14 (82.3%)
**South East (n = 24)**	6 (25.0%)	2 (8.3%)	3 (12.5%)	13 (54.2%)
**North East and Yorkshire (n = 29)**	8 (27.6%)	1 (3.4%)	4 (13.8%)	16 (65.5%)
**North West (n = 23)**	10 (43.5%)	1 (4.3%)	3 (13.0%)	9 (39.1%)

* One hospital is excluded from this n and the subsequent calculations as it does not have any information regarding G&A beds, despite reaching 100% capacity for mechanical ventilator bed capacity.
** 5 hospitals were excluded from this table as they had no ventilated beds at any time, or no data was available for their ventilated bed capacity in the dataset.

For peer review only

SFigure 3: Regional Bed Occupancy Across England, Stratified by COVID-19 Status

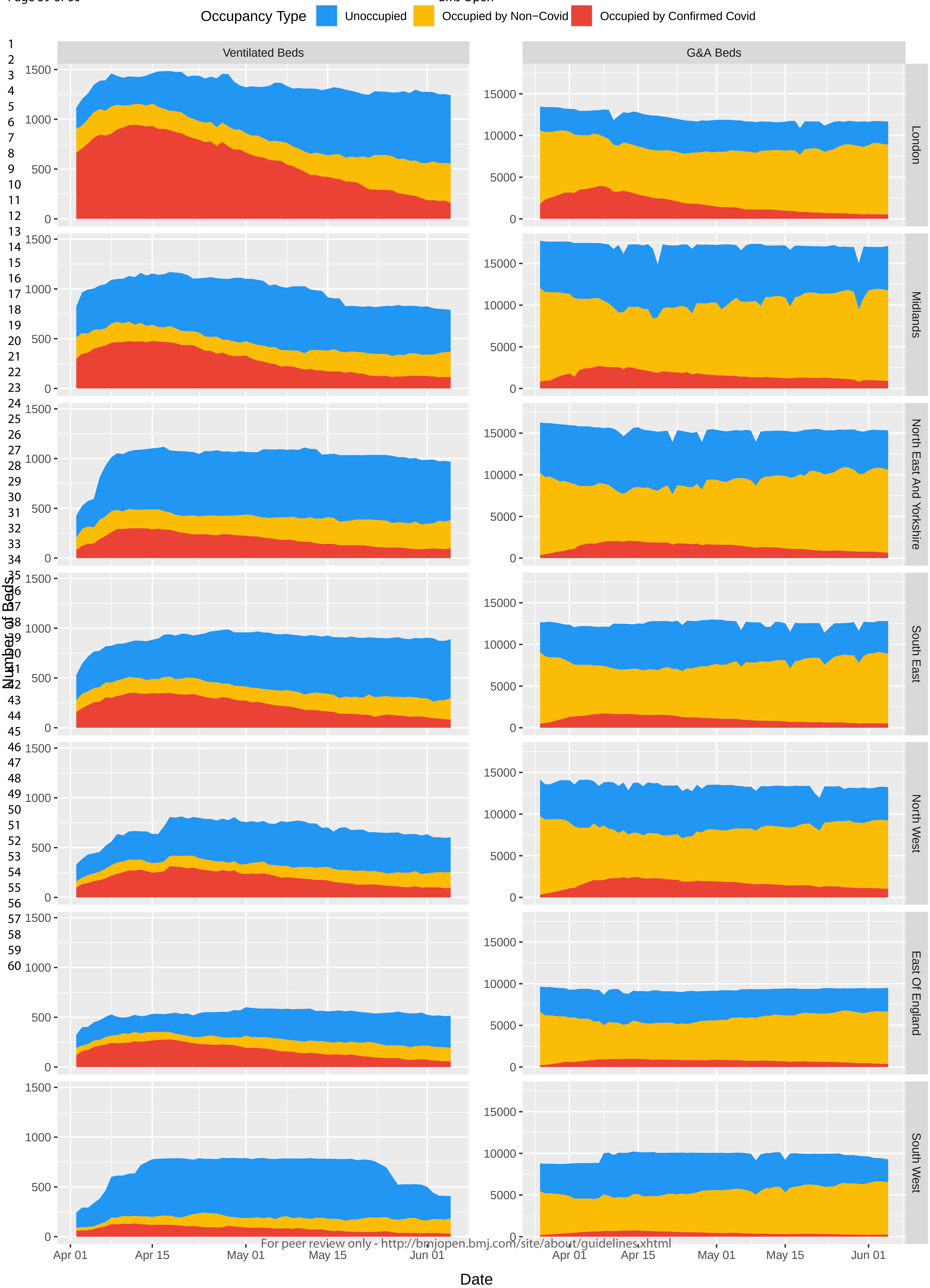

SFigure 3: Bed Occupancy Across England by geographical region, stratified by COVID-19 Status

Legend: SFigure 3A (Left) illustrates the time-varying trends in mechanical ventilator bed capacity and occupancy across the 7 regions of England, from March 27th to June 5th; note that availability information is only present from 1st April onwards. SFigure 3B (Right) illustrates general and acute bed capacity and occupancy across the 7 regions of England, from 1st April to 5th June. Occupancy in both figures is stratified by whether the individual in the bed has a positive COVID-19 test or not).
Critical Care Beds

For peer review only

Critical Care Beds
In the context of surge capacity, at the site-level, 1558 hospital days (13.1%; median number of days per hospital = 10 [range: 1 to 65]) were at or above 85% of capacity, which corresponds to 120 hospitals spending at least 1 day at, or above, the aforementioned threshold. 948 hospital days (8.0%; median number of days per hospital = 6 [range: 1 to 51]) were spent above 92%, representing 102 hospitals. And 88 (50.9%) hospitals reached 100% capacity, representing 640 hospital days at saturation (median number of days per hospital = 5 [range: 1 to 51])). At the trust-level, 965 trust days (11.0%; median number of days per trust = 8 [range: 1 to 56]) were at or above 85% of capacity, representing 80 trusts. 567 trust days (6.5%; median number of days per trust = 5 [range: 1 to 47]) were spent above 92%, representing 64 trusts. And 47 (37.6%) trusts reached 100% capacity, representing 339 trust days at saturation (median number of days per trust = 5 [range: 1 to 21])). At the STP-level, 138 STP days (median number of days per STP = 5 [range: 1 to 43]) were at or above 85% of capacity, representing 18 STPs. 74 STP days (median number of days per STP = 2 [range: 1 to 26]) were spent above 92%, representing 14 STPs. And 6 STPs reached 100% capacity, representing 34 STP days at saturation (median number of days per STP = 3 [range: 1 to 19])). See SFigure 4 for a visual summary of these results. See SFigure 5 for the aggregate occupancy, stratified by COVID-19 status at the regional level.

In the context of baseline capacity, at the trust-level, 2620 trust days (22.1%; median number of days per trust = 27 [range: 1 to 69]) were at or above 100% capacity, which corresponds to 92 trusts spending at least 1 day at, or above, their-pre-pandemic baseline. 230 trusts days (median number of days per trust = 9 [range: 1 to 49]) were at or above 200% capacity, which corresponds to 19 trusts spending at least 1 day more than 100% above their-pre-pandemic baseline. At the STP-level, 620 STP days (median number of days per STP = 24 [range: 1 to 63]) were at an occupancy-level above 100% of baseline availability, which corresponds to 27 STPs spending at least 1 day at, or above, their-pre-pandemic baseline. 44 STP days (median number of days per STP = 14 [range: 10 to 20]) were at an occupancy-level above 200% of baseline availability which corresponds to 3 STPs spending at least 1 day more than 100% above their-pre-pandemic baseline. See SFigure 8 for a visual summary.

HDU/ITU Beds
The following results should be interpreted in the context of the date range available, i.e. data is only present after the 27th of April. Thus, the results are likely a significant underestimation of peak occupancy as, in retrospect, the peak number of cases and fatalities in the UK were near the beginning of April.

In the context of surge capacity, at the site-level, 315 hospital days (2.7%; median number of days per hospital = 2 [range: 1 to 39]) were at or above 85% of capacity, which corresponds to 59 hospitals spending at least 1 day at, or above, the aforementioned threshold. 216 hospital days (1.8%; median number of days per hospital = 1 [range: 1 to 39]) were spent above 92%, representing 45 hospitals. And 40 hospitals reached 100% capacity, representing 192 hospital days at saturation (median number of days per hospital = 1 [range: 1 to 39])). At the trust-level, 192 trust days (median number of days per trust = 3 [range: 1 to 39]) were at or above 85% of capacity, representing 36 trusts. 122 trust days (median number of days per trust = 2 [range: 1 to 39]) were spent above 92%, representing 24 trusts. And 19 trusts reached 100% capacity, representing 106 trust days at saturation (median number of days per trust = 2 [range: 1 to 39])). At the STP-level, 138 STP days (median number of days per STP = 3 [range: 1 to 43]) were at or above 85% of capacity, representing 18 STPs. 74 STP days (median number of days per STP= 2 [range: 1 to 26]) were spent above 92%, representing 14 STPs. And 6 STPs reached 100% capacity, representing 34 STP days at saturation (median number of days per STP = 3 [range: 1 to 19])). See SFigure 5 for a visual summary.

For peer review only

SFigure 4a: Number of Hospitals/Trusts/STPs at Varying Critical Care Bed Occupancy Levels Compared to Surge Capacity

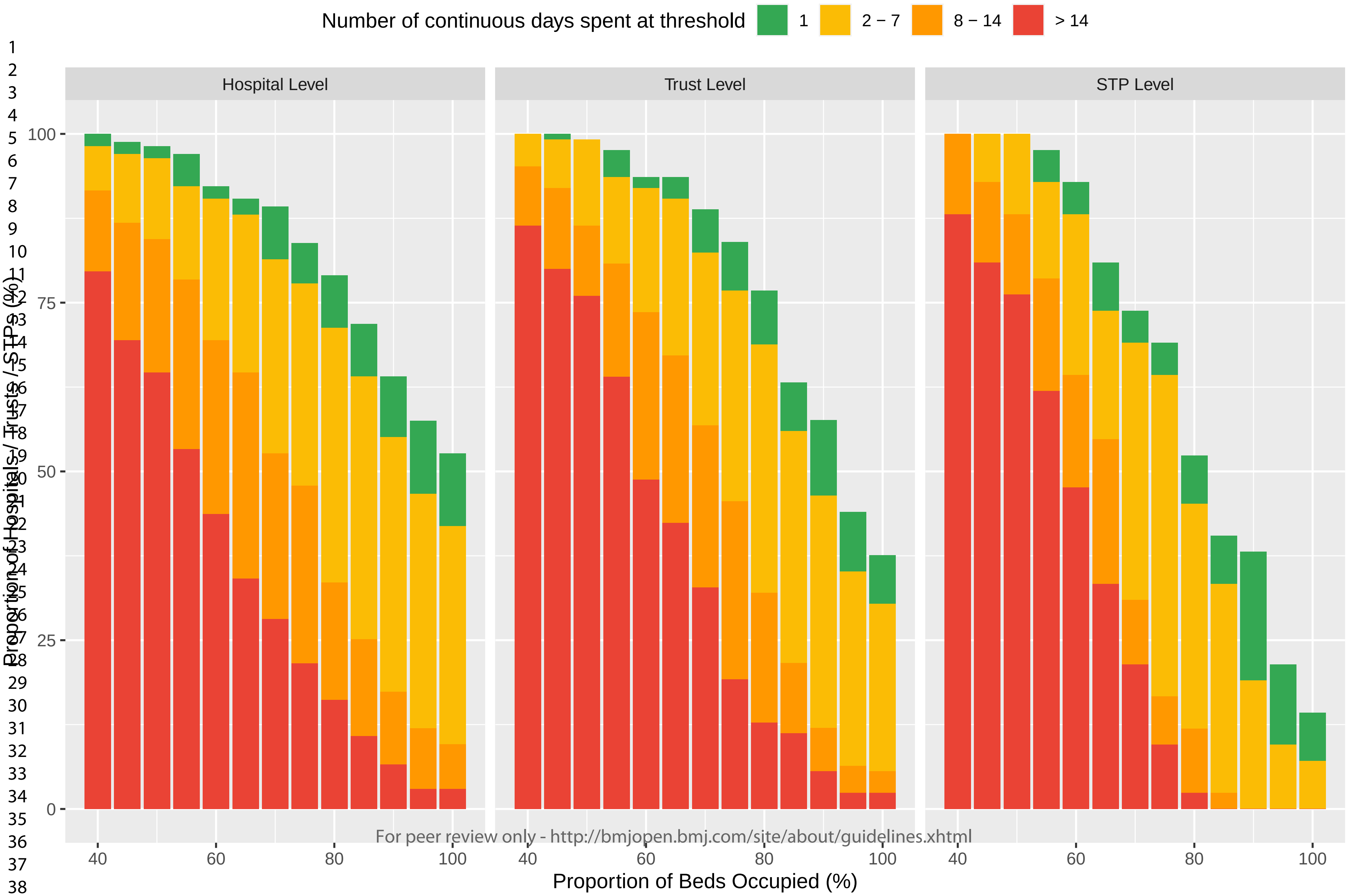

For peer review only - http://bmjopen.bmj.com/site/about/guidelines.xhtml

SFigure 4b: Number of Hospitals/Trusts/STPs at Varying HDU/ITU Bed Occupancy Levels Compared to Surge Capacity

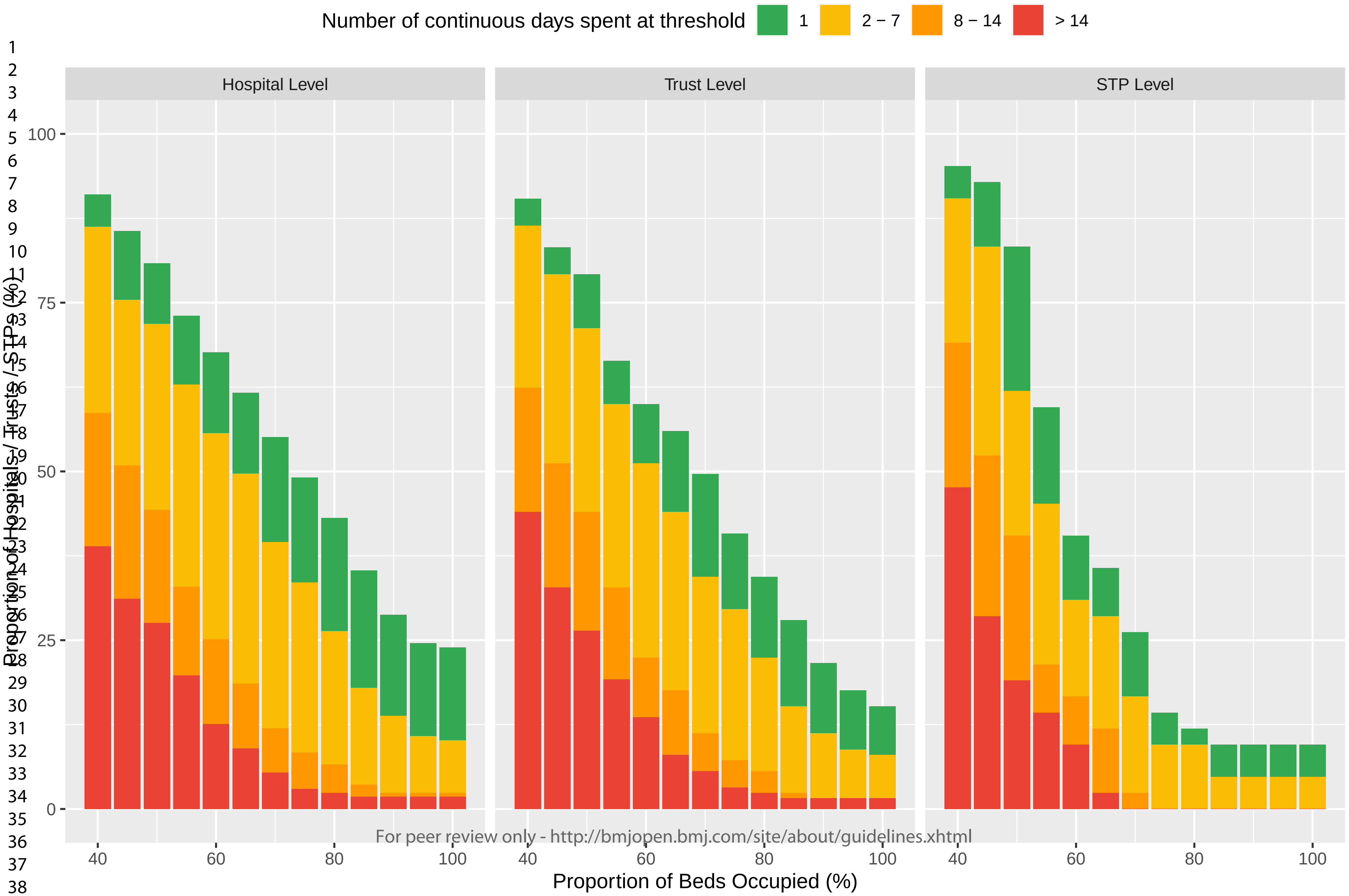

SFigure 4: Critical Care (Top) & HDU/ITU (Bottom) Occupancy (Based on Surge Capacities) Across England

Legend: SFigure 4A (Top) illustrates the proportion of hospitals/trusts/STPs at different occupancy thresholds for surge critical care bed capacity, across England, from April 1st to June 5th. SFigure 4B (Bottom) illustrates the proportion of hospitals/trusts/STPs at different occupancy thresholds for surge critical care bed capacity, across England, from April 1st to June 5th. The superimposed colours represent how long the trusts spent at each specific threshold.

For peer review only

SFigure 5: Number of Trusts/STPs at Varying G&A Bed Occupancy Levels Compared to Baseline Capacity

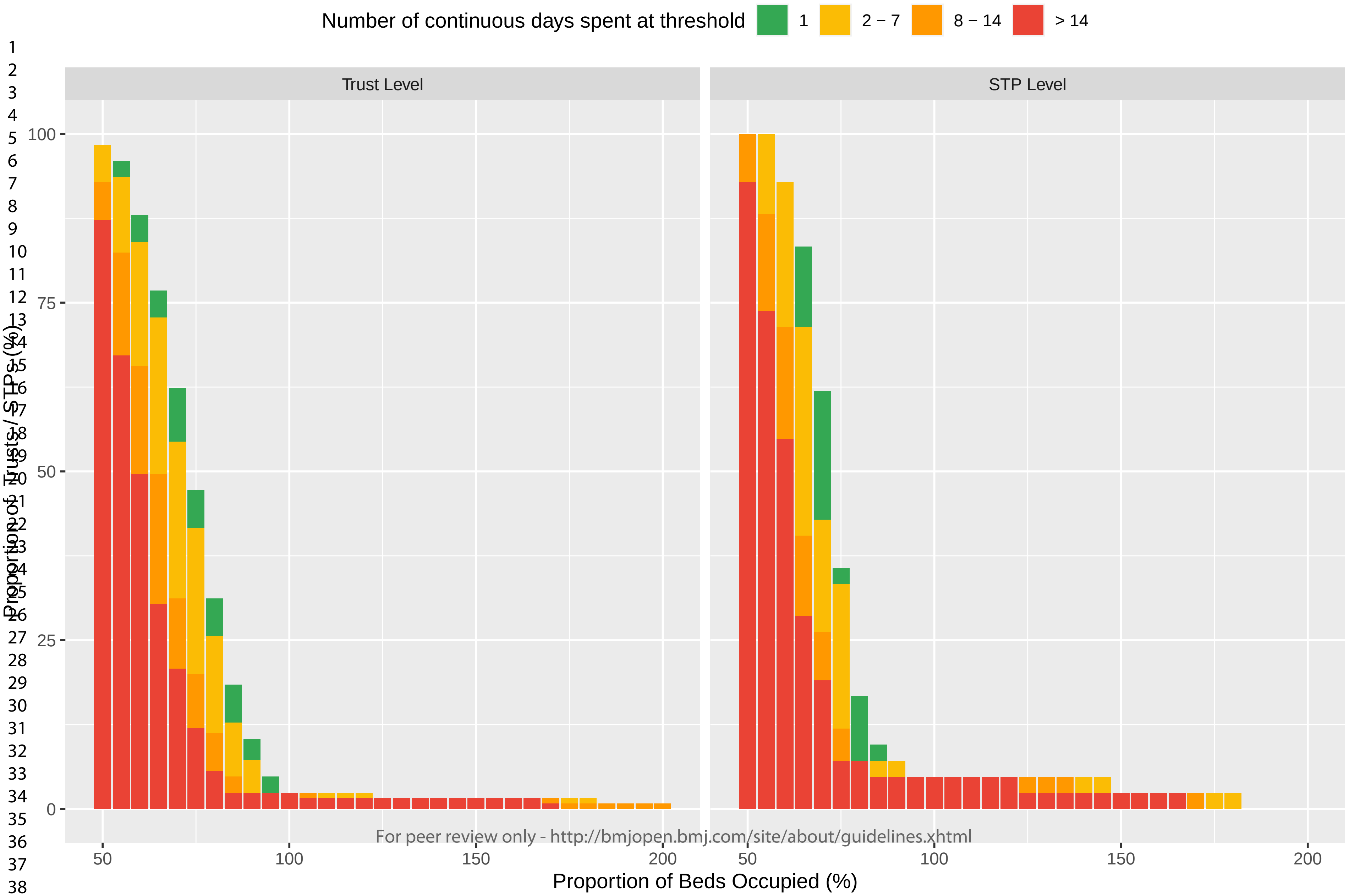

For peer review only - http://bmjopen.bmj.com/site/about/guidelines.xhtml

sFigure 5: Trust-Level General & Acute Bed Occupancy (Based on Baseline Capacities) Across England

Legend: The proportion of all trusts, and sustainability and transformation partnerships (STPs), at varying general and acute (G&A) bed occupancy thresholds relative to their baseline (mean availability January-March 2020) capacity, across England, from April 1st to June 5th. The superimposed colours represent how long the trusts spent at each specific threshold.

For peer review only

SFigure 6: Number of Trusts/STPs at Varying Ventilated Bed Occupancy Levels Compared to Baseline Capacity

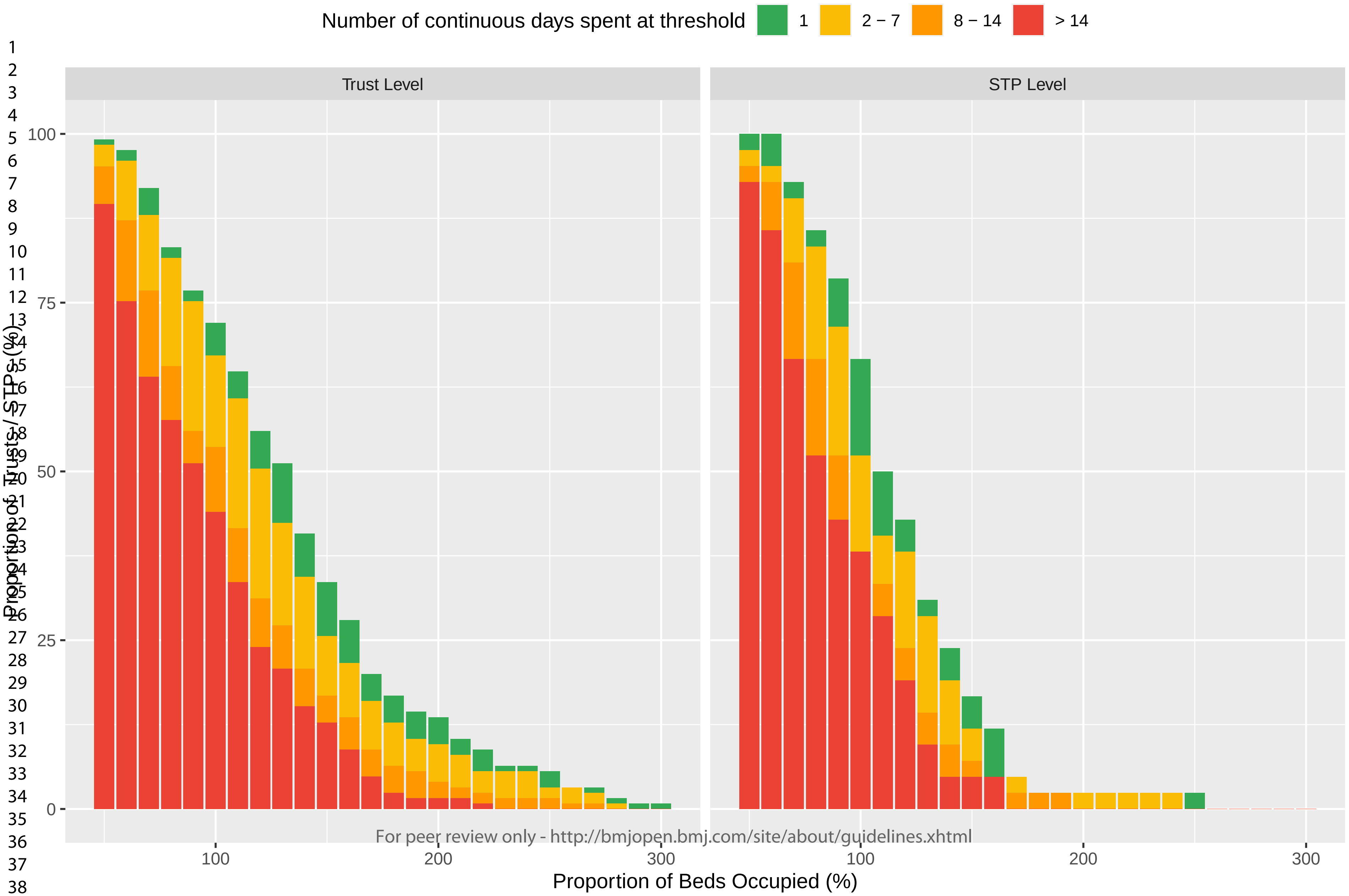

sFigure 6: Trust-Level Ventilator Bed Occupancy (Based on Baseline Capacities) Across England

Legend: The proportion of all trusts, and sustainability and transformation partnerships (STPs), at varying ventilator bed occupancy thresholds relative to their baseline capacity, across England, from April 1st to June 5th. The superimposed colours represent how long the trusts spent at each specific threshold.

For peer review only

SFigure 7: Regional Critical Care Bed Occupancy, Stratified by COVID-19 Status

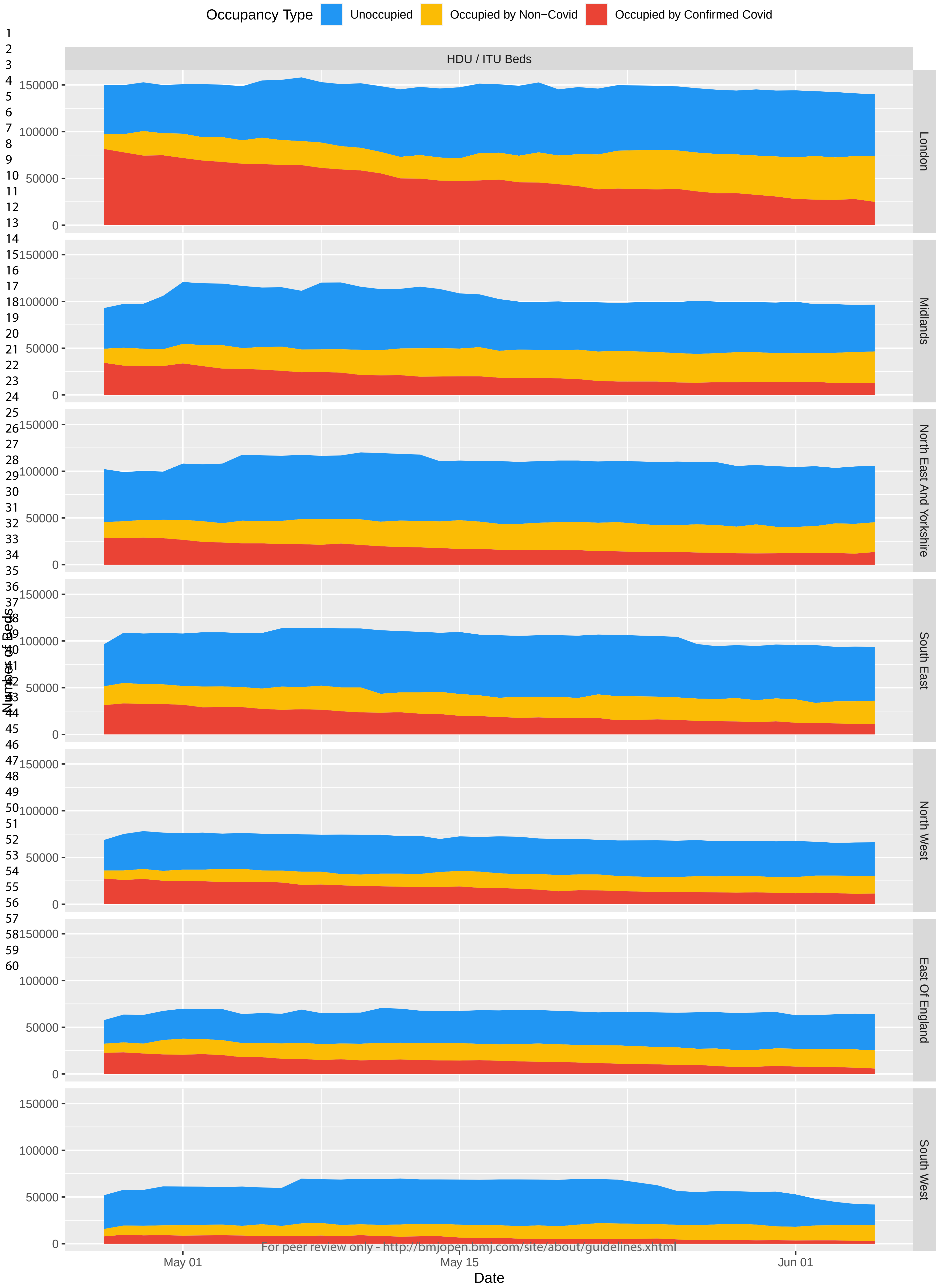

SFigure 7: Regional Critical Care Bed Occupancy, Stratified by COVID-19 Status

Legend: The time-varying trends in critical care bed capacity and occupancy across the 7 regions of England, from March 27th to June 5th. Occupancy is stratified by whether the individual in the bed has a positive COVID-19 test or not).

For peer review only

SFigure 8: Proportion of Trusts/STPs at Varying Critical Care Bed Occupancy Levels Compared to Baseline Capacity

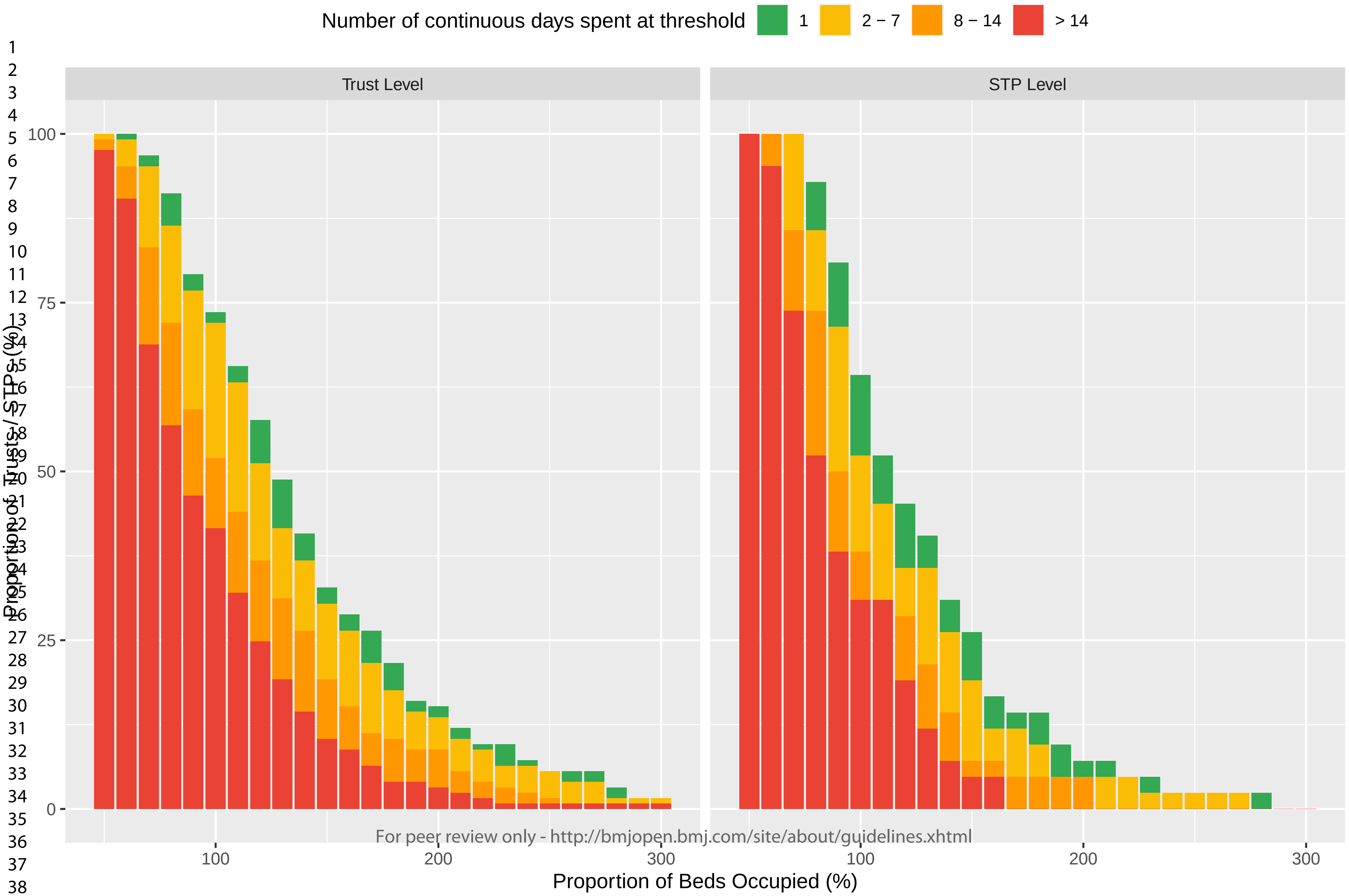

SFigure 8: Critical Care Bed Occupancy (Based on Baseline Capacities) Across England

Legend: SFigure 6A (Left) illustrates the proportion of trusts at different occupancy thresholds based on baseline critical care bed capacity, across England, from April 1st to June 5th. SFigure 6B (Right) illustrates the proportion of STPs at different occupancy thresholds based on their baseline critical care bed capacity, across England, from April 1st to June 5th. The superimposed colours represent how long the trusts spent at each specific threshold.

For peer review only

SFigure 9: Number of Trusts Operating Above Various Ventilated Bed Surge Capacity Thresholds

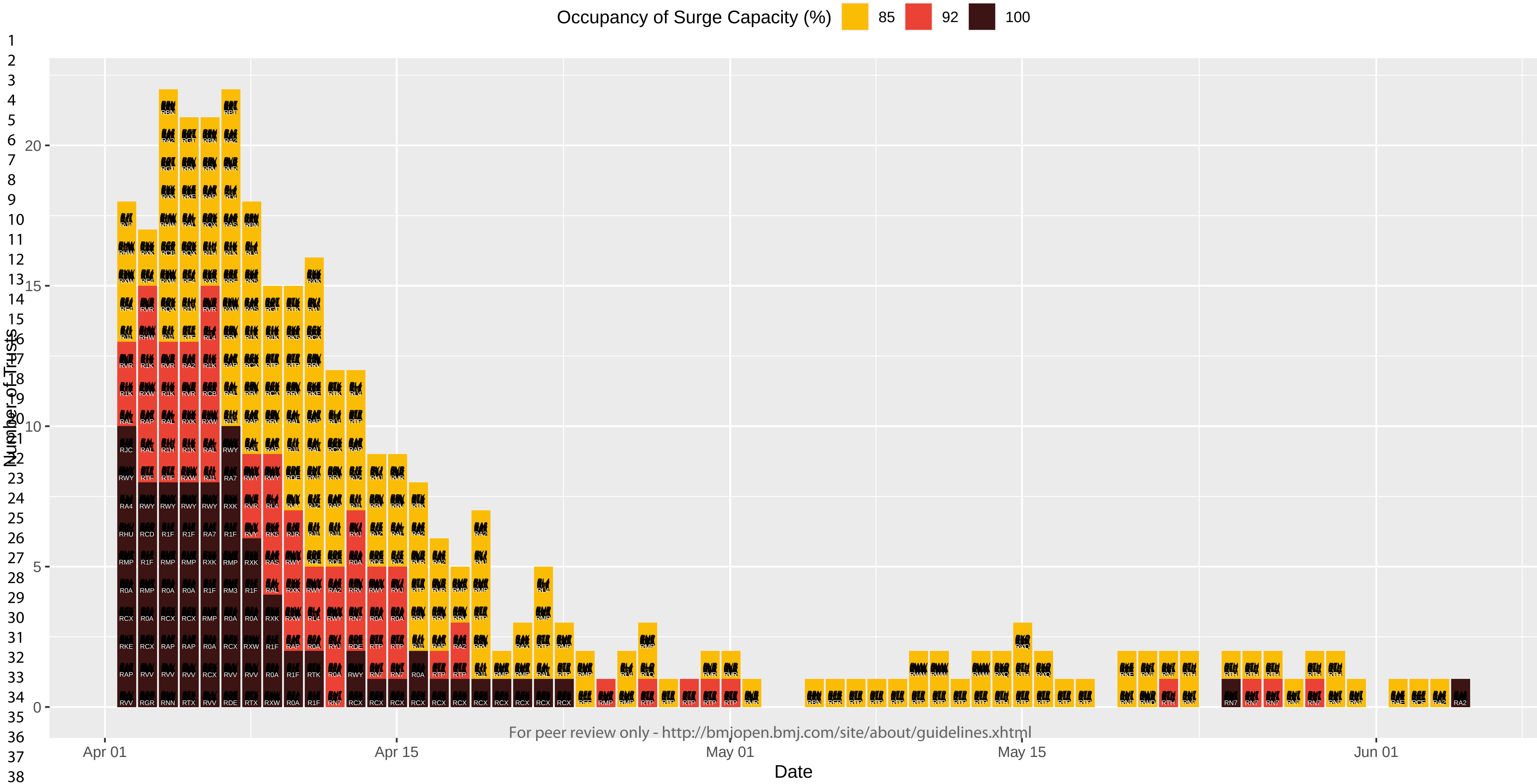

SFigure 9: Trust-Level Ventilator Bed Occupancy (Based on Surge Capacities) Across England

Legend: The conversion of trust code to name for all trusts included in the figure are: Manchester University NHS Foundation Trust (R0A), Isle Of Wight NHS Trust (R1F), Barts Health NHS Trust (R1H), London North West University Healthcare NHS Trust (R1K), Royal Surrey County Hospital NHS Foundation Trust (RA2), Yeovil District Hospital NHS Foundation Trust (RA4), University Hospitals Bristol NHS Foundation Trust (RA7), University Hospitals Bristol And Weston NHS Foundation Trust (RA7), Bradford Teaching Hospitals NHS Foundation Trust (RAE), Royal Free London NHS Foundation Trust (RAL), North Middlesex University Hospital NHS Trust (RAP), The Hillingdon Hospitals NHS Foundation Trust (RAS), Kingston Hospital NHS Foundation Trust (RAX), St Helens And Knowsley Teaching Hospitals NHS Trust (RBN), Mid Cheshire Hospitals NHS Foundation Trust (RBT), York Teaching Hospital NHS Foundation Trust (RCB), Harrogate And District NHS Foundation Trust (RCD), Airedale NHS Foundation Trust (RCF), The Queen Elizabeth Hospital, King's Lynn, NHS Foundation Trust (RCX), East Suffolk And North Essex NHS Foundation Trust (RDE), Barking, Havering And Redbridge University Hospitals NHS Trust (RF4), Barnsley Hospital NHS Foundation Trust (RFF), The Rotherham NHS Foundation Trust (RFR), West Suffolk NHS Foundation Trust (RGR), Cambridge University Hospitals NHS Foundation Trust (RGT), Portsmouth Hospitals NHS Trust (RHU), Royal Berkshire NHS Foundation Trust (RHW), Guy's And St Thomas' NHS Foundation Trust (RJ1), St George's University Hospitals NHS Foundation Trust (RJ7), South Warwickshire NHS Foundation Trust (RJC), Countess Of Chester Hospital NHS Foundation Trust (RJR), King's College Hospital NHS Foundation Trust (RJZ), Sherwood Forest Hospitals NHS Foundation Trust (RK5), Whittington Health NHS Trust (RKE), The Royal Wolverhampton NHS Trust (RL4), Wye Valley NHS Trust (RLQ), Salford Royal NHS Foundation Trust (RM3), Tameside And Glossop Integrated Care NHS Foundation Trust (RMP), Dartford And Gravesham NHS Trust (RN7), North Cumbria Integrated Care NHS Foundation Trust (RNN), Homerton University Hospital NHS Foundation Trust (RQX), Wrightington, Wigan And Leigh NHS Foundation Trust (RRF), University College London Hospitals NHS Foundation Trust (RRV), Northumbria Healthcare NHS Foundation Trust (RTF), Oxford University Hospitals NHS Foundation Trust (RTH), Ashford And St Peter's Hospitals NHS Foundation Trust (RTK), Surrey And Sussex Healthcare NHS Trust (RTP), University Hospitals Of Morecambe Bay NHS Foundation Trust (RTX), Epsom And St Helier University Hospitals NHS Trust (RVR), East Kent Hospitals University NHS Foundation Trust (RVV), Southport And Ormskirk Hospital NHS Trust (RVY), United Lincolnshire Hospitals NHS Trust (RWD), Warrington And Halton Hospitals NHS Foundation Trust (RWW), Calderdale And Huddersfield NHS Foundation Trust (RWY), Sandwell And West Birmingham Hospitals NHS Trust (RXK), Buckinghamshire Healthcare NHS Trust (RXQ), East Lancashire Hospitals NHS Trust (RXR), Shrewsbury And Telford Hospital NHS Trust (RXW), Imperial College Healthcare NHS Trust (RYJ)

For peer review only

SFigure 10a: Proportion of Hospitals/Trusts/STPs at Varying Ventilated Bed Occupancy Levels Compared to Surge Capacity

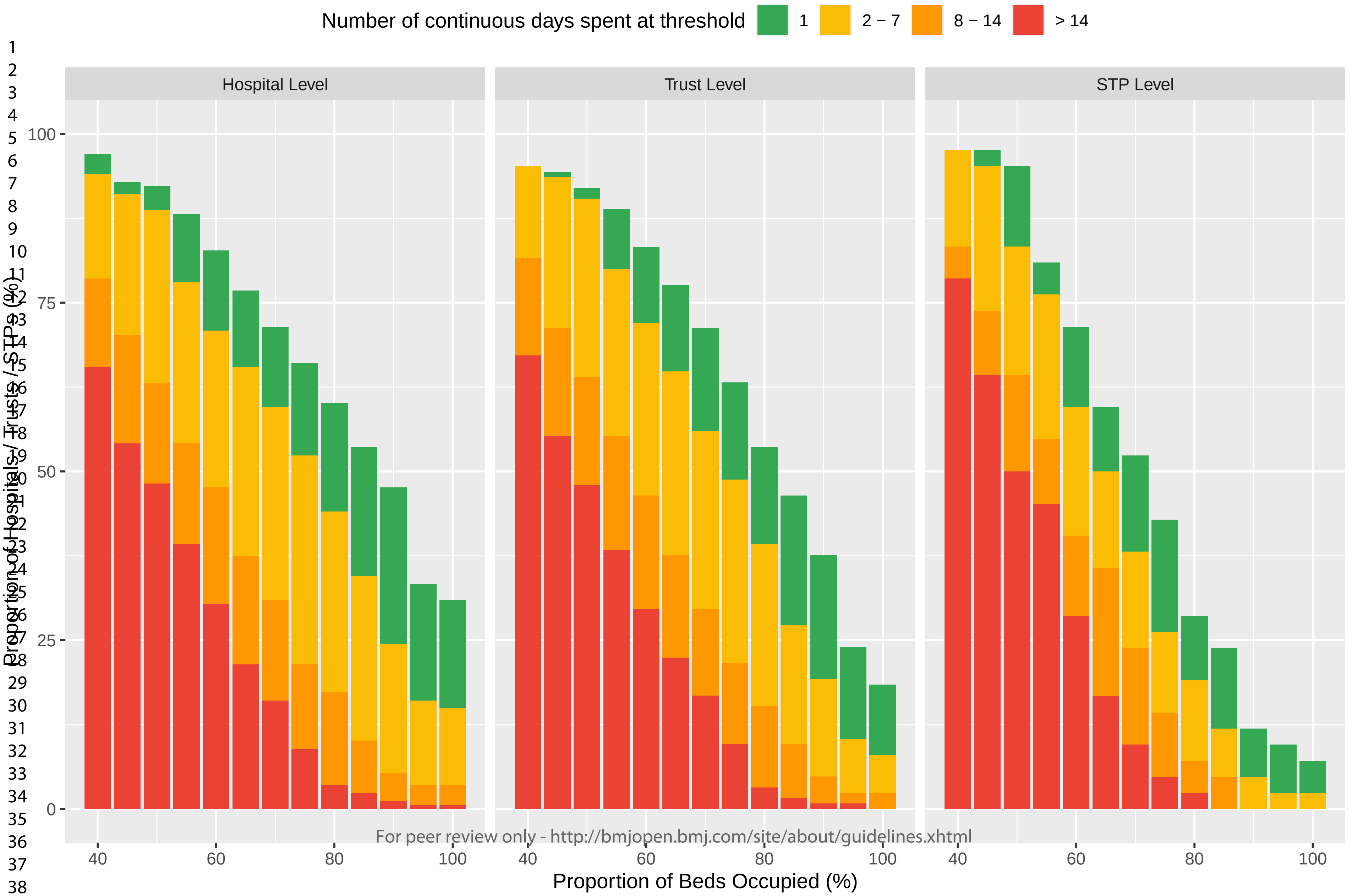

SFigure 10b: Proportion of Hospitals/Trusts/STPs at Varying G&A Bed Occupancy Levels Compared to Surge Capacity

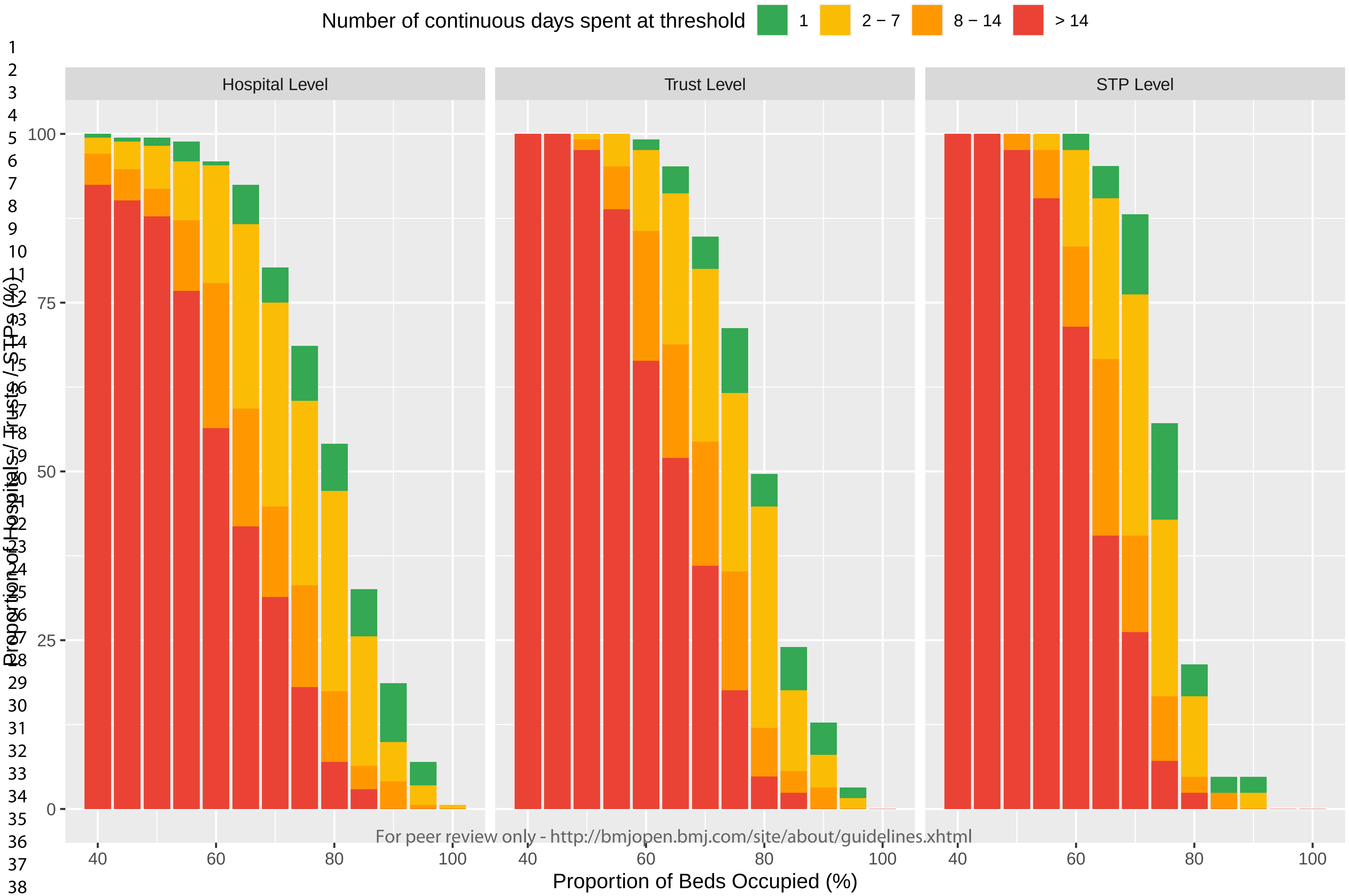

SFigure 10: Mechanical Ventilator Beds (Top) & General and Acute (Bottom) Occupancy (Based on Surge Capacities) Across England

Legend: SFigure 8A (Top) illustrates the proportion of STPs at different occupancy thresholds for surge mechanical ventilator bed capacity, across England. SFigure 8B (Bottom) illustrates the proportion of STPs at different occupancy thresholds for surge general and acute capacity, across England. The superimposed colours represent how long the trusts spent at each specific threshold.

For peer review only

SFigure 11: Ribbon Plots of STP Minimum and Maximum Occupancy

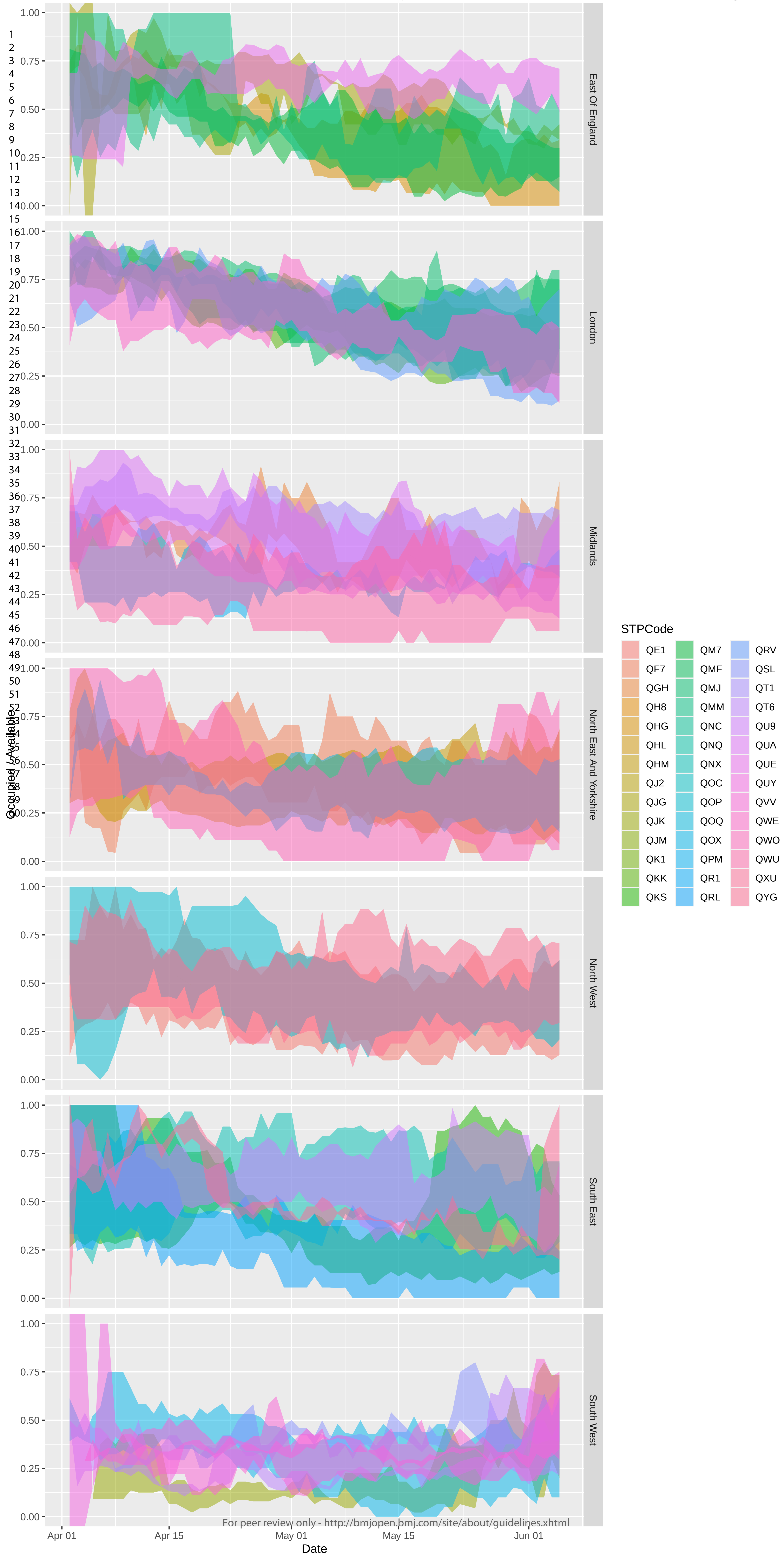

Supplementary Video 1

[See attached link for time-lapse of G&A bed capacity at the STP level across England]

Supplementary Video 2

[See attached link for time-lapse of mechanical ventilator bed capacity at the STP level across England]

Supplementary References

35 King's F. Sustainability and transformation plans (STPs) explained. 2017. https://www.kingsfund.org.uk/topics/integrated-care/sustainability-transformation-plans-explained.

36 Public Health England. COVID-19: investigation and initial clinical management of possible cases. 2020; published online Jan 10. https://www.gov.uk/government/publications/wuhan-novel-coronavirus-initial-investigation-of-possible-cases/investigation-and-initial-clinical-management-of-possible-cases-of-wuhan-novel-coronavirus-wn-cov-infection (accessed June 23, 2020).

37 Statistics. Statistics » Bed Availability and Occupancy Data – Overnight. https://www.england.nhs.uk/statistics/statistical-work-areas/bed-availability-and-occupancy/bed-data-overnight/. (accessed June 23, 2020).

38 Statistics. Statistics » Critical Care Bed Capacity and Urgent Operations Cancelled. https://www.england.nhs.uk/statistics/statistical-work-areas/critical-care-capacity (accessed June 23, 2020).

39 England NHS. NHS England » NHS steps up coronavirus fight with two more Nightingale Hospitals. https://www.england.nhs.uk/2020/04/nhs-steps-up-coronavirus-fight-with-two-more-nightingale-hospitals/ (accessed June 23, 2020).

40 England NHS. NHS England » NHS to build more Nightingale Hospitals, as London set for opening. https://www.england.nhs.uk/2020/04/nhs-to-build-more-nightingale-hospitals-as-london-set-for-opening/ (accessed June 23, 2020).

For peer review only

STROBE Statement—checklist of items that should be included in reports of observational studies

	Item No	Recommendation	Page No
**Title and abstract**	1	(*a*) Indicate the study's design with a commonly used term in the title or the abstract	1
		(*b*) Provide in the abstract an informative and balanced summary of what was done and what was found	2
**Introduction**			
Background/rationale	2	Explain the scientific background and rationale for the investigation being reported	4
Objectives	3	State specific objectives, including any prespecified hypotheses	4
**Methods**			
Study design	4	Present key elements of study design early in the paper	5
Setting	5	Describe the setting, locations, and relevant dates, including periods of recruitment, exposure, follow-up, and data collection	5
Participants	6	(*a*) *Cohort study*—Give the eligibility criteria, and the sources and methods of selection of participants. Describe methods of follow-up	5-6
		(*b*) *Cohort study*—For matched studies, give matching criteria and number of exposed and unexposed	NA
Variables	7	Clearly define all outcomes, exposures, predictors, potential confounders, and effect modifiers. Give diagnostic criteria, if applicable	6
Data sources/ measurement	8*	For each variable of interest, give sources of data and details of methods of assessment (measurement). Describe comparability of assessment methods if there is more than one group	*5-6*
Bias	9	Describe any efforts to address potential sources of bias	Supplementary material; quality control
Study size	10	Explain how the study size was arrived at	5
Quantitative variables	11	Explain how quantitative variables were handled in the analyses. If applicable, describe which groupings were chosen and why	5 & Supplementary material; quality control
Statistical methods	12	(*a*) Describe all statistical methods, including those used to control for confounding	6
		(*b*) Describe any methods used to examine subgroups and interactions	6 & Supplementary material; statistical analysis
		(*c*) Explain how missing data were addressed	Supplementary material; quality control
		(*d*) *Cohort study*—If applicable, explain how loss to follow-up was addressed	NA
		(*e*) Describe any sensitivity analyses	6

For peer review only - http://bmjopen.bmj.com/site/about/guidelines.xhtml

**Results**			
Participants	13*	(a) Report numbers of individuals at each stage of study—eg numbers potentially eligible, examined for eligibility, confirmed eligible, included in the study, completing follow-up, and analysed	7-15
		(b) Give reasons for non-participation at each stage	7
		(c) Consider use of a flow diagram	NA
Descriptive data	14*	(a) Give characteristics of study participants (eg demographic, clinical, social) and information on exposures and potential confounders	Supplementary table 3
		(b) Indicate number of participants with missing data for each variable of interest	Supplementary material; quality control
		(c) *Cohort study*—Summarise follow-up time (eg, average and total amount)	7-8
Outcome data	15*	*Cohort study*—Report numbers of outcome events or summary measures over time	*7-8*
Main results	16	(*a*) Give unadjusted estimates and, if applicable, confounder-adjusted estimates and their precision (eg, 95% confidence interval). Make clear which confounders were adjusted for and why they were included	NA
		(*b*) Report category boundaries when continuous variables were categorized	NA
		(*c*) If relevant, consider translating estimates of relative risk into absolute risk for a meaningful time period	NA
Other analyses	17	Report other analyses done—eg analyses of subgroups and interactions, and sensitivity analyses	Supplementary material; Critical care
**Discussion**			
Key results	18	Summarise key results with reference to study objectives	9
Limitations	19	Discuss limitations of the study, taking into account sources of potential bias or imprecision. Discuss both direction and magnitude of any potential bias	9-10
Interpretation	20	Give a cautious overall interpretation of results considering objectives, limitations, multiplicity of analyses, results from similar studies, and other relevant evidence	10-11
Generalisability	21	Discuss the generalisability (external validity) of the study results	11
**Other information**			
Funding	22	Give the source of funding and the role of the funders for the present study and, if applicable, for the original study on which the present article is based	No Funding

For peer review only

BMJ Open

A descriptive analysis of hospital bed capacity and usage across secondary healthcare providers in England during the first wave of the COVID-19 Pandemic

Journal:	*BMJ Open*
Manuscript ID	bmjopen-2020-042945.R2
Article Type:	Original research
Date Submitted by the Author:	08-Dec-2020
Complete List of Authors:	Mateen, Bilal Akhter; The Alan Turing Institute,<br>Wilde, Harrison; University of Warwick<br>Dennis, John; University of Exeter, The Institute of Biomedical & Clinical Science<br>Duncan, Andrew; Imperial College London<br>Thomas, Nick; University of Exeter, The Institute of Biomedical & Clinical Science<br>McGovern, Andrew; University of Exeter, The Institute of Biomedical & Clinical Science<br>Denaxas, S; UCL<br>Keeling, Matt; University of Warwick,<br>Vollmer, Sebastian; University of Warwick
**Primary Subject Heading**:	Health policy
Secondary Subject Heading:	Infectious diseases, Intensive care, Medical management, Public health, Health policy
Keywords:	COVID-19, INTENSIVE & CRITICAL CARE, Public health < INFECTIOUS DISEASES, Health policy < HEALTH SERVICES ADMINISTRATION & MANAGEMENT

Note: The following files were submitted by the author for peer review, but cannot be converted to PDF. You must view these files (e.g. movies) online.

G&A Time Lapse.mp4
Vent Time Lapse.mp4

SCHOLARONE™ Manuscripts

For peer review only - http://bmjopen.bmj.com/site/about/guidelines.xhtml

I, the Submitting Author has the right to grant and does grant on behalf of all authors of the Work (as defined in the below author licence), an exclusive licence and/or a non-exclusive licence for contributions from authors who are: i) UK Crown employees; ii) where BMJ has agreed a CC-BY licence shall apply, and/or iii) in accordance with the terms applicable for US Federal Government officers or employees acting as part of their official duties; on a worldwide, perpetual, irrevocable, royalty-free basis to BMJ Publishing Group Ltd ("BMJ") its licensees and where the relevant Journal is co-owned by BMJ to the co-owners of the Journal, to publish the Work in this journal and any other BMJ products and to exploit all rights, as set out in our licence.

The Submitting Author accepts and understands that any supply made under these terms is made by BMJ to the Submitting Author unless you are acting as an employee on behalf of your employer or a postgraduate student of an affiliated institution which is paying any applicable article publishing charge ("APC") for Open Access articles. Where the Submitting Author wishes to make the Work available on an Open Access basis (and intends to pay the relevant APC), the terms of reuse of such Open Access shall be governed by a Creative Commons licence – details of these licences and which Creative Commons licence will apply to this Work are set out in our licence referred to above.

Other than as permitted in any relevant BMJ Author's Self Archiving Policies, I confirm this Work has not been accepted for publication elsewhere, is not being considered for publication elsewhere and does not duplicate material already published. I confirm all authors consent to publication of this Work and authorise the granting of this licence.

For peer review only

A descriptive analysis of hospital bed capacity and usage across secondary healthcare providers in England during the first wave of the COVID-19 Pandemic

Bilal A. Mateen (MBBS)[1,2,3]*, Harrison Wilde (BSc)[4], John M. Dennis (PhD)[5], Andrew Duncan (PhD)[1,6], Nicholas J. Thomas (MRCP)[5,7] , Andrew P. McGovern (MD Res)[5,7], Spiros Denaxas (PhD)[1,8,9], Matt J Keeling (PhD)[10], Sebastian J. Vollmer (PhD)[1,4]

1 The Alan Turing Institute. Address: British Library, 96 Euston Road, London, NW1 2DB, UK
2 University of Warwick Medical School, Social Science and Systems in Health Unit. Address: Medical School Building, University of Warwick, Coventry, CV4 7AL, UK
3 Kings College Hospital NHS Foundation Trust. Address: Hambleden Wing, Kings College Hospital, Denmark Hill, London, SE5 9RS, UK
4 University of Warwick, Department of Statistics. Address: Department of Statistics, University of Warwick, Coventry, CV4 7AL, UK
5 University of Exeter Medical School. Address: Institute of Biomedical & Clinical Science, RILD Building, Royal Devon & Exeter Hospital, Barrack Road, Exeter EX2 5DW, UK
6 Imperial College London, Faculty of Natural Sciences. Address: Huxley Building, London, SW7 2AZ, UK
7 Royal Devon and Exeter NHS Foundation Trust, Diabetes and Endocrinology. Address: Exeter, Barrack Road, EX2 5DW
8 University College London, Institute of Health Informatics. Address: University College London, 222 Euston Rd, London NW1 2DA, UK
9 Health Data Research UK. Address: Gibbs Building, 215 Euston Road, London, NW1 2BE, UK
10 The Zeeman Institute for Systems Biology & Infectious Disease Epidemiology Research. Address: School of Life Sciences and Mathematics Institute, University of Warwick, Coventry, CV4 7AL, United Kingdom.

Author for Correspondence (*)
Dr. Bilal A. Mateen
Social Science and Systems in Health Unit
Warwick Medical School, University of Warwick
Coventry, CV4 7AL, UK
Email: bilal.mateen@nhs.net
Tel: +44 (0)24 7657 4880

Word Count: 3,899

For peer review only

Abstract

Objectives: In this study, we describe the pattern of bed occupancy across England during the peak of the first wave of the COVID-19 pandemic.

Design: Descriptive survey

Setting: All non-specialist secondary care providers in England, from March 27th to 5th June 2020

Participants: Acute (non-specialist) Trusts with a type 1 (i.e. 24 hours/day, consultant-led) accident and emergency department (n = 125), Nightingale (Field) Hospitals (n = 7), and independent sector secondary care providers (n = 195).

Main Outcome Measures: Two thresholds for 'safe occupancy' were utilized, 85% as per Royal College of Emergency Medicine and 92% as per NHS Improvement.

Results: At peak availability, there were 2711 additional beds compatible with mechanical ventilation across England, reflecting a 53% increase in capacity, and occupancy never exceeded 62%. A consequence of the repurposing of beds meant that at the trough, there were 8.7% (8,508) fewer general and acute (G&A) beds across England, but occupancy never exceeded 72%. The closest to (surge) capacity that any trust in England reached was 99.8% for general and acute beds. For beds compatible with mechanical ventilation there were 326 trust-days (3.7%) spent above 85% of surge capacity, and 154 trust-days (1.8%) spent above 92%. 23 trusts spent a cumulative 81 days at 100% saturation of their surge ventilator bed capacity (median number of days per trust = 1 [range: 1 to 17]). However, only 3 STPs (aggregates of geographically co-located trusts) reached 100% saturation of their mechanical ventilation beds.

Conclusions: Throughout the first wave of the pandemic, an adequate supply of all bed-types existed at a national level. Due to an unequal distribution of bed utilization, many trusts spent a significant period operating above 'safe-occupancy' thresholds, despite substantial capacity in geographically co-located trusts; a key operational issue to address in preparing for a potential second wave.

For peer review only

Article Summary

Strengths and Limitations of this Study

- The use of an administrative data that is collected by the statutory regulator as part of its legal mandate resulted in minimal missing information.
- Results are presented in the context of several geographic units of healthcare provision (i.e. hospital/site, trust, and STP-level), thus providing a much richer understanding of resource utilization that is less prone to the diluent effects of higher level geographies.
- The data represents a daily snapshot and therefore is unable to capture the nuances of the hospital throughput; in essence, both under and over-reporting of occupancy is possible using this method.
- The use of the occupancy thresholds reflects a limitation of our analysis, in that a proxy for adverse outcomes had to be utilized given that the necessary information was not readily available to directly explore the relationship between occupancy and patient-level outcome.
- The results of this study may not be generalizable to other countries given that it is specific to the UK National Health System infrastructure.

For peer review only

Introduction

The ability of hospitals to cope with large influxes of patients, either due to a pandemic illness or seasonal increases in respiratory disease exacerbations is in part dictated by the availability of beds.[1] Since 1987, when formal reporting of the number of hospital beds began in the UK, there has been a sustained decline in the number of available beds across the NHS.[2] In recent years, this issue has garnered more attention due to the annual 'winter bed crisis',[3,4] where the end of the calendar year heralds a surge in emergency admissions often resulting in hospitals operating well above quality and operational performance tipping points, i.e. 85% or 92% total bed occupancy.[5-7] The saturation of hospital beds is not only problematic through it impact on the ability of the workforce to deliver high-quality care,[8] but additionally the bottle-necking of the emergency care workflow has been shown to contribute to suboptimal outcomes for patients,[9] including increased numbers of healthcare-acquired infections,[10] and increased mortality.[11-13]

These concerns about the NHS' ability to cope with large influxes of patients took on a new level of significance in early 2020, when the World Health Organization (WHO) formally declared COVID-19 a pandemic illness, due to its virulence, and the magnitude of the disease's impact globally.[14] As early reports from China were published, it became apparent that a relatively large proportion of individuals who contracted COVID-19 required admission to hospital,[15] for example due to: new oxygen requirements, sepsis, acute respiratory distress syndrome (ARDS), and even multi-organ dysfunction (MODS). Forecasts of the potential number of people requiring hospital admission and mechanical ventilation across the UK suggested that the baseline capacity of the NHS would be insufficient.[16] In an effort to ensure sufficient capacity the British government instituted a series of policies, including facilitating the discharge of individuals who had been delayed due to non-medical reasons in an effort to unlock capacity,[17] cancelling all non-urgent clinical work, opening large field hospitals (i.e. the Nightingale hospitals),[18] and increasing mechanical ventilator availability for clinical areas repurposed to manage high care patients.[19]

The UK has started making significant strides towards rolling back its non-pharmacological interventions including. reopening schools, and planning for the discontinuation of shielding for vulnerable people,[20] signaling an end to the first wave of the pandemic.[21] Following these changes, there is the potential for a second wave of infections in the coming months. Understanding regional differences in hospital capacity is fundamental

For peer review only

to informing the UK's response to a second wave, as well as for elucidating how to safely wind down repurposed surge capacity such as operating theatres to allow other much needed clinical activity to restart.[22] However, other than a few isolated news reports of hospitals exceeding their ventilator capacity,[23] it is unclear how well the NHS as a whole managed to respond to the additional demand for beds over recent months. In this study, we sought to describe the pattern of bed occupancy in hospitals across England during the first wave of the COVID-19 pandemic.

For peer review only

Methods

Primary Data Source

Data were accessed from the daily situation reports ('SitReps', covering the previous 24 hours) provided to the Scientific Pandemic Influence Group on Modelling (SPI-M) by NHS England on behalf of all secondary care providers. All NHS acute care providers, independent sector care providers, and field hospitals in England submitting information to the daily situation reports were eligible for inclusion.

Study population

The data is presented in the context of several different units of secondary care provision: hospitals/sites, trusts, sustainability and transformation partnerships (STPs; aggregates of geographically co-located trusts), regions, and the whole of England (i.e. national), where each is an aggregate of the preceding unit (the structure of UK care providers is explained in the supplementary material).

Inclusion and Exclusion Criteria

Exclusions were applied at the trust level for NHS-specific care providers. Exclusion criteria were as follows: acute specialist trusts: women and/or children (n = 4), neurology & ophthalmology (n = 2), heart & lung (n = 3), orthopedic, burns & plastics (n = 4), cancer (n = 3). The remaining care providers were grouped into three categories and analyzed separately: 1) Acute (non-specialist) Trusts with a type 1 (i.e. 24 hours/day, consultant-led) accident and emergency department (n = 125); 2) Nightingale (Field) Hospitals (n = 7), and; 3) independent sector providers (n = 195).

Recruitment Period

Data was available from 27th March 2020 (the first available SitRep) to 5th June 2020 inclusive.

Recorded Information

The data specification comprised resource utilization and capacity-specific information, including the number of beds at each trust, stratified by several factors of interest, including acuity and COVID-19 ascertainment (further defined in supplementary material). Notably, beds were only recorded as being available if they were 'funded' (i.e. there was adequate staffing and resources for the bed to be occupied), so as to prevent counting of beds that could not accommodate a new patient. Bed acuity was organized into: general and acute (G&A), beds compatible

For peer review only

with non-mechanical ventilation, and beds compatible with mechanical ventilation. Occupancy is calculated based on the status of each bed at 08:00 each day, and then later separated by the proportion that had a positive COVID-19 test; there was no available information on the temporal relationship between admission and a positive test, and thus this data reflects some combination of community-acquired and nosocomial COVID-19.

Reporting fields changed on the 27th of April 2020, with several additional columns being added, which included specific fields for level 2 (HDU) and level 3 (ITU) beds. The impact of these changes is detailed in the supplementary material. However, one crucial outcome was that it became apparent the definition of critical care beds utilized prior to 27th April 2020 was not consistent with prior reporting practices of only including level 2 (HDU) and level 3 (ITU) beds,[24] as the newly reported values did not equal the simultaneously reported critical care values. As such, any results pertaining to critical care, HDU and ITU are reported separately in the supplementary material.

NHS England reports trust-level data, whereas we additionally attempted to disaggregate this information into the individual hospitals that the trusts comprise. Not all of the trusts were amenable to disaggregation from the trust-level data into independently reported sites in the available extracts, resulting in a final sample of 173 unique hospital sites, comprising 91.7% of the total number of ventilated beds and 81.4% of the general and acute beds when compared to trust level. The change in data reporting introduced on the 27th of April 2020 also resulted in variation in information capture; for data prior to the 27th of April, the results available reflect 89.6% of all mechanical ventilator beds and 86.9% of general and acute beds, where data from the 27th onwards the results reflect 93.0% of all mechanical ventilator beds but 77.0% of general and acute beds.

<u>Outcome</u>

The primary outcomes of interest were bed availability, and bed occupancy by patients with and without COVID-19, for each level of secondary care provision, i.e. hospital, trust, sustainability and transformation partnership (STP; aggregates of geographically co-located trusts). Different 'safe occupancy' thresholds were used to interpret the results; 85% as per the Royal College of Emergency Medicine, and 92% as per NHS Improvement. We also compared occupancy against baseline bed occupancy (see supplementary material for definitions), and 100% of surge capacity.

For peer review only

Statistical Analysis

We generated and reported descriptive summaries (e.g. medians, ranges, counts, proportions) of the data. We reported absolute numbers for hospital, trusts and STPs attaining specific occupancy thresholds. In light of the discordant critical care and HDU/ITU values, this analysis was handled and reported separately (see supplementary material). To capture the temporal aspect of the information available, the number of hospital-days, trust-days, and STP-days above hospital baseline capacity and surge capacities of 85%, 92% and 100% is also reported. Full details on the quality control procedures are reported in the supplementary material (SFigures 1 & SFigure 2). Details on aggregation and disaggregation of geographic information are provided in STable 1 & STable 2. Analyses were carried out in R,[25] ggplot2 package.[26] Maps were acquired from the UK's Office for National Statistics Open Geography Portal.[27]

Patient and Public Involvement

No patients were involved in the design of the study, interpretation of the results, or drafting of this manuscript.

For peer review only

Results

National Mobilization

During the first wave of the pandemic, the NHS repurposed general/acute beds into those suitable for higher acuity patients (i.e. HDU/ITU), and patients requiring mechanical ventilation. Available ventilated bed capacity peaked at an additional 2711 beds, a 53% increase from a baseline of 4123 beds. Ventilated beds occupancy never exceeded 62% of this capacity at a national level (Figure 1), and the proportion of occupied beds which contained patients with COVID-19 fluctuated between 30.4% and 76.0% over the course of the first wave, however there were notable regional differences in COVID-19 specific demand (Figure 2 & SFigure 3). Similar patterns were observed in critical care/HDU and ITU beds (SFigure 4). A consequence of the repurposing of beds for higher acuity patients there was a 8.7% reduction (n= 8,508) of general and acute beds from a baseline of 97,293 beds. There was a large reduction of the number of beds occupied by patients without COVID-19; 53,136 fewer beds were occupied (58.8% reduction) at the nidus compared to average occupancy from January to March 2020. Total bed occupancy never exceeded 72% nationally (Figure 1). Note: Data was relatively complete over the observation period (27th March to 5th June 2020), with no unavailable records for COVID-specific occupancy across G&A and mechanical ventilation compatible beds, and less than 10% for non-COVID/unoccupied beds (see supplementary material).

Occupancy relative to Baseline Capacity

Out of the 125 trusts (aggregates of hospitals), 3 trusts (2.4%) at some point during the first wave were operating above their baseline bed availability for general and acute beds (124 trust-days [1.4% of the total 8738 days at risk]; median number of days per trust = 36 days [range: 30 to 58]; SFigure 5). For beds compatible with mechanical ventilation, 87 trusts (69.6%) at some point during the first wave were operating above their baseline bed availability (2456 trust-days [28.1% of total at risk]; median number of days per trust = 24 days [range: 1 to 61]; SFigure 6). Similar results to that of mechanical ventilation compatible beds were seen for critical care / HDU and ITU bed occupancy (see supplementary material, SFigure 7 & SFigure 8).

Occupancy relative to Surge Capacity

Table 1 summarizes the number of hospitals, trusts and STPs operating above the pre-specified thresholds for 'safe occupancy', and details the duration (i.e. median number of days) that each spent above the designated thresholds.

For peer review only

Hospital-level Occupancy

Of the total 11,851 English hospital-days at risk over the study period, 494 hospital-days (4.17% of the total days at risk) were at or above 85% of G&A bed (surge) capacity, 110 hospital-days (0.92%) were at or above 92% of G&A bed (surge) capacity, and only 10 were spent at 100% of G&A surge capacity (Figure 3). Similarly, for beds compatible with mechanical ventilation there were 586 hospital-days (4.94%) spent above 85% of surge capacity, 320 hospital-days (2.70%) were spent above 92%, and 226 hospital-days (1.9%) were spent at 100% occupancy (see Figure 4). Summaries of the size and geographic locations of hospitals stratified by saturation are in STable 3.

Trust-level Bed Occupancy

Over the study period, there were 287 trust-days (3.3% of total days at risk) where general and acute bed occupancy exceeded 85% occupancy of surge capacity, and 57 trust-days (0.7%) were at or above 92% of bed (surge) capacity. The closest to capacity any trust in England reached was 99.8% for general and acute beds. However, for beds compatible with mechanical ventilation there were 326 trust-days (3.7%) spent above 85% of surge capacity, and 154 trust-days (1.8%) spent above 92%. 23 trusts reached 100% saturation of their mechanical ventilator bed capacity (Figure 5 & SFigure 9).

Table 1: The Number of Hospital/Trusts/STPs at each Occupancy Threshold for Different Bed Types

Bed Type	Organizational Unit	Occupancy Threshold					
		> 85%		> 92%		= 100%	
		Number (%)	Median number of days at or above threshold (range)	Number (%)	Median number of days at or above threshold (range)	Number (%)	Median number of days at or above threshold (range)
General & Acute	Hospital/Site (n = 173)	56 (32.4%)	6 (1 to 45]	19 (11.0%)	3 (1 to 19)	1 (0.6%)	10 days
	Trust (n = 125)	30 (24.0%)	5 (1 to 46)	14 (11.2%)	3 (1 to 13)	0 (0.0%)	-
	Sustainability and Transformation Partnership (n = 42)	2 (4.8%)	10 (3 to 17)	2 (4.8%)	1 (1 to 1)	0 (0.0%)	-
Mechanical Ventilation	Hospital/Site (n = 173)	91 (52.6%)	4 (1 to 48)	72 (41.6%)	3 (1 to 48)	52 (30.0%)	2 (1 to 48)
	Trust (n = 125)	58 (46.4%)	3 (1 to 27)	40 (32.0%)	2 (1 to 17)	23 (18.4%)	1 (1 to 17)

For peer review only

	**Sustainability and Transformation Partnership (n = 42)**	10 (23.8%)	2 (1 to 11)	5 (10.4%)	1 (1 to 6)	3 (7.1%)	1 (1 to 2)

Sustainability and Transformation Partnerships (STP) level Bed Occupancy

Across the 42 STPs (aggregates of geographically co-located trusts), there were 20 STP-days (0.7% of total days at risk) where general and acute bed occupancy exceeded 85% occupancy of surge capacity. The highest any STP reached for G&A bed occupancy was 92.7%. For beds compatible with mechanical ventilation, there were 35 STP-days (1.2%) where occupancy exceeded 85% occupancy of surge capacity, 11 STP-days (0.4%) in excess of 92% occupancy, and 4 STP-days (0.1%) at full occupancy (all of which were for STPs outside London: 1) Somerset, 2) Suffolk and North East Essex, and 3) Shropshire, Telford and Wrekin; SFigure 10). Figure 6 illustrates the number of STPs operating at each distinct occupancy threshold as a proportion of baseline and actual surge capacity. The full time-lapse for G&A (Supplementary Video 1) & ventilator bed (Supplementary Video 2) occupancy over the period of interest can be found in the supplementary material. A similar pattern was seen in the context of critical care / HDU & ITU beds across the STPs (SFigure 7 & SFigure 8).

For peer review only

<u>Field (Nightingale) Hospital Occupancy</u>

Of the reported bed capacity achievable through opening the Nightingale hospitals, at peak occupancy only 1.23% of the theoretical maximum were being utilized (Table 2). This equates to 618 bed days for patients with COVID-19 requiring mechanical ventilation, and 1483 bed days for all other types of intervention for patients with COVID-19 (i.e. oxygenation, non-invasive respiratory support, non-respiratory organ support, etc.).

Table 2: Field (Nightingale) Hospital Occupancy, and Capacity

<u>Nightingale Hospital Location</u>	<u>Number of Occupied Beds at peak*</u>	<u>Maximum number of Operational Beds*</u>	<u>Maximum Theoretical Capacity (Beds)</u>
London (Excel Centre)	66	112	4000
Manchester (Convention Centre)	47	72	1000
Birmingham (National Exhibition Centre)	0	0	2000
Bristol (University of West England)	NA	NA	1000
Washington (Centre of Excellence for Sustainable Advanced Manufacturing)	NA	NA	450

Harrogate (Convention Centre)	0	0	500
Exeter (Westpoint Arena)	NA	NA	200

Independent Sector Care Providers

Variations in reporting meant that the number of providers reporting each day varied, median 181 providers (range: 172 to 187). At peak occupancy, no more than 134 independent sector beds were occupied with patients who were confirmed COVID-19 positive. With regards to patients without COVID-19, at peak occupancy there were 1350 people in independent sector beds, representing a peak saturation of 18.7% (based on the total number of beds reported during contractual negotiations). In summary, there were 3360 bed days for patients with confirmed COVID-19 accommodated by the independent sector (86 mechanical ventilator bed days, 104 non-invasive ventilation bed days, and 3170 other bed days), and 53937 bed days for patients without COVID-19 (2771 mechanical ventilator bed days, 2046 non-invasive ventilation bed days, and 49120 other bed days), between 2nd April and 5th June across England.

For peer review only

Discussion

This national study of hospital-level bed occupancy provides unique and timely insight into the impact of COVID-19 on bed-specific resource utilization across an entire country. Our analysis suggests that the response of the NHS and British government to COVID-19 was sufficient to alleviate early concerns regarding the number of mechanical ventilators and critical care beds at a national level, however local variation in demand (i.e. regional variation in COVID-19 prevalence) still meant that many trusts reached 100% capacity for both. Moreover, examining occupancy in the context of different organizational units (i.e. trust-level versus STP-level), suggests that the higher-order networks (i.e. STPs) were not efficiently utilized to off-load disproportionately impacted trusts, as it was theoretically possible to have 95.1% fewer trust days at 100% mechanical ventilator bed capacity assuming load was better distributed. On the other hand, despite a reduction in overall capacity, G&A bed occupancy-levels relatively infrequently reached 'unsafe' levels, even at the individual hospital-level. This in part may explain why the field hospitals and independent sector care provider beds were never substantially utilized. Only a very small fraction of the theoretical maximum field hospital bed capacity was operationalized (1.23%). Similarly, despite signing a 14 week block contract with all of the major independent sector care providers valued at £235 million,[28] these beds too remained largely unoccupied, with less than 24% of the theoretical maximum beds days for established ventilators (i.e. not including the 1012 theatre-specific mechanical ventilators) having been used.

Context

Initial estimates suggested that an additional 30,000 mechanical ventilators would be necessary to accommodate the impact of the COVID-19 pandemic. These estimates were later updated to just 18,000 mechanical ventilators, from an estimated baseline of 8,000 across the UK.[29] It is difficult to determine the accuracy of these projections, as they were made in the absence of the impact of non-pharmacological interventions. However, the results of our study suggests that, at the population level, UK-based models of ventilator and bed resource utilization which integrated the impact of non-pharmacological interventions were actually remarkably accurate.[16,30] Arguably the most influential modelling study was that of the Imperial MRC group, wherein the authors clearly illustrate that with full 'lockdown' (i.e. the suite of non-pharmacological interventions that were eventually instituted), that critical care bed capacity would not be overwhelmed.[16] The nuance that this modelling study lacked was that it failed to explicitly incorporate the impact of unequal distribution of burden,

For peer review only

which manifested in our data as specific hospitals and trusts reaching full occupancy, despite the fact that at the national-level there was a substantial number of unoccupied beds.

This retrospective analysis also highlights some of the early incorrect assumptions made about the UK's baseline resource availability. For example, in contrast to ministerial statements suggesting that there were approximately 8,000 ventilators in the UK prior the pandemic,[29] our results identified only 4123 operational beds compatible with mechanical ventilation on the first day of reporting in England. Even after acknowledging that our value does not account for the devolved nations (Wales, Scotland, and Northern Ireland), it is unlikely that the initial figures reported by members of parliament truly reflected operational capacity, as that would suggest only 50% of such equipment was in England, despite it representing 84% of the UK population. Interestingly, the absolute increase in ventilator numbers due to the government incentives (e.g. the UK's Industrial Ventilator Challenge) is much more similar to our reported results.

Strengths and Limitations

There are several strengths to this study. For example, the use of an administrative (i.e. 'SitRep') data that is a statutory collection by NHS England, via a well-established reporting mechanism that has been exploited for research,[31] confers robustness to the data. One example of how this robustness manifested is, unlike other attempts to collect data at a national level to inform the COVID-19 response plan in the UK,[32] the degree of missingness in the data utilized in this study was minimal (see supplementary material). Moreover, in light of the unique access to the raw 'SitRep' data, we have been able to present our results not only at the trust-level, to which previous endeavors have been limited,[33] but rather have been able to present information at a much more granular layer (i.e. hospital/site-level) thus providing a much richer understanding of resource utilization that is less prone to the diluent effects of higher level geographies. Finally, a further strength of this study is the relative simplicity of the analysis; there are no complex statistical methods utilized as the descriptive summaries presented are sufficient to describe the experiences of nationalized (single-payer) health system in a high-income economy during the first wave of the COVID-19 pandemic.

Notably though, there are also several limitations to the dataset and our analysis. Principally we have no information on individual clinician and patient behaviour that will have inevitably influence these occupancy rates, and thus cannot comment on these factors. Secondly, there are limitations inherent to the SitRep data. In

For peer review only

particular, data were not available during February and early March during which some early 'bed mobilization' was likely carried out, and thus our observation period does not cover the entirety of the first wave (however, we believe it is unlikely that this undermines the major findings of this study). Moreover, changes introduced in 'SitRep' data collection half-way through the reporting period limited our ability to investigate critical care bed occupancy which was the third bed-specific potential concern identified by forecasting experts. The hospital-level results should also be interpreted with caution as it is an incomplete representation of the core trust-level information, and thus may not truly reflect the exact position of each organization; for example, the trust corresponding to the single site that achieved 100% G&A occupancy was never itself at 100% total occupancy. On a related note, the core weakness of the 'SitRep' data is that it presents data as a daily snapshot (at 08:00/8am), and therefore is unable to capture the nuances of the hospital throughput; in essence, both under and over-reporting of occupancy is possible using this method. As such, any marginal results where hospitals are only just over one of the 'safe occupancy-level' thresholds should be interpreted with caution as they could represent reporting artefacts. Moreover, the use of the occupancy thresholds reflects a limitation of our analysis, in that a proxy for adverse outcomes had to be utilized given that the necessary information was not readily available to directly explore the relationship between occupancy and patient-level outcome. Finally, the results of this study may not be generalizable to other countries given that it is specific to the UK National Health System infrastructure and reporting systems; for example, it is difficult to draw comparisons with other countries as UK specific factors such as reporting definitions are likely to mediate the hypothesized occupancy-mortality risk relationship, which will inevitably limit the ecological validity of these results in other geographical settings.

For peer review only

Implications for Policy Makers, and Clinicians

This study illustrates the potential for near real-time results reporting by which to determine the need for and effectiveness of government policies introduced to address resource utilization-specific issues as a consequence of the COVID-19 pandemic. For example, due to an unequal distribution of the resource utilization burden across England, many trusts spent a significant period of time operating above 'safe-occupancy' thresholds, despite the fact that in the vast majority of circumstances there was relief capacity in geographically co-located trusts (i.e. at the STP-level). Out of the 81 trust-days spent at 100% saturation of their mechanical ventilation beds (which pertains to 23 trusts reaching this threshold), on all but 5 days there was spare capacity at the corresponding STP level, which would have resulted in only 4 trusts reaching 100% saturation at any point (SFigure 11: STP min-

max occupancy plots). This reflects a key operational issue for policymakers to address in preparing for a potential second wave, and would have been identifiable if the SitRep data had been utilized for now-casting. Moreover, other policies for which these results may be relevant, include the creation of the Nightingale (field) hospitals, and independent sector network partnership. Our results suggest that the early investment and the creation of an operational field hospital and independent sector network may yield more overtly positive results in the winter, when G&A occupancy-levels regularly exceed 92%,[34] however, during the first wave of the pandemic they were under-utilized.

Conclusion

Using administrative data submitted by all secondary care organizations in England, we can conclude that at the national level there was an adequate supply of all bed-types throughout the first wave of the COVID-19 pandemic. However, the burden of need was not equally distributed, and thus in many cases local demand exceeded the supply of beds, especially where it concerned mechanical ventilation. Although several of the policies introduced by the government, both historical (i.e. STPs) and pandemic-specific (e.g. the independent sector block contract), could have potentially addressed this issue, there is evidence that these interventions were not optimally utilized. As such, we hope that this paper acts as exemplar for how routinely collected administrative data can be used to evaluate policy interventions, especially in the context of the COVID-19 pandemic, as well as highlighting the need for locally-relevant (in lieu of national or regional summaries), near-real-time information on service use for operational decision making.

For peer review only

Acknowledgements

BAM, SJV, and SD are supported by The Alan Turing Institute (EPSRC grant EP/N510129/). JMD is supported by an Independent Fellowship funded by Research England's Expanding Excellence in England (E3) fund. SJV is supported by the University of Warwick IAA funding. HW is supported by the Feuer International Scholarship in Artificial Intelligence. JMD, NJT, and APM are supported by the NIHR Exeter Clinical Research Facility. We thank NHS Improvement & NHS England for providing access to the SitRep data and Prof. Matt Keeling for establishing data access. Finally, we thank Dr. Bu'Hussain Hayee for sense-checking the final draft.

Contributors

Based on the CRediT Taxonomy, the authors of this study made contributions to this manuscript in the following ways: conceptualization (BAM & SJV); data curation (HW & SJV); methodology (HW, BAM & SJV); formal analysis (HW, BAM, JMD, SD, SJV); project administration & supervision (BAM, MK & SJV); visualization (HW, BAM, JMD, NJT, APM, AD, SJV); resources (SJV & MK); writing the original draft (BAM & HW); reviewing and editing the draft (all authors).

Declaration of interest

APM declares previous research funding from Eli Lilly and Company, Pfizer, and AstraZeneca. SJV declares funding from IQVIA. All other authors declare no competing interests.

Ethics & Governance

Data utilized in this study were made available through an agreement between the University of Warwick and the Scientific Pandemic Influence Group on Modelling (SPI-M), whom were acting on behalf of the British Government. The study was reviewed and approved by the Warwick BSREC (BSREC 120/19-20).

Data Availability Statement

Trust-level data will eventually be published by NHS England as a freely accessible data resource, but outputs have been delayed by the COVID-19 pandemic. For expedited or more granular access, requests will need to be made directly to NHS England (contact via england.dailysitrep@nhs.net). All code for this study is available on request.

For peer review only

Guarantor Statement

The corresponding (BAM) and the senior author (SJV) had full access to all data, and attest to the integrity of the analysis. The decision to submit for publication was agreed by all authors, and BAM and SJV act as guarantors of the work as presented.

Role of the Funding Source

There was no direct funding for this study. No funder was involved in the study design, analysis, interpretation of data; in the writing of the report; or in the decision to submit the paper for publication.

Dissemination

We will work with the relevant governmental groups to ensure that the patient organizations are informed of these results in a timely manner.

For peer review only

References

1 NHS hospital bed numbers. The King's Fund. 2020; published online March 26. Available at: https://www.kingsfund.org.uk/publications/nhs-hospital-bed-numbers (accessed June 21, 2020).

2 Appleby J. The hospital bed: on its way out? *BMJ* 2013; **346**: f1563.

3 The NHS this winter: looking beneath the national view | The Health Foundation. The Health Foundation. Available at: https://www.health.org.uk/news-and-comment/blogs/the-nhs-this-winter-looking-beneath-the-national-view (accessed June 21, 2020).

4 British Medical Association. Bed occupancy in the NHS - Pressures - BMA. The British Medical Association is the trade union and professional body for doctors in the UK. 2020; published online Jan 20. Available at: https://www.bma.org.uk/advice-and-support/nhs-delivery-and-workforce/pressures/bed-occupancy-in-the-nhs (accessed June 21, 2020).

5 Bagust A, Place M, Posnett JW. Dynamics of bed use in accommodating emergency admissions: stochastic simulation model. *BMJ* 1999; **319**: 155–8.

6 Fixing Emergency Department Staffing, Systems & Support to deliver excellent patient centered care. The Royal College of Emergency Medicine. Available at: https://www.rcem.ac.uk/docs/Policy/RCEM%20Vision%202020.pdf.

7 Improvement NHS. NHS review of winter 2017/18: annex. Available at: https://improvement.nhs.uk/documents/3202/Winter_review_annex_2.pdf (accessed June 22, 2020).

8 Against the odds: experiences from the NHS front line. RCP London. 2017; published online Feb 28. Available at: https://www.rcplondon.ac.uk/projects/outputs/against-odds-experiences-nhs-front-line (accessed June 23, 2020).

9 The implications of high bed occupancy rates on readmission rates in England | The Health Foundation. The Health Foundation. Available at: https://www.health.org.uk/publications/journal-articles/the-implications-of-high-bed-occupancy-rates-on-readmission- (accessed June 22, 2020).

10 Great Britain: National Audit Office. National Audit Office (NAO) - Department of Health:

For peer review only

Emergency Admissions to Hospital: Managing the Demand - HC 739: Managing the Demand, Department of Health. The Stationery Office, 2013.

11 Cowan RM, Trzeciak S. Clinical review: Emergency department overcrowding and the potential impact on the critically ill. *Crit Care* 2005; **9**: 291–5.

12 Sprivulis PC, Da Silva J-A, Jacobs IG, Frazer ARL, Jelinek GA. The association between hospital overcrowding and mortality among patients admitted via Western Australian emergency departments. *Med J Aust* 2006; **184**: 208–12.

13 Emergency and acute medical care in over 16s: service delivery and organisation. NICE. Available at: https://www.nice.org.uk/guidance/ng94/evidence/39.bed-occupancy-pdf-172397464704.

14 World Health Organization. Coronavirus Disease (COVID-19) Situation Reports. Available at:https://www.who.int/emergencies/diseases/novel-coronavirus-2019/situation-reports (accessed May 21, 2020).

15 Zhou F, Yu T, Du R, *et al.* Clinical course and risk factors for mortality of adult inpatients with COVID-19 in Wuhan, China: a retrospective cohort study. *Lancet* 2020; **395**: 1054–62.

16 Ferguson NM, Laydon D, Nedjati-Gilani G, *et al.* Impact of non-pharmaceutical interventions (NPIs) to reduce COVID-19 mortality and healthcare demand. 2020. *DOI* 2020;**10**:77482.

17 Stevens S. NHS-E and NHS-I LETTER ON NEXT STEPS ON NHS RESPONSE TO COVID-19. NHS England. Available at: https://www.england.nhs.uk/coronavirus/wp-content/uploads/sites/52/2020/03/urgent-next-steps-on-nhs-response-to-covid-19-letter-simon-stevens.pdf.

18 NHS England. New NHS nightingale hospital to fight coronavirus. Available at: https://www.england.nhs.uk/2020/03/new-nhs-nightingale-hospital-to-fight-coronavirus/ (accessed June 21, 2020).

19 UK Government Ventilator Challenge. 2020; published online April 22. Available at: https://www.gov.uk/government/topical-events/coronavirus-uk-government-ventilator-challenge/about (accessed June 21, 2020).

20 Department of Health, Care S. Plans to ease guidance for over 2 million shielding. 2020; published

For peer review only

online June 22. Available at: https://www.gov.uk/government/news/plans-to-ease-guidance-for-over-2-million-shielding (accessed June 23, 2020).

21 Prime Minister's Office, 10 Downing Street. Six people can meet outside under new measures to ease lockdown. 2020; published online May 28. Available at: https://www.gov.uk/government/news/pm-six-people-can-meet-outside-under-new-measures-to-ease-lockdown (accessed June 21, 2020).

22 Shock to the system: COVID-19's long-term impact on the NHS | The Health Foundation. The Health Foundation. Available at: https://www.health.org.uk/news-and-comment/blogs/shock-to-the-system-covid-19s-long-term-impact-on-the-nhs (accessed June 23, 2020).

23 Coronavirus: 'Critical incident' declared at Northwick Park Hospital in London after running out of intensive care beds [Internet]. ITV News. 2020 [cited 24 June 2020]. Available from: https://www.itv.com/news/2020-03-20/northwick-park-hospital-london-coronavirus-covid-19/

24 Monthly Trust SitRep (MSitRep) Critical Care Bed Capacity and Urgent Operations Cancelled. NHS England. Available at: https://www.england.nhs.uk/statistics/wp-content/uploads/sites/2/2019/11/MSitRep-Guidance-v1.09.pdf (accessed June 22, 2020).

25 R Core Team. R: A language and environment for statistical computing. R Foundation for Statistical Computing. Vienna, Austria. 2011.

26 Wickham H. ggplot2: Elegant Graphics for Data Analysis. Springer-Verlag New York. 2016.

27 Sustainability and Transformation Partnerships (April 2020) Boundaries. Available at: https://geoportal.statistics.gov.uk/datasets/sustainability-and-transformation-partnerships-april-2020-boundaries-en-buc?geometry=-43.431%2C48.021%2C39.142%2C57.304 (accessed June 23, 2020).

28 Permain N. COVID-19: Partnership working with the Independent Sector Providers and the Independent Healthcare Providers Network (IHPN). NHS England. Available at: https://www.england.nhs.uk/coronavirus/wp-content/uploads/sites/52/2020/03/ihpn-partnership-letter-25-march-2020.pdf (accessed June 22, 2020).

29 Balogun B. Coronavirus: Ventilator availability in the UK. 2020; published online June 3. Available at:

For peer review only

https://commonslibrary.parliament.uk/research-briefings/cbp-8904/ (accessed June 23, 2020).

30 Deasy J, Rocheteau E, Kohler K, *et al.* Forecasting ultra-early intensive care strain from COVID-19 in England. *medRxiv* 2020. https://www.medrxiv.org/content/medrxiv/early/2020/03/23/2020.03.19.20039057.full.pdf.

31 Friebel R, Fisher R, Deeny SR, Gardner T, Molloy A, Steventon A. The implications of high bed occupancy rates on readmission rates in England: A longitudinal study. *Health Policy* 2019; **123**: 765–72.

32 Dennis JM, Mateen BA, Sonabend R, Thomas NJ, Patel KA, Hattersley AT, Denaxas S, McGovern AP, Vollmer SJ. Type 2 Diabetes and COVID-19–Related Mortality in the Critical Care Setting: A National Cohort Study in England, March–July 2020. Diabetes Care. 2020 Oct 23.

33 The NHS this winter: looking beneath the national view | The Health Foundation. The Health Foundation. Available at: https://www.health.org.uk/news-and-comment/blogs/the-nhs-this-winter-looking-beneath-the-national-view (accessed June 23, 2020).

34 The NHS this winter: looking beneath the national view | The Health Foundation. The Health Foundation. Available at: https://www.health.org.uk/news-and-comment/blogs/the-nhs-this-winter-looking-beneath-the-national-view (accessed June 23, 2020).

For peer review only

Figure Legends

Figure 1: National and Regional Bed Occupancy

Legend: (Top) An epidemic curve showing the number of confirmed cases of COVID-19 across England, based on the date that the specimen was taken; raw data is available at: https://coronavirus.data.gov.uk/details/cases?areaType=nation&areaName=England. The superimposed highly-saturated solid line represents a smoothened function of the raw data, whereas the less saturated solid line represents the underlying raw values. The former is based on the ggplot loess fit for trend lines, using local poly-regression curve fitting. (Middle) Total capacity and occupancy status for general and acute (G&A) at the national level over the course of the first wave. (Bottom) Total capacity and occupancy status for bed compatible with mechanical ventilation at the national level.

Figure 2: Total bed occupancy in each of the 7 regions of England

Legend: (Top) COVID-19 specific occupancy in each of the 7 regions across England, for both general and acute (G&A; Left), as well as bed compatible with mechanical ventilation (Right). (Bottom) Total occupancy (COVID-19 positive and negative) in each of the 7 regions across England, for both general and acute (G&A; Left), as well as bed compatible with mechanical ventilation (Right). Note: the highly-saturated solid line represents a smoothened function of the raw data, whereas the less saturated solid line represents the underlying raw values. The former is based on the ggplot loess fit for trend lines, using local poly-regression curve fitting.

Figure 3: Hospital-level General & Acute Bed Occupancy (Based on Surge Capacities) Across England

Legend: The number of hospitals with general and acute bed occupancy in excess of the thresholds for 'safe and effective' functioning, i.e. 85% as defined by the Royal College of Emergency Medicine,[6] and 92% as defined by NHS Improvement and NHS England (green and yellow, respectively),[7] across England, from 26th March to June 5th. Note: all data was missing for the 29th of March and the 24th of May.

Figure 4: Hospital-level Ventilator Bed Occupancy (Based on Baseline Capacities) Across England

Legend: The number of hospitals with occupancy of mechanical ventilation beds in excess of the thresholds for 'safe and effective' functioning, i.e. 85% as defined by the Royal College of Emergency Medicine,[6] and 92% as defined by NHS Improvement and NHS England (green and yellow, respectively),[7] across England, from 1st April to June 5th. Note: all data was missing for the 24th of May.

For peer review only

Figure 5: Trust-Level Ventilator Bed Occupancy (Based on Surge Capacities) Across England

Legend: The number of trusts with occupancy of mechanical ventilation beds in excess of the thresholds for 'safe and effective' functioning, i.e. 85% as defined by the Royal College of Emergency Medicine,[6] and 92% as defined by NHS Improvement and NHS England (yellow and red, respectively),[7] across England, from March 26th to June 5th. Note: all data was missing for the 29th of March and the 24th of May. Several hospitals reported values consistent with 100% occupancy (black).

Figure 6: Peak Sustainability and Transformation Partnership (STP) Bed Occupancy Across England

Legend: The date on which general and acute bed occupancy (Left) and mechanical ventilator beds (Right) peaked, based on surge capacities at the Sustainability and Transformation Partnership (STP) level, across England. The geo-temporal pattern of peak occupancy clearly demonstrates that there was always residual G&A capacity at the STP level, and that all regions across England experienced similar levels of saturation. However, saturation of mechanical ventilator beds differed substantially by location.

For peer review only

Figure 1: Total Bed Occupancy and Daily Cases

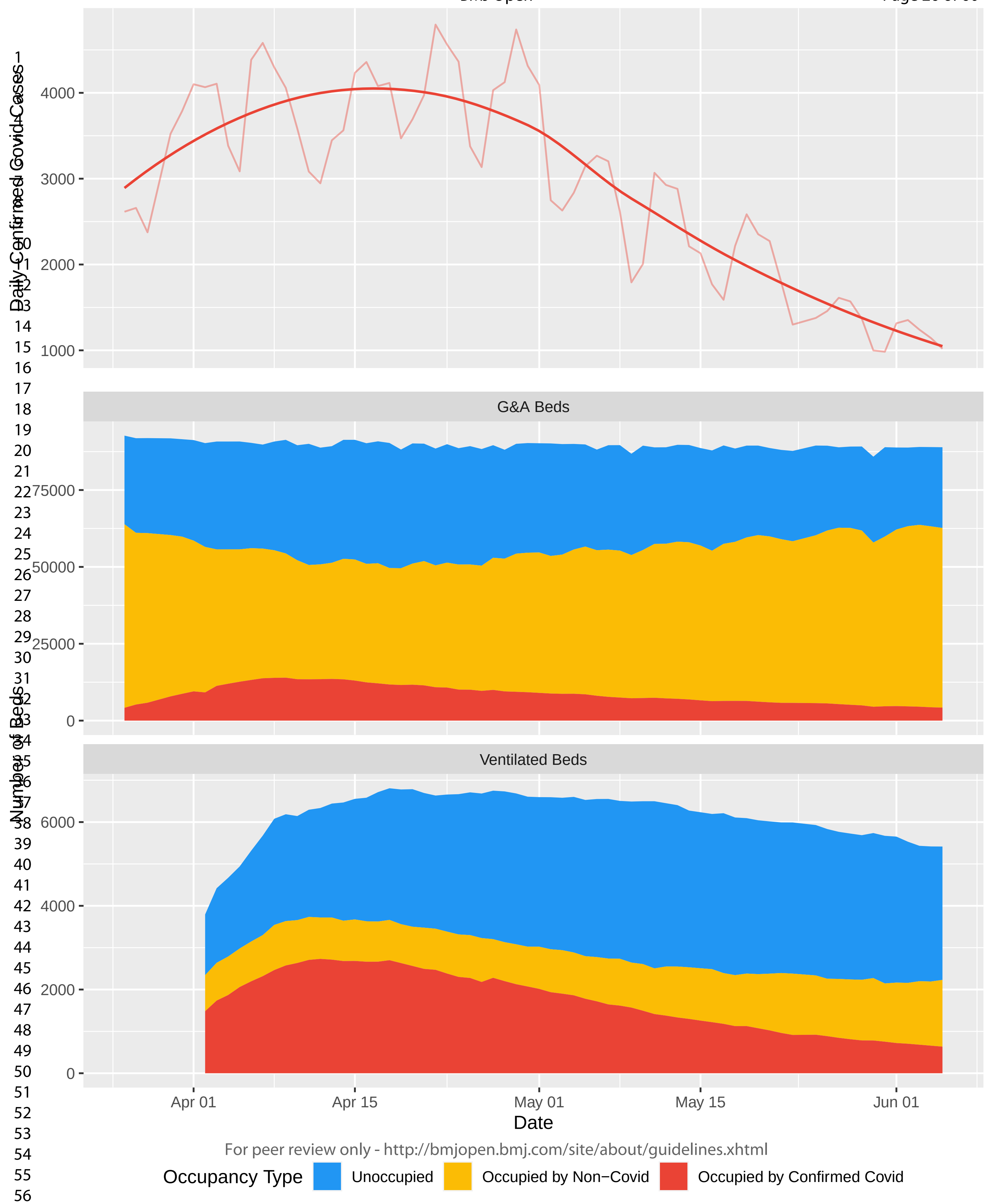

Figure 2: Percentage Bed Occupancy by Region

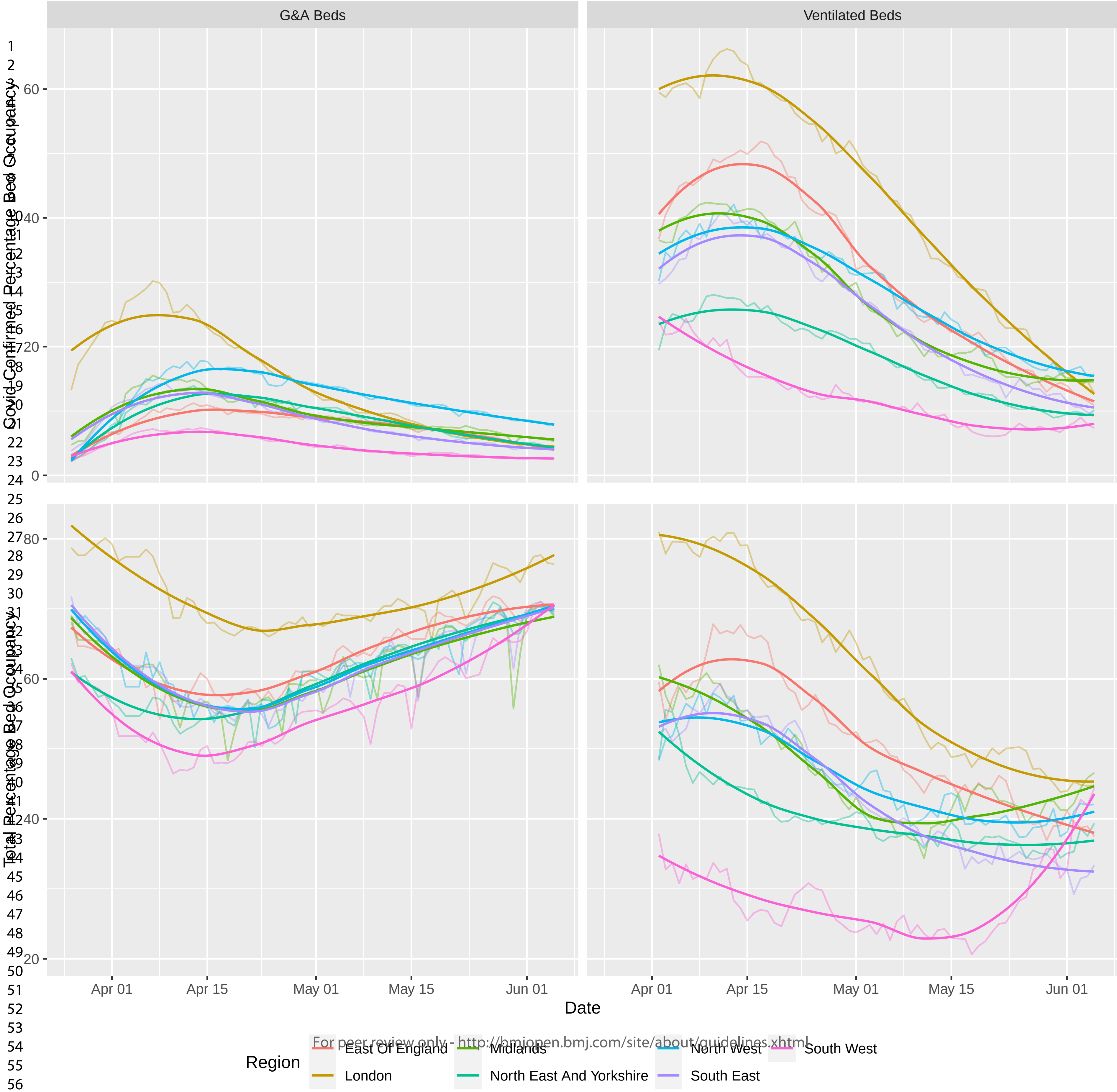

For peer review only - http://bmjopen.bmj.com/site/about/guidelines.xhtml

Figure 3: Number of Hospitals Operating Above Various G&A Bed Surge Capacity Thresholds

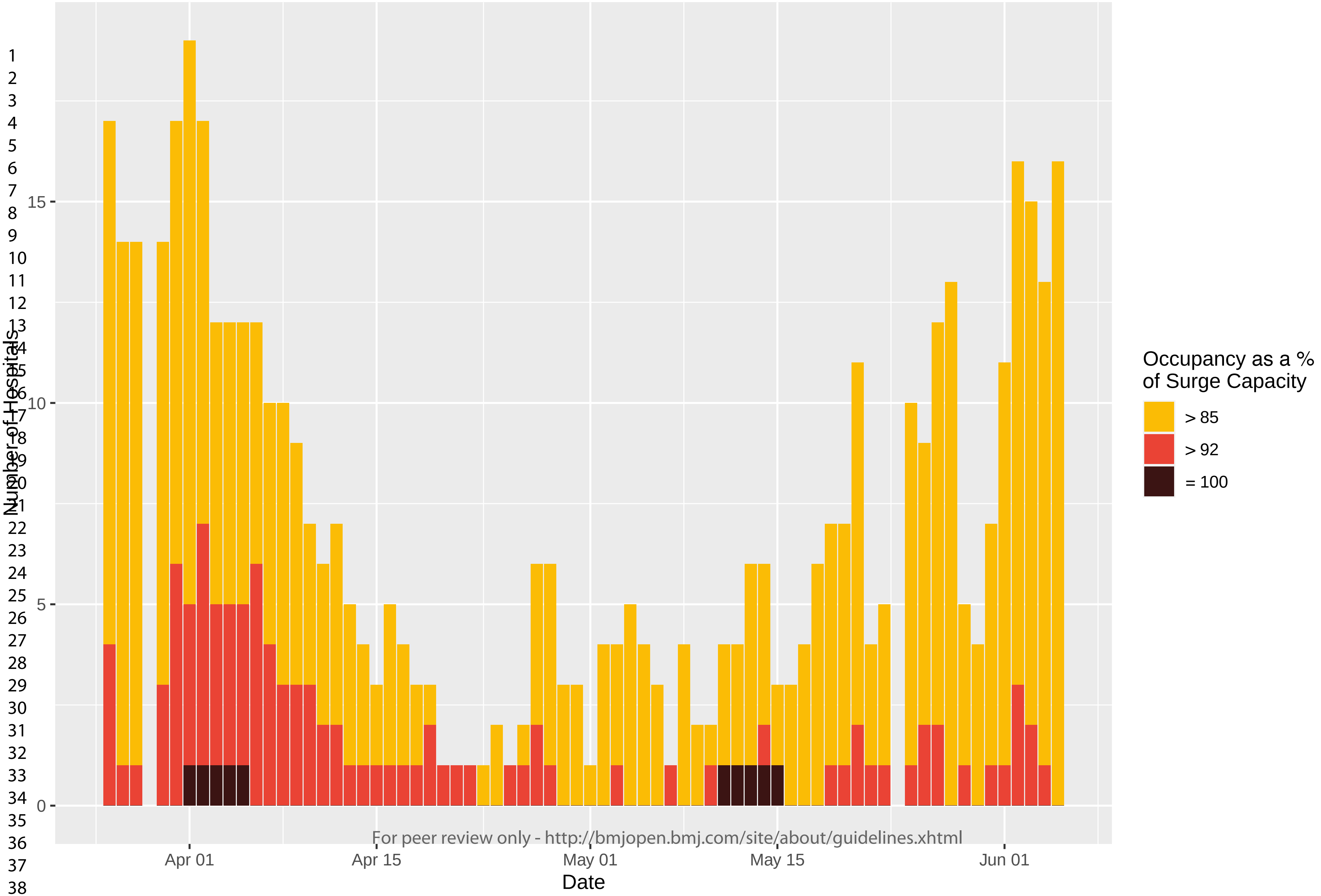

Figure 4: Number of Hospitals Operating Above Various Ventilated Bed Surge Capacity Thresholds

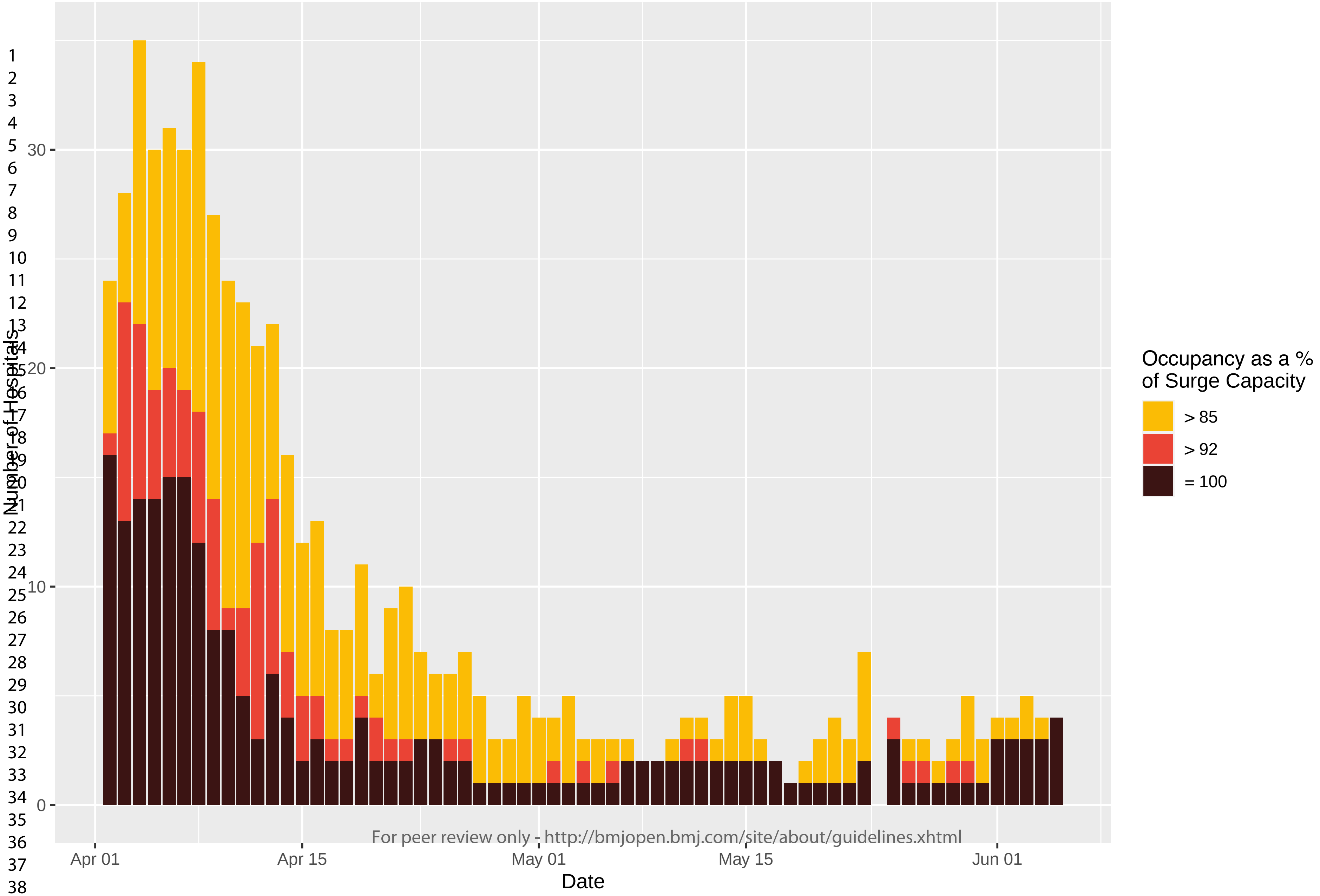

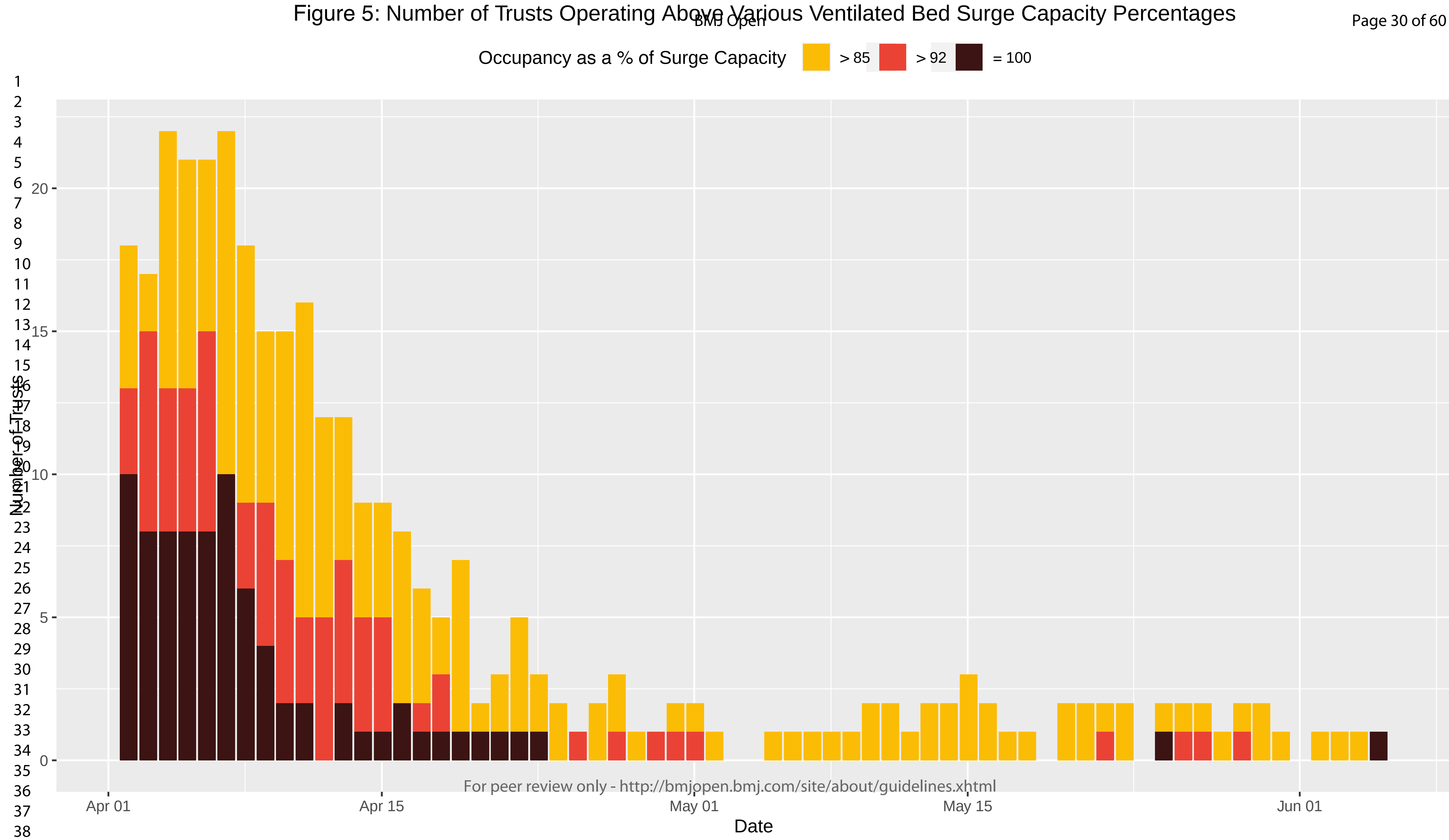
Figure 5: Number of Trusts Operating Above Various Ventilated Bed Surge Capacity Percentages
Occupancy as a % of Surge Capacity
> 85
> 92
= 100
Number of Trusts
0
5
10
15
20
Date
Apr 01
Apr 15
May 01
May 15
Jun 01

Figure 6: Peak STP Bed Occupancy Based on Surge Capacity

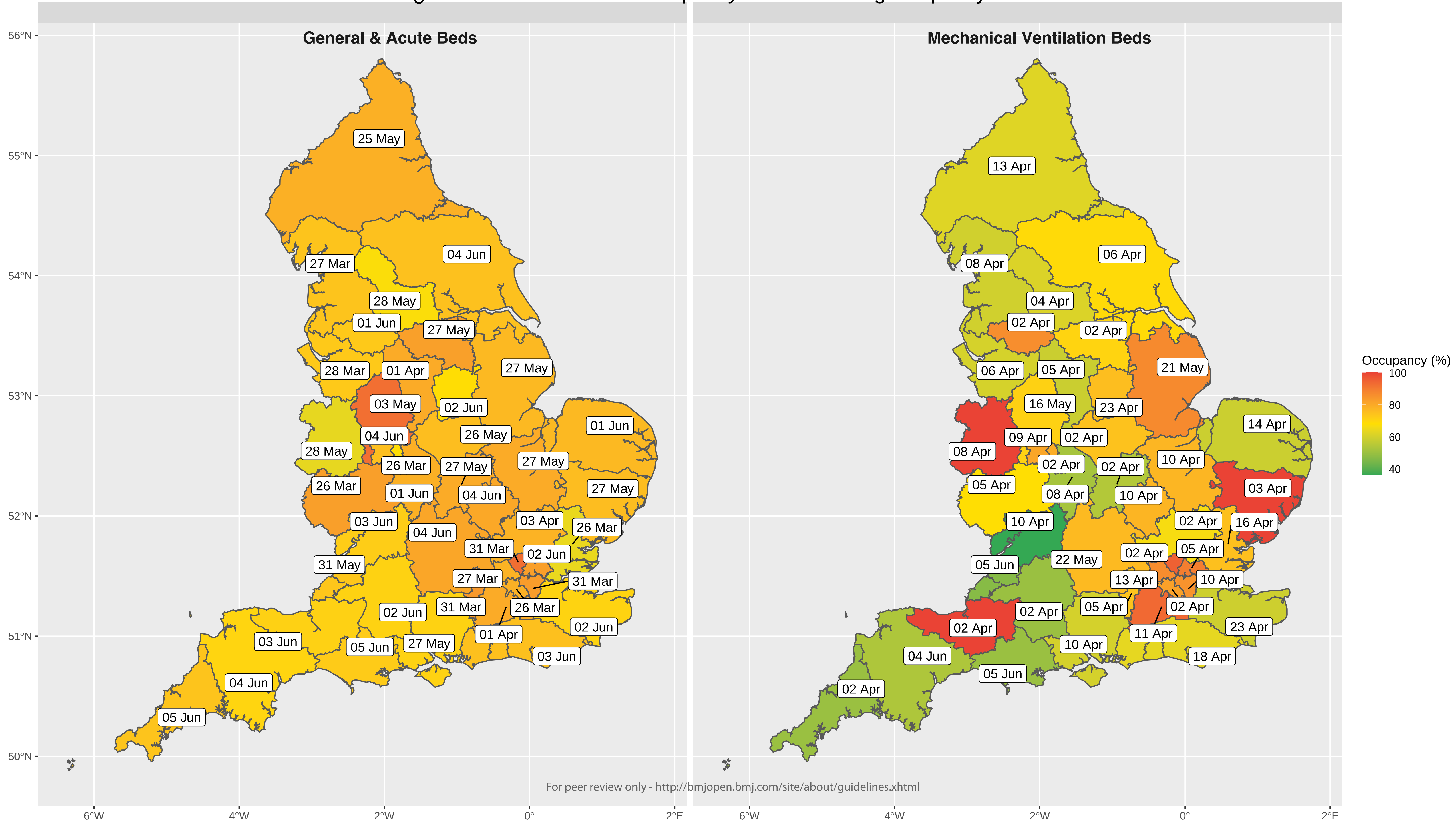

For peer review only - http://bmjopen.bmj.com/site/about/guidelines.xhtml

Supplementary Material

Supplementary Methods

Representativeness of Sample
Using the 2nd of May (randomly chosen) as an exemplar date, the non-specialist acute trusts to which we have restricted this survey represented 6,359 of the 6,866 beds (i.e. 92.6%) compatible with mechanical ventilation across England (comprising all institutions reporting to SitRep). Similarly, for all bed types, our sample represents 92.4% (i.e. 98,882 or the total 106,981 across England).

Organizational Units of Healthcare Provision
Although hospitals are relatively self-explanatory, the remaining units may require further context for readers unfamiliar with the organization structure utilized in the UK, as such, the following are brief summaries of the higher-order units of healthcare provision. Trusts are the core functional unit of hospital-based (i.e. secondary) care provision in England. They represent the first-level of aggregation above individual sites/hospitals, i.e. a trust is a collection of 1 or more geographically co-located hospitals which for specific administrative reasons operate as a single entity, although individual hospitals retain differing degrees of financial and operational autonomy depending on the specific trust structure. STPs are the aggregated unit of trusts, in combination with other units of healthcare provision, such as Clinical Commissioning Groups which administer the portion of the healthcare budget allocated to a specific geographic locale. There are 42 STPs each mapped to a specific health-geography 'footprint', and their express purpose was to deliver improvements pertaining to efficacy of services and integration of geographically co-located care providers.[35] The STPs are then mapped to 7 distinct geographical regions across England.

COVID-19 Status Recording
Data reported with reference to COVID-19 status, for example the number of general and acute beds occupied by individuals with the infection, refers to those whom had a confirmed positive result from a reverse transcriptase polymerase chain reaction (PCR) of nasopharyngeal and/or oropharyngeal swab.[36\] PCR was the only available testing method during the study period. Although national testing policy changed throughout the study period, all people for whom there was a suspicion of COVID-19 infection and who were admitted to hospital were tested, and potentially re-tested multiple times if the initial results were negative but clinical suspicion remained high (recorded as suspected COVID; for the purposes of the subsequent analysis 'confirmed' and 'suspected' were treated as one group due to the relatively small numbers reported for the latter).

Historical Baselines for Bed Availability
Baseline data comprised: 1) the average number of general and acute (G&A) beds available between January-March 2020, sourced from previously published routine situation report (SitRep) data;[37] 2) the number of critical care beds prior to the first reported case of COVID-19 in the UK, i.e. the value reported on the 30th January 2020 Critical Care SitRep,[38] and similarly to previous modelling studies was used for the baseline availabilities of HDU / ITU, critical care and ventilated beds;[30] 3) the maximum theoretical capacity for field hospitals was based on official government press releases;[39,40] and, 4) independent sector provider baseline capacity extracted from appendix 1 of the NHS England documentation confirming the 14 week block contract with the Independent Healthcare Providers Network.[28] Baselines were available for all of the trusts from the sources mentioned above and was propagated through into the STP and Regional datasets alongside aggregation of other values. Baseline bed numbers were not available for site level data. The choice of the period prior to the first wave of the pandemic instead of the historical baseline from 12 months prior was informed by two important piece of information: 1) the UK has experienced a gradual downward trend in bed numbers [1], and thus to be able to use the comparable period from 2019 we would have required an adjustment for that trend to produce a realistic baseline (there was a chance that we would have hypothesized there being more beds than were created after the first few weeks of mobilization by over-estimating the baseline number without this correction); 2) we deemed that use of the exact number of beds available at the time of operational planning (i.e. in February/early march) had greater ecological validity, as this was about reflecting the change from what we know was available rather than an abstracted version of what might have existed relative to similar periods in previous years.

Quality Control
All of the data was acquired through the daily site reports provided by NHS-Improvement & NHS England. These reports were loaded and appended sequentially with checks to ensure consistency in headings and data composition. The data spanned multiple sheets; these sheets were joined using Hospital, Trust and STP level

codes where appropriate. In some cases it was necessary to resort to using site names where no codes were present on the sheets containing hospital-level information regarding general and acute and critical care bed availability and occupancy. It was immediately apparent that extracting comprehensive site-level data from these records was non-trivial and for reasons discussed later, we maintain two datasets moving forward: one at site-level and one at trust-level that is used to aggregate to STP, Regional and Total figures as well.

Bed availability and total occupancy was recorded directly for G&A and critical care beds, alongside percentages of covid-confirmed occupants allowing for the calculation of a covid / non-covid / unoccupied breakdown for the G&A beds *only* (due to discrepancies in the definition of HDU / ITU and critical care beds, the percentage occupancy for critical care beds often resulted in impossible values of over 100%; it was decided to forego calculating a covid-breakdown for these beds due to how prolific these inconsistencies and issues were). For all of the other bed types, data was recorded in a different way. The number of covid positive patients (and in some cases covid-suspected patients), non-covid patients and the remaining unoccupied beds were recorded, allowing for total occupancy and availability to be calculated through simple transformation of these columns.

There are two key dates and several more minor milestones in the period we have data for (26th March to 5th June) where significant, non-trivial changes occurred in the site report structure and content. Prior to April 1st there was no information on bed availability beyond G&A and Critical Care beds; only the number of covid-positive patients were recorded for each type of bed. After the 1st of April, more granular bed availability was provided along with the means to work out the covid/non-covid breakdown of occupancy for Ventilated beds. From the 27th April onwards similar breakdowns and availability were recorded for HDU / ITU, IDU and most other types of bed at a site-level.

After loading in the data and accounting for the above described changes to its composition, the trust-level data used for the majority of our analysis had:

- 8.7% of Ventilated bed non-covid and unoccupied numbers missing across all records (no missing records for covid occupancy)
- No missing records for G&A, Critical care bed availability and occupancy
- No missing records for HDU / ITU after April 26th, otherwise 45.0%
- All other columns containing information regarding the hospital, trust, etc. were complete

Both datasets were filtered to remove children's hospitals, mental health hospitals and other sites / trusts that were not relevant to the analysis. STP linkage data was acquired via NHS Digital's library of public datasets source: (https://digital.nhs.uk/services/organisation-data-service/data-downloads/other-nhs-organisations) and augmented to include populations within STPs to facilitate our "beds-per-capita" figures (values were scrapped manually from the NHS England website, source: https://www.england.nhs.uk/integratedcare/stps/view-stps/). It was found that 7 trust codes were duplicated across 2 STPs; it was inappropriate to double count them so they were arbitrarily assigned to one of the STPs. The following table contains the STP and Trust code pairs that were chosen / removed from the linkage data to ensure a one-to-one mapping:

STable 1: STP and trust code pairs (for duplicated trusts)

Trust Code	STP Code (Assigned)	STP Code (Removed)
RDU	QRL	QNQ
RFS	QJ2	QF7
RK9	QJK	QT6
RMC	QOP	QHM
RNN	QE1	QHM
RVR	QXU	QWE
RVY	QYG	QE1

Additionally, due to some trust-level mergers that took place and missing data in the source, 4 updated STP-Trust pairs were manually added to the linkage data to facilitate their inclusion in the analysis (source:

For peer review only

https://www.england.nhs.uk/integratedcare/stps/view-stps/).

STable 2: Updated Trust-STP pairs (for merged trusts)

Trust Code	STP Code
RBA	QSL
RA3	QUY
RTP	QNX

Finally, it was found that two STPs spanned two regions. It was decided that QHM should fall under the North West region (all but one of its trusts are in that region) and QF7 should fall under the Midlands region (all but one of its trusts are in that region). The region definitions are inferred from the regions assigned to each trust in the site reports making up our primary dataset.

Despite our best efforts there were some missing values that persisted in critical columns outside of the key milestones mentioned in the section above. Moreover, in preparing the data it was noted that on several occasions there were substantial and improbable changes in the number of available beds that lasted 24 hours (even after allowing for the weekly trend of cyclical fluctuations in beds availability), prior to reversion to a value that fit the overall trend. These outliers follow from the reasonable assumption of the presence of data entry errors; it was decided that a cleaning rule should be applied to the data to avoid these seemingly impossible daily fluctuations and outliers.

First, a rolling median centred on each record was calculated using the 5 applicable days surrounding the record (smaller windows used at extremities of the data with correction not being possible at its absolute extremes). Missing values as well as values deemed to be outliers (a change greater than the 95th percentile of all differences between each record and the centred median spanning five days around it) were replaced with the aforementioned rolling median values. Highly improbable fluctuations were filtered out and missing values could be imputed in a robust way. This imputation and outlier detection process was applied to every applicable bed column spanning every type contained in the data. Only after this cleaning took place were other columns created through transformation, e.g. the number of available ventilated beds etc. The effect of cleaning the data is shown below in a before and after comparison, 4 trusts were chosen for their high initial volatility in G&A bed occupancy (See SFigure 1 & SFigure 2).

Statistical Analysis Notes
Temporalized values, i.e. hospital-days, were calculated by multiplying the absolute number of each functional unit for which data was available, and the number of days for which data is available for each.

After cleaning the data, two more key issues had to be dealt with in the trust-level and site-level datasets respectively:

1. Due to the aforementioned trust-level mergers, the composition of the data changed slightly throughout its duration. In an effort to achieve consistency, we merged and coalesced records prior to each mergers' appearance in the data to match their state post-merger. I.e. any rows corresponding to trusts that were eventually merged into some other trust were merged consistently throughout the dataset, even before this change actually took place. This was applied to records for the trusts RQ8, RDD and RAJ which were merged to fall under the single code RAJ on April 1st. This was also applied to RC9 and RC1 merged into RC9, and RA7 and RA3 merged into RA7: mergers that also occurred on April 1st and were reflected in the data shortly after.
2. It was observed that in one of the sheets relied upon for ventilated bed numbers, separate rows were included for both the sites and the corresponding trusts (given a "catch-all" label as their organisation type rather than "site"). In cases where only one hospital was associated with a trust, the numbers for that hospital were sometimes - inconsistently - recorded in the catch-all row rather than the site row as was done fairly consistently across all other situations. To achieve consistency without losing significant portions of the site-level data, we coalesced those rows where only one site was present and the catch all row contained numbers whilst the site row had zeroes or missing values. In order to achieve this, the organisation types of the two rows were swapped so that the catch-all row would be used in place of the site row, such that the site code and name was consistent throughout the entirety of the data.

Data Limitations

One persistent concern was the formulas by which bed occupancy proportions were generated. For example, the COVID-19 specific G&A bed percentage-occupancy was initially calculated as the sum of COVID-19 patients in IDU (infectious disease unit) beds and COVID-19 patients in "any other beds" divided by the total number of available G&A beds. This eventually changed to being the sum of the number of mechanical ventilated beds, non-invasive ventilated beds, oxygen-supporting beds and "any other beds" occupied by COVID patients minus the number of HDU / ITU beds occupied by confirmed COVID patients, all divided by the total number of available G&A beds. Whilst this is not in-and-of-itself problematic, the nature of the "any other beds" item was deemed concerning by the authors.

To understand the aforementioned concern, we first need to explain the data specification in more detail. It was noted that columns of the form "Number of Covid-19 confirmed patients in … beds at 0800" did not seem to contain values consistent with "Number of … beds available, as at 08:00 (COVID)", which we expected to have mirrored values. Importantly, the latter set of columns did not contain an "any other bed" column. As such, the formula used by NHS-E in the above calculation of G&A bed proportions drew the "any other beds" value from the first set of data, whereas all of the other information was drawn from the latter columns as they were internally consistent. We acknowledge that the use of this formula could have introduced an error of unknown magnitude or direction (as the two versions of data reporting were not consistent). Similar issues were seen with the independent sector data as well.

For peer review only

SFigure 1: G&A Bed Availability Across the Most Volatile Trusts in Terms of G&A Bed Occupancy (Pre−Correction)

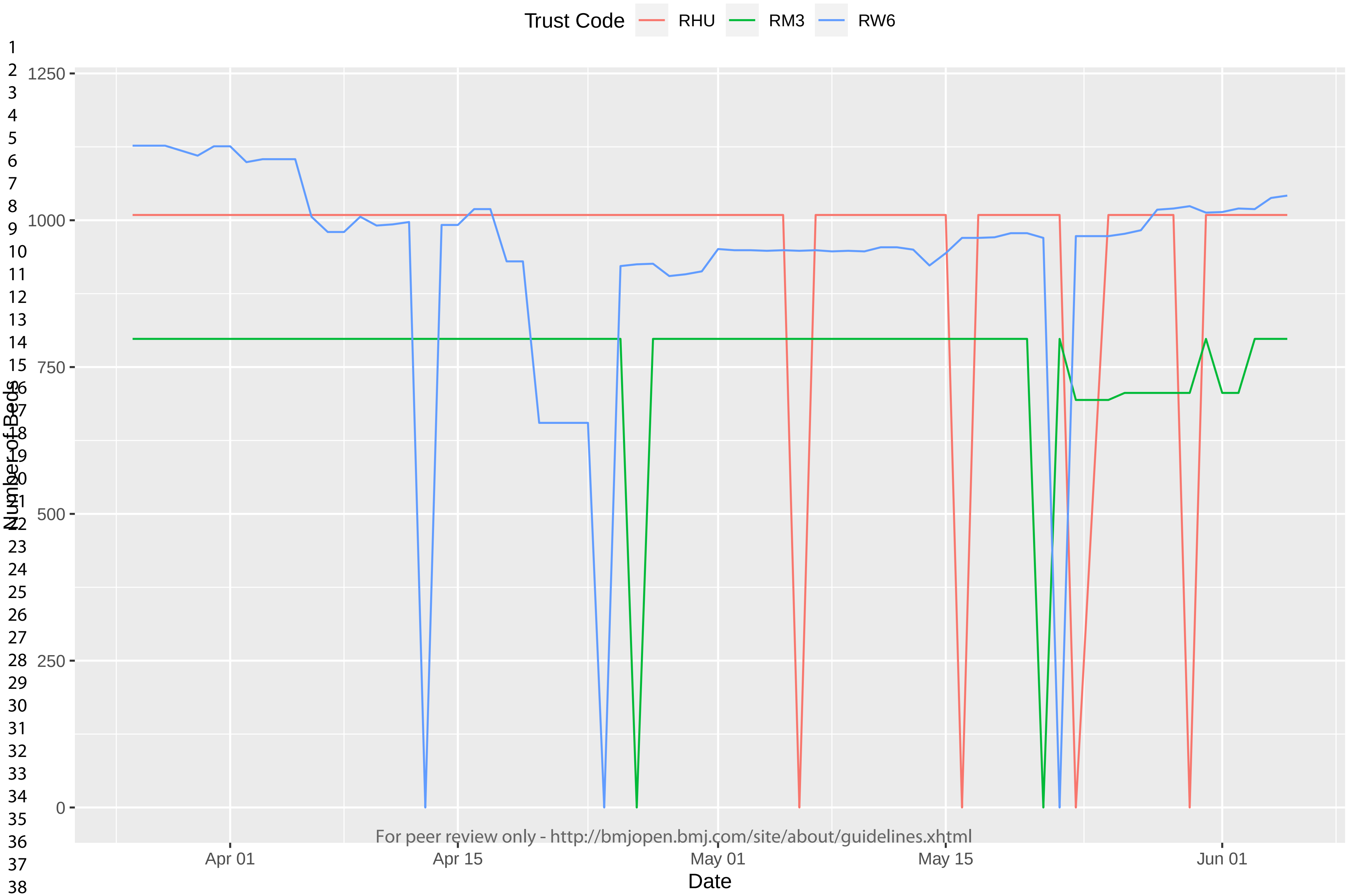

SFigure 2: G&A Bed Availability Across the Most Volatile Trusts in Terms of G&A Bed Occupancy (Post−Correction)

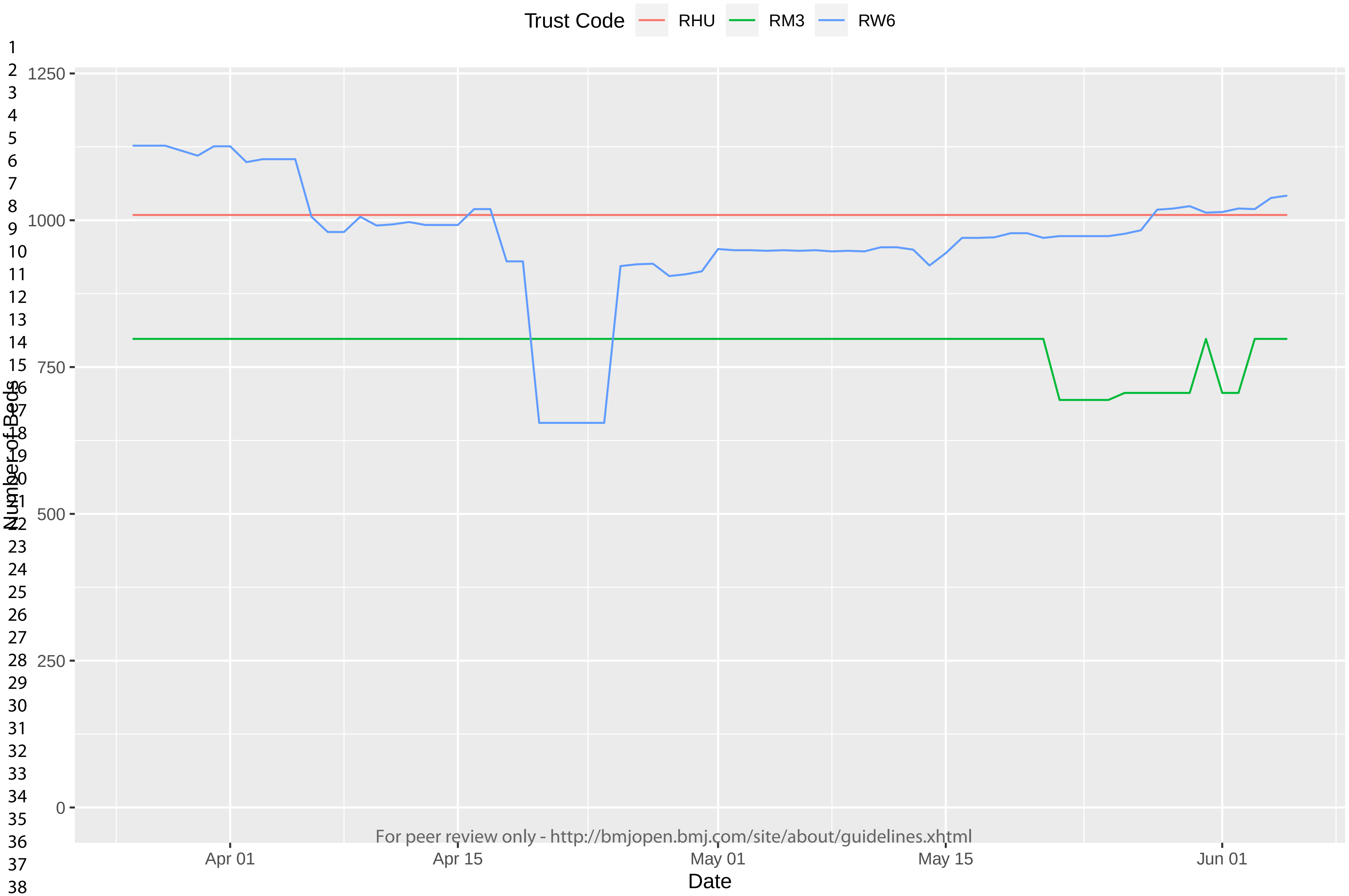

For peer review only - http://bmjopen.bmj.com/site/about/guidelines.xhtml

Supplementary Results

STable 3: Descriptive Summaries of the Size and Geographic Locations of Hospitals Stratified by the Peak Occupancy Achieved

	**Hospitals reaching 100% saturation of mechanical ventilation beds (n = 51*)**	**Hospitals reaching >92%, but not 100% saturation of mechanical ventilation bed (n = 20)**	**Hospitals reaching >85%, but not 92% saturation of mechanical ventilation bed (n = 19)**	**All other Hospitals (n = 77**)**
**Peak G&A bed capacity (Median [Range])**	438 [197 - 1012]	484 [256 - 841]	459 [253 - 910]	558 [44 - 1499]
**Peak mechanical ventilation compatible bed capacity (restricted to April 2nd onwards) (Median [Range])**	29 [7 - 77]	49 [18 - 141]	33 [15 - 153]	40 [7 - 159]
**Peak HDU/ITU bed capacity (restricted to April 27th onwards) (Median [Range])**	29 [0 - 99]	45 [17 - 161]	37 [14 - 157]	40 [6 - 152]
Location				
England (n = 167)	***51 (30.5%)***	***20 (12.0%)***	***19 (11.4%)***	***77 (46.1%)***
**London (n = 27)**	6 (22.2%)	11 (40.7%)	7 (25.9%)	3 (11.1%)
**Midlands (n = 27)**	11 (40.7%)	3 (11.1%)	1 (3.7%)	12 (44.4%)
**East of England (n = 20)**	7 (35.0%)	2 (10.0%)	1 (5.0%)	10 (50.0%)
**South West (n = 17)**	3 (17.6%)	0 (0.0%)	0 (0.0%)	14 (82.3%)
**South East (n = 24)**	6 (25.0%)	2 (8.3%)	3 (12.5%)	13 (54.2%)
**North East and Yorkshire (n = 29)**	8 (27.6%)	1 (3.4%)	4 (13.8%)	16 (65.5%)
**North West (n = 23)**	10 (43.5%)	1 (4.3%)	3 (13.0%)	9 (39.1%)

* One hospital is excluded from this n and the subsequent calculations as it does not have any information regarding G&A beds, despite reaching 100% capacity for mechanical ventilator bed capacity.
** 5 hospitals were excluded from this table as they had no ventilated beds at any time, or no data was available for their ventilated bed capacity in the dataset.

For peer review only

SFigure 3: Regional Bed Occupancy Across England, Stratified by COVID-19 Status

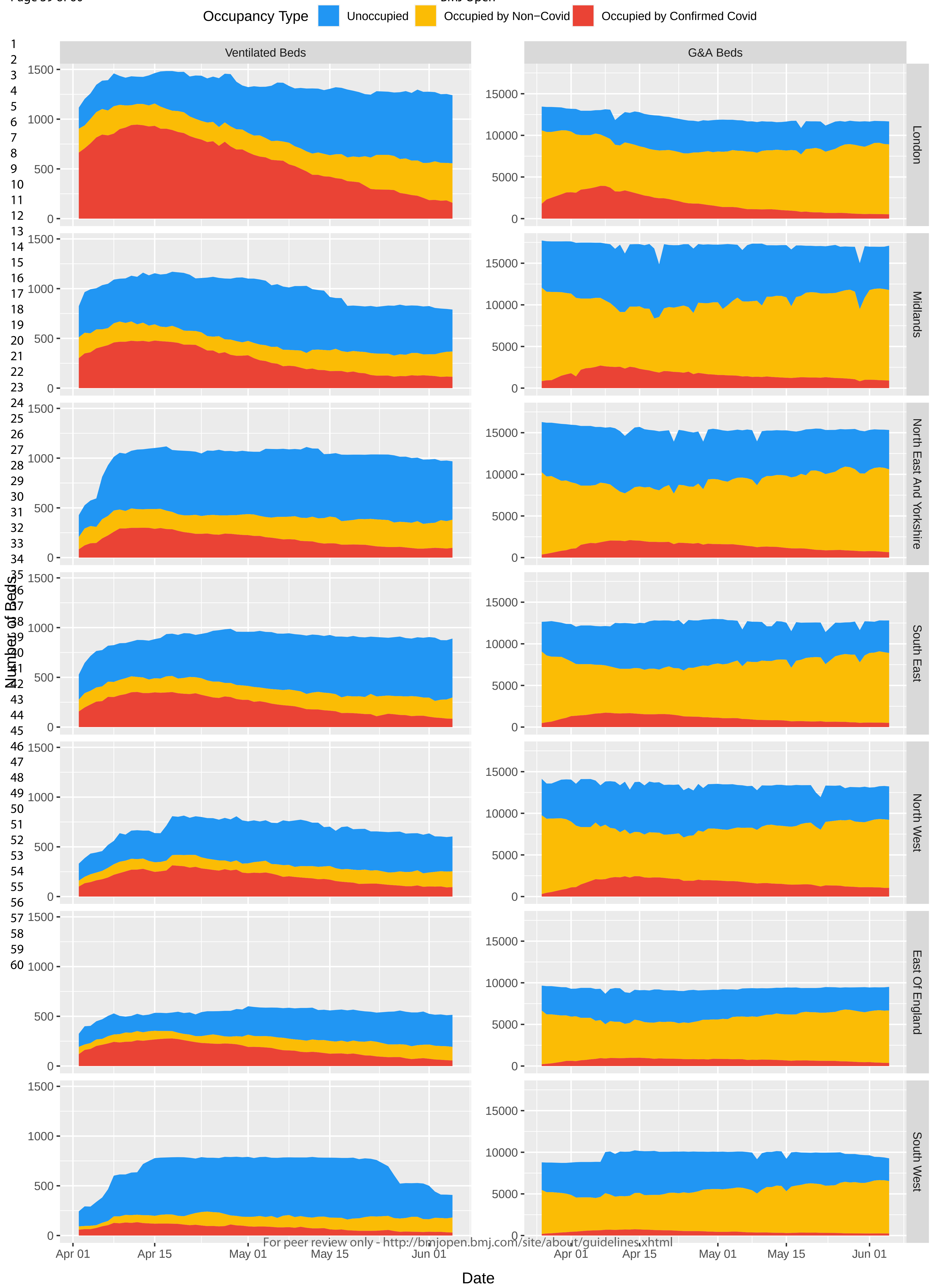

SFigure 3: Bed Occupancy Across England by geographical region, stratified by COVID-19 Status

Legend: SFigure 3A (Left) illustrates the time-varying trends in mechanical ventilator bed capacity and occupancy across the 7 regions of England, from March 27th to June 5th; note that availability information is only present from 1st April onwards. SFigure 3B (Right) illustrates general and acute bed capacity and occupancy across the 7 regions of England, from 1st April to 5th June. Occupancy in both figures is stratified by whether the individual in the bed has a positive COVID-19 test or not).
Critical Care Beds

For peer review only

Critical Care Beds
In the context of surge capacity, at the site-level, 1558 hospital days (13.1%; median number of days per hospital = 10 [range: 1 to 65]) were at or above 85% of capacity, which corresponds to 120 hospitals spending at least 1 day at, or above, the aforementioned threshold. 948 hospital days (8.0%; median number of days per hospital = 6 [range: 1 to 51]) were spent above 92%, representing 102 hospitals. And 88 (50.9%) hospitals reached 100% capacity, representing 640 hospital days at saturation (median number of days per hospital = 5 [range: 1 to 51])). At the trust-level, 965 trust days (11.0%; median number of days per trust = 8 [range: 1 to 56]) were at or above 85% of capacity, representing 80 trusts. 567 trust days (6.5%; median number of days per trust = 5 [range: 1 to 47]) were spent above 92%, representing 64 trusts. And 47 (37.6%) trusts reached 100% capacity, representing 339 trust days at saturation (median number of days per trust = 5 [range: 1 to 21])). At the STP-level, 138 STP days (median number of days per STP = 5 [range: 1 to 43]) were at or above 85% of capacity, representing 18 STPs. 74 STP days (median number of days per STP = 2 [range: 1 to 26]) were spent above 92%, representing 14 STPs. And 6 STPs reached 100% capacity, representing 34 STP days at saturation (median number of days per STP = 3 [range: 1 to 19])). See SFigure 4 for a visual summary of these results. See SFigure 5 for the aggregate occupancy, stratified by COVID-19 status at the regional level.

In the context of baseline capacity, at the trust-level, 2620 trust days (22.1%; median number of days per trust = 27 [range: 1 to 69]) were at or above 100% capacity, which corresponds to 92 trusts spending at least 1 day at, or above, their-pre-pandemic baseline. 230 trusts days (median number of days per trust = 9 [range: 1 to 49]) were at or above 200% capacity, which corresponds to 19 trusts spending at least 1 day more than 100% above their-pre-pandemic baseline. At the STP-level, 620 STP days (median number of days per STP = 24 [range: 1 to 63]) were at an occupancy-level above 100% of baseline availability, which corresponds to 27 STPs spending at least 1 day at, or above, their-pre-pandemic baseline. 44 STP days (median number of days per STP = 14 [range: 10 to 20]) were at an occupancy-level above 200% of baseline availability which corresponds to 3 STPs spending at least 1 day more than 100% above their-pre-pandemic baseline. See SFigure 8 for a visual summary.

HDU/ITU Beds
The following results should be interpreted in the context of the date range available, i.e. data is only present after the 27th of April. Thus, the results are likely a significant underestimation of peak occupancy as, in retrospect, the peak number of cases and fatalities in the UK were near the beginning of April.

In the context of surge capacity, at the site-level, 315 hospital days (2.7%; median number of days per hospital = 2 [range: 1 to 39]) were at or above 85% of capacity, which corresponds to 59 hospitals spending at least 1 day at, or above, the aforementioned threshold. 216 hospital days (1.8%; median number of days per hospital = 1 [range: 1 to 39]) were spent above 92%, representing 45 hospitals. And 40 hospitals reached 100% capacity, representing 192 hospital days at saturation (median number of days per hospital = 1 [range: 1 to 39])). At the trust-level, 192 trust days (median number of days per trust = 3 [range: 1 to 39]) were at or above 85% of capacity, representing 36 trusts. 122 trust days (median number of days per trust = 2 [range: 1 to 39]) were spent above 92%, representing 24 trusts. And 19 trusts reached 100% capacity, representing 106 trust days at saturation (median number of days per trust = 2 [range: 1 to 39])). At the STP-level, 138 STP days (median number of days per STP = 3 [range: 1 to 43]) were at or above 85% of capacity, representing 18 STPs. 74 STP days (median number of days per STP= 2 [range: 1 to 26]) were spent above 92%, representing 14 STPs. And 6 STPs reached 100% capacity, representing 34 STP days at saturation (median number of days per STP = 3 [range: 1 to 19])). See SFigure 5 for a visual summary.

For peer review only

SFigure 4a: Number of Hospitals/Trusts/STPs at Varying Critical Care Bed Occupancy Levels Compared to Surge Capacity

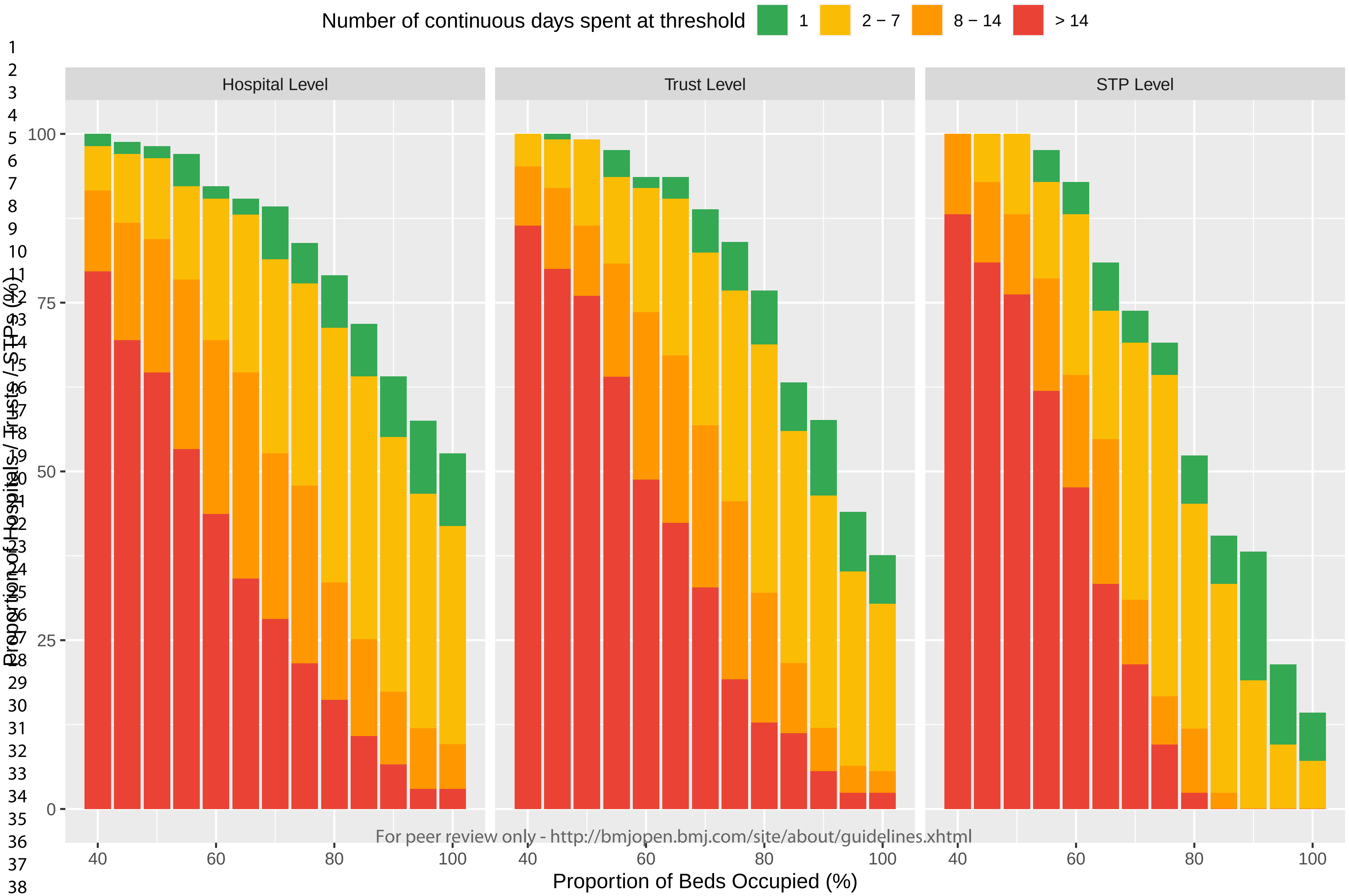

For peer review only - http://bmjopen.bmj.com/site/about/guidelines.xhtml

SFigure 4b: Number of Hospitals/Trusts/STPs at Varying HDU/ITU Bed Occupancy Levels Compared to Surge Capacity

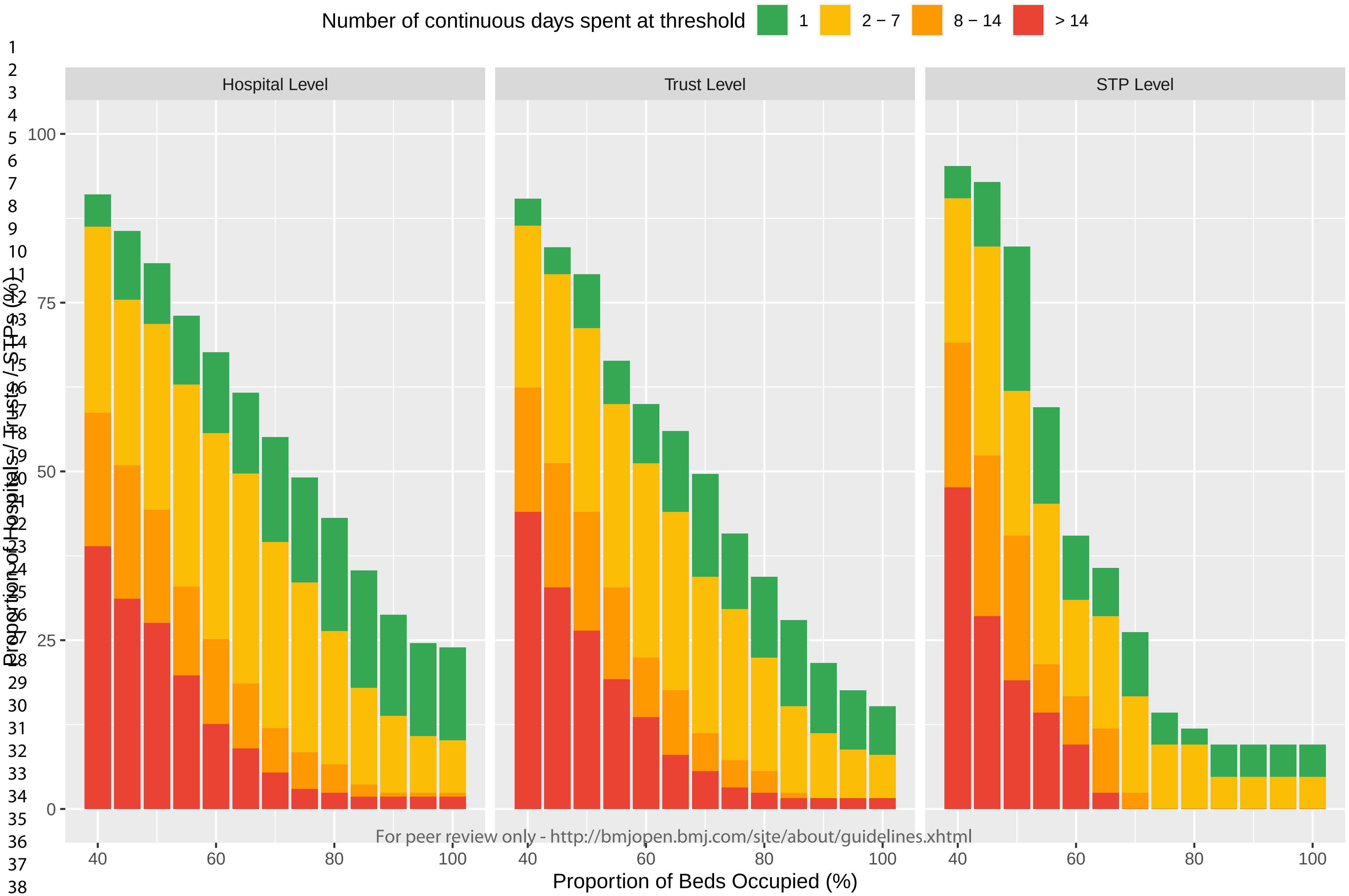

SFigure 4: Critical Care (Top) & HDU/ITU (Bottom) Occupancy (Based on Surge Capacities) Across England

Legend: SFigure 4A (Top) illustrates the proportion of hospitals/trusts/STPs at different occupancy thresholds for surge critical care bed capacity, across England, from April 1st to June 5th. SFigure 4B (Bottom) illustrates the proportion of hospitals/trusts/STPs at different occupancy thresholds for surge critical care bed capacity, across England, from April 1st to June 5th. The superimposed colours represent how long the trusts spent at each specific threshold.

For peer review only

SFigure 5: Number of Trusts/STPs at Varying G&A Bed Occupancy Levels Compared to Baseline Capacity

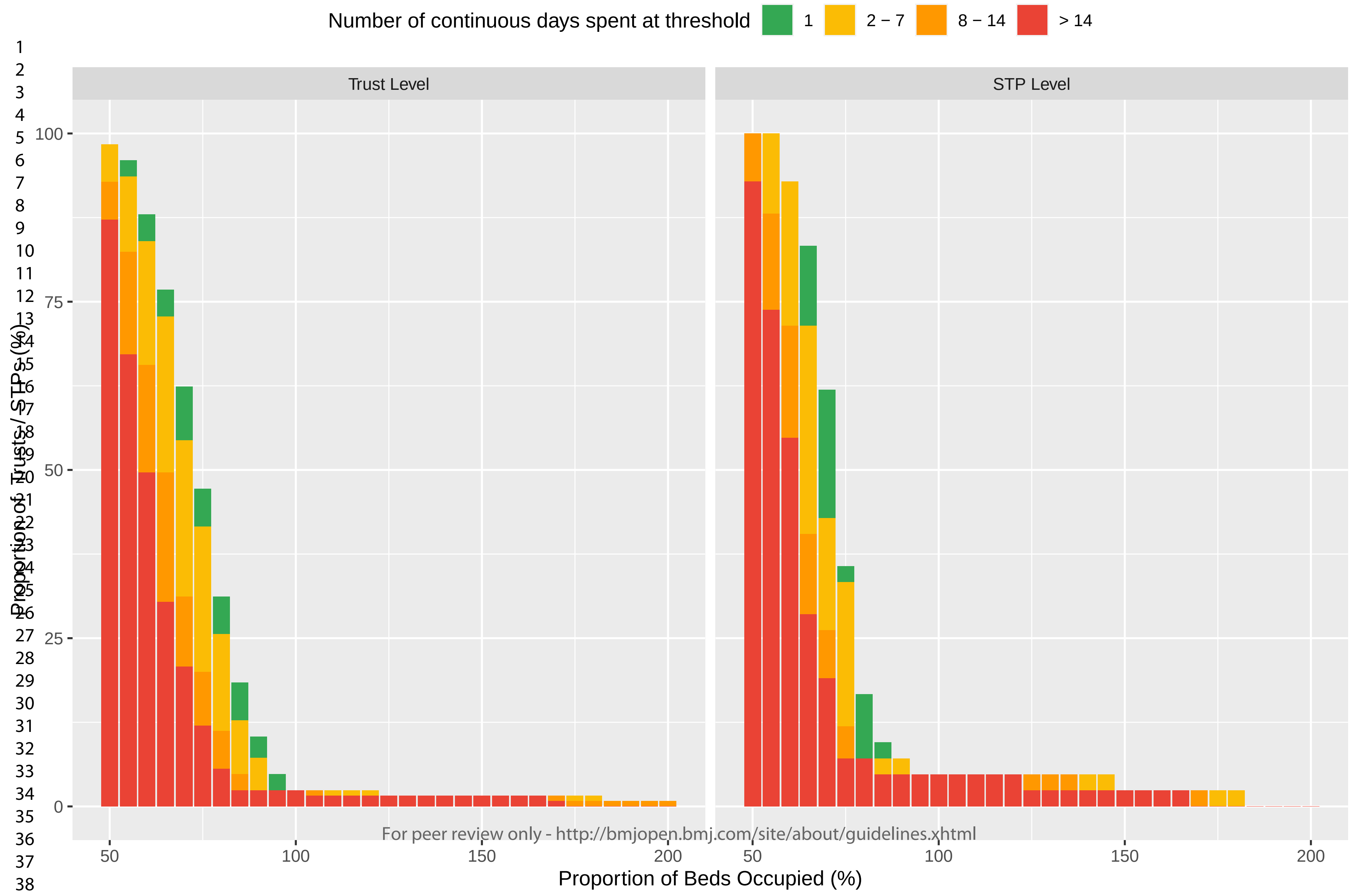

sFigure 5: Trust-Level General & Acute Bed Occupancy (Based on Baseline Capacities) Across England

Legend: The proportion of all trusts, and sustainability and transformation partnerships (STPs), at varying general and acute (G&A) bed occupancy thresholds relative to their baseline (mean availability January-March 2020) capacity, across England, from April 1st to June 5th. The superimposed colours represent how long the trusts spent at each specific threshold.

For peer review only

SFigure 6: Number of Trusts/STPs at Varying Ventilated Bed Occupancy Levels Compared to Baseline Capacity

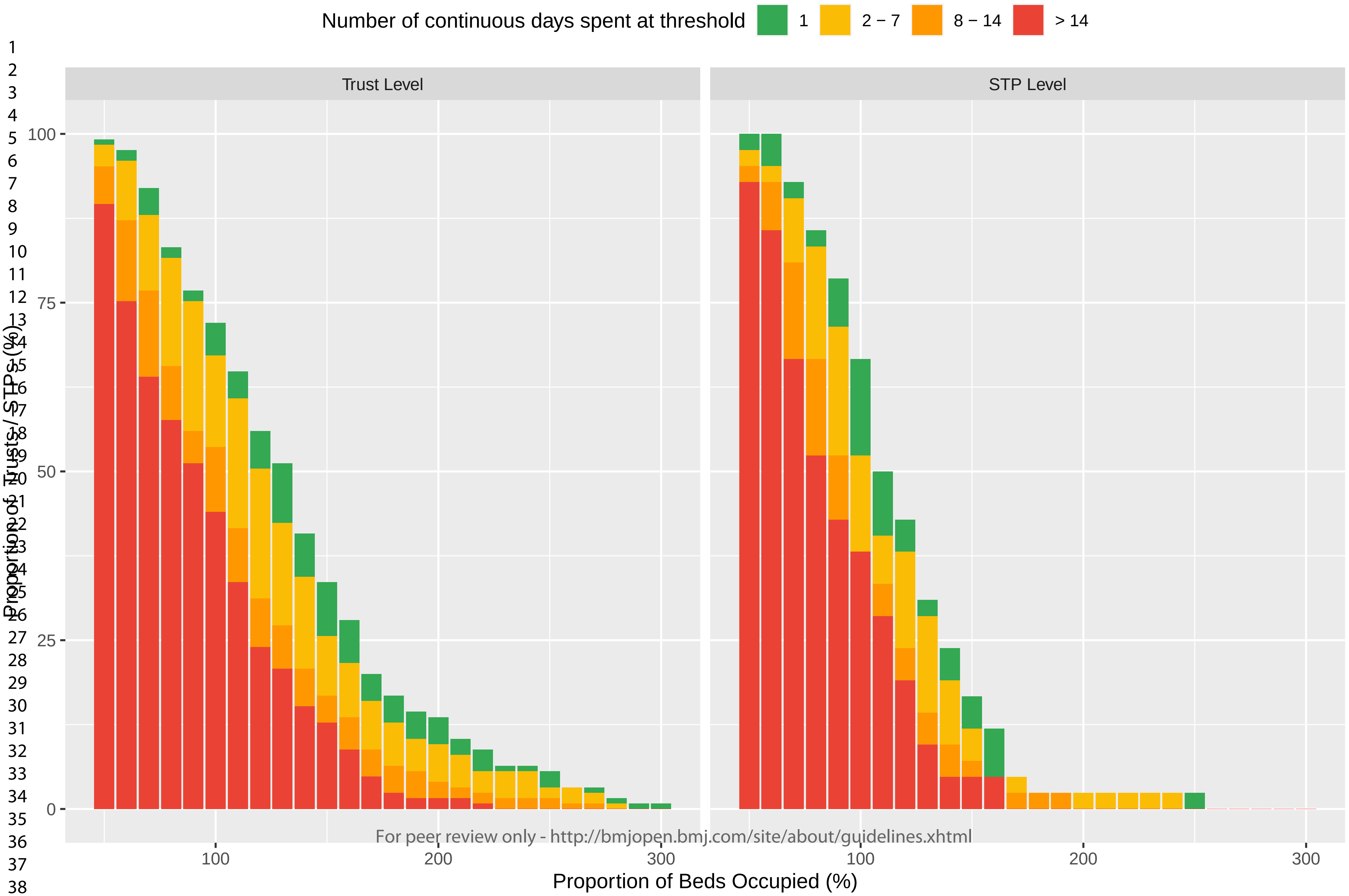

For peer review only - http://bmjopen.bmj.com/site/about/guidelines.xhtml

sFigure 6: Trust-Level Ventilator Bed Occupancy (Based on Baseline Capacities) Across England

Legend: The proportion of all trusts, and sustainability and transformation partnerships (STPs), at varying ventilator bed occupancy thresholds relative to their baseline capacity, across England, from April 1st to June 5th. The superimposed colours represent how long the trusts spent at each specific threshold.

For peer review only

SFigure 7: Regional Critical Care Bed Occupancy, Stratified by COVID-19 Status

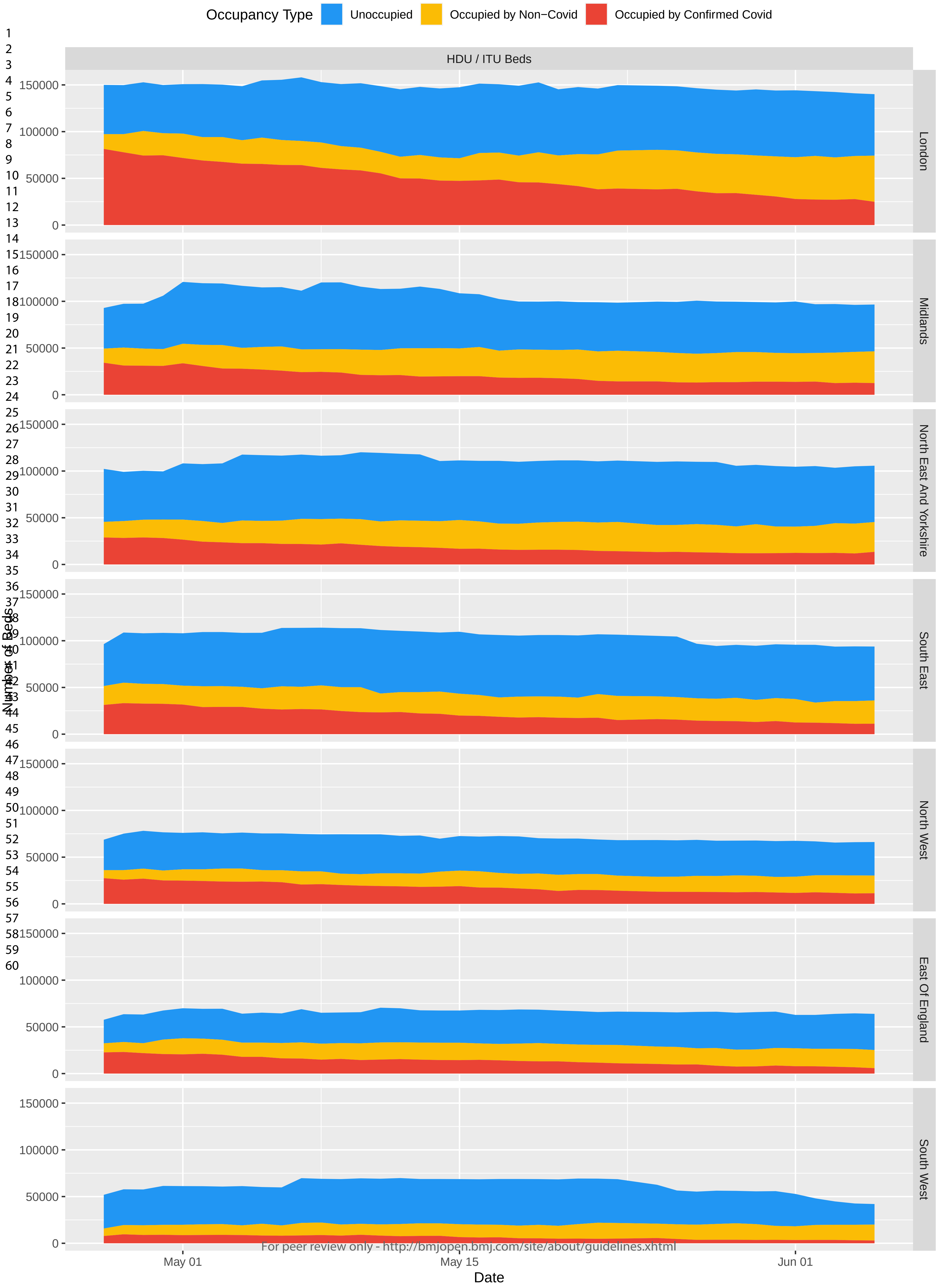

SFigure 7: Regional Critical Care Bed Occupancy, Stratified by COVID-19 Status

Legend: The time-varying trends in critical care bed capacity and occupancy across the 7 regions of England, from March 27th to June 5th. Occupancy is stratified by whether the individual in the bed has a positive COVID-19 test or not).

For peer review only

SFigure 8: Proportion of Trusts/STPs at Varying Critical Care Bed Occupancy Levels Compared to Baseline Capacity

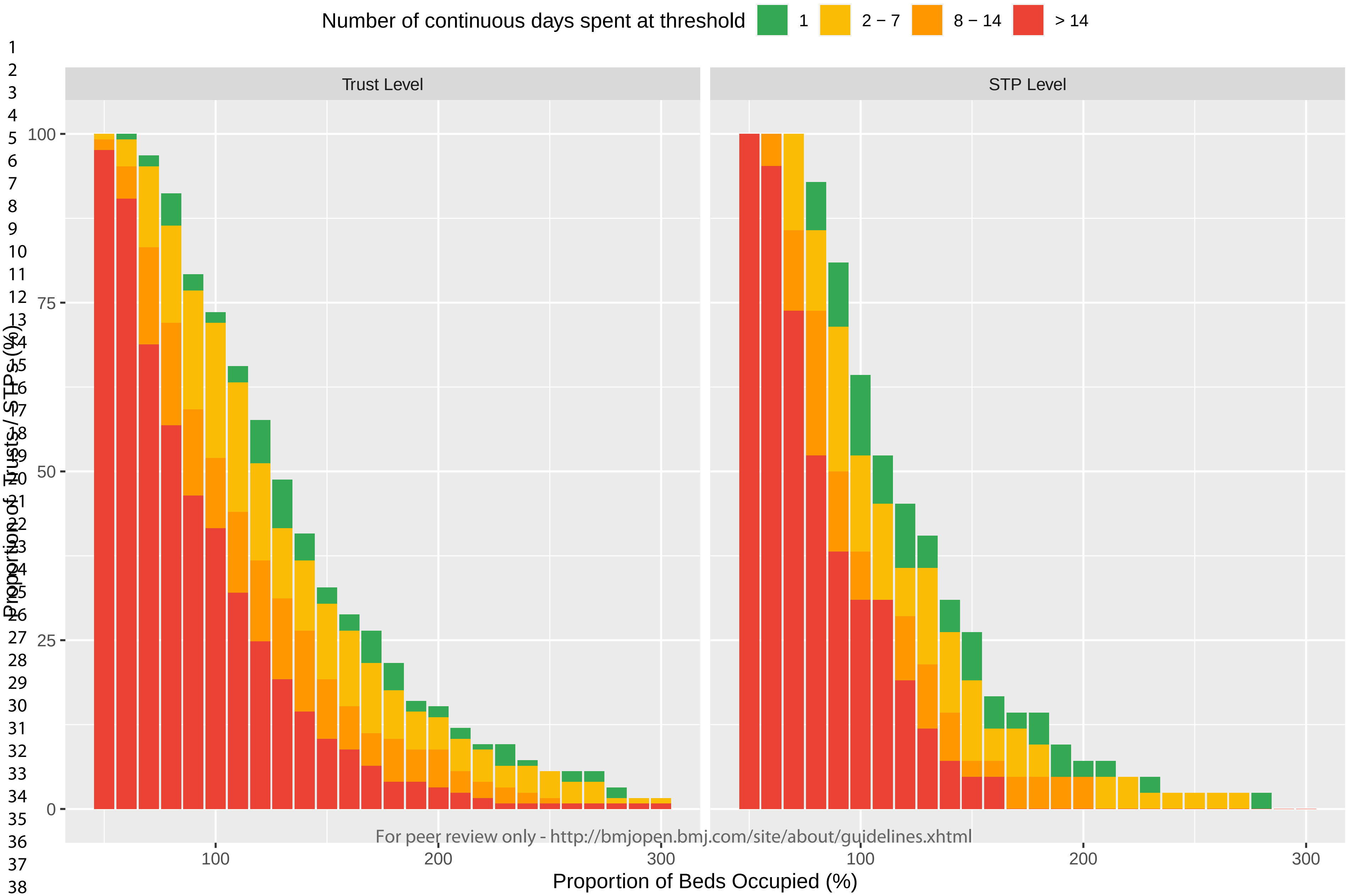

SFigure 8: Critical Care Bed Occupancy (Based on Baseline Capacities) Across England

Legend: SFigure 6A (Left) illustrates the proportion of trusts at different occupancy thresholds based on baseline critical care bed capacity, across England, from April 1st to June 5th. SFigure 6B (Right) illustrates the proportion of STPs at different occupancy thresholds based on their baseline critical care bed capacity, across England, from April 1st to June 5th. The superimposed colours represent how long the trusts spent at each specific threshold.

For peer review only

SFigure 9: Number of Trusts Operating Above Various Ventilated Bed Surge Capacity Thresholds

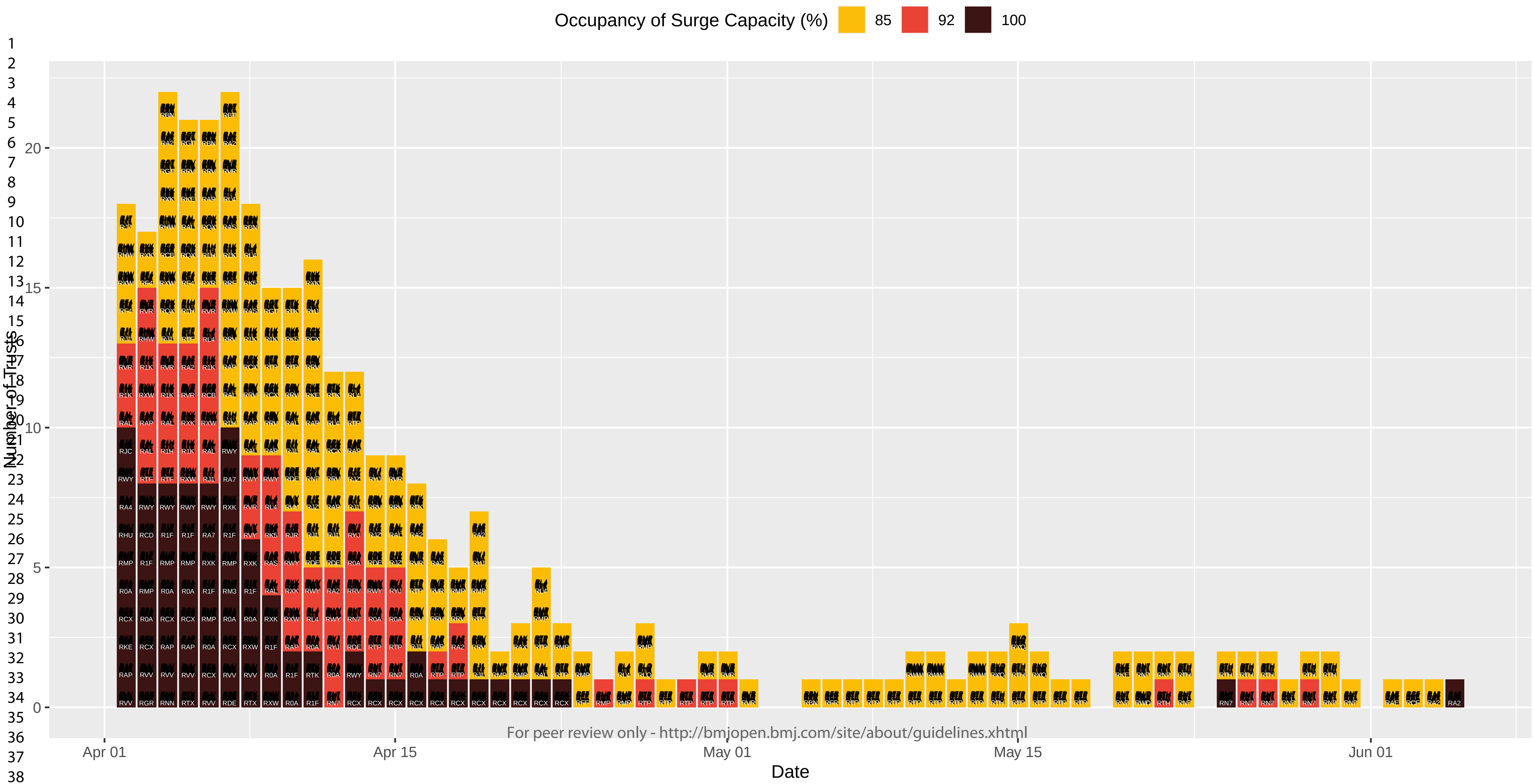

For peer review only - http://bmjopen.bmj.com/site/about/guidelines.xhtml

SFigure 9: Trust-Level Ventilator Bed Occupancy (Based on Surge Capacities) Across England

Legend: The conversion of trust code to name for all trusts included in the figure are: Manchester University NHS Foundation Trust (R0A), Isle Of Wight NHS Trust (R1F), Barts Health NHS Trust (R1H), London North West University Healthcare NHS Trust (R1K), Royal Surrey County Hospital NHS Foundation Trust (RA2), Yeovil District Hospital NHS Foundation Trust (RA4), University Hospitals Bristol NHS Foundation Trust (RA7), University Hospitals Bristol And Weston NHS Foundation Trust (RA7), Bradford Teaching Hospitals NHS Foundation Trust (RAE), Royal Free London NHS Foundation Trust (RAL), North Middlesex University Hospital NHS Trust (RAP), The Hillingdon Hospitals NHS Foundation Trust (RAS), Kingston Hospital NHS Foundation Trust (RAX), St Helens And Knowsley Teaching Hospitals NHS Trust (RBN), Mid Cheshire Hospitals NHS Foundation Trust (RBT), York Teaching Hospital NHS Foundation Trust (RCB), Harrogate And District NHS Foundation Trust (RCD), Airedale NHS Foundation Trust (RCF), The Queen Elizabeth Hospital, King's Lynn, NHS Foundation Trust (RCX), East Suffolk And North Essex NHS Foundation Trust (RDE), Barking, Havering And Redbridge University Hospitals NHS Trust (RF4), Barnsley Hospital NHS Foundation Trust (RFF), The Rotherham NHS Foundation Trust (RFR), West Suffolk NHS Foundation Trust (RGR), Cambridge University Hospitals NHS Foundation Trust (RGT), Portsmouth Hospitals NHS Trust (RHU), Royal Berkshire NHS Foundation Trust (RHW), Guy's And St Thomas' NHS Foundation Trust (RJ1), St George's University Hospitals NHS Foundation Trust (RJ7), South Warwickshire NHS Foundation Trust (RJC), Countess Of Chester Hospital NHS Foundation Trust (RJR), King's College Hospital NHS Foundation Trust (RJZ), Sherwood Forest Hospitals NHS Foundation Trust (RK5), Whittington Health NHS Trust (RKE), The Royal Wolverhampton NHS Trust (RL4), Wye Valley NHS Trust (RLQ), Salford Royal NHS Foundation Trust (RM3), Tameside And Glossop Integrated Care NHS Foundation Trust (RMP), Dartford And Gravesham NHS Trust (RN7), North Cumbria Integrated Care NHS Foundation Trust (RNN), Homerton University Hospital NHS Foundation Trust (RQX), Wrightington, Wigan And Leigh NHS Foundation Trust (RRF), University College London Hospitals NHS Foundation Trust (RRV), Northumbria Healthcare NHS Foundation Trust (RTF), Oxford University Hospitals NHS Foundation Trust (RTH), Ashford And St Peter's Hospitals NHS Foundation Trust (RTK), Surrey And Sussex Healthcare NHS Trust (RTP), University Hospitals Of Morecambe Bay NHS Foundation Trust (RTX), Epsom And St Helier University Hospitals NHS Trust (RVR), East Kent Hospitals University NHS Foundation Trust (RVV), Southport And Ormskirk Hospital NHS Trust (RVY), United Lincolnshire Hospitals NHS Trust (RWD), Warrington And Halton Hospitals NHS Foundation Trust (RWW), Calderdale And Huddersfield NHS Foundation Trust (RWY), Sandwell And West Birmingham Hospitals NHS Trust (RXK), Buckinghamshire Healthcare NHS Trust (RXQ), East Lancashire Hospitals NHS Trust (RXR), Shrewsbury And Telford Hospital NHS Trust (RXW), Imperial College Healthcare NHS Trust (RYJ)

For peer review only

SFigure 10a: Proportion of Hospitals/Trusts/STPs at Varying Ventilated Bed Occupancy Levels Compared to Surge Capacity

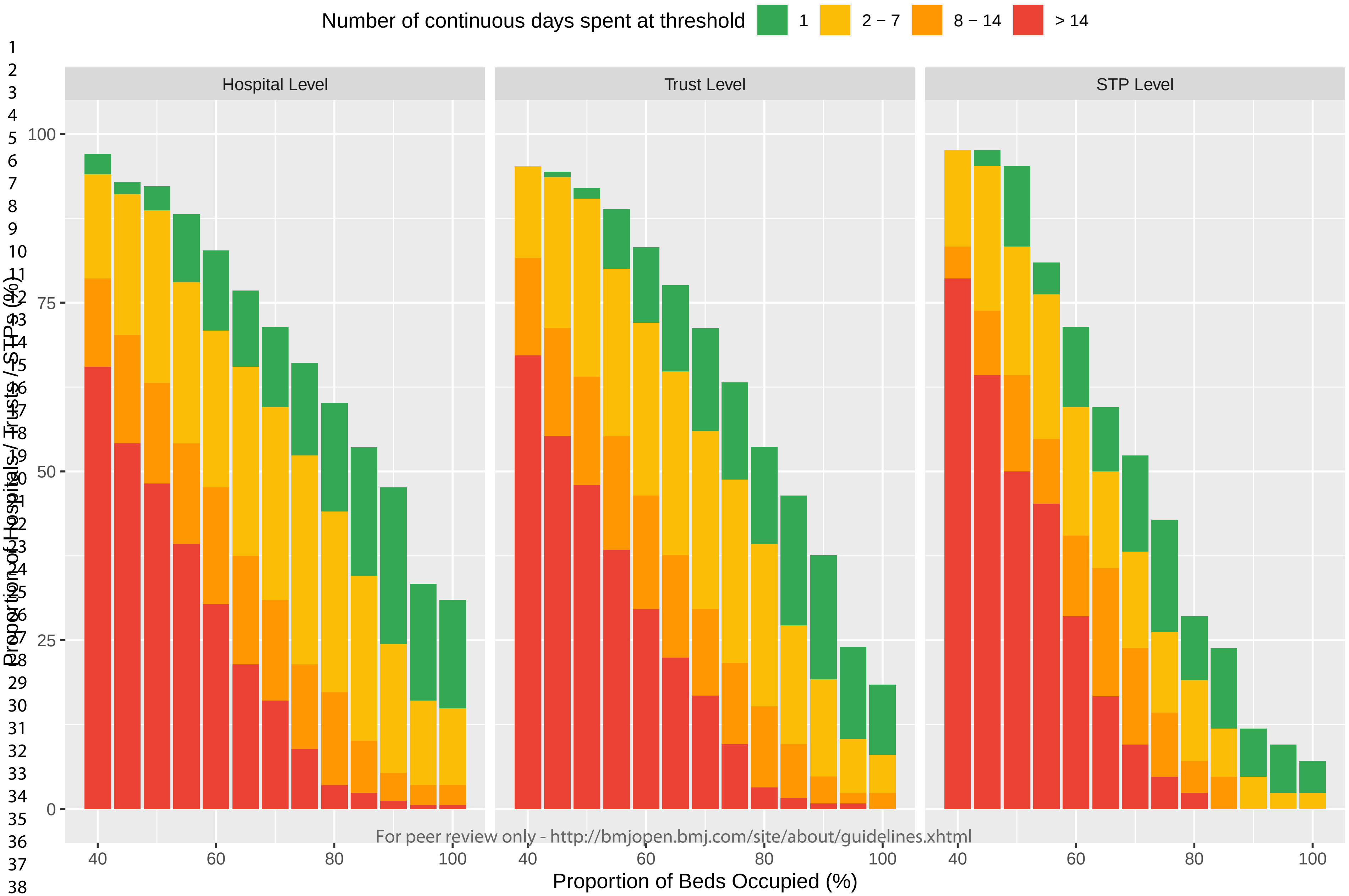

For peer review only - http://bmjopen.bmj.com/site/about/guidelines.xhtml

SFigure 10b: Proportion of Hospitals/Trusts/STPs at Varying G&A Bed Occupancy Levels Compared to Surge Capacity

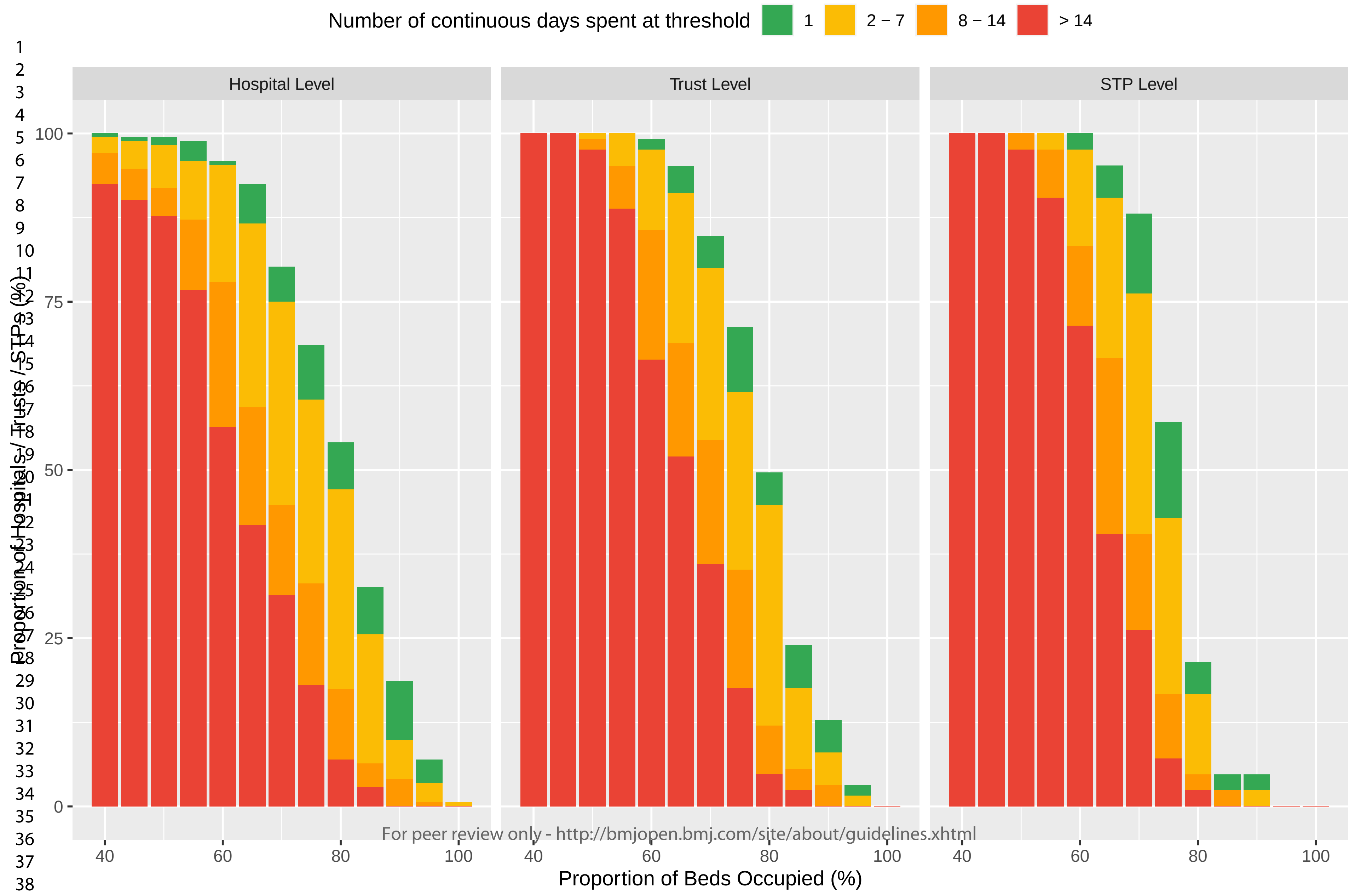

For peer review only - http://bmjopen.bmj.com/site/about/guidelines.xhtml

SFigure 10: Mechanical Ventilator Beds (Top) & General and Acute (Bottom) Occupancy (Based on Surge Capacities) Across England

Legend: SFigure 8A (Top) illustrates the proportion of STPs at different occupancy thresholds for surge mechanical ventilator bed capacity, across England. SFigure 8B (Bottom) illustrates the proportion of STPs at different occupancy thresholds for surge general and acute capacity, across England. The superimposed colours represent how long the trusts spent at each specific threshold.

For peer review only

SFigure 11: Ribbon Plots of STP Minimum and Maximum Occupancy

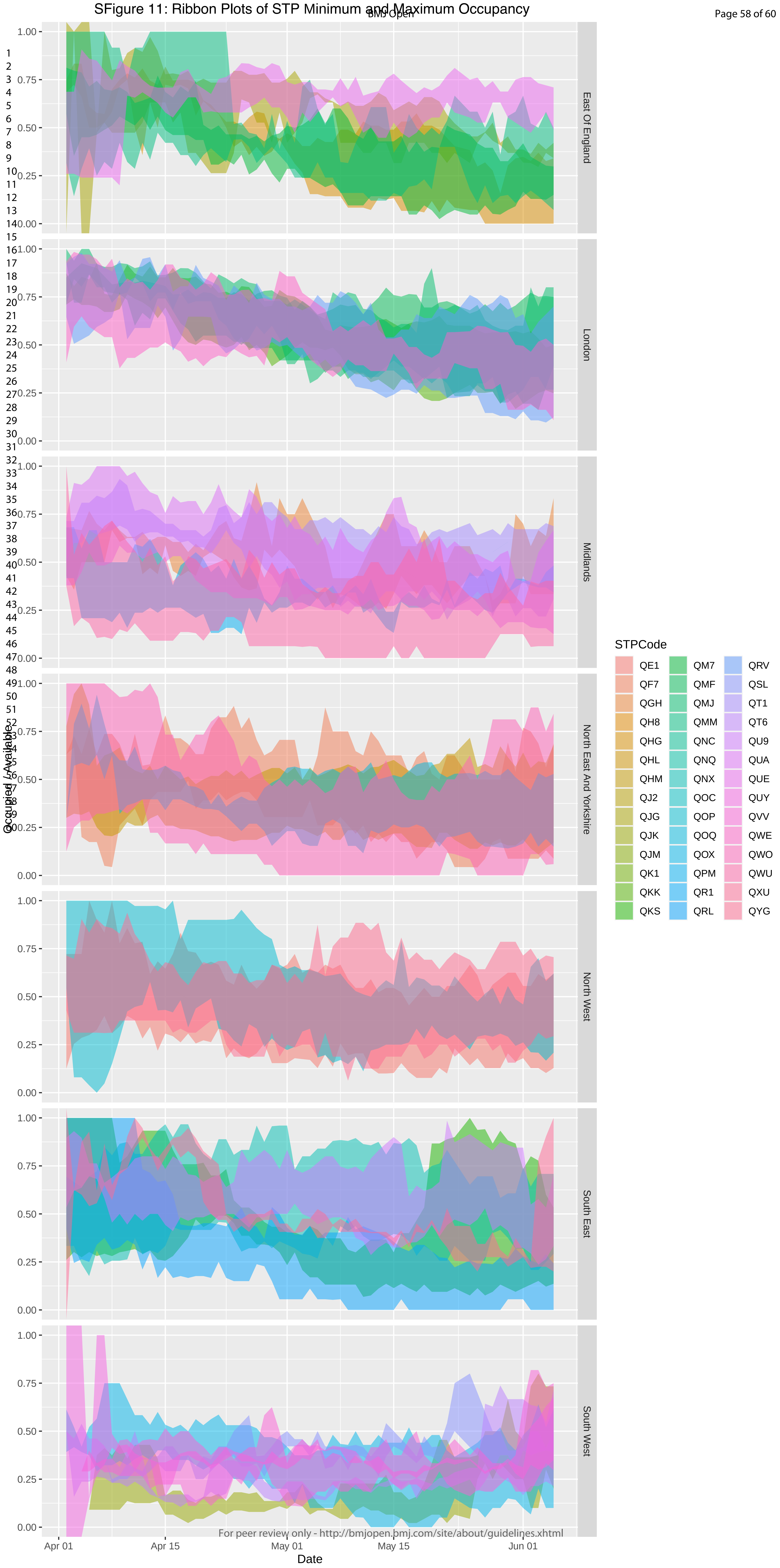

For peer review only - http://bmjopen.bmj.com/site/about/guidelines.xhtml

Supplementary Video 1

[See attached link for time-lapse of G&A bed capacity at the STP level across England]

Supplementary Video 2

[See attached link for time-lapse of mechanical ventilator bed capacity at the STP level across England]

Supplementary References

35 King's F. Sustainability and transformation plans (STPs) explained. 2017. https://www.kingsfund.org.uk/topics/integrated-care/sustainability-transformation-plans-explained.

36 Public Health England. COVID-19: investigation and initial clinical management of possible cases. 2020; published online Jan 10. https://www.gov.uk/government/publications/wuhan-novel-coronavirus-initial-investigation-of-possible-cases/investigation-and-initial-clinical-management-of-possible-cases-of-wuhan-novel-coronavirus-wn-cov-infection (accessed June 23, 2020).

37 Statistics. Statistics » Bed Availability and Occupancy Data – Overnight. https://www.england.nhs.uk/statistics/statistical-work-areas/bed-availability-and-occupancy/bed-data-overnight/. (accessed June 23, 2020).

38 Statistics. Statistics » Critical Care Bed Capacity and Urgent Operations Cancelled. https://www.england.nhs.uk/statistics/statistical-work-areas/critical-care-capacity (accessed June 23, 2020).

39 England NHS. NHS England » NHS steps up coronavirus fight with two more Nightingale Hospitals. https://www.england.nhs.uk/2020/04/nhs-steps-up-coronavirus-fight-with-two-more-nightingale-hospitals/ (accessed June 23, 2020).

40 England NHS. NHS England » NHS to build more Nightingale Hospitals, as London set for opening. https://www.england.nhs.uk/2020/04/nhs-to-build-more-nightingale-hospitals-as-london-set-for-opening/ (accessed June 23, 2020).

For peer review only

STROBE Statement—checklist of items that should be included in reports of observational studies

	Item No	Recommendation	Page No
**Title and abstract**	1	(*a*) Indicate the study's design with a commonly used term in the title or the abstract	1
		(*b*) Provide in the abstract an informative and balanced summary of what was done and what was found	2
**Introduction**			
Background/rationale	2	Explain the scientific background and rationale for the investigation being reported	4
Objectives	3	State specific objectives, including any prespecified hypotheses	4
**Methods**			
Study design	4	Present key elements of study design early in the paper	5
Setting	5	Describe the setting, locations, and relevant dates, including periods of recruitment, exposure, follow-up, and data collection	5
Participants	6	(*a*) *Cohort study*—Give the eligibility criteria, and the sources and methods of selection of participants. Describe methods of follow-up	5-6
		(*b*) *Cohort study*—For matched studies, give matching criteria and number of exposed and unexposed	NA
Variables	7	Clearly define all outcomes, exposures, predictors, potential confounders, and effect modifiers. Give diagnostic criteria, if applicable	6
Data sources/ measurement	8*	For each variable of interest, give sources of data and details of methods of assessment (measurement). Describe comparability of assessment methods if there is more than one group	*5-6*
Bias	9	Describe any efforts to address potential sources of bias	Supplementary material; quality control
Study size	10	Explain how the study size was arrived at	5
Quantitative variables	11	Explain how quantitative variables were handled in the analyses. If applicable, describe which groupings were chosen and why	5 & Supplementary material; quality control
Statistical methods	12	(*a*) Describe all statistical methods, including those used to control for confounding	6
		(*b*) Describe any methods used to examine subgroups and interactions	6 & Supplementary material; statistical analysis
		(*c*) Explain how missing data were addressed	Supplementary material; quality control
		(*d*) *Cohort study*—If applicable, explain how loss to follow-up was addressed	NA
		(*e*) Describe any sensitivity analyses	6

For peer review only - http://bmjopen.bmj.com/site/about/guidelines.xhtml

**Results**			
Participants	13*	(a) Report numbers of individuals at each stage of study—eg numbers potentially eligible, examined for eligibility, confirmed eligible, included in the study, completing follow-up, and analysed	7-15
		(b) Give reasons for non-participation at each stage	7
		(c) Consider use of a flow diagram	NA
Descriptive data	14*	(a) Give characteristics of study participants (eg demographic, clinical, social) and information on exposures and potential confounders	Supplementary table 3
		(b) Indicate number of participants with missing data for each variable of interest	Supplementary material; quality control
		(c) *Cohort study*—Summarise follow-up time (eg, average and total amount)	7-8
Outcome data	15*	*Cohort study*—Report numbers of outcome events or summary measures over time	*7-8*
Main results	16	(*a*) Give unadjusted estimates and, if applicable, confounder-adjusted estimates and their precision (eg, 95% confidence interval). Make clear which confounders were adjusted for and why they were included	NA
		(*b*) Report category boundaries when continuous variables were categorized	NA
		(*c*) If relevant, consider translating estimates of relative risk into absolute risk for a meaningful time period	NA
Other analyses	17	Report other analyses done—eg analyses of subgroups and interactions, and sensitivity analyses	Supplementary material; Critical care
**Discussion**			
Key results	18	Summarise key results with reference to study objectives	9
Limitations	19	Discuss limitations of the study, taking into account sources of potential bias or imprecision. Discuss both direction and magnitude of any potential bias	9-10
Interpretation	20	Give a cautious overall interpretation of results considering objectives, limitations, multiplicity of analyses, results from similar studies, and other relevant evidence	10-11
Generalisability	21	Discuss the generalisability (external validity) of the study results	11
**Other information**			
Funding	22	Give the source of funding and the role of the funders for the present study and, if applicable, for the original study on which the present article is based	No Funding

For peer review only
